# Global, regional, and national temporal trends in prevalence for nasopharynx cancer across adolescents and young adults, 1990–2021: an age-period-cohort analysis based on the global burden of disease study 2021

**DOI:** 10.1186/s12903-025-06750-4

**Published:** 2025-09-26

**Authors:** Yanhua Tian, Wenbo Sun, Jijun Song

**Affiliations:** 1 Department of Otorhinolaryngology, Zhoukou Central Hospital, Zhoukou Medical Science Research Center, Zhoukou, 466000 Henan P. R. China; 2 Department of Pharmacy, Zhoukou Central Hospital, Zhoukou Medical Science Research Center, Zhoukou, Henan China

**Keywords:** Nasopharynx cancer, Adolescents and young adults, Prevalence, Age-period-cohort analysis, Global burden of disease

## Abstract

**Background:**

Nasopharynx cancer (NPC) is a highly aggressive malignancy that is relatively rare on a global scale but exhibits marked geographic clustering, with high incidence in regions such as Southeast Asia, North Africa, and the Middle East. However, comprehensive global epidemiological assessments remain limited. This study analyzed temporal trends in the prevalence of NPC among adolescents and young adults (AYAs) from 1990 to 2021 using an age-period-cohort (APC) model.

**Methods:**

This study utilized data from the Global Burden of Disease 2021. The study population comprised individuals aged 15–39 years. Age-standardized prevalence rates (ASPR) were calculated, with net and local drift used to assess overall and age-specific trends. The APC model was employed to separate the independent effects of age, period, and birth cohort on changes in NPC prevalence.

**Results:**

From 1990 to 2021, the number of NPC cases among AYAs globally increased by 89.16%, reaching 128278.84 cases in 2021, although the ASPR rose only slightly (+0.21%/year, 95% CI: −0.02 to 0.44). A marked increase in ASPR was observed in the high-middle SDI region (+1.70%/year, 95% CI: 1.30 to 2.11), contrasting with declining trends in high SDI and low SDI regions. The age effect demonstrated increasing NPC prevalence with age, peaking in the 35–39 age group. Period effects revealed a resurgence in NPC risk after globally 2010, particularly in rapidly industrializing high-middle SDI regions. Birth cohort effects improved in high SDI regions but did not show significant improvement in high-middle SDI regions.

**Conclusion:**

NPC among AYAs shows divergent regional trends, with sharp increases observed in high-middle SDI regions, suggesting a need for further investigation into region-specific risk patterns and public health responses to address the growing burden, particularly in resource-limited settings.

**Supplementary Information:**

The online version contains supplementary material available at 10.1186/s12903-025-06750-4.

## Introduction

Nasopharyngeal cancer (NPC) is a malignant tumor arising from the epithelial lining of the nasopharynx, characterized by distinctive geographical distribution patterns and biological features, with a notably aggressive and metastatic behavior [1]. Although NPC remains relatively rare on a global scale, it exhibits marked geographic clustering in regions such as southern China, Southeast Asia, and parts of the Middle East and North Africa, where it poses a significant regional public health challenge [2]. Unlike other head and neck malignancies, NPC is etiologically linked to a complex interplay of factors, including Epstein–Barr virus (EBV) infection [3, 4], genetic susceptibility [5], and environmental exposures such as consumption of preserved foods and tobacco use [5]. While considerable progress has been made in understanding the epidemiology and implementing interventions for NPC among adults and older populations, the disease burden among adolescents and young adults (AYAs) remains under-characterized, particularly with respect to cross-national trends and long-term temporal patterns [6].

The AYAs’ period represents a critical transitional phase from dependence to independence, during which health status profoundly influences educational attainment, social engagement, reproductive health, and ultimately, long-term disease burden and economic productivity [7]. Recognizing the importance of this life stage, the World Health Organization and several national health authorities have prioritized AYAs’ health within chronic disease prevention frameworks [8]. The 2024 clinical practice guidelines on adolescent and young adult oncology issued by the U.S. National Comprehensive Cancer Network also emphasize that AYAs constitute a distinct age group with unique medical and psychosocial needs [9]. In this population, NPC may exhibit distinct biological behaviors. For instance, dynamic fluctuations in sex hormone levels during puberty and early adulthood may trigger reactivation of latent EBV infections [10]. Concurrently, the rising prevalence of electronic cigarette use among AYAs has emerged as a potential oncogenic exposure [11, 12]. Some studies suggest that NPC in AYAs may display heightened biological aggressiveness, increased radiosensitivity, and greater heterogeneity in survival outcomes compared to older populations [13–16].

Although data from the Global Burden of Disease (GBD) study indicate that the age-standardized incidence rate of NPC declined at an average annual rate of 1.06% between 1990 and 2021, while the age-standardized prevalence rate (ASPR) increased marginally at 0.07% per year [17], these aggregate trends may obscure age-specific dynamics, particularly within the AYAs population. Existing studies have largely been descriptive in nature and have not adequately disentangled the respective contributions of age, period, and birth cohort effects to the observed burden of NPC.

The GBD dataset, with its standardized methodology and broad spatiotemporal coverage, serves as an essential resource for global health research [18]. The age–period–cohort (APC) model offers a robust analytical framework to disentangle the independent contributions of age, period, and cohort effects, thereby providing critical insights into the temporal dynamics of disease burden. Leveraging data from GBD 2021 and the APC modeling approach, this study aimed to: (1) delineate the spatiotemporal trends in the prevalence of NPC among AYAs at global, regional, and national levels from 1990 to 2021; (2) quantify the relative contributions of age, period, and cohort effects to the evolving epidemiological patterns of NPC; and (3) assess disparities in NPC burden across countries with varying levels of the Socio-demographic Index (SDI). The findings of this study are expected to inform early screening strategies, optimize resource allocation, and guide policy prioritization for NPC control in the AYAs population.

## Methods

### Data and data source

This study utilized data from the GBD 2021, a comprehensive public health resource. GBD 2021 provides standardized and comparable data on disease burden across 204 countries and territories from 1990 to 2021. It encompasses a wide range of health indicators, including incidence, prevalence, mortality, and disability-adjusted life years (DALYs), for various diseases, injuries, and risk factors [18].

To ensure data consistency and comparability, GBD 2021 uses a Bayesian meta-regression modeling tool, DisMod-MR 2.1, to ensure internal consistency and cross-country comparability of estimates. This model synthesizes diverse data sources (e.g., population censuses, household surveys, medical records, verbal autopsies) and makes assumptions to adjust for data quality issues, biases, and missing data across different locations and time points.

For this study, we specifically extracted data related to NPC among AYAs aged 15–39 years. The extracted data included the number of prevalent cases and age-specific prevalence rates at global, regional, and national levels from 1990 to 2021. Additionally, corresponding population data were extracted to facilitate the construction of an APC model and the analysis of temporal trends.

All necessary GBD 2021 data were retrieved on May 24, 2025, using the Global Health Data Exchange query tool (https://vizhub.healthdata.org/gbd-results/). The specific data files accessed included estimates of NPC prevalence and the number of prevalent cases stratified by global, regional, national, age group, and year, as well as the percentage change in NPC prevalent cases from 1990 to 2021.

NPC codes used in the GBD 2021 come from two different sets of rules: the International Classification of Diseases, Ninth Revision (ICD-9), and ICD-10. Specifically, the ICD-9 codes range from 147 to 147.9, while the ICD-10 codes cover C11-C11.9 [18].

The SDI serves as a composite measure integrating three key components: lag-distributed income per capita, the average educational attainment in years, and the total fertility rate among females under 25 years of age. This index captures the socioeconomic and demographic factors that shape health outcomes across different regions, with higher SDI values signifying more advanced socioeconomic development [19]. According to the GBD 2021 framework, the 204 countries and territories analyzed are classified into five SDI quintiles: high, high-middle, middle, low-middle, and low, based on their respective index values.

### Study population

AYAs are defined as individuals aged between 15 and 39 years [9, 20]. To capture the heterogeneity of NPC prevalence across distinct developmental stages and sociobehavioral transitions within this age group, we further stratified AYAs into five subgroups based on the age classification used in the GBD 2021 database: 15–19, 20–24, 25–29, 30–34, and 35–39 years.

### Analysis of overall temporal trends

To assess temporal trends in NPC across AYAs population from 1990 to 2021, the ASPR and its corresponding 95% uncertainty intervals (UIs) were utilized, with all rates expressed per 100,000. The formula for calculating ASPR was as follows:

$$\:ASPR=\frac{\sum\:_{i=1}^{A}\:(ai\times\:wi)}{\sum\:_{i=1}^{A}\:wi}\times\:100,000$$

Where $\:ai$ represents the age-specific prevalence rate for the $\:ith$ age group, $\:wi$ denotes the standard population weight for the same age group, and *A* is the total number of age groups. This approach ensures comparability across different populations and time periods by accounting for variations in age structure.

In terms of prevalence counts, the study calculated the relative proportion of NPC cases across five age groups (15−19, 20−24, 25−29, 30−34, and 35−39 years). The analysis also illustrated the temporal trends in the age distribution of NPC prevalence counts over time.

### Uncertainty interval

In GBD 2021, uncertainty analysis is conducted for all final estimates to generate 95% UIs, which are completed by the 2.5th and 97.5th percentile values from 500 draws, reflecting the uncertainty in the estimates. The estimation results are calculated based on data from global, seven super-regional, 21 regional, 204 national, and 811 subnational locations and are analyzed from 1990 to 2021 [21].

### Age-period-cohort analysis

To elucidate the long-term epidemiological trends of NPC among AYAs and identify its underlying drivers, we employed a classical APC modelling approach. This method decomposes temporal variations in disease prevalence into three distinct effects: the age effect, reflecting physiological changes associated with individual aging; the period effect, capturing the influence of time-specific social and environmental exposures; and the cohort effect, indicating risk patterns linked to conditions experienced by individuals born in the same time period [22]. Unlike conventional linear trend analyses, the APC model enables the identification of dynamic shifts in disease burden across population structures and sociocultural transitions, thereby offering a valuable framework for the development of targeted prevention and control strategies.

The APC analysis was conducted using data from the GBD 2021, with stratification by five-year age groups (15–19, 20–24, 25–29, 30–34, and 35–39 years) and six consecutive calendar periods (1992–1996, 1997–2001, 2002–2006, 2007–2011, 2012–2016, and 2017–2021). Based on these parameters, ten equally spaced birth cohorts were derived: 1952–1961, 1957–1966, 1962–1971, 1967–1976, 1972–1981, 1977–1986, 1982–1991, 1987–1996, 1992–2001, and 1997–2006.

Given the intrinsic linear dependency among age, period, and cohort (birth cohort = period − age), it is not feasible to simultaneously estimate all three effects in a traditional regression framework due to the issue of perfect collinearity. To address this identification problem, we adopted the estimable function approach within a log-linear Poisson regression framework. This model treats the number of prevalent cases as the outcome variable and incorporates the population size as an offset term to estimate the independent effects of age, period, and cohort. Following the identifiability principles established by Holford and Clayton et al. [23, 24], the model decomposes the temporal trends into a linear component—termed “drift”—and nonlinear deviations—referred to as “effect curves.” This allows for the derivation of statistically identifiable parameters, including net drift, local drift, and relative risks (RRs), which capture the underlying dynamics of disease burden across time and demographic strata.

*Net drift* represents the average annual percentage change in ASPR across the entire study population, capturing the overall temporal trend in disease burden. *Local drift* reflects the annual percentage change in ASPR within each specific age group and is instrumental in identifying age strata with rapidly increasing or decreasing prevalence. The statistical significance of these trends was assessed using Wald chi-square tests [25]. Furthermore, we computed the RR curves corresponding to period and cohort effects, along with their 95% confidence intervals, to elucidate the specific contributions of calendar time and birth cohort to NPC risk. RRs were calculated as the ratio of each period or cohort’s risk relative to a designated reference category. The choice of the reference period or birth cohort is made arbitrarily and does not affect the interpretation of the results. All general statistical analyses were performed using R software (version 4.4.2, R Foundation for Statistical Computing, Vienna, Austria). APC analysis was conducted using the official web-based APC analysis tool provided by the US National Cancer Institute (NCI) (https://analysistools.cancer.gov/apc/) [25].

## Results

### Trends in NPC ASPR across AYAs population, 1990 − 2021

Table 1 presents data on population size, number of prevalent cases, ASPR, and *net drift* of NPC among AYAs globally and across SDI regions from 1990 to 2021. Globally, the number of prevalent NPC cases among AYAs increased by 89.16% (95% CI: 56.65 to 133.02) over the past 32 years, reaching 128,278.84 cases (95% UI: 107,263.42–154,886.09) in 2021. While all SDI regions except the high SDI region showed statistically significant increases in prevalent cases. Notably, although the proportion of the global population residing in high-middle SDI regions declined from 19.94% in 1990 to 16.52% in 2021, the proportion of NPC cases in this region increased markedly from 34.07 to 45.38%, a 151.92% increase in case counts (95% CI: 85.91 to 252.90). In 2021, the global ASPR for NPC among AYAs was 4.19 per 100,000 population (95% UI: 3.51–5.06), representing a 28.13% increase since 1990. The APC model estimated a non-significant global *net drift* in ASPR of 0.21% per year (95% CI: −0.02 to 0.44). Among the five SDI regions, only high SDI and low SDI regions exhibited declining ASPR, with *net drift* estimates of − 0.25% (95% CI: −0.42 to −0.07) and −0.52% (95% CI: −0.71 to −0.33), respectively. In contrast, the high-middle SDI region demonstrated the most pronounced increase, with a *net drift* of 1.70% (95% CI: 1.30 to 2.11).

**Table 1:** Trends of nasopharynx cancer across adolescents and young adults from 1990 to 2021 by SDI quintiles

	Global		High SDI		High-middle SDI		Middle SDI		Low-middle SDI		Low SDI	
	1990	2021	1990	2021	1990	2021	1990	2021	1990	2021	1990	2021
Population												
No (×10^6^)	5333.62 (5231.04, 544.65)	7891.35 (7666.73, 8131.22)	879.53 (857.73, 901.20)	1094.05 (1063.56, 1125.00)	1063.53 (1028.13, 1100.54)	1304.03 (1251.36, 1359.98)	1722.91 (1667.96, 1774.14)	2448.54 (2353.58, 2542.11)	1161.41 (1120.14, 1200.68)	1921.11 (1821.41, 2023.27)	501.30 (487.91, 514.76)	1117.38 (1068.35, 1166.40)
Percentage of global level (%)	100	100	16.49	13.86	19.94	16.52	32.30	31.03	21.78	24.34	9.40	14.16
Prevalence												
No.	67815.15 (59354.04, 76997.69)	128278.84 (107263.42, 154886.09)	9591.04 (8877.14, 10387.6)	10425.03 (9298.01, 11715.11)	23106.60 (18484.61, 29023.35)	58209.32 (42240.54, 79610.61)	27964.21 (23774.25, 32670.48)	45614.93 (38227.07, 54358.05)	5296.03 (4376.61, 6407.91)	9895.64 (8112.68, 11975.37)	1838.02 (1438.06, 2318.04)	4099.30 (3167.85, 5343.89)
Percentage of global level (%)	100	100	14.14	8.13	34.07	45.38	41.24	35.56	7.81	7.71	2.71	3.20
Percentage change (%)	89.16 (56.65, 133.02)		8.70 (−2.85, 21.41)		151.92 (85.91, 252.90)		63.12 (32.36, 104.30)		86.85 (57.00, 136.29)		123.03 (82.83, 187.73)	
Age-standardised prevalence rate												
Rate per 100,000	3.27 (2.86, 3.72)	4.19 (3.51, 5.06)	2.66 (2.46, 2.88)	2.69 (2.41, 3.02)	5.15 (4.12, 6.47)	11.34 (8.24, 15.51)	4.09 (3.47, 4.77)	4.63 (3.88, 5.52)	1.29 (1.06, 1.56)	1.27 (1.04, 1.54)	1.11 (0.87, 1.41)	1.01 (0.78, 1.32)
APC model estimates												
* Net drift* of prevalence (% per year)	0.21 (−0.02 to 0.44)		−0.25 (−0.42 to −0.07)		1.70 (1.30 to 2.11)		0.22 (0.01 to 0.43)		−0.05 (−0.17 to 0.07)		−0.52 (−0.71 to −0.33)	

Parentheses for GBD estimates denote 95% uncertainty intervals and parentheses for *net drift* denote 95% CIs

APC, age period cohort; GBD, Global Burden of Diseases; SDI, sociodemographic index

**Fig. 1:**
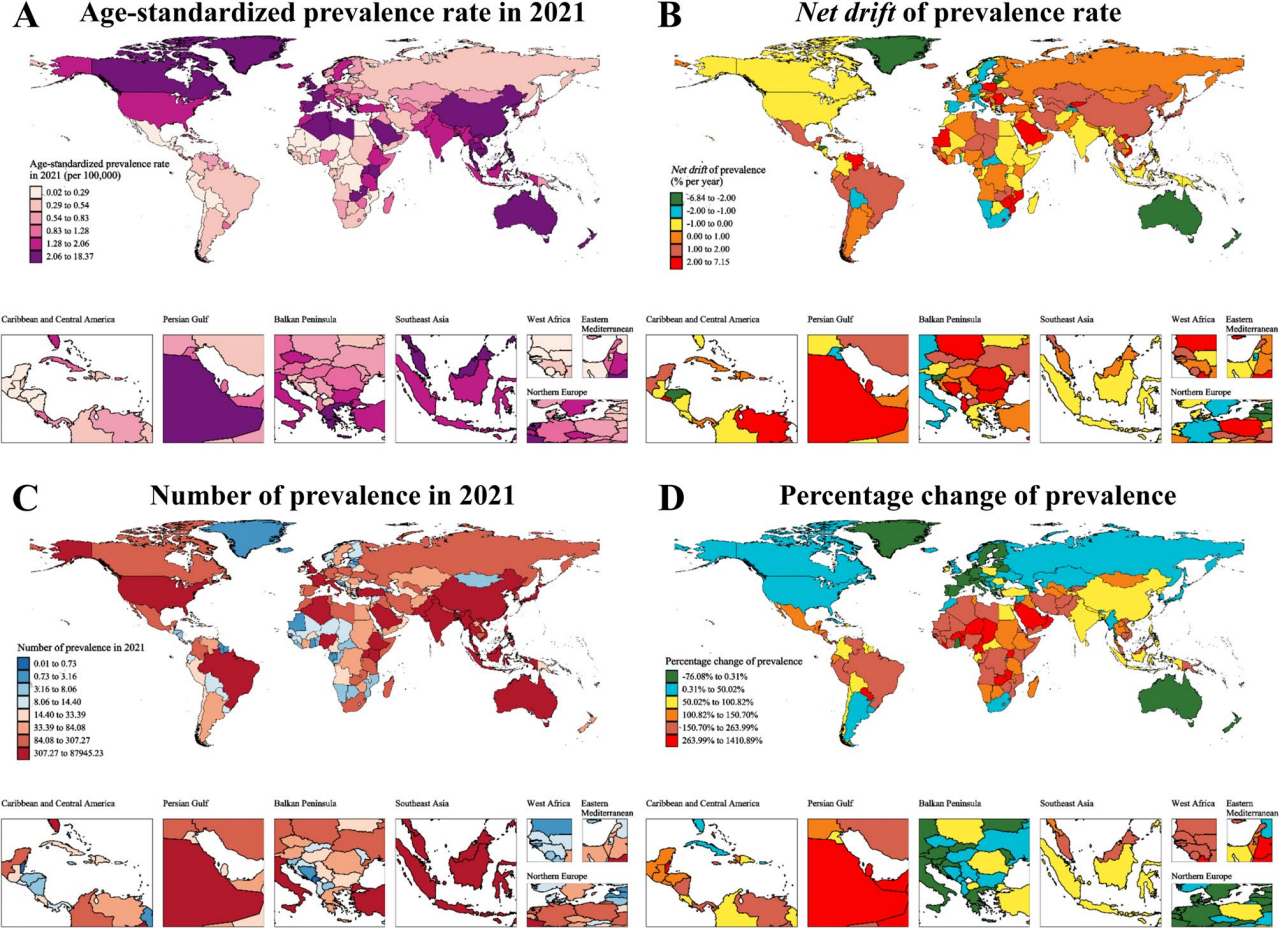
The age-standardized prevalence rate, *net drift*, number of cases, and percentage changes of nasopharynx cancer across adolescents and young adults in 204 countries and regions in 2021. (A) Map showing the age-standardized prevalence rate of nasopharynx cancer across adolescents and young adults in 2021. (B) Map showing the *net drift* of age-standardized prevalence rate of nasopharynx cancer across adolescents and young adults from 1990 to 2021. (C) Map showing the number of prevalence of nasopharynx cancer across adolescents and young adults in 2021. (D) Map showing the percentage changes of prevalent cases of nasopharynx cancer across adolescents and young adults

Figure 1 and Table S1 present the ASPR and case counts of NPC among AYAs across countries and regions in 2021, along with temporal trends in disease burden from 1990 to 2021. In 2021, six countries—China, India, Viet Nam, the United States of America, Indonesia, and Malaysia—each reported over 2,000 NPC cases in the AYA population. China ranked first with 87,945.23 cases, substantially exceeding India, which reported 8,074.50 cases. Compared to 1990, the number of NPC cases among AYAs increased by 100.82% (95% CI: 51.04 to 166.16) in China, 76.58% (95% CI: 46.88 to 116.36) in India, 195.67% (95% CI: 87.10 to 369.69) in Viet Nam, 0.40% (95% CI: −6.86 to 8.33) in the United States, 67.99% (95% CI: 19.69 to 137.05) in Indonesia, and 201.94% (95% CI: 117.31 to 338.21) in Malaysia. Collectively, these countries accounted for 82.81% of the global NPC burden among AYAs. With respect to ASPR, 11 countries or territories exhibited rates above the global average, with Taiwan (province of China), mainland China, and Malaysia ranking highest at 18.37 (95% UI: 11.85−27.22), 16.11 (95% UI: 12.29−21.07), and 15.99 (95% UI: 10.28−23.68) per 100,000 population, respectively. According to the APC model estimates, ASPR trends among AYAs increased in 104 Out of 204 countries/territories, decreased in 73, and could not be determined in 33 due to insufficient data. Lesotho exhibited the most pronounced rise in ASPR, with a *net drift* of 7.15%. Despite both being among the most populous nations with high NPC case counts, India and China demonstrated divergent ASPR trends: India showed a decreasing trend (*net drift*: −0.25%, 95% CI: −0.38 to −0.11), while China exhibited a sustained upward trajectory (*net drift*: 1.66%, 95% CI: 1.26 to 2.07). These findings underscore substantial global heterogeneity in NPC prevalence trends among AYAs.

### Temporal trends in the prevalence of NPC in different age groups across AYAs population

**Fig. 2:**
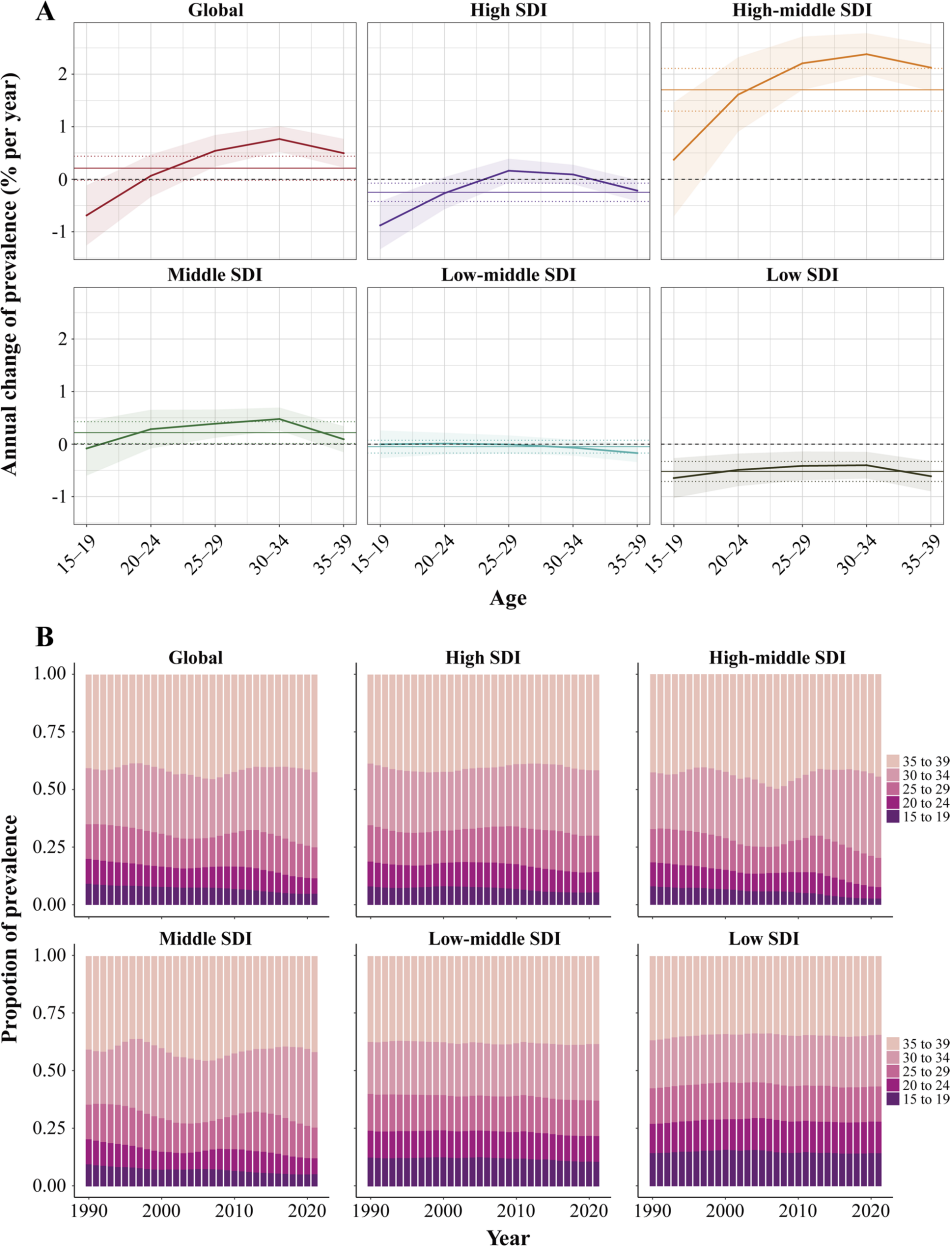
*Net drift*, *local drift* and age distribution of prevalence from 1990 to 2021 for nasopharynx cancer across adolescents and young adults across 5 SDI regions. (A) *Local drift* of prevalence from 1990 to 2021 for nasopharynx cancer across adolescents and young adults for five age groups (15−19, 20−24, 25−29, 30−34, and 35−39 years) from 1990 to 2021. The lines and shaded areas represent the *local drift* and the corresponding 95% confidence intervals, respectively. The solid horizontal line in each plot indicates the *net drift*; the dashed Lines indicate the 95% confidence interval. The labels 15, 20, 25, 30, and 35 represent the age groups 15–19, 20–24, 25–29, 30–34, and 35–39, respectively. (B) Temporal changes in age distribution of nasopharynx cancer across adolescents and young adults from 1990 to 2021

Figure 2A and Table S2 illustrate the annual percentage changes in age-specific prevalence rates—referred to as *local drift*—for NPC among AYAs at the global level and across SDI regions. Globally, the prevalence of NPC among AYAs showed an increasing trend in the 25–29, 30–34, and 35–39 age groups. However, marked regional differences in age-specific trends were observed. In high SDI regions, prevalence declined significantly among those aged 15–19 and 35–39 years. In middle SDI regions, NPC prevalence increased in the 25–29 and 30–34 age groups. In contrast, low-middle SDI regions showed no statistically significant changes in prevalence across any age group. Compared with the relatively stable patterns observed in the above three SDI categories, low SDI regions experienced consistent declines across all age groups, suggesting broad reductions in burden. Conversely, in high-middle SDI regions, all age groups except for the 15–19 age group demonstrated statistically significant increases in NPC prevalence. Further detailing national and regional variability, Table S3 provides *local drift* estimates for each age group across 204 countries and territories.

Figure 2B illustrates the temporal trends in the age distribution of NPC incidence among AYAs. Globally, the burden of NPC has progressively shifted from adolescents to young adults. This trend is also observed in high SDI, high-middle SDI, and middle SDI regions. However, the age distribution in low-middle SDI and low SDI regions has remained relatively stable over the past 32 years, with a persistently higher proportion of cases occurring in the 15–19 and 20–24 age groups.

### Age, period and birth cohort effects on NPC prevalence across AYAs population

**Fig. 3:**
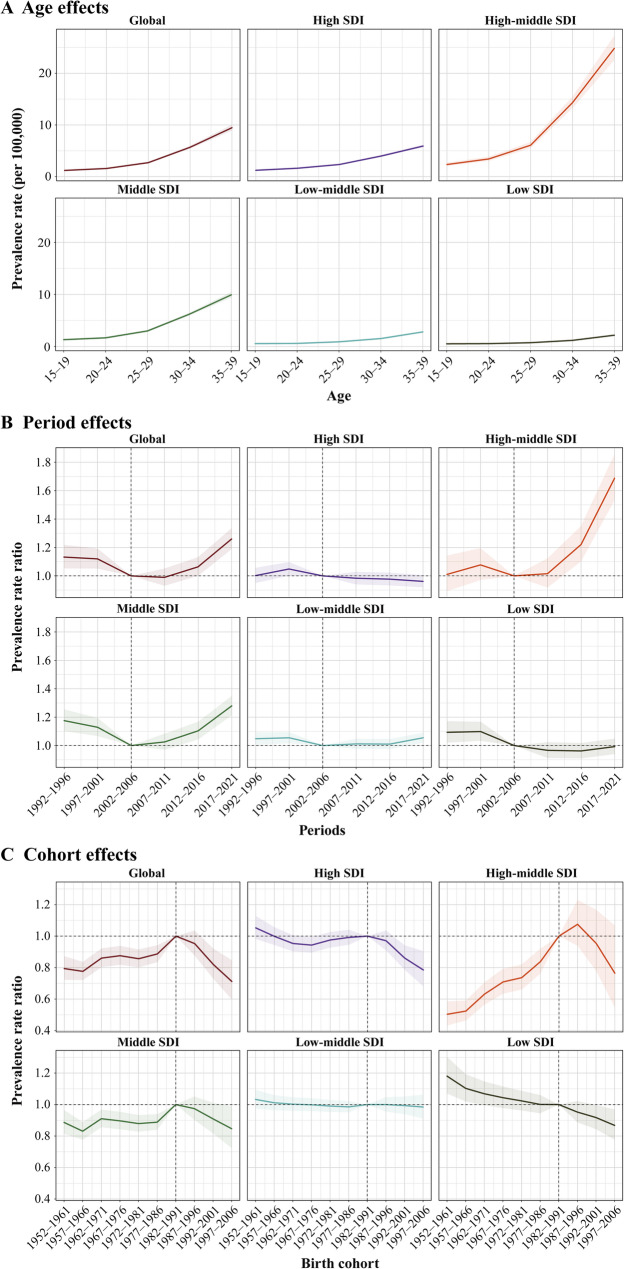
Age, period, and birth cohort effects of nasopharynx cancer across adolescents and young adults across 5 SDI regions. (A) Age effect, expressed by age-specific prevalence. The labels 15, 20, 25, 30, and 35 represent the age groups 15–19, 20–24, 25–29, 30–34, and 35–39, respectively. (B) Period effect, expressed as relative risk by prevalence, with the reference period set to 2002−2006. (C) Birth cohort effect, expressed as relative risk by prevalence, with the reference cohort set to 1982−1991. Lines and shaded areas indicate prevalence or relative risk and their corresponding 95% confidence intervals

Figure 3 and Tables S4–S6 present the age, period, and birth cohort effects on NPC incidence among AYAs. Globally, and across all SDI regions, the age effect exhibited a consistent pattern: the lowest NPC incidence was observed in the 15–19 age group, followed by a steady increase, peaking in the 35–39 age group. This age effect was most pronounced in the high-middle SDI region, followed by the middle SDI region. Regarding period effects, the risk of NPC in AYAs showed an initial decline followed by a subsequent increase globally and in the high-middle and middle SDI regions, most notably in the high-middle SDI region. Period effects remained relatively stable in high, low-middle, and low SDI regions. Using the 2002–2006 period as a reference, the RRs for the 1992–1996 and 2017–2021 periods were as follows: high SDI, 1.00 (95% CI: 0.95 to 1.06) and 0.96 (95% CI: 0.92 to 1.01); high-middle SDI, 1.01 (95% CI: 0.89 to 1.14) and 1.69 (95% CI: 1.53 to 1.86); middle SDI, 1.18 (95% CI: 1.10 to 1.25) and 1.28 (95% CI: 1.21 to 1.35); low-middle SDI, 1.05 (95% CI: 1.00 to 1.09) and 1.05 (95% CI: 1.02 to 1.09); and low SDI, 1.09 (95% CI: 1.02 to 1.17) and 0.99 (95% CI: 0.94 to 1.05). For birth cohort effects, a pattern of increasing followed by decreasing risk was observed globally and in the high-middle and middle SDI regions. Improvements in incidence were noted in the high and low SDI regions, while the birth cohort effect remained relatively stable in the low-middle SDI region. Using the 1982–1991 birth cohort as a reference, the RR for the 1997–2006 birth cohort ranged from 0.78 (95% CI: 0.68 to 0.90) in the high SDI region to 0.87 (95% CI: 0.78 to 0.97) in the low SDI region. No significant improvements in birth cohort effects were observed in the high-middle SDI region [RR, 0.76 (95% CI: 0.55 to 1.07)] or the low-middle SDI region [RR, 0.98 (95% CI: 0.91 to 1.06)]. These findings highlight substantial variations in age, period, and birth cohort effects across different SDI regions, with the highest burden of NPC among AYAs observed in the high-middle SDI region, reflecting the complex and diverse nature of NPC epidemiological trends.

**Fig. 4:**
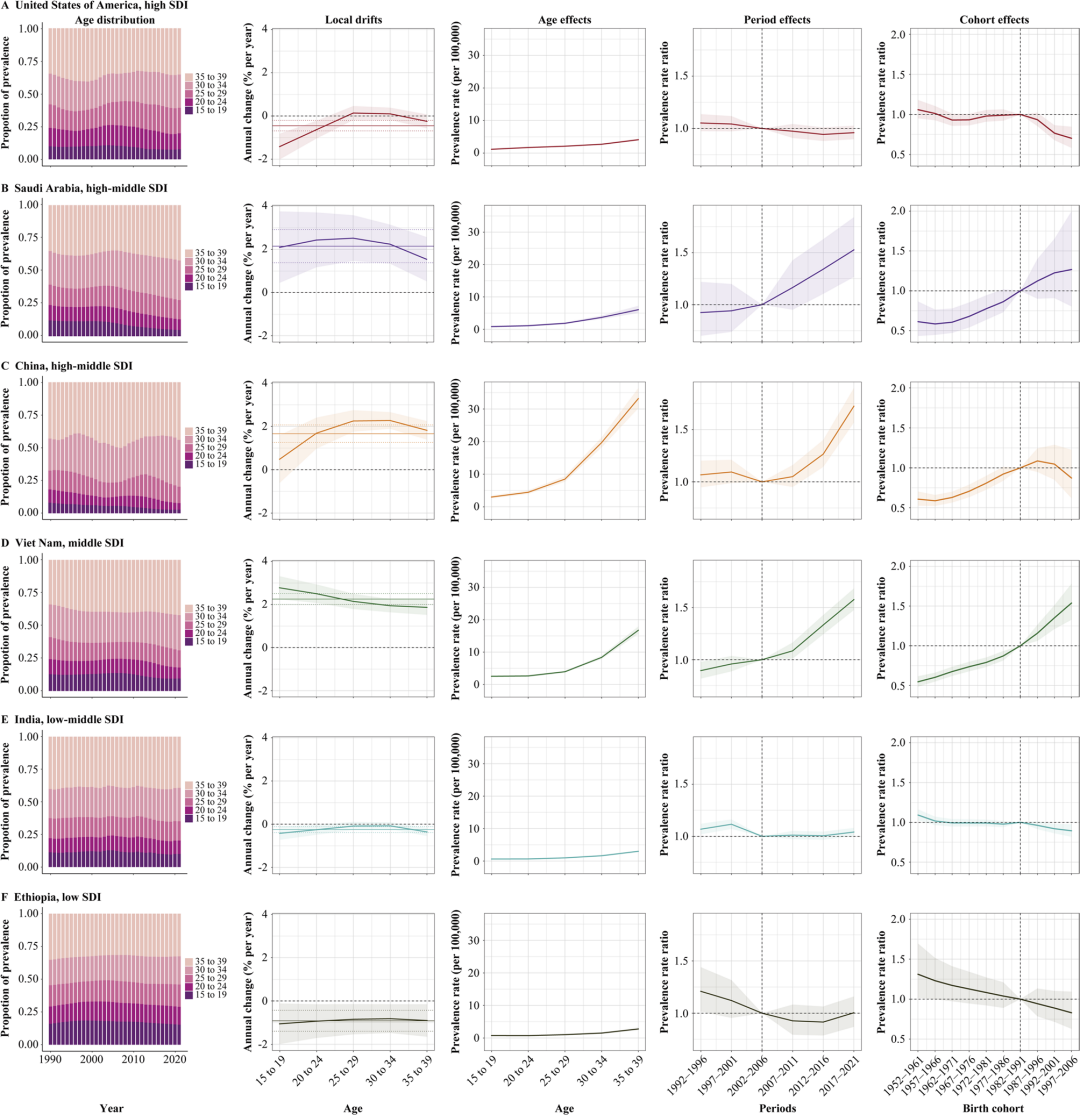
Age, period and birth cohort effects of nasopharynx cancer across adolescents and young adults in exemplary countries. Age distribution of prevalence demonstrates the temporal changes in relative proportion of prevalence from 1990 to 2021 across five age groups (15−19, 20−24, 25−29, 30−34, and 35−39 years). *Net drift* denotes the annual percentage change (% per year) in overall prevalence among the five age groups from 1990 to 2021. *Local drift* denotes the annual percentage change of age-specific prevalence (% per year) from 1990 to 2021 for five age groups. Age effect, expressed as age-specific prevalence. Period effect, expressed as relative risk of prevalence, with the reference period set to 2002−2006. Birth cohort effect, expressed as prevalence relative risk, with the reference cohort set to 1982−1991. The labels 15, 20, 25, 30, and 35 represent the age groups 15–19, 20–24, 25–29, 30–34, and 35–39, respectively. Lines and shaded areas indicate prevalence or relative risk and their corresponding 95% confidence intervals

Tables S7–S9 detail the age, period, and birth cohort effects on NPC prevalence among AYAs for individual countries. To further illustrate the global disparities in NPC prevalence, Fig. 4 showcases six representative countries spanning different SDI levels. Among high SDI countries, the United States exhibits a favorable trend, with a *net drift* of −0.45% (95% CI: −0.69 to −0.21). While the age effect peaks in the 35–39 age group, it remains below the global average; both period and birth cohort effects demonstrate declining RRs. Saudi Arabia and China, both classified as high-middle SDI, experience worsening NPC burdens, with *net drifts* of 2.15% (95% CI: 1.39 to 2.92) and 1.66% (95% CI: 1.26 to 2.07), respectively. Incidence rates are increasing across all age groups in both countries except for the 15–19 age group in China. China displays more pronounced age and period effects compared to Saudi Arabia, while birth cohort effects, although improving in both countries, show no statistically significant difference. Viet Nam, a middle SDI nation, faces a more severe NPC burden than the overall middle SDI region. All age groups in Viet Nam experience significant increases in incidence, with worsening trends observed in recent years for age, period, and birth cohort RRs. India, classified as low-middle SDI, has the second highest number of NPC cases among AYAs after China, yet displays a more optimistic trend, with a *net drift* of −0.25% (95% CI: −0.38 to −0.11) and declining period and birth cohort RRs over time. Ethiopia, a low SDI country, shows continued improvement in its NPC burden, with a *net drift* of − 0.92% (95% CI: −1.40 to −0.43), decreasing incidence across all age groups, and consistently improving period and birth cohort RRs. These case studies underscore the substantial heterogeneity in NPC incidence trends among AYAs across countries with varying SDI levels.

## Discussion

The global AYA population, currently exceeding 2.9 billion, constitutes the world’s largest demographic group [26]. AYAs diagnosed with cancer face numerous unique psychosocial challenges and often experience low clinical trial enrollment, hindering advancements in treatment efficacy [27, 28]. The United Nations Sustainable Development Goal 3 (SDG3) emphasizes “ensuring healthy lives and promoting well-being for all at all ages” [29]. However, the fragmented understanding of NPC poses a barrier to achieving SDG3. This study represents the first comprehensive age-period-cohort analysis of the NPC burden in AYAs, aligning with global calls for data-driven cancer control strategies.

Our findings reveal a striking regional polarization in NPC incidence trends among AYAs globally. While high SDI and low SDI regions demonstrate improvements in NPC burden, the high-middle SDI region has emerged as a hotspot for increasing incidence, with its proportion of cases rising from 34.07% in 1990 to 45.38% in 2021. The ASPR in this region has climbed by 1.70% annually (95% CI: 1.30 to 2.11), with mainland China, Taiwan (province of China), Vietnam, and Malaysia identified as high-burden countries/regions. Furthermore, within the high-middle SDI region, the NPC burden is shifting from adolescents to young adults, accompanied by a Surge in period effect risk. In high SDI regions, early intervention efforts appear to have yielded measurable benefits, with the RR for individuals born between 1997 and 2006 declining to 0.78 (95% CI: 0.68 to 0.90). Conversely, no statistically significant improvement was observed in the same cohort within high-middle SDI regions [RR, 0.76 (95% CI: 0.55 to 1.07)], suggesting a lag in risk factor control within this region.

Our findings share common ground with existing literature while also revealing important distinctions. Previous studies have indicated a decline in NPC incidence rates across the general population, yet a continued rise in prevalence [17]. However, analyses focusing on AYAs have found a doubling of incidence and tripling of prevalence [30], highlighting the complex and nuanced epidemiological characteristics of NPC across different populations. Consistent with previous research [31, 32], this study observed significant geographical disparities in NPC prevalence, with Southeast Asia and China remaining high-incidence regions. This aligns with the known distribution of risk factors, including EBV infection, genetic susceptibility, environmental exposures, and specific dietary habits [3–5]. Building upon trend studies, this study utilizes APC modeling to dissect the complex influence of temporal dimensions—age, period, and birth cohort—on the NPC burden in AYAs at global, regional, and national levels.

The age effect reveals an increasing risk of NPC with advancing age. This trend, consistent across all SDI regions and particularly pronounced in the high-middle SDI region, suggests a cumulative exposure to pathogenic factors as AYAs grow older. These factors may include persistent EBV infection, tobacco and alcohol use, and air pollution [31, 33]. China, as a significant component of high-middle SDI regions, experiences a high prevalence of NPC in provinces from eastern to southeastern China, particularly in Guangdong and Guangxi provinces [1]. Genetic susceptibility represents an important factor in NPC etiology. Certain ancestral lineages, particularly Southeast Asian, Chinese (especially Cantonese), and Inuit populations, are associated with higher NPC risk. This genetic predisposition persists even when individuals migrate to low-risk regions, although the risk may decrease across generations [34, 35]. Early dietary exposures prevalent in Chinese coastal regions, such as consumption of highly salted fish and meat, constitute a recognized environmental risk factor for nasopharyngeal carcinoma, as cooking produces high levels of nitrites that are highly carcinogenic and EBV-activating, promoting nasopharyngeal carcinoma development [36]. In the Taiwan Province of China, the combined effects of alcohol consumption and smoking, coupled with betel nut chewing, further exacerbate the NPC burden [37]. Furthermore, the age effect also reflects the influence of immune system development and functional changes on NPC susceptibility, encompassing the incomplete maturation of the immune system during adolescence and the diminished immunosurveillance associated with immunosenescence in later years [38, 39].

The period effect reflects the immediate impact of societal and environmental factors on NPC risk within AYAs during specific time periods. Globally, and in the high-middle and middle SDI regions, the period effect for NPC exhibits an initial decline followed by a subsequent rise. From the 1990s to the early 2000s, improvements in basic healthcare infrastructure and widespread public health education contributed to an overall decrease in NPC risk. However, a reversal in this trend is observed after 2010, particularly in China, Vietnam, and Saudi Arabia, potentially linked to accelerated industrialization, increased air pollution, and rising tobacco use among young people [40–42]. Studies have shown that air pollution and PM2.5 exposure play significant roles in NPC pathogenesis, especially in the context of rapid urbanization [43]. Moreover, the increasing prevalence of e-cigarette use among AYAs raises concerns, as aldehyde compounds in e-cigarettes can activate the EBV lytic switch gene BZLF1, potentially increasing the risk of viral reactivation [44]. In response, the US FDA has implemented measures to restrict e-cigarette use among youth [45, 46]. In some regions, the failure of NPC prevention and control resources to adapt to changing behaviors and lifestyles among adolescents has contributed to a resurgence in risk [47]. This underscores the need for future prevention strategies to address the comprehensive health risks associated with evolving societal and environmental landscapes.

The birth cohort effect reflects differences in risk factor exposure among individuals born in different periods, which in turn influences NPC burden [25]. In high SDI regions, the reduced RR of NPC prevalence observed among the 1997–2006 birth cohort is likely due to improved infection control during infancy and early childhood, better dietary hygiene, and enhanced EBV prevention strategies. In contrast, this declining trend is not observed in the high-middle SDI region. In some high-middle SDI countries, such as Saudi Arabia, a persistently high prevalence of EBV infection remains a major contributor to NPC burden [48]. In China, genetic factors, particularly the enrichment of certain HLA gene loci in East Asian populations, further increase susceptibility to NPC [49–51]. The situation is particularly severe in Southeast Asian countries, where children born after 1990 have experienced increased exposure to processed meats and higher ambient PM2.5 concentrations. The combined influence of genetic predisposition and detrimental environmental exposures has resulted in a substantial increase in NPC burden among recent birth cohorts in these regions [52–54].

Given the persistent increase and significant heterogeneity in the NPC burden among AYAs, prevention and control strategies should prioritize early screening, health education, environmental interventions, and life-course health management. In high-burden countries/regions, efforts should focus on strengthening early screening and diagnosis of NPC, along with surveillance, management, and vaccination for EBV-related diseases [55, 56]. Simultaneously, interventions targeting risk factors such as adolescent dietary patterns, tobacco use, and air pollution should be intensified, complemented by enhanced health education and early risk identification in schools and communities. Furthermore, in countries/regions where birth cohort risk has not shown improvement, preventive strategies should be shifted towards childhood and adolescence, encompassing nutritional improvements, lifestyle interventions, and vaccination, to establish a more comprehensive life-course prevention framework [3].

This study has several limitations. First, estimates derived from the GBD database are subject to uncertainties related to data completeness, heterogeneity in cancer registry quality, and reliance on modeled estimates where primary data are sparse. While the GBD modeling process is robust, the availability of NPC-specific information is limited. Second, variations in healthcare systems across different countries and regions can influence disease identification and diagnosis. More developed healthcare settings may have better capacity for identifying and diagnosing NPC, potentially leading to higher case counts, whereas less developed settings may experience underdiagnosis and underestimation of the true burden. Third, inherent lags in data collection mean that our data may not fully reflect the most recent health trends and patterns. This lag could affect the timeliness of our response to the impact of NPC and the development of effective policies. Therefore, caution is warranted when interpreting these findings at the national level to avoid overinterpretation or misrepresentation of the data.

## Conclusions

Although the global number of NPC cases among AYAs has increased, the overall ASPR remains largely stable. In contrast, a marked rise in ASPR within high-middle SDI regions—driven by worsening period effects and stagnant cohort improvements—suggests suboptimal control of modifiable risk factors in these settings. These findings underscore the urgent need for targeted public health strategies, including early screening, EBV vaccination, and risk-reduction initiatives tailored to AYAs in high-burden regions. Strengthening surveillance systems and integrating youth-focused cancer prevention into national programs are essential to curb the rising burden and promote health equity across populations.

## Supplementary information

Supplementary Material 1. The prevalence number and age-standardized prevalence rate in 2021, as well as* net drift* of prevalence from 1990 to 2021 for nasopharynx cancer in adolescents and young adults across countries.

Supplementary Material 2. The *local drift *of prevalence from 1990 to 2021 for nasopharynx cancer in adolescents and young adults for five age groups across SDI quintiles.

Supplementary Material 3. The *local drift *of prevalence from 1990 to 2021 for nasopharynx cancer in adolescents and young adults for five age groups across countries.

Supplementary Material 4. Age effects on nasopharynx cancer prevalence in adolescents and young adults across SDI quintiles.

Supplementary Material 5. Period effects on nasopharynx cancer prevalence in adolescents and young adults across SDI quintiles.

Supplementary Material 6. Cohort effects on nasopharynx cancer prevalence in adolescents and young adults across SDI quintiles.

Supplementary Material 7. Age effects on nasopharynx cancer prevalence in adolescents and young adults across countries.

Supplementary Material 8. Period effects on nasopharynx cancer prevalence in adolescents and young adults across countries.

Supplementary Material 9. Cohort effects on nasopharynx cancer prevalence in adolescents and young adults across countries.

## Data Availability

Publicly available datasets were analyzed in this study. GBD study 2021 data resources were available online from the Global Health Data Exchange query tool (http://ghdx.healthdata.org/gbd-results-tool).
