# Supplementary material for: Global, regional, and national temporal trends in prevalence for nasopharynx cancer across adolescents and young adults, 1990–2021: an age-period-cohort analysis based on the global burden of disease study 2021
Source: BMC Oral Health. 2025 Sep 26;25:1435. doi: 10.1186/s12903-025-06750-4 (PMC12465747; doi:10.1186/s12903-025-06750-4)
Supplement: Supplementary file 1 — Supplementary Material 1. The prevalence number and age-standardized prevalence rate in 2021, as well as net drift of prevalence from 1990 to 2021 for nasopharynx cancer in adolescents and young adults across countries. [file 12903_2025_6750_MOESM1_ESM.docx]

**Supplementary Table 1** The prevalence number and age-standardized prevalence rate in 2021, as well as net drift of prevalence from 1990 to 2021 for nasopharynx cancer in adolescents and young adults across countries

| **Location** | **Number** | **ASPR (per 100,000)** | ***Net Drift* (%/year)** |
| --- | --- | --- | --- |
| Afghanistan | 61.77 (27.34, 103.07) | 0.59 (0.26, 1.00) | -0.88 (-2.48 to 0.76) |
| Albania | 9.17 (5.64, 14.54) | 0.95 (0.59, 1.51) | 2.6 (-0.68 to 5.99) |
| Algeria | 910.77 (563.71, 1411.76) | 5.01 (3.09, 7.78) | 0.66 (0.23 to 1.09) |
| American Samoa | 0.29 (0.16, 0.49) | 1.75 (0.96, 2.99) | NA (NA to NA) |
| Andorra | 0.28 (0.14, 0.52) | 1.00 (0.49, 1.86) | NA (NA to NA) |
| Angola | 32.61 (18.82, 53.14) | 0.30 (0.17, 0.49) | 0.16 (-2.17 to 2.55) |
| Antigua and Barbuda | 0.14 (0.11, 0.19) | 0.42 (0.33, 0.55) | NA (NA to NA) |
| Argentina | 65.72 (47.02, 90.47) | 0.37 (0.27, 0.51) | 0.07 (-1.16 to 1.31) |
| Armenia | 5.19 (3.94, 6.59) | 0.47 (0.35, 0.59) | 0.96 (-3.19 to 5.3) |
| Australia | 339.72 (234.42, 474.39) | 3.56 (2.45, 4.97) | -2.33 (-2.97 to -1.69) |
| Austria | 35.36 (23.65, 50.84) | 1.18 (0.79, 1.7) | -0.63 (-2.27 to 1.04) |
| Azerbaijan | 11.15 (6.1, 19.17) | 0.26 (0.14, 0.44) | 0.12 (-2.72 to 3.05) |
| Bahamas | 1.53 (1.1, 2.04) | 0.99 (0.71, 1.31) | NA (NA to NA) |
| Bahrain | 8.88 (4.99, 15.17) | 1.17 (0.66, 2.01) | 1.4 (-4.76 to 7.96) |
| Bangladesh | 1025.65 (499.4, 1903.72) | 1.52 (0.74, 2.81) | 0.27 (-0.1 to 0.64) |
| Barbados | 0.91 (0.64, 1.26) | 0.9 (0.64, 1.25) | NA (NA to NA) |
| Belarus | 17.1 (11.23, 24.8) | 0.5 (0.33, 0.74) | -0.56 (-3.34 to 2.3) |
| Belgium | 62.4 (41.13, 92.02) | 1.68 (1.1, 2.47) | -0.55 (-1.88 to 0.8) |
| Belize | 0.71 (0.56, 0.9) | 0.39 (0.31, 0.49) | NA (NA to NA) |
| Benin | 7.96 (4.1, 14.25) | 0.17 (0.09, 0.31) | -0.81 (-6.78 to 5.54) |
| Bermuda | 0.27 (0.16, 0.41) | 1.45 (0.89, 2.24) | NA (NA to NA) |
| Bhutan | 4.57 (2.29, 8.64) | 1.31 (0.66, 2.47) | -0.57 (-8.54 to 8.09) |
| Bolivia (Plurinational State of) | 9.4 (5.06, 15.96) | 0.19 (0.1, 0.33) | -1.49 (-5.19 to 2.35) |
| Bosnia and Herzegovina | 3 (2.01, 4.16) | 0.28 (0.19, 0.39) | 2.94 (-5.83 to 12.53) |
| Botswana | 4.85 (2.18, 9.12) | 0.44 (0.2, 0.83) | -0.01 (-5.03 to 5.27) |
| Brazil | 448.15 (402.86, 505.19) | 0.51 (0.46, 0.58) | 1.76 (1.21 to 2.32) |
| Brunei Darussalam | 7.62 (4.94, 11.2) | 3.43 (2.21, 5.06) | -0.46 (-6.13 to 5.54) |
| Bulgaria | 29.59 (19.89, 42.86) | 1.39 (0.93, 2.03) | 2.01 (-0.03 to 4.09) |
| Burkina Faso | 14.44 (7.93, 23.53) | 0.19 (0.1, 0.31) | 0.57 (-3.3 to 4.6) |
| Burundi | 68.03 (38.12, 116.93) | 1.38 (0.78, 2.37) | -0.92 (-2.39 to 0.57) |
| Cabo Verde | 1.14 (0.61, 2.03) | 0.44 (0.24, 0.78) | NA (NA to NA) |
| Cambodia | 196.22 (115.94, 322.23) | 2.7 (1.6, 4.43) | 0.74 (-0.27 to 1.75) |
| Cameroon | 27.64 (15.13, 46.28) | 0.24 (0.13, 0.4) | 0.28 (-2.62 to 3.26) |
| Canada | 295.55 (200.92, 420.73) | 2.33 (1.58, 3.32) | -0.6 (-1.24 to 0.04) |
| Central African Republic | 5.35 (3.04, 8.68) | 0.27 (0.15, 0.44) | -1.04 (-5.71 to 3.86) |
| Chad | 9.7 (5.42, 16.55) | 0.18 (0.1, 0.31) | 1.41 (-3.45 to 6.51) |
| Chile | 21.32 (14.43, 30.4) | 0.29 (0.2, 0.42) | 1.15 (-1.35 to 3.73) |
| China | 87945.23 (67089.69, 114858.65) | 16.11 (12.29, 21.07) | 1.66 (1.26 to 2.07) |
| Colombia | 89.02 (60.48, 130.4) | 0.44 (0.3, 0.65) | -0.43 (-1.5 to 0.65) |
| Comoros | 4.26 (2.29, 7.49) | 1.44 (0.77, 2.53) | 3.49 (-4.1 to 11.69) |
| Congo | 7.33 (4.17, 12.19) | 0.34 (0.2, 0.58) | -0.25 (-5.32 to 5.08) |
| Cook Islands | 0.02 (0.01, 0.04) | 0.42 (0.21, 0.76) | NA (NA to NA) |
| Costa Rica | 19.11 (13.47, 26.83) | 0.98 (0.69, 1.38) | -0.2 (-2.71 to 2.36) |
| Cote d'Ivoire | 37.96 (20.01, 66.42) | 0.35 (0.19, 0.62) | 1.00 (-1.13 to 3.17) |
| Croatia | 8.85 (6.05, 12.29) | 0.66 (0.45, 0.92) | 1.00 (-2.48 to 4.61) |
| Cuba | 31.71 (21.44, 44.97) | 0.85 (0.58, 1.21) | 0.64 (-1.08 to 2.4) |
| Cyprus | 4.15 (2.4, 6.88) | 0.72 (0.41, 1.21) | 3.69 (-5.3 to 13.54) |
| Czechia | 45.44 (28.54, 69.04) | 1.4 (0.88, 2.12) | 1.91 (0.31 to 3.54) |
| Democratic People's Republic of Korea | 494.95 (283.37, 842.55) | 4.61 (2.64, 7.85) | 0.34 (-0.27 to 0.96) |
| Democratic Republic of the Congo | 80.94 (46.18, 132.43) | 0.25 (0.14, 0.41) | 0.09 (-1.35 to 1.55) |
| Denmark | 14.37 (9.48, 20.72) | 0.78 (0.51, 1.12) | -0.44 (-3.16 to 2.35) |
| Djibouti | 6.84 (3.09, 13.41) | 1.26 (0.57, 2.47) | 1.72 (-5.07 to 8.99) |
| Dominica | 0.12 (0.07, 0.19) | 0.47 (0.28, 0.73) | NA (NA to NA) |
| Dominican Republic | 24.12 (14.52, 37.54) | 0.53 (0.32, 0.83) | 0.99 (-1.16 to 3.19) |
| Ecuador | 13.02 (8.54, 18.69) | 0.18 (0.12, 0.26) | 0.44 (-2.6 to 3.57) |
| Egypt | 45.85 (28.56, 68.13) | 0.11 (0.07, 0.16) | -0.47 (-2.18 to 1.28) |
| El Salvador | 11.02 (7.79, 15.51) | 0.44 (0.31, 0.62) | 2.7 (-0.87 to 6.41) |
| Equatorial Guinea | 2.08 (0.99, 3.98) | 0.33 (0.16, 0.64) | -3.05 (-14.59 to 10.04) |
| Eritrea | 40.85 (23.19, 68.69) | 1.57 (0.89, 2.65) | 0.33 (-1.74 to 2.45) |
| Estonia | 2.2 (1.4, 3.26) | 0.53 (0.34, 0.79) | -2.6 (-10.79 to 6.35) |
| Eswatini | 3.03 (1.56, 4.97) | 0.62 (0.32, 1.02) | 2.24 (-8.74 to 14.55) |
| Ethiopia | 602.07 (389.44, 969.93) | 1.46 (0.95, 2.35) | -0.92 (-1.4 to -0.43) |
| Fiji | 1.02 (0.56, 1.73) | 0.28 (0.15, 0.48) | NA (NA to NA) |
| Finland | 11.22 (7.37, 16.5) | 0.64 (0.42, 0.94) | 0.54 (-2.54 to 3.72) |
| France | 649.06 (432.27, 933.1) | 3.16 (2.1, 4.54) | 0.33 (-0.09 to 0.74) |
| Gabon | 2.34 (1.22, 4.09) | 0.34 (0.18, 0.59) | 0.86 (-10.19 to 13.28) |
| Gambia | 1.91 (1.11, 3.09) | 0.21 (0.12, 0.33) | NA (NA to NA) |
| Georgia | 8.99 (6.16, 12.89) | 0.76 (0.52, 1.08) | 0.34 (-2.7 to 3.48) |
| Germany | 228.18 (153.58, 330.58) | 0.83 (0.56, 1.21) | -1.78 (-2.42 to -1.13) |
| Ghana | 2.83 (1.46, 5.24) | 0.02 (0.01, 0.04) | -6.84 (-12.38 to -0.95) |
| Greece | 64.23 (48.32, 85.73) | 2.11 (1.59, 2.81) | -0.93 (-2.09 to 0.24) |
| Greenland | 1.37 (0.77, 2.14) | 6.23 (3.49, 9.72) | -2 (-12.2 to 9.38) |
| Grenada | 0.28 (0.19, 0.4) | 0.7 (0.48, 1) | NA (NA to NA) |
| Guam | 1.17 (0.8, 1.67) | 2.14 (1.47, 3.07) | 0.32 (-10.52 to 12.46) |
| Guatemala | 17.35 (13.66, 21.6) | 0.27 (0.21, 0.34) | -0.42 (-3.01 to 2.24) |
| Guinea | 14.14 (8.24, 23.14) | 0.3 (0.17, 0.48) | 0.45 (-2.84 to 3.85) |
| Guinea-Bissau | 2.06 (1.19, 3.38) | 0.27 (0.15, 0.44) | 0.46 (-11.13 to 13.57) |
| Guyana | 0.88 (0.54, 1.34) | 0.29 (0.18, 0.45) | NA (NA to NA) |
| Haiti | 26.64 (13.06, 43.81) | 0.49 (0.24, 0.81) | 0.43 (-1.94 to 2.87) |
| Honduras | 6.77 (3.32, 12.27) | 0.16 (0.08, 0.3) | -2.4 (-6.3 to 1.65) |
| Hungary | 30.99 (21.18, 43.59) | 1.04 (0.71, 1.47) | 0.69 (-1.29 to 2.7) |
| Iceland | 2.25 (1.46, 3.31) | 1.78 (1.16, 2.63) | 1.36 (-9.22 to 13.17) |
| India | 8074.5 (6695.08, 9705.21) | 1.34 (1.12, 1.62) | -0.25 (-0.38 to -0.11) |
| Indonesia | 2340.74 (1523.46, 3499.45) | 2.01 (1.31, 3) | -0.09 (-0.34 to 0.16) |
| Iran (Islamic Republic of) | 160.28 (126.69, 204.47) | 0.43 (0.34, 0.55) | 1.28 (0.3 to 2.26) |
| Iraq | 106.1 (63.8, 172.59) | 0.64 (0.39, 1.04) | -0.46 (-1.63 to 0.73) |
| Ireland | 21.95 (15.15, 30.95) | 1.32 (0.91, 1.86) | 1.08 (-1.28 to 3.5) |
| Israel | 33.5 (22.65, 49.05) | 1.01 (0.68, 1.47) | -0.16 (-2.02 to 1.72) |
| Italy | 309.11 (237.24, 404.88) | 1.83 (1.4, 2.4) | -1.06 (-1.58 to -0.54) |
| Jamaica | 6.98 (4.42, 10.6) | 0.58 (0.37, 0.88) | 0.77 (-3.41 to 5.14) |
| Japan | 312.91 (249.4, 392.09) | 0.92 (0.74, 1.16) | 1.29 (0.73 to 1.86) |
| Jordan | 76.11 (43.69, 128.06) | 1.47 (0.85, 2.47) | 0.25 (-1.4 to 1.93) |
| Kazakhstan | 59.82 (45.69, 78.17) | 0.82 (0.63, 1.08) | 1.18 (-0.16 to 2.54) |
| Kenya | 419.88 (287.88, 602.93) | 2.13 (1.47, 3.06) | 0.72 (0.07 to 1.37) |
| Kiribati | 0.24 (0.13, 0.42) | 0.52 (0.27, 0.91) | NA (NA to NA) |
| Kuwait | 24.73 (15.74, 36.02) | 1.03 (0.66, 1.51) | -1.4 (-4.02 to 1.3) |
| Kyrgyzstan | 24.41 (16.14, 35.34) | 0.89 (0.58, 1.28) | 2.44 (-0.29 to 5.24) |
| Lao People's Democratic Republic | 62.98 (37.71, 101.52) | 2.01 (1.21, 3.24) | -0.23 (-1.85 to 1.42) |
| Latvia | 2.4 (1.57, 3.5) | 0.4 (0.27, 0.58) | -6.12 (-12.97 to 1.26) |
| Lebanon | 26.71 (16.21, 42.71) | 1.07 (0.65, 1.72) | 2 (-0.93 to 5.02) |
| Lesotho | 4.32 (2.44, 7.03) | 0.55 (0.31, 0.9) | 7.15 (-1.77 to 16.87) |
| Liberia | 4.69 (2.48, 8.54) | 0.23 (0.12, 0.41) | 3.9 (-6.92 to 15.98) |
| Libya | 185.41 (110.91, 284.41) | 5.84 (3.49, 8.96) | 1.87 (0.89 to 2.86) |
| Lithuania | 3.19 (2.29, 4.48) | 0.37 (0.26, 0.52) | -2.88 (-8.41 to 2.99) |
| Luxembourg | 3.07 (2.22, 4.23) | 1.3 (0.94, 1.79) | -2.82 (-11.57 to 6.79) |
| Madagascar | 134.88 (78.32, 216.18) | 1.26 (0.73, 2.03) | -0.19 (-1.25 to 0.89) |
| Malawi | 25.67 (14.73, 42.49) | 0.36 (0.21, 0.59) | 0.08 (-2.35 to 2.56) |
| Malaysia | 2286.61 (1471.48, 3385.64) | 15.99 (10.28, 23.68) | 0.96 (0.62 to 1.3) |
| Maldives | 2.02 (1.27, 3.11) | 0.65 (0.4, 1) | 0.45 (-10.91 to 13.25) |
| Mali | 11.9 (6.96, 19.61) | 0.15 (0.09, 0.25) | -0.26 (-3.84 to 3.46) |
| Malta | 5.97 (3.8, 8.98) | 4.07 (2.6, 6.12) | -0.3 (-5.38 to 5.05) |
| Marshall Islands | 0.21 (0.11, 0.38) | 0.91 (0.46, 1.64) | NA (NA to NA) |
| Mauritania | 2.7 (1.26, 5.35) | 0.18 (0.08, 0.35) | 4.29 (-6.85 to 16.76) |
| Mauritius | 10.4 (8.15, 13.12) | 2.22 (1.74, 2.8) | 2.45 (-1.78 to 6.85) |
| Mexico | 128.25 (113.91, 143.42) | 0.25 (0.22, 0.28) | 1.09 (0.04 to 2.15) |
| Micronesia (Federated States of) | 0.34 (0.17, 0.59) | 0.9 (0.46, 1.56) | NA (NA to NA) |
| Monaco | 0.26 (0.14, 0.44) | 2.66 (1.43, 4.54) | NA (NA to NA) |
| Mongolia | 7.56 (4.62, 11.7) | 0.58 (0.35, 0.89) | 0.98 (-2.87 to 4.99) |
| Montenegro | 0.63 (0.41, 0.96) | 0.29 (0.19, 0.45) | NA (NA to NA) |
| Morocco | 202.16 (115.08, 365) | 1.36 (0.77, 2.46) | -0.12 (-0.91 to 0.68) |
| Mozambique | 5.87 (3.26, 10) | 0.06 (0.03, 0.1) | 4.25 (-4.25 to 13.52) |
| Myanmar | 421.73 (258.95, 649.06) | 1.92 (1.18, 2.96) | -0.98 (-1.51 to -0.44) |
| Namibia | 6.46 (3.42, 10.95) | 0.65 (0.34, 1.1) | -1.49 (-5.38 to 2.56) |
| Nauru | 0.05 (0.02, 0.09) | 1.1 (0.48, 2.07) | NA (NA to NA) |
| Nepal | 169.48 (98.3, 286.11) | 1.36 (0.79, 2.28) | 0.17 (-0.74 to 1.09) |
| Netherlands | 123.49 (85.27, 173.92) | 2.26 (1.56, 3.19) | -0.46 (-1.37 to 0.45) |
| New Zealand | 40.49 (29.77, 53.09) | 2.11 (1.55, 2.77) | -2.39 (-4.33 to -0.4) |
| Nicaragua | 7.55 (4.84, 11.53) | 0.27 (0.17, 0.41) | -0.58 (-4.44 to 3.43) |
| Niger | 10.34 (4.74, 21.08) | 0.14 (0.06, 0.29) | 1.28 (-3.48 to 6.28) |
| Nigeria | 756.85 (430.16, 1171.4) | 0.96 (0.55, 1.47) | 0.4 (-0.08 to 0.88) |
| Niue | 0.01 (0, 0.01) | 1.3 (0.69, 2.28) | NA (NA to NA) |
| North Macedonia | 3.9 (2.63, 5.81) | 0.47 (0.32, 0.71) | 1.47 (-5.9 to 9.4) |
| Northern Mariana Islands | 0.31 (0.17, 0.53) | 1.81 (0.99, 3.05) | NA (NA to NA) |
| Norway | 11.7 (8.18, 16.01) | 0.66 (0.46, 0.9) | -0.68 (-3.23 to 1.94) |
| Oman | 18.23 (9.72, 31.26) | 0.69 (0.37, 1.18) | 0.69 (-2.71 to 4.2) |
| Pakistan | 1701.58 (1202.44, 2383.33) | 1.82 (1.29, 2.54) | 0.31 (-0.01 to 0.63) |
| Palau | 0.01 (0, 0.01) | 0.12 (0.06, 0.22) | NA (NA to NA) |
| Palestine | 9.46 (6.05, 14.41) | 0.46 (0.29, 0.7) | -1.69 (-5.46 to 2.24) |
| Panama | 10.08 (7.47, 13.33) | 0.62 (0.46, 0.82) | 0.52 (-3.3 to 4.5) |
| Papua New Guinea | 22.69 (10.93, 42.9) | 0.56 (0.27, 1.07) | -0.39 (-5.24 to 4.71) |
| Paraguay | 8.7 (5.42, 13.33) | 0.29 (0.18, 0.44) | 0.91 (-3.07 to 5.06) |
| Peru | 30.33 (18.8, 46.3) | 0.2 (0.13, 0.31) | 1.83 (-0.44 to 4.16) |
| Philippines | 1287.61 (1034.05, 1579.27) | 2.86 (2.3, 3.5) | -0.23 (-0.6 to 0.14) |
| Poland | 104.69 (85.55, 129.58) | 0.75 (0.61, 0.93) | 2.02 (0.78 to 3.28) |
| Portugal | 50.89 (33.65, 74.26) | 1.63 (1.07, 2.38) | -0.36 (-1.71 to 1) |
| Puerto Rico | 12.53 (8.07, 18.63) | 1.19 (0.77, 1.77) | 1.19 (-1.5 to 3.96) |
| Qatar | 16.64 (9.11, 28.61) | 0.9 (0.49, 1.57) | 2.16 (-4.36 to 9.12) |
| Republic of Korea | 174.47 (109.84, 261.2) | 0.99 (0.62, 1.48) | 1.86 (0.94 to 2.78) |
| Republic of Moldova | 9.65 (7.42, 12.7) | 0.71 (0.55, 0.94) | -0.58 (-3.66 to 2.59) |
| Romania | 71.94 (47.22, 104.62) | 1.23 (0.81, 1.79) | 2.98 (1.63 to 4.35) |
| Russian Federation | 221 (197.56, 249.97) | 0.41 (0.37, 0.46) | 0.42 (-0.32 to 1.17) |
| Rwanda | 82.43 (44.71, 146.15) | 1.55 (0.84, 2.74) | -1.75 (-2.99 to -0.49) |
| Saint Kitts and Nevis | 0.13 (0.08, 0.19) | 0.55 (0.37, 0.81) | NA (NA to NA) |
| Saint Lucia | 0.72 (0.55, 0.93) | 1.06 (0.81, 1.37) | NA (NA to NA) |
| Saint Vincent and the Grenadines | 0.33 (0.26, 0.43) | 0.81 (0.63, 1.04) | NA (NA to NA) |
| Samoa | 2.5 (1.32, 4.35) | 3.41 (1.81, 5.93) | -0.47 (-11.23 to 11.59) |
| San Marino | 0.25 (0.13, 0.42) | 2.75 (1.42, 4.64) | NA (NA to NA) |
| Sao Tome and Principe | 0.03 (0.02, 0.06) | 0.04 (0.02, 0.07) | NA (NA to NA) |
| Saudi Arabia | 584.59 (329.89, 951.15) | 2.75 (1.55, 4.49) | 2.15 (1.39 to 2.92) |
| Senegal | 11.91 (5.88, 22.13) | 0.21 (0.1, 0.38) | 2 (-2.5 to 6.72) |
| Serbia | 17.59 (9.78, 29.61) | 0.54 (0.3, 0.91) | 0.89 (-1.76 to 3.62) |
| Seychelles | 1.35 (0.8, 2.17) | 3.17 (1.89, 5.11) | 0.82 (-10.57 to 13.66) |
| Sierra Leone | 6.35 (3.32, 10.95) | 0.19 (0.1, 0.33) | 1.99 (-6.79 to 11.59) |
| Singapore | 172.43 (115.62, 247.69) | 8.34 (5.6, 11.97) | -0.28 (-1.12 to 0.56) |
| Slovakia | 12.51 (7.78, 19.31) | 0.64 (0.4, 1) | 1.52 (-1.79 to 4.95) |
| Slovenia | 3.38 (2.08, 5.09) | 0.54 (0.33, 0.82) | -3.12 (-9.25 to 3.42) |
| Solomon Islands | 1.87 (1.01, 3.19) | 0.76 (0.41, 1.29) | -0.98 (-12.17 to 11.63) |
| Somalia | 91.74 (49.74, 158.56) | 1.27 (0.68, 2.2) | -0.22 (-1.61 to 1.18) |
| South Africa | 118.4 (95.87, 149.24) | 0.47 (0.38, 0.59) | -1.01 (-2.03 to 0.02) |
| South Sudan | 39.86 (21.36, 71.53) | 1.22 (0.65, 2.19) | 0.67 (-1.14 to 2.52) |
| Spain | 304.2 (203.86, 450.79) | 2.27 (1.52, 3.34) | -1.73 (-2.27 to -1.18) |
| Sri Lanka | 199.98 (110.83, 322.8) | 2.45 (1.36, 3.97) | 0.53 (-0.32 to 1.39) |
| Sudan | 74.07 (37.92, 122.06) | 0.43 (0.22, 0.71) | -0.72 (-2.08 to 0.66) |
| Suriname | 1.84 (1.19, 2.77) | 0.86 (0.55, 1.28) | 2 (-9.96 to 15.55) |
| Sweden | 45.46 (32.66, 61.29) | 1.31 (0.94, 1.77) | -1.1 (-2.77 to 0.6) |
| Switzerland | 18.44 (12.36, 26.79) | 0.62 (0.41, 0.9) | -3.73 (-5.66 to -1.76) |
| Syrian Arab Republic | 12.44 (8.14, 18.35) | 0.26 (0.17, 0.39) | 0.54 (-2.1 to 3.24) |
| Taiwan (Province of China) | 1593.96 (1025.37, 2366.34) | 18.37 (11.85, 27.22) | -1.48 (-1.78 to -1.18) |
| Tajikistan | 26.75 (16.98, 39.76) | 0.64 (0.41, 0.95) | -1.14 (-3.13 to 0.9) |
| Thailand | 1792.06 (1132.43, 2717.41) | 7.87 (4.96, 11.96) | 1.41 (1.05 to 1.76) |
| Timor-Leste | 7.39 (4.28, 12.31) | 1.53 (0.89, 2.55) | 2.22 (-3.64 to 8.44) |
| Togo | 6.63 (3.65, 11.24) | 0.21 (0.12, 0.36) | -1.07 (-7.18 to 5.45) |
| Tokelau | 0.01 (0, 0.01) | 1.32 (0.71, 2.24) | NA (NA to NA) |
| Tonga | 0.24 (0.12, 0.44) | 0.69 (0.35, 1.29) | NA (NA to NA) |
| Trinidad and Tobago | 3.36 (2.38, 4.57) | 0.64 (0.45, 0.87) | 5.92 (-5 to 18.08) |
| Tunisia | 297.96 (175.09, 472.34) | 6.35 (3.71, 10.1) | 1.5 (0.76 to 2.24) |
| Turkey | 469.42 (293.96, 727.89) | 1.44 (0.9, 2.24) | 0.67 (0.17 to 1.18) |
| Turkmenistan | 19.58 (14.27, 26.85) | 0.94 (0.69, 1.29) | 1.79 (-0.87 to 4.52) |
| Tuvalu | 0.04 (0.02, 0.07) | 0.84 (0.46, 1.42) | NA (NA to NA) |
| Uganda | 473.78 (279.08, 755.69) | 3.18 (1.87, 5.05) | -0.29 (-0.87 to 0.3) |
| Ukraine | 99.48 (64.24, 145.34) | 0.63 (0.41, 0.92) | 1.33 (0.21 to 2.46) |
| United Arab Emirates | 44.35 (23.53, 72.82) | 1.06 (0.59, 1.73) | 0.48 (-1.79 to 2.81) |
| United Kingdom | 527.62 (493.4, 563.19) | 2.32 (2.17, 2.48) | 0.45 (0 to 0.9) |
| United Republic of Tanzania | 301.2 (164.75, 520.72) | 1.43 (0.78, 2.48) | -0.13 (-0.83 to 0.59) |
| United States of America | 2368.58 (2198.4, 2539.44) | 2.06 (1.91, 2.2) | -0.45 (-0.69 to -0.21) |
| United States Virgin Islands | 0.37 (0.19, 0.63) | 1.57 (0.83, 2.74) | NA (NA to NA) |
| Uruguay | 10.84 (7.48, 15.26) | 0.9 (0.62, 1.26) | 1.78 (-1.66 to 5.35) |
| Uzbekistan | 112.16 (74.53, 160.11) | 0.8 (0.53, 1.14) | 1.02 (-0.09 to 2.14) |
| Vanuatu | 0.74 (0.4, 1.3) | 0.66 (0.36, 1.16) | NA (NA to NA) |
| Venezuela (Bolivarian Republic of) | 52.99 (33.4, 75.99) | 0.55 (0.35, 0.8) | 3.08 (1.47 to 4.72) |
| Viet Nam | 3213.35 (1897.15, 5175.69) | 7.76 (4.57, 12.48) | 2.25 (2 to 2.51) |
| Yemen | 38.12 (18.87, 66.1) | 0.3 (0.15, 0.51) | -0.44 (-2.71 to 1.87) |
| Zambia | 181.29 (62.36, 393.68) | 2.51 (0.86, 5.41) | 1.94 (0.79 to 3.12) |
| Zimbabwe | 52.51 (30.88, 82.12) | 0.89 (0.52, 1.4) | 2.91 (0.93 to 4.92) |
