# Supplementary material for: Global, regional, and national temporal trends in prevalence for nasopharynx cancer across adolescents and young adults, 1990–2021: an age-period-cohort analysis based on the global burden of disease study 2021
Source: BMC Oral Health. 2025 Sep 26;25:1435. doi: 10.1186/s12903-025-06750-4 (PMC12465747; doi:10.1186/s12903-025-06750-4)
Supplement: Supplementary file 2 — Supplementary Material 2. The local drift of prevalence from 1990 to 2021 for nasopharynx cancer in adolescents and young adults for five age groups across SDI quintiles. [file 12903_2025_6750_MOESM2_ESM.docx]

**Supplementary Table 2** The local drift of prevalence from 1990 to 2021 for nasopharynx cancer in adolescents and young adults for five age groups across SDI quintiles

| **Location** | **Age** | ***Local drift* (%/year)** |
| --- | --- | --- |
| Global | 15 to 19 | -0.69 (-1.25 to -0.12) |
| Global | 20 to 24 | 0.07 (-0.33 to 0.47) |
| Global | 25 to 29 | 0.54 (0.24 to 0.84) |
| Global | 30 to 34 | 0.77 (0.52 to 1.01) |
| Global | 35 to 39 | 0.50 (0.23 to 0.77) |
| High SDI | 15 to 19 | -0.88 (-1.33 to -0.42) |
| High SDI | 20 to 24 | -0.26 (-0.57 to 0.04) |
| High SDI | 25 to 29 | 0.16 (-0.06 to 0.39) |
| High SDI | 30 to 34 | 0.09 (-0.09 to 0.27) |
| High SDI | 35 to 39 | -0.22 (-0.42 to -0.01) |
| High-middle SDI | 15 to 19 | 0.37 (-0.70 to 1.46) |
| High-middle SDI | 20 to 24 | 1.61 (0.91 to 2.32) |
| High-middle SDI | 25 to 29 | 2.21 (1.70 to 2.71) |
| High-middle SDI | 30 to 34 | 2.38 (1.98 to 2.78) |
| High-middle SDI | 35 to 39 | 2.12 (1.68 to 2.57) |
| Middle SDI | 15 to 19 | -0.08 (-0.60 to 0.44) |
| Middle SDI | 20 to 24 | 0.28 (-0.08 to 0.65) |
| Middle SDI | 25 to 29 | 0.39 (0.12 to 0.66) |
| Middle SDI | 30 to 34 | 0.48 (0.26 to 0.69) |
| Middle SDI | 35 to 39 | 0.09 (-0.15 to 0.34) |
| Low-middle SDI | 15 to 19 | 0.00 (-0.27 to 0.26) |
| Low-middle SDI | 20 to 24 | 0.01 (-0.19 to 0.21) |
| Low-middle SDI | 25 to 29 | -0.01 (-0.18 to 0.16) |
| Low-middle SDI | 30 to 34 | -0.07 (-0.22 to 0.08) |
| Low-middle SDI | 35 to 39 | -0.17 (-0.34 to 0.00) |
| Low SDI | 15 to 19 | -0.65 (-1.02 to -0.27) |
| Low SDI | 20 to 24 | -0.49 (-0.8 to -0.18) |
| Low SDI | 25 to 29 | -0.41 (-0.69 to -0.14) |
| Low SDI | 30 to 34 | -0.4 (-0.65 to -0.15) |
| Low SDI | 35 to 39 | -0.61 (-0.90 to -0.33) |
