# Supplementary material for: Global, regional, and national temporal trends in prevalence for nasopharynx cancer across adolescents and young adults, 1990–2021: an age-period-cohort analysis based on the global burden of disease study 2021
Source: BMC Oral Health. 2025 Sep 26;25:1435. doi: 10.1186/s12903-025-06750-4 (PMC12465747; doi:10.1186/s12903-025-06750-4)
Supplement: Supplementary file 3 — Supplementary Material 3. The local drift of prevalence from 1990 to 2021 for nasopharynx cancer in adolescents and young adults for five age groups across countries. [file 12903_2025_6750_MOESM3_ESM.docx]

**Supplementary Table 3** The local drift of prevalence from 1990 to 2021 for nasopharynx cancer in adolescents and young adults for five age groups across countries

| **Location** | **Age** | ***Local drift*** (%/year) |
| --- | --- | --- |
| Afghanistan | 15 to 19 | -0.54 (-3.46 to 2.48) |
| Afghanistan | 20 to 24 | -0.67 (-2.99 to 1.69) |
| Afghanistan | 25 to 29 | -0.88 (-2.99 to 1.28) |
| Afghanistan | 30 to 34 | -0.97 (-3.45 to 1.57) |
| Afghanistan | 35 to 39 | -1.36 (-4.4 to 1.79) |
| Albania | 15 to 19 | 1.48 (-5.65 to 9.16) |
| Albania | 20 to 24 | 2.85 (-2.04 to 7.98) |
| Albania | 25 to 29 | 2.87 (-1.61 to 7.55) |
| Albania | 30 to 34 | 2.97 (-1.2 to 7.31) |
| Albania | 35 to 39 | 2.80 (-2.57 to 8.48) |
| Algeria | 15 to 19 | 0.78 (-0.13 to 1.7) |
| Algeria | 20 to 24 | 0.73 (0.02 to 1.43) |
| Algeria | 25 to 29 | 0.58 (-0.01 to 1.17) |
| Algeria | 30 to 34 | 0.59 (0.05 to 1.12) |
| Algeria | 35 to 39 | 0.59 (-0.06 to 1.25) |
| Angola | 15 to 19 | -0.3 (-4.76 to 4.37) |
| Angola | 20 to 24 | -0.07 (-3.68 to 3.68) |
| Angola | 25 to 29 | -0.04 (-3.28 to 3.31) |
| Angola | 30 to 34 | 0.46 (-2.82 to 3.85) |
| Angola | 35 to 39 | 0.62 (-3.03 to 4.42) |
| Argentina | 15 to 19 | 0.52 (-1.94 to 3.04) |
| Argentina | 20 to 24 | 0.54 (-1.37 to 2.49) |
| Argentina | 25 to 29 | 0.2 (-1.57 to 2) |
| Argentina | 30 to 34 | -0.2 (-1.9 to 1.53) |
| Argentina | 35 to 39 | -0.65 (-2.64 to 1.37) |
| Armenia | 15 to 19 | 2.48 (-5.48 to 11.12) |
| Armenia | 20 to 24 | 0.83 (-5.29 to 7.34) |
| Armenia | 25 to 29 | -0.31 (-5.96 to 5.69) |
| Armenia | 30 to 34 | 0.25 (-5.83 to 6.73) |
| Armenia | 35 to 39 | 1.06 (-6.79 to 9.58) |
| Australia | 15 to 19 | -3.71 (-5.44 to -1.94) |
| Australia | 20 to 24 | -2.85 (-3.98 to -1.7) |
| Australia | 25 to 29 | -1.98 (-2.75 to -1.21) |
| Australia | 30 to 34 | -1.59 (-2.2 to -0.99) |
| Australia | 35 to 39 | -1.45 (-2.16 to -0.74) |
| Austria | 15 to 19 | -1.25 (-5.27 to 2.93) |
| Austria | 20 to 24 | -0.6 (-3.34 to 2.22) |
| Austria | 25 to 29 | -0.13 (-2.27 to 2.06) |
| Austria | 30 to 34 | -0.24 (-2.13 to 1.69) |
| Austria | 35 to 39 | -0.7 (-2.89 to 1.55) |
| Azerbaijan | 15 to 19 | 0.32 (-5.29 to 6.25) |
| Azerbaijan | 20 to 24 | -0.22 (-4.53 to 4.27) |
| Azerbaijan | 25 to 29 | -0.23 (-4.04 to 3.73) |
| Azerbaijan | 30 to 34 | 0.31 (-3.76 to 4.55) |
| Azerbaijan | 35 to 39 | 0.25 (-5.11 to 5.92) |
| Bahrain | 15 to 19 | 3.01 (-9.91 to 17.79) |
| Bahrain | 20 to 24 | 1.25 (-8.66 to 12.23) |
| Bahrain | 25 to 29 | 1.21 (-7.59 to 10.86) |
| Bahrain | 30 to 34 | 1.48 (-4.67 to 8.02) |
| Bahrain | 35 to 39 | -0.01 (-7.04 to 7.56) |
| Bangladesh | 15 to 19 | 0.87 (0.12 to 1.62) |
| Bangladesh | 20 to 24 | 0.82 (0.21 to 1.44) |
| Bangladesh | 25 to 29 | 0.41 (-0.12 to 0.93) |
| Bangladesh | 30 to 34 | -0.12 (-0.61 to 0.36) |
| Bangladesh | 35 to 39 | -0.54 (-1.08 to 0) |
| Belarus | 15 to 19 | -0.05 (-7.02 to 7.45) |
| Belarus | 20 to 24 | -0.37 (-5.41 to 4.94) |
| Belarus | 25 to 29 | -0.06 (-3.57 to 3.58) |
| Belarus | 30 to 34 | -0.69 (-3.47 to 2.18) |
| Belarus | 35 to 39 | -1.51 (-4.33 to 1.4) |
| Belgium | 15 to 19 | -1.02 (-4.24 to 2.32) |
| Belgium | 20 to 24 | -0.97 (-3.22 to 1.34) |
| Belgium | 25 to 29 | -0.5 (-2.3 to 1.32) |
| Belgium | 30 to 34 | -0.12 (-1.65 to 1.44) |
| Belgium | 35 to 39 | -0.11 (-1.81 to 1.61) |
| Benin | 15 to 19 | -2.5 (-20.68 to 19.84) |
| Benin | 20 to 24 | -0.72 (-7.99 to 7.11) |
| Benin | 25 to 29 | -0.53 (-7.83 to 7.35) |
| Benin | 30 to 34 | 0.08 (-5.88 to 6.43) |
| Benin | 35 to 39 | -0.24 (-7.47 to 7.55) |
| Bhutan | 15 to 19 | -0.34 (-21.17 to 25.98) |
| Bhutan | 20 to 24 | -0.64 (-15.69 to 17.09) |
| Bhutan | 25 to 29 | 0.7 (-7.12 to 9.18) |
| Bhutan | 30 to 34 | -1.4 (-7.57 to 5.18) |
| Bhutan | 35 to 39 | -0.77 (-8.36 to 7.44) |
| Bolivia (Plurinational State of) | 15 to 19 | -1.74 (-9.24 to 6.38) |
| Bolivia (Plurinational State of) | 20 to 24 | -1.78 (-7.66 to 4.47) |
| Bolivia (Plurinational State of) | 25 to 29 | -1.53 (-6.76 to 4) |
| Bolivia (Plurinational State of) | 30 to 34 | -0.97 (-5.93 to 4.25) |
| Bolivia (Plurinational State of) | 35 to 39 | -1.16 (-6.66 to 4.66) |
| Bosnia and Herzegovina | 15 to 19 | 0.14 (-20.5 to 26.13) |
| Bosnia and Herzegovina | 20 to 24 | 2.68 (-13.28 to 21.58) |
| Bosnia and Herzegovina | 25 to 29 | 7.47 (-2.78 to 18.8) |
| Bosnia and Herzegovina | 30 to 34 | 3.75 (-3.54 to 11.58) |
| Bosnia and Herzegovina | 35 to 39 | 1.6 (-6.45 to 10.35) |
| Botswana | 15 to 19 | 0.22 (-8.11 to 9.31) |
| Botswana | 20 to 24 | 1.21 (-7.06 to 10.22) |
| Botswana | 25 to 29 | 0.57 (-7.13 to 8.92) |
| Botswana | 30 to 34 | -0.76 (-7.14 to 6.06) |
| Botswana | 35 to 39 | -1.26 (-8.4 to 6.42) |
| Brazil | 15 to 19 | 3.04 (1.98 to 4.11) |
| Brazil | 20 to 24 | 2.57 (1.72 to 3.42) |
| Brazil | 25 to 29 | 1.7 (0.92 to 2.49) |
| Brazil | 30 to 34 | 1.04 (0.28 to 1.8) |
| Brazil | 35 to 39 | 0.47 (-0.43 to 1.38) |
| Brunei Darussalam | 15 to 19 | -1.98 (-20.2 to 20.4) |
| Brunei Darussalam | 20 to 24 | -1.64 (-9.06 to 6.38) |
| Brunei Darussalam | 25 to 29 | -0.68 (-6.52 to 5.53) |
| Brunei Darussalam | 30 to 34 | 0.64 (-4.44 to 6) |
| Brunei Darussalam | 35 to 39 | 1.31 (-4.14 to 7.07) |
| Bulgaria | 15 to 19 | 2.56 (-2 to 7.32) |
| Bulgaria | 20 to 24 | 2.21 (-1.38 to 5.93) |
| Bulgaria | 25 to 29 | 1.92 (-0.91 to 4.82) |
| Bulgaria | 30 to 34 | 1.66 (-0.71 to 4.09) |
| Bulgaria | 35 to 39 | 1.72 (-0.78 to 4.28) |
| Burkina Faso | 15 to 19 | -1.02 (-9.1 to 7.78) |
| Burkina Faso | 20 to 24 | 0.03 (-5.68 to 6.08) |
| Burkina Faso | 25 to 29 | 0.15 (-5.07 to 5.65) |
| Burkina Faso | 30 to 34 | 1.12 (-3.61 to 6.08) |
| Burkina Faso | 35 to 39 | 2.57 (-4.01 to 9.6) |
| Burundi | 15 to 19 | -1.09 (-3.87 to 1.78) |
| Burundi | 20 to 24 | -0.85 (-3.23 to 1.58) |
| Burundi | 25 to 29 | -0.78 (-2.93 to 1.4) |
| Burundi | 30 to 34 | -0.74 (-2.72 to 1.28) |
| Burundi | 35 to 39 | -1.1 (-3.27 to 1.13) |
| Cambodia | 15 to 19 | 0.7 (-1.5 to 2.96) |
| Cambodia | 20 to 24 | 0.57 (-1.13 to 2.3) |
| Cambodia | 25 to 29 | 0.71 (-0.66 to 2.11) |
| Cambodia | 30 to 34 | 0.82 (-0.36 to 2.02) |
| Cambodia | 35 to 39 | 0.85 (-0.51 to 2.23) |
| Cameroon | 15 to 19 | -1.6 (-8.39 to 5.7) |
| Cameroon | 20 to 24 | 0.12 (-4.05 to 4.48) |
| Cameroon | 25 to 29 | 0.57 (-3.42 to 4.73) |
| Cameroon | 30 to 34 | 0.94 (-2.65 to 4.66) |
| Cameroon | 35 to 39 | 1.58 (-3.2 to 6.59) |
| Canada | 15 to 19 | -1.56 (-3.15 to 0.06) |
| Canada | 20 to 24 | -0.5 (-1.58 to 0.6) |
| Canada | 25 to 29 | -0.1 (-0.94 to 0.76) |
| Canada | 30 to 34 | -0.2 (-0.92 to 0.54) |
| Canada | 35 to 39 | -0.49 (-1.3 to 0.34) |
| Central African Republic | 15 to 19 | -1.76 (-10.91 to 8.33) |
| Central African Republic | 20 to 24 | -1.69 (-9.42 to 6.69) |
| Central African Republic | 25 to 29 | -1.24 (-7.56 to 5.51) |
| Central African Republic | 30 to 34 | -0.68 (-6.59 to 5.59) |
| Central African Republic | 35 to 39 | 0.1 (-7.21 to 8) |
| Chad | 15 to 19 | 4.97 (-6.65 to 18.03) |
| Chad | 20 to 24 | -0.14 (-7.26 to 7.54) |
| Chad | 25 to 29 | 0.51 (-6.42 to 7.95) |
| Chad | 30 to 34 | 0.6 (-5.31 to 6.89) |
| Chad | 35 to 39 | 1.18 (-5.91 to 8.8) |
| Chile | 15 to 19 | 0.28 (-5.1 to 5.96) |
| Chile | 20 to 24 | 1.22 (-2.77 to 5.38) |
| Chile | 25 to 29 | 1.73 (-1.7 to 5.28) |
| Chile | 30 to 34 | 1.57 (-1.72 to 4.97) |
| Chile | 35 to 39 | 1.1 (-2.81 to 5.16) |
| China | 15 to 19 | 0.48 (-0.61 to 1.59) |
| China | 20 to 24 | 1.69 (0.97 to 2.41) |
| China | 25 to 29 | 2.25 (1.76 to 2.75) |
| China | 30 to 34 | 2.27 (1.89 to 2.66) |
| China | 35 to 39 | 1.82 (1.39 to 2.25) |
| Colombia | 15 to 19 | -0.51 (-2.74 to 1.77) |
| Colombia | 20 to 24 | -0.4 (-2.07 to 1.29) |
| Colombia | 25 to 29 | -0.31 (-1.78 to 1.18) |
| Colombia | 30 to 34 | -0.32 (-1.79 to 1.18) |
| Colombia | 35 to 39 | -0.58 (-2.42 to 1.29) |
| Comoros | 15 to 19 | 12.5 (-3.56 to 31.23) |
| Comoros | 20 to 24 | 7.04 (-6.86 to 23.02) |
| Comoros | 25 to 29 | 1.92 (-9.25 to 14.47) |
| Comoros | 30 to 34 | -0.9 (-8.07 to 6.82) |
| Comoros | 35 to 39 | -2.94 (-10.98 to 5.83) |
| Congo | 15 to 19 | 0.18 (-10.58 to 12.24) |
| Congo | 20 to 24 | 0.24 (-8.14 to 9.38) |
| Congo | 25 to 29 | -0.68 (-7.04 to 6.11) |
| Congo | 30 to 34 | -0.65 (-6.19 to 5.22) |
| Congo | 35 to 39 | -0.35 (-7.41 to 7.24) |
| Costa Rica | 15 to 19 | -0.03 (-5.37 to 5.62) |
| Costa Rica | 20 to 24 | 0.47 (-3.52 to 4.63) |
| Costa Rica | 25 to 29 | 0.26 (-3.18 to 3.82) |
| Costa Rica | 30 to 34 | -0.55 (-3.81 to 2.83) |
| Costa Rica | 35 to 39 | -1.08 (-4.98 to 2.98) |
| Coted'Ivoire | 15 to 19 | 0.83 (-3.38 to 5.23) |
| Coted'Ivoire | 20 to 24 | 1.19 (-2.01 to 4.5) |
| Coted'Ivoire | 25 to 29 | 1.28 (-1.83 to 4.49) |
| Coted'Ivoire | 30 to 34 | 1.02 (-1.76 to 3.88) |
| Coted'Ivoire | 35 to 39 | 0.75 (-2.76 to 4.4) |
| Croatia | 15 to 19 | 2.23 (-5.46 to 10.54) |
| Croatia | 20 to 24 | 0.85 (-4.97 to 7.04) |
| Croatia | 25 to 29 | 0.67 (-4.26 to 5.86) |
| Croatia | 30 to 34 | 0.99 (-3.11 to 5.27) |
| Croatia | 35 to 39 | 0.18 (-3.82 to 4.35) |
| Cuba | 15 to 19 | 0.59 (-3.28 to 4.61) |
| Cuba | 20 to 24 | 0.36 (-2.47 to 3.27) |
| Cuba | 25 to 29 | 0.37 (-1.9 to 2.69) |
| Cuba | 30 to 34 | 0.57 (-1.55 to 2.73) |
| Cuba | 35 to 39 | 1.25 (-1.66 to 4.24) |
| Cyprus | 15 to 19 | 4.29 (-16.63 to 30.47) |
| Cyprus | 20 to 24 | 5.44 (-8.02 to 20.86) |
| Cyprus | 25 to 29 | 4.26 (-6.89 to 16.75) |
| Cyprus | 30 to 34 | 4.23 (-6.57 to 16.28) |
| Cyprus | 35 to 39 | 0.32 (-7.29 to 8.54) |
| Czechia | 15 to 19 | 2.24 (-1.46 to 6.08) |
| Czechia | 20 to 24 | 2.62 (-0.04 to 5.36) |
| Czechia | 25 to 29 | 2.21 (0.05 to 4.42) |
| Czechia | 30 to 34 | 1.71 (-0.18 to 3.65) |
| Czechia | 35 to 39 | 0.91 (-1.1 to 2.97) |
| Democratic People's Republic of Korea | 15 to 19 | -0.15 (-1.72 to 1.46) |
| Democratic People's Republic of Korea | 20 to 24 | 0.3 (-0.78 to 1.39) |
| Democratic People's Republic of Korea | 25 to 29 | 0.5 (-0.27 to 1.27) |
| Democratic People's Republic of Korea | 30 to 34 | 0.55 (-0.06 to 1.16) |
| Democratic People's Republic of Korea | 35 to 39 | 0.56 (-0.15 to 1.27) |
| Democratic Republic of the Congo | 15 to 19 | -0.02 (-2.87 to 2.91) |
| Democratic Republic of the Congo | 20 to 24 | 0.08 (-2.22 to 2.43) |
| Democratic Republic of the Congo | 25 to 29 | 0.06 (-1.95 to 2.12) |
| Democratic Republic of the Congo | 30 to 34 | 0.09 (-1.82 to 2.03) |
| Democratic Republic of the Congo | 35 to 39 | 0.22 (-2 to 2.5) |
| Denmark | 15 to 19 | -1.17 (-8.06 to 6.24) |
| Denmark | 20 to 24 | -0.2 (-4.74 to 4.56) |
| Denmark | 25 to 29 | 0.28 (-3.14 to 3.83) |
| Denmark | 30 to 34 | -0.34 (-3.28 to 2.69) |
| Denmark | 35 to 39 | -0.56 (-3.83 to 2.81) |
| Djibouti | 15 to 19 | 3.42 (-9.22 to 17.83) |
| Djibouti | 20 to 24 | 3.97 (-7.56 to 16.93) |
| Djibouti | 25 to 29 | 2.87 (-6.65 to 13.37) |
| Djibouti | 30 to 34 | 0.3 (-6.09 to 7.13) |
| Djibouti | 35 to 39 | -1.52 (-8.56 to 6.06) |
| Dominican Republic | 15 to 19 | 1.91 (-2.48 to 6.49) |
| Dominican Republic | 20 to 24 | 1.41 (-2.02 to 4.96) |
| Dominican Republic | 25 to 29 | 0.98 (-1.96 to 4) |
| Dominican Republic | 30 to 34 | 0.68 (-2.16 to 3.59) |
| Dominican Republic | 35 to 39 | 0.07 (-3.59 to 3.87) |
| Ecuador | 15 to 19 | 0.62 (-5.23 to 6.83) |
| Ecuador | 20 to 24 | 0.95 (-3.68 to 5.79) |
| Ecuador | 25 to 29 | 0.7 (-3.58 to 5.18) |
| Ecuador | 30 to 34 | 0.32 (-3.6 to 4.41) |
| Ecuador | 35 to 39 | -0.22 (-5.23 to 5.06) |
| Egypt | 15 to 19 | 0.06 (-3.39 to 3.64) |
| Egypt | 20 to 24 | -0.22 (-3.06 to 2.7) |
| Egypt | 25 to 29 | -0.25 (-2.68 to 2.23) |
| Egypt | 30 to 34 | -0.57 (-2.78 to 1.69) |
| Egypt | 35 to 39 | -1.29 (-3.86 to 1.36) |
| El Salvador | 15 to 19 | 3.37 (-3.22 to 10.4) |
| El Salvador | 20 to 24 | 2.76 (-2.5 to 8.3) |
| El Salvador | 25 to 29 | 2.73 (-2.19 to 7.9) |
| El Salvador | 30 to 34 | 1.71 (-3.24 to 6.91) |
| El Salvador | 35 to 39 | 2.74 (-4.07 to 10.04) |
| Equatorial Guinea | 15 to 19 | -6.88 (-27.59 to 19.76) |
| Equatorial Guinea | 20 to 24 | -4.68 (-21.78 to 16.16) |
| Equatorial Guinea | 25 to 29 | -3.11 (-19.04 to 15.95) |
| Equatorial Guinea | 30 to 34 | -1.39 (-17.74 to 18.21) |
| Equatorial Guinea | 35 to 39 | 1.6 (-18.33 to 26.41) |
| Eritrea | 15 to 19 | 0.19 (-3.73 to 4.28) |
| Eritrea | 20 to 24 | 0.28 (-3 to 3.68) |
| Eritrea | 25 to 29 | 0.46 (-2.49 to 3.5) |
| Eritrea | 30 to 34 | 0.47 (-2.27 to 3.29) |
| Eritrea | 35 to 39 | 0.25 (-2.71 to 3.31) |
| Estonia | 15 to 19 | -0.58 (-21.46 to 25.85) |
| Estonia | 20 to 24 | -3.66 (-18.14 to 13.4) |
| Estonia | 25 to 29 | -3.94 (-14.71 to 8.18) |
| Estonia | 30 to 34 | -0.86 (-6.77 to 5.42) |
| Estonia | 35 to 39 | -3.21 (-9.65 to 3.69) |
| Eswatini | 15 to 19 | -2.1 (-22.8 to 24.15) |
| Eswatini | 20 to 24 | 1.53 (-15.36 to 21.78) |
| Eswatini | 25 to 29 | 5.19 (-8.86 to 21.39) |
| Eswatini | 30 to 34 | 2.74 (-8.68 to 15.59) |
| Eswatini | 35 to 39 | 5.24 (-13.03 to 27.36) |
| Ethiopia | 15 to 19 | -1.06 (-1.99 to -0.12) |
| Ethiopia | 20 to 24 | -0.93 (-1.71 to -0.15) |
| Ethiopia | 25 to 29 | -0.85 (-1.54 to -0.15) |
| Ethiopia | 30 to 34 | -0.82 (-1.48 to -0.15) |
| Ethiopia | 35 to 39 | -0.9 (-1.65 to -0.15) |
| Finland | 15 to 19 | 1.6 (-5.81 to 9.6) |
| Finland | 20 to 24 | 1.15 (-4.01 to 6.57) |
| Finland | 25 to 29 | 0.41 (-3.72 to 4.72) |
| Finland | 30 to 34 | 0.04 (-3.52 to 3.73) |
| Finland | 35 to 39 | -0.44 (-4.36 to 3.65) |
| France | 15 to 19 | -0.23 (-1.23 to 0.78) |
| France | 20 to 24 | 0.47 (-0.22 to 1.18) |
| France | 25 to 29 | 0.93 (0.37 to 1.49) |
| France | 30 to 34 | 0.75 (0.26 to 1.23) |
| France | 35 to 39 | -0.06 (-0.59 to 0.47) |
| Gabon | 15 to 19 | -2.26 (-23.67 to 25.17) |
| Gabon | 20 to 24 | -0.58 (-17.9 to 20.39) |
| Gabon | 25 to 29 | 0.46 (-14.39 to 17.89) |
| Gabon | 30 to 34 | 1.65 (-10.02 to 14.82) |
| Gabon | 35 to 39 | 4.76 (-13.47 to 26.83) |
| Georgia | 15 to 19 | -0.2 (-7.14 to 7.25) |
| Georgia | 20 to 24 | -0.71 (-5.78 to 4.64) |
| Georgia | 25 to 29 | -0.39 (-4.41 to 3.79) |
| Georgia | 30 to 34 | 0.95 (-2.96 to 5.03) |
| Georgia | 35 to 39 | 1.81 (-2.98 to 6.83) |
| Germany | 15 to 19 | -2.35 (-3.98 to -0.68) |
| Germany | 20 to 24 | -1.9 (-3.01 to -0.78) |
| Germany | 25 to 29 | -1.41 (-2.26 to -0.55) |
| Germany | 30 to 34 | -1.24 (-1.93 to -0.53) |
| Germany | 35 to 39 | -1.85 (-2.6 to -1.1) |
| Ghana | 15 to 19 | -5.5 (-23.21 to 16.28) |
| Ghana | 20 to 24 | -6.72 (-13.62 to 0.72) |
| Ghana | 25 to 29 | -6.89 (-13.87 to 0.65) |
| Ghana | 30 to 34 | -7.31 (-13.26 to -0.95) |
| Ghana | 35 to 39 | -7.9 (-14.08 to -1.26) |
| Greece | 15 to 19 | -1.47 (-4.42 to 1.58) |
| Greece | 20 to 24 | -1.17 (-3.11 to 0.8) |
| Greece | 25 to 29 | -1.1 (-2.63 to 0.46) |
| Greece | 30 to 34 | -0.63 (-1.94 to 0.7) |
| Greece | 35 to 39 | -0.26 (-1.73 to 1.24) |
| Greenland | 15 to 19 | -1.28 (-22.87 to 26.36) |
| Greenland | 20 to 24 | -1.69 (-18.9 to 19.17) |
| Greenland | 25 to 29 | -2.62 (-16.94 to 14.16) |
| Greenland | 30 to 34 | -2.68 (-13.71 to 9.75) |
| Greenland | 35 to 39 | -1.89 (-9.33 to 6.16) |
| Guam | 15 to 19 | 0.25 (-21.71 to 28.38) |
| Guam | 20 to 24 | 0.53 (-17.14 to 21.97) |
| Guam | 25 to 29 | -0.2 (-15.48 to 17.82) |
| Guam | 30 to 34 | 0.44 (-12.71 to 15.56) |
| Guam | 35 to 39 | 0.61 (-8.18 to 10.24) |
| Guatemala | 15 to 19 | -0.13 (-5.03 to 5.01) |
| Guatemala | 20 to 24 | -0.09 (-4.17 to 4.16) |
| Guatemala | 25 to 29 | -0.59 (-4.17 to 3.13) |
| Guatemala | 30 to 34 | -0.37 (-3.98 to 3.37) |
| Guatemala | 35 to 39 | -0.91 (-5.18 to 3.57) |
| Guinea | 15 to 19 | 1.14 (-5.23 to 7.94) |
| Guinea | 20 to 24 | 0.92 (-4.08 to 6.19) |
| Guinea | 25 to 29 | 0.22 (-4.37 to 5.03) |
| Guinea | 30 to 34 | 0.19 (-4.55 to 5.17) |
| Guinea | 35 to 39 | -0.25 (-5.63 to 5.43) |
| Guinea-Bissau | 15 to 19 | -4.87 (-26 to 22.3) |
| Guinea-Bissau | 20 to 24 | -1.23 (-18.78 to 20.13) |
| Guinea-Bissau | 25 to 29 | 1.17 (-14.72 to 20.04) |
| Guinea-Bissau | 30 to 34 | 4.02 (-11.5 to 22.25) |
| Guinea-Bissau | 35 to 39 | 4.24 (-16.2 to 29.65) |
| Haiti | 15 to 19 | 0.87 (-3.66 to 5.62) |
| Haiti | 20 to 24 | 0.33 (-3.32 to 4.12) |
| Haiti | 25 to 29 | 0.22 (-3.06 to 3.61) |
| Haiti | 30 to 34 | 0.35 (-2.97 to 3.79) |
| Haiti | 35 to 39 | 0.28 (-3.84 to 4.57) |
| Honduras | 15 to 19 | -2.51 (-10 to 5.6) |
| Honduras | 20 to 24 | -3.09 (-8.92 to 3.11) |
| Honduras | 25 to 29 | -3.44 (-8.73 to 2.16) |
| Honduras | 30 to 34 | -2.48 (-8.08 to 3.45) |
| Honduras | 35 to 39 | -0.72 (-7.79 to 6.9) |
| Hungary | 15 to 19 | 1.22 (-3.74 to 6.42) |
| Hungary | 20 to 24 | 1.82 (-1.51 to 5.27) |
| Hungary | 25 to 29 | 1.26 (-1.37 to 3.96) |
| Hungary | 30 to 34 | 0.24 (-1.95 to 2.49) |
| Hungary | 35 to 39 | -0.87 (-2.98 to 1.28) |
| Iceland | 15 to 19 | 0.7 (-21.17 to 28.65) |
| Iceland | 20 to 24 | 1.55 (-16.07 to 22.86) |
| Iceland | 25 to 29 | 1.69 (-13.34 to 19.32) |
| Iceland | 30 to 34 | 2.85 (-8.89 to 16.1) |
| Iceland | 35 to 39 | 0.49 (-8.22 to 10.02) |
| India | 15 to 19 | -0.42 (-0.72 to -0.12) |
| India | 20 to 24 | -0.25 (-0.48 to -0.02) |
| India | 25 to 29 | -0.08 (-0.27 to 0.11) |
| India | 30 to 34 | -0.07 (-0.24 to 0.09) |
| India | 35 to 39 | -0.36 (-0.54 to -0.18) |
| Indonesia | 15 to 19 | 0 (-0.58 to 0.59) |
| Indonesia | 20 to 24 | -0.1 (-0.54 to 0.33) |
| Indonesia | 25 to 29 | -0.12 (-0.46 to 0.23) |
| Indonesia | 30 to 34 | -0.09 (-0.38 to 0.19) |
| Indonesia | 35 to 39 | -0.16 (-0.48 to 0.16) |
| Iran (Islamic Republic of) | 15 to 19 | 1.56 (-0.39 to 3.55) |
| Iran (Islamic Republic of) | 20 to 24 | 1.56 (0 to 3.14) |
| Iran (Islamic Republic of) | 25 to 29 | 1.29 (-0.05 to 2.65) |
| Iran (Islamic Republic of) | 30 to 34 | 1.14 (-0.15 to 2.46) |
| Iran (Islamic Republic of) | 35 to 39 | 0.84 (-0.82 to 2.52) |
| Iraq | 15 to 19 | 0.32 (-2.07 to 2.76) |
| Iraq | 20 to 24 | -0.47 (-2.37 to 1.46) |
| Iraq | 25 to 29 | -0.61 (-2.25 to 1.05) |
| Iraq | 30 to 34 | -0.71 (-2.22 to 0.82) |
| Iraq | 35 to 39 | -0.85 (-2.62 to 0.95) |
| Ireland | 15 to 19 | -0.27 (-5.45 to 5.21) |
| Ireland | 20 to 24 | 0.57 (-3.39 to 4.7) |
| Ireland | 25 to 29 | 2.03 (-1.18 to 5.34) |
| Ireland | 30 to 34 | 1.93 (-0.86 to 4.8) |
| Ireland | 35 to 39 | 1.45 (-1.85 to 4.86) |
| Israel | 15 to 19 | -0.42 (-4.32 to 3.64) |
| Israel | 20 to 24 | -0.26 (-3.16 to 2.73) |
| Israel | 25 to 29 | -0.01 (-2.57 to 2.62) |
| Israel | 30 to 34 | 0.03 (-2.4 to 2.53) |
| Israel | 35 to 39 | -0.15 (-2.97 to 2.74) |
| Italy | 15 to 19 | -1.35 (-2.66 to -0.03) |
| Italy | 20 to 24 | -1.09 (-1.98 to -0.2) |
| Italy | 25 to 29 | -0.93 (-1.62 to -0.23) |
| Italy | 30 to 34 | -0.98 (-1.56 to -0.4) |
| Italy | 35 to 39 | -0.91 (-1.57 to -0.24) |
| Jamaica | 15 to 19 | 0.26 (-8.45 to 9.79) |
| Jamaica | 20 to 24 | -0.17 (-6.68 to 6.8) |
| Jamaica | 25 to 29 | 0.96 (-4.73 to 7) |
| Jamaica | 30 to 34 | 1.25 (-4 to 6.79) |
| Jamaica | 35 to 39 | 1.74 (-5.38 to 9.39) |
| Japan | 15 to 19 | 1.16 (-0.17 to 2.51) |
| Japan | 20 to 24 | 1.27 (0.37 to 2.17) |
| Japan | 25 to 29 | 1.37 (0.62 to 2.13) |
| Japan | 30 to 34 | 1.41 (0.7 to 2.13) |
| Japan | 35 to 39 | 1.28 (0.42 to 2.14) |
| Jordan | 15 to 19 | 0.42 (-2.86 to 3.81) |
| Jordan | 20 to 24 | 0.52 (-2.06 to 3.17) |
| Jordan | 25 to 29 | 0.57 (-1.7 to 2.9) |
| Jordan | 30 to 34 | 0.12 (-2.06 to 2.35) |
| Jordan | 35 to 39 | -0.29 (-3.07 to 2.58) |
| Kazakhstan | 15 to 19 | 1.25 (-1.61 to 4.18) |
| Kazakhstan | 20 to 24 | 0.9 (-1.29 to 3.14) |
| Kazakhstan | 25 to 29 | 0.87 (-0.96 to 2.74) |
| Kazakhstan | 30 to 34 | 1.31 (-0.48 to 3.13) |
| Kazakhstan | 35 to 39 | 1.49 (-0.73 to 3.75) |
| Kenya | 15 to 19 | 0.79 (-0.45 to 2.05) |
| Kenya | 20 to 24 | 0.66 (-0.38 to 1.7) |
| Kenya | 25 to 29 | 0.62 (-0.29 to 1.55) |
| Kenya | 30 to 34 | 0.67 (-0.19 to 1.54) |
| Kenya | 35 to 39 | 0.82 (-0.21 to 1.86) |
| Kuwait | 15 to 19 | -0.36 (-6.35 to 6.02) |
| Kuwait | 20 to 24 | -1.52 (-6.24 to 3.42) |
| Kuwait | 25 to 29 | -1.36 (-4.8 to 2.2) |
| Kuwait | 30 to 34 | -1.75 (-4.59 to 1.18) |
| Kuwait | 35 to 39 | -2.04 (-5.7 to 1.76) |
| Kyrgyzstan | 15 to 19 | 2.51 (-2.9 to 8.21) |
| Kyrgyzstan | 20 to 24 | 2.5 (-1.89 to 7.09) |
| Kyrgyzstan | 25 to 29 | 2.42 (-1.33 to 6.3) |
| Kyrgyzstan | 30 to 34 | 2.78 (-0.87 to 6.56) |
| Kyrgyzstan | 35 to 39 | 2.05 (-2.33 to 6.64) |
| Lao People's Democratic Republic | 15 to 19 | -0.07 (-3.63 to 3.63) |
| Lao People's Democratic Republic | 20 to 24 | 0.09 (-2.65 to 2.9) |
| Lao People's Democratic Republic | 25 to 29 | -0.11 (-2.33 to 2.16) |
| Lao People's Democratic Republic | 30 to 34 | -0.46 (-2.38 to 1.5) |
| Lao People's Democratic Republic | 35 to 39 | -0.59 (-2.72 to 1.58) |
| Latvia | 15 to 19 | -8.36 (-25.06 to 12.06) |
| Latvia | 20 to 24 | -8.12 (-20.64 to 6.37) |
| Latvia | 25 to 29 | -6.44 (-16.51 to 4.83) |
| Latvia | 30 to 34 | -3 (-8.62 to 2.96) |
| Latvia | 35 to 39 | -3.97 (-9.75 to 2.18) |
| Lebanon | 15 to 19 | 3.11 (-3.13 to 9.76) |
| Lebanon | 20 to 24 | 2.77 (-1.95 to 7.72) |
| Lebanon | 25 to 29 | 2.14 (-1.82 to 6.26) |
| Lebanon | 30 to 34 | 1.49 (-2.05 to 5.15) |
| Lebanon | 35 to 39 | 0.45 (-3.67 to 4.74) |
| Lesotho | 15 to 19 | 11.2 (-3.65 to 28.33) |
| Lesotho | 20 to 24 | 11.84 (-1.87 to 27.47) |
| Lesotho | 25 to 29 | 9.79 (-3 to 24.26) |
| Lesotho | 30 to 34 | 5 (-6.06 to 17.36) |
| Lesotho | 35 to 39 | -0.6 (-9.14 to 8.73) |
| Liberia | 15 to 19 | 0.19 (-20.58 to 26.41) |
| Liberia | 20 to 24 | 2.5 (-12.56 to 20.16) |
| Liberia | 25 to 29 | 2.89 (-11.55 to 19.69) |
| Liberia | 30 to 34 | 5.83 (-9.16 to 23.29) |
| Liberia | 35 to 39 | 7.95 (-12.09 to 32.55) |
| Libya | 15 to 19 | 2.37 (0.37 to 4.41) |
| Libya | 20 to 24 | 2.36 (0.76 to 3.98) |
| Libya | 25 to 29 | 2.08 (0.72 to 3.46) |
| Libya | 30 to 34 | 1.6 (0.4 to 2.82) |
| Libya | 35 to 39 | 1.04 (-0.39 to 2.5) |
| Lithuania | 15 to 19 | -4.25 (-20.67 to 15.56) |
| Lithuania | 20 to 24 | -1.04 (-8.93 to 7.54) |
| Lithuania | 25 to 29 | -2.4 (-8.45 to 4.05) |
| Lithuania | 30 to 34 | -2.78 (-7.62 to 2.32) |
| Lithuania | 35 to 39 | -3.65 (-9 to 2.02) |
| Low SDI | 15 to 19 | -0.65 (-1.02 to -0.27) |
| Low SDI | 20 to 24 | -0.49 (-0.8 to -0.18) |
| Low SDI | 25 to 29 | -0.41 (-0.69 to -0.14) |
| Low SDI | 30 to 34 | -0.4 (-0.65 to -0.15) |
| Low SDI | 35 to 39 | -0.61 (-0.9 to -0.33) |
| Luxembourg | 15 to 19 | -2.72 (-23.48 to 23.67) |
| Luxembourg | 20 to 24 | -3.38 (-18.89 to 15.08) |
| Luxembourg | 25 to 29 | -4.5 (-15.84 to 8.38) |
| Luxembourg | 30 to 34 | -2.04 (-8.94 to 5.38) |
| Luxembourg | 35 to 39 | -1.45 (-9.63 to 7.47) |
| Madagascar | 15 to 19 | 0.07 (-1.97 to 2.15) |
| Madagascar | 20 to 24 | -0.02 (-1.72 to 1.71) |
| Madagascar | 25 to 29 | -0.15 (-1.67 to 1.39) |
| Madagascar | 30 to 34 | -0.33 (-1.77 to 1.13) |
| Madagascar | 35 to 39 | -0.5 (-2.17 to 1.2) |
| Malawi | 15 to 19 | -0.25 (-4.84 to 4.57) |
| Malawi | 20 to 24 | -0.03 (-3.86 to 3.96) |
| Malawi | 25 to 29 | 0.11 (-3.32 to 3.66) |
| Malawi | 30 to 34 | 0.28 (-3.12 to 3.79) |
| Malawi | 35 to 39 | 0.19 (-3.56 to 4.09) |
| Malaysia | 15 to 19 | 0.91 (0.15 to 1.68) |
| Malaysia | 20 to 24 | 1.03 (0.45 to 1.61) |
| Malaysia | 25 to 29 | 0.94 (0.48 to 1.4) |
| Malaysia | 30 to 34 | 0.95 (0.59 to 1.32) |
| Malaysia | 35 to 39 | 0.97 (0.56 to 1.37) |
| Maldives | 15 to 19 | -3.29 (-24.77 to 24.34) |
| Maldives | 20 to 24 | -2.16 (-19.53 to 18.95) |
| Maldives | 25 to 29 | -0.18 (-15.82 to 18.36) |
| Maldives | 30 to 34 | 3.09 (-11.88 to 20.6) |
| Maldives | 35 to 39 | 4.2 (-15.39 to 28.34) |
| Mali | 15 to 19 | -0.91 (-7.25 to 5.87) |
| Mali | 20 to 24 | -0.85 (-6.13 to 4.73) |
| Mali | 25 to 29 | -1.31 (-6.31 to 3.97) |
| Mali | 30 to 34 | 0.1 (-5.11 to 5.59) |
| Mali | 35 to 39 | 1.43 (-5.52 to 8.89) |
| Malta | 15 to 19 | -5.05 (-21.15 to 14.32) |
| Malta | 20 to 24 | 0.28 (-5.67 to 6.6) |
| Malta | 25 to 29 | 0.25 (-5.07 to 5.86) |
| Malta | 30 to 34 | 1.37 (-3.66 to 6.66) |
| Malta | 35 to 39 | 2.11 (-3.44 to 7.99) |
| Mauritania | 15 to 19 | -1.2 (-22.22 to 25.52) |
| Mauritania | 20 to 24 | 3.68 (-11.93 to 22.05) |
| Mauritania | 25 to 29 | 2.84 (-13.25 to 21.91) |
| Mauritania | 30 to 34 | 7.31 (-8.15 to 25.38) |
| Mauritania | 35 to 39 | 8.97 (-11.41 to 34.05) |
| Mauritius | 15 to 19 | 3.82 (-6.34 to 15.07) |
| Mauritius | 20 to 24 | 3.06 (-3.93 to 10.57) |
| Mauritius | 25 to 29 | 2.73 (-2.49 to 8.23) |
| Mauritius | 30 to 34 | 1.69 (-2.52 to 6.1) |
| Mauritius | 35 to 39 | 0.96 (-3.26 to 5.36) |
| Mexico | 15 to 19 | 1.18 (-0.96 to 3.38) |
| Mexico | 20 to 24 | 1.38 (-0.27 to 3.05) |
| Mexico | 25 to 29 | 1.25 (-0.21 to 2.74) |
| Mexico | 30 to 34 | 0.97 (-0.45 to 2.41) |
| Mexico | 35 to 39 | 0.7 (-1.02 to 2.44) |
| Mongolia | 15 to 19 | 0.6 (-7.1 to 8.94) |
| Mongolia | 20 to 24 | 0.24 (-5.68 to 6.52) |
| Mongolia | 25 to 29 | 1.04 (-4.14 to 6.5) |
| Mongolia | 30 to 34 | 1.51 (-3.96 to 7.28) |
| Mongolia | 35 to 39 | 1.54 (-5.61 to 9.23) |
| Morocco | 15 to 19 | 0.77 (-0.9 to 2.46) |
| Morocco | 20 to 24 | 0.16 (-1.16 to 1.51) |
| Morocco | 25 to 29 | -0.3 (-1.41 to 0.82) |
| Morocco | 30 to 34 | -0.53 (-1.5 to 0.44) |
| Morocco | 35 to 39 | -0.71 (-1.78 to 0.37) |
| Mozambique | 15 to 19 | 10.16 (-5.42 to 28.31) |
| Mozambique | 20 to 24 | 5.28 (-8.56 to 21.23) |
| Mozambique | 25 to 29 | 1.57 (-10.15 to 14.83) |
| Mozambique | 30 to 34 | 3.01 (-7.87 to 15.18) |
| Mozambique | 35 to 39 | 0.89 (-6.74 to 9.13) |
| Myanmar | 15 to 19 | -0.78 (-2.02 to 0.47) |
| Myanmar | 20 to 24 | -0.96 (-1.88 to -0.03) |
| Myanmar | 25 to 29 | -1.04 (-1.77 to -0.31) |
| Myanmar | 30 to 34 | -1 (-1.62 to -0.37) |
| Myanmar | 35 to 39 | -1.12 (-1.82 to -0.43) |
| Namibia | 15 to 19 | -1.12 (-8.71 to 7.11) |
| Namibia | 20 to 24 | -1.61 (-7.52 to 4.69) |
| Namibia | 25 to 29 | -2.04 (-7.4 to 3.64) |
| Namibia | 30 to 34 | -2.27 (-7.55 to 3.31) |
| Namibia | 35 to 39 | -0.57 (-7.51 to 6.89) |
| Nepal | 15 to 19 | 0.13 (-1.73 to 2.02) |
| Nepal | 20 to 24 | 0.24 (-1.24 to 1.74) |
| Nepal | 25 to 29 | 0.26 (-1.03 to 1.55) |
| Nepal | 30 to 34 | 0.19 (-0.99 to 1.39) |
| Nepal | 35 to 39 | 0.09 (-1.26 to 1.46) |
| Netherlands | 15 to 19 | -0.87 (-3.03 to 1.33) |
| Netherlands | 20 to 24 | -0.6 (-2.18 to 1) |
| Netherlands | 25 to 29 | -0.25 (-1.46 to 0.97) |
| Netherlands | 30 to 34 | -0.21 (-1.25 to 0.83) |
| Netherlands | 35 to 39 | -0.31 (-1.49 to 0.88) |
| New Zealand | 15 to 19 | -4.47 (-10.37 to 1.81) |
| New Zealand | 20 to 24 | -2.58 (-5.73 to 0.67) |
| New Zealand | 25 to 29 | -2.14 (-4.31 to 0.08) |
| New Zealand | 30 to 34 | -1.51 (-3.22 to 0.22) |
| New Zealand | 35 to 39 | -1.07 (-3.18 to 1.09) |
| Nicaragua | 15 to 19 | -1.31 (-8.85 to 6.84) |
| Nicaragua | 20 to 24 | -1.5 (-7.4 to 4.78) |
| Nicaragua | 25 to 29 | -1.44 (-6.68 to 4.11) |
| Nicaragua | 30 to 34 | 0 (-5.33 to 5.64) |
| Nicaragua | 35 to 39 | 1.4 (-5.57 to 8.87) |
| Niger | 15 to 19 | 1.58 (-9.06 to 13.45) |
| Niger | 20 to 24 | 1.04 (-5.84 to 8.42) |
| Niger | 25 to 29 | 0.56 (-6.14 to 7.73) |
| Niger | 30 to 34 | 1.19 (-4.48 to 7.2) |
| Niger | 35 to 39 | 1.84 (-5.27 to 9.49) |
| Nigeria | 15 to 19 | 0.36 (-0.66 to 1.38) |
| Nigeria | 20 to 24 | 0.47 (-0.28 to 1.23) |
| Nigeria | 25 to 29 | 0.45 (-0.22 to 1.13) |
| Nigeria | 30 to 34 | 0.42 (-0.2 to 1.04) |
| Nigeria | 35 to 39 | 0.3 (-0.45 to 1.07) |
| North Macedonia | 15 to 19 | -1.24 (-19.99 to 21.9) |
| North Macedonia | 20 to 24 | 3.45 (-7.57 to 15.78) |
| North Macedonia | 25 to 29 | 3 (-5.98 to 12.83) |
| North Macedonia | 30 to 34 | 1.61 (-5.07 to 8.75) |
| North Macedonia | 35 to 39 | 1.15 (-6.31 to 9.2) |
| Norway | 15 to 19 | -0.15 (-5.63 to 5.64) |
| Norway | 20 to 24 | -0.25 (-4.31 to 3.98) |
| Norway | 25 to 29 | -0.48 (-3.89 to 3.04) |
| Norway | 30 to 34 | -1.17 (-4.83 to 2.62) |
| Norway | 35 to 39 | -1.39 (-6.1 to 3.56) |
| Oman | 15 to 19 | 0.25 (-7.14 to 8.22) |
| Oman | 20 to 24 | 0.68 (-4.65 to 6.3) |
| Oman | 25 to 29 | 0.96 (-3.64 to 5.78) |
| Oman | 30 to 34 | 0.86 (-3.61 to 5.53) |
| Oman | 35 to 39 | 0.67 (-4.44 to 6.05) |
| Pakistan | 15 to 19 | 0.5 (-0.12 to 1.13) |
| Pakistan | 20 to 24 | 0.49 (-0.02 to 1) |
| Pakistan | 25 to 29 | 0.39 (-0.06 to 0.84) |
| Pakistan | 30 to 34 | 0.21 (-0.2 to 0.64) |
| Pakistan | 35 to 39 | -0.01 (-0.49 to 0.47) |
| Palestine | 15 to 19 | -3.49 (-10.92 to 4.56) |
| Palestine | 20 to 24 | -2.55 (-8.32 to 3.59) |
| Palestine | 25 to 29 | -1.13 (-6.23 to 4.26) |
| Palestine | 30 to 34 | -0.63 (-6.06 to 5.11) |
| Palestine | 35 to 39 | -0.46 (-7.51 to 7.14) |
| Panama | 15 to 19 | -0.79 (-8.36 to 7.42) |
| Panama | 20 to 24 | -0.46 (-6.27 to 5.71) |
| Panama | 25 to 29 | 0.67 (-4.4 to 6.01) |
| Panama | 30 to 34 | 1.44 (-3.96 to 7.14) |
| Panama | 35 to 39 | 1.68 (-5.48 to 9.37) |
| Papua New Guinea | 15 to 19 | -0.95 (-18.81 to 20.85) |
| Papua New Guinea | 20 to 24 | 0 (-4.86 to 5.11) |
| Papua New Guinea | 25 to 29 | -0.04 (-4.19 to 4.29) |
| Papua New Guinea | 30 to 34 | 0.08 (-3.45 to 3.74) |
| Papua New Guinea | 35 to 39 | -0.7 (-4.13 to 2.84) |
| Paraguay | 15 to 19 | 1.57 (-5.09 to 8.71) |
| Paraguay | 20 to 24 | 0.52 (-4.89 to 6.23) |
| Paraguay | 25 to 29 | 0.42 (-5.8 to 7.05) |
| Paraguay | 30 to 34 | 0.64 (-5.18 to 6.81) |
| Paraguay | 35 to 39 | 1.05 (-6.07 to 8.7) |
| Peru | 15 to 19 | 2.64 (-1.65 to 7.12) |
| Peru | 20 to 24 | 2.18 (-1.39 to 5.88) |
| Peru | 25 to 29 | 1.94 (-1.33 to 5.32) |
| Peru | 30 to 34 | 1.57 (-1.53 to 4.76) |
| Peru | 35 to 39 | 0.92 (-2.77 to 4.76) |
| Philippines | 15 to 19 | 0.14 (-0.73 to 1.01) |
| Philippines | 20 to 24 | -0.12 (-0.75 to 0.52) |
| Philippines | 25 to 29 | -0.36 (-0.86 to 0.13) |
| Philippines | 30 to 34 | -0.44 (-0.85 to -0.03) |
| Philippines | 35 to 39 | -0.4 (-0.86 to 0.06) |
| Poland | 15 to 19 | 2.41 (-0.59 to 5.5) |
| Poland | 20 to 24 | 2.7 (0.64 to 4.81) |
| Poland | 25 to 29 | 2.35 (0.69 to 4.03) |
| Poland | 30 to 34 | 1.8 (0.44 to 3.18) |
| Poland | 35 to 39 | 0.98 (-0.38 to 2.35) |
| Portugal | 15 to 19 | 0.1 (-3.13 to 3.44) |
| Portugal | 20 to 24 | -0.37 (-2.66 to 1.96) |
| Portugal | 25 to 29 | -0.65 (-2.48 to 1.22) |
| Portugal | 30 to 34 | -0.54 (-2.1 to 1.06) |
| Portugal | 35 to 39 | -0.47 (-2.21 to 1.31) |
| Puerto Rico | 15 to 19 | -0.46 (-7.02 to 6.57) |
| Puerto Rico | 20 to 24 | 1.26 (-2.87 to 5.57) |
| Puerto Rico | 25 to 29 | 1.71 (-1.71 to 5.25) |
| Puerto Rico | 30 to 34 | 1.76 (-1.62 to 5.25) |
| Puerto Rico | 35 to 39 | 1.87 (-2.57 to 6.51) |
| Qatar | 15 to 19 | 3.9 (-8.87 to 18.46) |
| Qatar | 20 to 24 | 4.53 (-6.58 to 16.97) |
| Qatar | 25 to 29 | 2.87 (-5.99 to 12.57) |
| Qatar | 30 to 34 | 0.52 (-5.29 to 6.69) |
| Qatar | 35 to 39 | -0.8 (-7.46 to 6.35) |
| Republic of Korea | 15 to 19 | 1.85 (-0.47 to 4.22) |
| Republic of Korea | 20 to 24 | 1.8 (0.26 to 3.36) |
| Republic of Korea | 25 to 29 | 1.61 (0.41 to 2.83) |
| Republic of Korea | 30 to 34 | 1.82 (0.82 to 2.83) |
| Republic of Korea | 35 to 39 | 2.16 (1.06 to 3.27) |
| Republic of Moldova | 15 to 19 | -0.45 (-7.31 to 6.92) |
| Republic of Moldova | 20 to 24 | -0.86 (-6.06 to 4.62) |
| Republic of Moldova | 25 to 29 | -1.04 (-5.33 to 3.45) |
| Republic of Moldova | 30 to 34 | -0.45 (-4.16 to 3.4) |
| Republic of Moldova | 35 to 39 | -0.21 (-4.22 to 3.95) |
| Romania | 15 to 19 | 3.61 (0.59 to 6.72) |
| Romania | 20 to 24 | 3.36 (1.07 to 5.71) |
| Romania | 25 to 29 | 3.07 (1.23 to 4.94) |
| Romania | 30 to 34 | 2.58 (1 to 4.19) |
| Romania | 35 to 39 | 2.32 (0.67 to 4) |
| Russian Federation | 15 to 19 | -0.73 (-2.5 to 1.08) |
| Russian Federation | 20 to 24 | -0.31 (-1.59 to 0.99) |
| Russian Federation | 25 to 29 | 0.35 (-0.68 to 1.4) |
| Russian Federation | 30 to 34 | 1.15 (0.3 to 2.01) |
| Russian Federation | 35 to 39 | 1.56 (0.68 to 2.45) |
| Rwanda | 15 to 19 | -1.69 (-4.16 to 0.85) |
| Rwanda | 20 to 24 | -1.67 (-3.74 to 0.45) |
| Rwanda | 25 to 29 | -1.58 (-3.37 to 0.23) |
| Rwanda | 30 to 34 | -1.76 (-3.38 to -0.1) |
| Rwanda | 35 to 39 | -1.94 (-3.74 to -0.1) |
| Samoa | 15 to 19 | -0.41 (-22.24 to 27.54) |
| Samoa | 20 to 24 | -0.34 (-17.87 to 20.93) |
| Samoa | 25 to 29 | -0.29 (-15.63 to 17.84) |
| Samoa | 30 to 34 | -0.37 (-13.47 to 14.72) |
| Samoa | 35 to 39 | -0.84 (-9.52 to 8.68) |
| Saudi Arabia | 15 to 19 | 2.1 (0.46 to 3.76) |
| Saudi Arabia | 20 to 24 | 2.44 (1.18 to 3.71) |
| Saudi Arabia | 25 to 29 | 2.52 (1.47 to 3.59) |
| Saudi Arabia | 30 to 34 | 2.25 (1.35 to 3.15) |
| Saudi Arabia | 35 to 39 | 1.54 (0.53 to 2.57) |
| Senegal | 15 to 19 | 1.81 (-7.43 to 11.96) |
| Senegal | 20 to 24 | 1.89 (-4.43 to 8.63) |
| Senegal | 25 to 29 | 2.02 (-4.44 to 8.91) |
| Senegal | 30 to 34 | 2.43 (-3.17 to 8.34) |
| Senegal | 35 to 39 | 1.92 (-5.05 to 9.41) |
| Serbia | 15 to 19 | -0.51 (-7.11 to 6.56) |
| Serbia | 20 to 24 | 1.07 (-3.41 to 5.76) |
| Serbia | 25 to 29 | 1.63 (-1.87 to 5.26) |
| Serbia | 30 to 34 | 1.49 (-1.48 to 4.55) |
| Serbia | 35 to 39 | 0.95 (-2.12 to 4.1) |
| Seychelles | 15 to 19 | 0.17 (-21.78 to 28.29) |
| Seychelles | 20 to 24 | -0.15 (-17.72 to 21.16) |
| Seychelles | 25 to 29 | -0.53 (-15.84 to 17.55) |
| Seychelles | 30 to 34 | -0.89 (-13.92 to 14.12) |
| Seychelles | 35 to 39 | 5.17 (-13.14 to 27.36) |
| Sierra Leone | 15 to 19 | 0.43 (-19.74 to 25.65) |
| Sierra Leone | 20 to 24 | 3.23 (-9.85 to 18.22) |
| Sierra Leone | 25 to 29 | 4.2 (-6.82 to 16.52) |
| Sierra Leone | 30 to 34 | 3.98 (-6.73 to 15.92) |
| Sierra Leone | 35 to 39 | -0.85 (-8.6 to 7.56) |
| Singapore | 15 to 19 | 2.58 (0.47 to 4.74) |
| Singapore | 20 to 24 | 1.25 (-0.21 to 2.74) |
| Singapore | 25 to 29 | -0.2 (-1.29 to 0.9) |
| Singapore | 30 to 34 | -1.67 (-2.56 to -0.77) |
| Singapore | 35 to 39 | -3.21 (-4.15 to -2.26) |
| Slovakia | 15 to 19 | 2.66 (-4.98 to 10.92) |
| Slovakia | 20 to 24 | 1.87 (-3.82 to 7.9) |
| Slovakia | 25 to 29 | 1.58 (-2.95 to 6.31) |
| Slovakia | 30 to 34 | 1.52 (-2.17 to 5.34) |
| Slovakia | 35 to 39 | 0.09 (-3.39 to 3.7) |
| Slovenia | 15 to 19 | -7.61 (-24.28 to 12.74) |
| Slovenia | 20 to 24 | -5.2 (-17.06 to 8.37) |
| Slovenia | 25 to 29 | 0.05 (-5.23 to 5.63) |
| Slovenia | 30 to 34 | -1.03 (-5.82 to 4.01) |
| Slovenia | 35 to 39 | -1.01 (-6.08 to 4.34) |
| Solomon Islands | 15 to 19 | -1.66 (-23.21 to 25.94) |
| Solomon Islands | 20 to 24 | -1.7 (-18.99 to 19.28) |
| Solomon Islands | 25 to 29 | -2.13 (-17.19 to 15.66) |
| Solomon Islands | 30 to 34 | -2.77 (-15.56 to 11.95) |
| Solomon Islands | 35 to 39 | 3.16 (-14.81 to 24.92) |
| Somalia | 15 to 19 | -0.31 (-2.77 to 2.21) |
| Somalia | 20 to 24 | -0.06 (-2.25 to 2.17) |
| Somalia | 25 to 29 | -0.07 (-2.15 to 2.06) |
| Somalia | 30 to 34 | -0.15 (-2.08 to 1.82) |
| Somalia | 35 to 39 | -0.44 (-2.39 to 1.55) |
| South Africa | 15 to 19 | -0.68 (-2.97 to 1.67) |
| South Africa | 20 to 24 | -1.03 (-2.71 to 0.68) |
| South Africa | 25 to 29 | -1.22 (-2.61 to 0.19) |
| South Africa | 30 to 34 | -1.15 (-2.41 to 0.12) |
| South Africa | 35 to 39 | -1.1 (-2.56 to 0.38) |
| South Sudan | 15 to 19 | 1.3 (-2.09 to 4.81) |
| South Sudan | 20 to 24 | 0.91 (-2.04 to 3.95) |
| South Sudan | 25 to 29 | 0.63 (-1.99 to 3.31) |
| South Sudan | 30 to 34 | 0.35 (-2.09 to 2.85) |
| South Sudan | 35 to 39 | 0.14 (-2.59 to 2.94) |
| Spain | 15 to 19 | -1.78 (-3.17 to -0.38) |
| Spain | 20 to 24 | -1.51 (-2.47 to -0.55) |
| Spain | 25 to 29 | -1.6 (-2.32 to -0.87) |
| Spain | 30 to 34 | -1.83 (-2.43 to -1.23) |
| Spain | 35 to 39 | -1.82 (-2.5 to -1.15) |
| Sri Lanka | 15 to 19 | 0.81 (-1.16 to 2.82) |
| Sri Lanka | 20 to 24 | 0.85 (-0.57 to 2.3) |
| Sri Lanka | 25 to 29 | 0.47 (-0.68 to 1.63) |
| Sri Lanka | 30 to 34 | 0.28 (-0.71 to 1.28) |
| Sri Lanka | 35 to 39 | 0.25 (-0.9 to 1.41) |
| Sudan | 15 to 19 | -0.3 (-3.04 to 2.5) |
| Sudan | 20 to 24 | -0.36 (-2.55 to 1.88) |
| Sudan | 25 to 29 | -0.67 (-2.58 to 1.28) |
| Sudan | 30 to 34 | -0.93 (-2.73 to 0.91) |
| Sudan | 35 to 39 | -1.36 (-3.48 to 0.82) |
| Suriname | 15 to 19 | -1.75 (-23.38 to 25.99) |
| Suriname | 20 to 24 | -1.88 (-19.3 to 19.28) |
| Suriname | 25 to 29 | 0.73 (-15.59 to 20.21) |
| Suriname | 30 to 34 | 3.89 (-13.08 to 24.17) |
| Suriname | 35 to 39 | 8.71 (-11.72 to 33.89) |
| Sweden | 15 to 19 | -1.58 (-6.31 to 3.39) |
| Sweden | 20 to 24 | -0.6 (-3.56 to 2.46) |
| Sweden | 25 to 29 | -0.49 (-2.35 to 1.39) |
| Sweden | 30 to 34 | -1.27 (-2.89 to 0.38) |
| Sweden | 35 to 39 | -1.44 (-3.81 to 0.98) |
| Switzerland | 15 to 19 | -3.14 (-7.91 to 1.87) |
| Switzerland | 20 to 24 | -4.15 (-7.53 to -0.65) |
| Switzerland | 25 to 29 | -3.86 (-6.34 to -1.31) |
| Switzerland | 30 to 34 | -3.83 (-5.95 to -1.66) |
| Switzerland | 35 to 39 | -3.68 (-6.07 to -1.24) |
| Syrian Arab Republic | 15 to 19 | 1.2 (-3.68 to 6.33) |
| Syrian Arab Republic | 20 to 24 | 1.15 (-2.98 to 5.45) |
| Syrian Arab Republic | 25 to 29 | 0.99 (-2.86 to 4.99) |
| Syrian Arab Republic | 30 to 34 | -0.06 (-3.5 to 3.51) |
| Syrian Arab Republic | 35 to 39 | -0.49 (-4.81 to 4.01) |
| Taiwan (Province of China) | 15 to 19 | -1.8 (-2.65 to -0.95) |
| Taiwan (Province of China) | 20 to 24 | -1.95 (-2.48 to -1.41) |
| Taiwan (Province of China) | 25 to 29 | -1.82 (-2.2 to -1.44) |
| Taiwan (Province of China) | 30 to 34 | -1.27 (-1.54 to -1) |
| Taiwan (Province of China) | 35 to 39 | -0.68 (-0.95 to -0.41) |
| Tajikistan | 15 to 19 | -1.47 (-5.39 to 2.61) |
| Tajikistan | 20 to 24 | -1.31 (-4.4 to 1.89) |
| Tajikistan | 25 to 29 | -1.15 (-3.87 to 1.64) |
| Tajikistan | 30 to 34 | -0.86 (-3.72 to 2.09) |
| Tajikistan | 35 to 39 | -0.83 (-4.57 to 3.07) |
| Thailand | 15 to 19 | 0.95 (0.1 to 1.81) |
| Thailand | 20 to 24 | 1.06 (0.46 to 1.67) |
| Thailand | 25 to 29 | 1.34 (0.87 to 1.81) |
| Thailand | 30 to 34 | 1.74 (1.35 to 2.14) |
| Thailand | 35 to 39 | 1.86 (1.42 to 2.32) |
| Timor-Leste | 15 to 19 | 5.34 (-8.4 to 21.15) |
| Timor-Leste | 20 to 24 | 3.33 (-6.71 to 14.45) |
| Timor-Leste | 25 to 29 | 1.02 (-6.05 to 8.61) |
| Timor-Leste | 30 to 34 | 1.13 (-4.7 to 7.31) |
| Timor-Leste | 35 to 39 | -0.1 (-6.46 to 6.69) |
| Togo | 15 to 19 | -3.16 (-21.39 to 19.29) |
| Togo | 20 to 24 | -1.73 (-9.69 to 6.93) |
| Togo | 25 to 29 | 0.12 (-7.23 to 8.06) |
| Togo | 30 to 34 | 0.12 (-5.89 to 6.52) |
| Togo | 35 to 39 | -0.62 (-7.87 to 7.19) |
| Trinidad and Tobago | 15 to 19 | 4.67 (-17.7 to 33.13) |
| Trinidad and Tobago | 20 to 24 | 5.13 (-11.84 to 25.36) |
| Trinidad and Tobago | 25 to 29 | 8.28 (-5.4 to 23.93) |
| Trinidad and Tobago | 30 to 34 | 5.06 (-6.15 to 17.6) |
| Trinidad and Tobago | 35 to 39 | 6.74 (-11.76 to 29.11) |
| Tunisia | 15 to 19 | 1.65 (0.02 to 3.31) |
| Tunisia | 20 to 24 | 1.68 (0.43 to 2.95) |
| Tunisia | 25 to 29 | 1.55 (0.54 to 2.58) |
| Tunisia | 30 to 34 | 1.46 (0.59 to 2.34) |
| Tunisia | 35 to 39 | 1.16 (0.13 to 2.21) |
| Turkey | 15 to 19 | 0.95 (-0.09 to 2) |
| Turkey | 20 to 24 | 1.01 (0.2 to 1.83) |
| Turkey | 25 to 29 | 0.9 (0.18 to 1.62) |
| Turkey | 30 to 34 | 0.56 (-0.1 to 1.24) |
| Turkey | 35 to 39 | 0 (-0.79 to 0.81) |
| Turkmenistan | 15 to 19 | 1.45 (-3.54 to 6.7) |
| Turkmenistan | 20 to 24 | 1.41 (-2.53 to 5.51) |
| Turkmenistan | 25 to 29 | 1.81 (-1.83 to 5.58) |
| Turkmenistan | 30 to 34 | 2.07 (-1.85 to 6.16) |
| Turkmenistan | 35 to 39 | 2.17 (-2.99 to 7.61) |
| Uganda | 15 to 19 | -0.33 (-1.39 to 0.73) |
| Uganda | 20 to 24 | -0.23 (-1.13 to 0.68) |
| Uganda | 25 to 29 | -0.19 (-1.02 to 0.64) |
| Uganda | 30 to 34 | -0.24 (-1.05 to 0.57) |
| Uganda | 35 to 39 | -0.45 (-1.41 to 0.53) |
| Ukraine | 15 to 19 | 1.2 (-1.39 to 3.86) |
| Ukraine | 20 to 24 | 0.85 (-1.05 to 2.79) |
| Ukraine | 25 to 29 | 0.9 (-0.63 to 2.45) |
| Ukraine | 30 to 34 | 1.41 (0.1 to 2.74) |
| Ukraine | 35 to 39 | 2.1 (0.67 to 3.56) |
| United Arab Emirates | 15 to 19 | 0.88 (-4.48 to 6.54) |
| United Arab Emirates | 20 to 24 | 1.7 (-2.07 to 5.61) |
| United Arab Emirates | 25 to 29 | 1.03 (-1.87 to 4.01) |
| United Arab Emirates | 30 to 34 | -0.07 (-2.66 to 2.6) |
| United Arab Emirates | 35 to 39 | -1.1 (-4.13 to 2.03) |
| United Kingdom | 15 to 19 | -0.57 (-1.6 to 0.47) |
| United Kingdom | 20 to 24 | -0.09 (-0.83 to 0.64) |
| United Kingdom | 25 to 29 | 0.57 (-0.02 to 1.17) |
| United Kingdom | 30 to 34 | 1.02 (0.46 to 1.58) |
| United Kingdom | 35 to 39 | 1.35 (0.63 to 2.08) |
| United Republic of Tanzania | 15 to 19 | -0.03 (-1.39 to 1.35) |
| United Republic of Tanzania | 20 to 24 | 0 (-1.14 to 1.15) |
| United Republic of Tanzania | 25 to 29 | -0.05 (-1.06 to 0.97) |
| United Republic of Tanzania | 30 to 34 | -0.19 (-1.13 to 0.77) |
| United Republic of Tanzania | 35 to 39 | -0.34 (-1.44 to 0.76) |
| United States of America | 15 to 19 | -1.42 (-2.01 to -0.82) |
| United States of America | 20 to 24 | -0.63 (-1.04 to -0.22) |
| United States of America | 25 to 29 | 0.14 (-0.18 to 0.46) |
| United States of America | 30 to 34 | 0.1 (-0.19 to 0.38) |
| United States of America | 35 to 39 | -0.25 (-0.56 to 0.07) |
| Uruguay | 15 to 19 | 1.8 (-5.71 to 9.91) |
| Uruguay | 20 to 24 | 3.11 (-2.14 to 8.65) |
| Uruguay | 25 to 29 | 2.09 (-2.63 to 7.04) |
| Uruguay | 30 to 34 | 0.9 (-3.6 to 5.61) |
| Uruguay | 35 to 39 | 1.15 (-4.25 to 6.85) |
| Uzbekistan | 15 to 19 | 0.96 (-1.21 to 3.19) |
| Uzbekistan | 20 to 24 | 1.14 (-0.56 to 2.86) |
| Uzbekistan | 25 to 29 | 0.98 (-0.53 to 2.5) |
| Uzbekistan | 30 to 34 | 0.97 (-0.6 to 2.56) |
| Uzbekistan | 35 to 39 | 0.96 (-1.15 to 3.11) |
| Venezuela (Bolivarian Republic of) | 15 to 19 | 3.7 (0.7 to 6.78) |
| Venezuela (Bolivarian Republic of) | 20 to 24 | 3.82 (1.4 to 6.29) |
| Venezuela (Bolivarian Republic of) | 25 to 29 | 3.36 (1.14 to 5.63) |
| Venezuela (Bolivarian Republic of) | 30 to 34 | 2.69 (0.46 to 4.97) |
| Venezuela (Bolivarian Republic of) | 35 to 39 | 1.95 (-0.93 to 4.92) |
| Viet Nam | 15 to 19 | 2.78 (2.24 to 3.31) |
| Viet Nam | 20 to 24 | 2.5 (2.07 to 2.93) |
| Viet Nam | 25 to 29 | 2.14 (1.79 to 2.5) |
| Viet Nam | 30 to 34 | 1.94 (1.64 to 2.25) |
| Viet Nam | 35 to 39 | 1.87 (1.52 to 2.22) |
| Yemen | 15 to 19 | -0.46 (-5.19 to 4.5) |
| Yemen | 20 to 24 | -0.05 (-3.85 to 3.91) |
| Yemen | 25 to 29 | 0.15 (-3.02 to 3.42) |
| Yemen | 30 to 34 | -0.43 (-3.08 to 2.29) |
| Yemen | 35 to 39 | -1.27 (-4.1 to 1.64) |
| Zambia | 15 to 19 | 1.43 (-0.7 to 3.6) |
| Zambia | 20 to 24 | 1.64 (-0.16 to 3.48) |
| Zambia | 25 to 29 | 1.95 (0.31 to 3.62) |
| Zambia | 30 to 34 | 2.31 (0.69 to 3.95) |
| Zambia | 35 to 39 | 2.38 (0.48 to 4.31) |
| Zimbabwe | 15 to 19 | 3.06 (-0.39 to 6.63) |
| Zimbabwe | 20 to 24 | 2.95 (-0.03 to 6.02) |
| Zimbabwe | 25 to 29 | 2.78 (-0.01 to 5.65) |
| Zimbabwe | 30 to 34 | 2.94 (0.24 to 5.7) |
| Zimbabwe | 35 to 39 | 2.75 (-0.39 to 5.99) |
