# Supplementary material for: Global, regional, and national temporal trends in prevalence for nasopharynx cancer across adolescents and young adults, 1990–2021: an age-period-cohort analysis based on the global burden of disease study 2021
Source: BMC Oral Health. 2025 Sep 26;25:1435. doi: 10.1186/s12903-025-06750-4 (PMC12465747; doi:10.1186/s12903-025-06750-4)
Supplement: Supplementary file 4 — Supplementary Material 4. Age effects on nasopharynx cancer prevalence in adolescents and young adults across SDI quintiles. [file 12903_2025_6750_MOESM4_ESM.docx]

**Supplementary Table 4** Age effects on nasopharynx cancer prevalence in adolescents and young adults across SDI quintiles

| **Location** | **Age** | **Prevalence rate (per 100,000)** |
| --- | --- | --- |
| Global | 15 to 19 | 1.20 (1.11, 1.31) |
| Global | 20 to 24 | 1.58 (1.47, 1.69) |
| Global | 25 to 29 | 2.70 (2.55, 2.87) |
| Global | 30 to 34 | 5.68 (5.41, 5.97) |
| Global | 35 to 39 | 9.46 (8.92, 10.04) |
| High SDI | 15 to 19 | 1.23 (1.16, 1.31) |
| High SDI | 20 to 24 | 1.63 (1.55, 1.72) |
| High SDI | 25 to 29 | 2.35 (2.24, 2.46) |
| High SDI | 30 to 34 | 3.99 (3.83, 4.16) |
| High SDI | 35 to 39 | 5.92 (5.65, 6.20) |
| High-middle SDI | 15 to 19 | 2.35 (2.04, 2.70) |
| High-middle SDI | 20 to 24 | 3.42 (3.04, 3.85) |
| High-middle SDI | 25 to 29 | 6.08 (5.52, 6.70) |
| High-middle SDI | 30 to 34 | 14.3 (13.24, 15.45) |
| High-middle SDI | 35 to 39 | 24.8 (22.58, 27.25) |
| Middle SDI | 15 to 19 | 1.31 (1.22, 1.41) |
| Middle SDI | 20 to 24 | 1.66 (1.56, 1.78) |
| Middle SDI | 25 to 29 | 2.99 (2.83, 3.15) |
| Middle SDI | 30 to 34 | 6.21 (5.93, 6.50) |
| Middle SDI | 35 to 39 | 9.93 (9.41, 10.47) |
| Low-middle SDI | 15 to 19 | 0.55 (0.53, 0.57) |
| Low-middle SDI | 20 to 24 | 0.59 (0.57, 0.61) |
| Low-middle SDI | 25 to 29 | 0.90 (0.87, 0.93) |
| Low-middle SDI | 30 to 34 | 1.52 (1.48, 1.57) |
| Low-middle SDI | 35 to 39 | 2.80 (2.70, 2.91) |
| Low SDI | 15 to 19 | 0.51 (0.48, 0.55) |
| Low SDI | 20 to 24 | 0.55 (0.51, 0.58) |
| Low SDI | 25 to 29 | 0.73 (0.69, 0.77) |
| Low SDI | 30 to 34 | 1.17 (1.11, 1.23) |
| Low SDI | 35 to 39 | 2.17 (2.04, 2.29) |
