# Supplementary material for: Global, regional, and national temporal trends in prevalence for nasopharynx cancer across adolescents and young adults, 1990–2021: an age-period-cohort analysis based on the global burden of disease study 2021
Source: BMC Oral Health. 2025 Sep 26;25:1435. doi: 10.1186/s12903-025-06750-4 (PMC12465747; doi:10.1186/s12903-025-06750-4)
Supplement: Supplementary file 5 — Supplementary Material 5. Period effects on nasopharynx cancer prevalence in adolescents and young adults across SDI quintiles. [file 12903_2025_6750_MOESM5_ESM.docx]

**Supplementary Table 5** Period effects on nasopharynx cancer prevalence in adolescents and young adults across SDI quintiles

| **Location** | **Period** | **Prevalence rate ratio** |
| --- | --- | --- |
| Global | 1992 to 1996 | 1.13 (1.05, 1.22) |
| Global | 1997 to 2001 | 1.12 (1.05, 1.19) |
| Global | 2002 to 2006 | 1.00 (1.00, 1.00) |
| Global | 2007 to 2011 | 0.99 (0.93, 1.05) |
| Global | 2012 to 2016 | 1.06 (1.00, 1.13) |
| Global | 2017 to 2021 | 1.26 (1.19, 1.34) |
| High SDI | 1992 to 1996 | 1.00 (0.95, 1.06) |
| High SDI | 1997 to 2001 | 1.05 (1.00, 1.10) |
| High SDI | 2002 to 2006 | 1.00 (1.00, 1.00) |
| High SDI | 2007 to 2011 | 0.98 (0.94, 1.03) |
| High SDI | 2012 to 2016 | 0.98 (0.93, 1.02) |
| High SDI | 2017 to 2021 | 0.96 (0.92, 1.01) |
| High-middle SDI | 1992 to 1996 | 1.01 (0.89, 1.14) |
| High-middle SDI | 1997 to 2001 | 1.08 (0.97, 1.19) |
| High-middle SDI | 2002 to 2006 | 1.00 (1.00, 1.00) |
| High-middle SDI | 2007 to 2011 | 1.02 (0.92, 1.12) |
| High-middle SDI | 2012 to 2016 | 1.22 (1.10, 1.35) |
| High-middle SDI | 2017 to 2021 | 1.69 (1.53, 1.86) |
| Middle SDI | 1992 to 1996 | 1.18 (1.10, 1.25) |
| Middle SDI | 1997 to 2001 | 1.13 (1.07, 1.19) |
| Middle SDI | 2002 to 2006 | 1.00 (1.00, 1.00) |
| Middle SDI | 2007 to 2011 | 1.03 (0.97, 1.08) |
| Middle SDI | 2012 to 2016 | 1.10 (1.04, 1.17) |
| Middle SDI | 2017 to 2021 | 1.28 (1.21, 1.35) |
| Low-middle SDI | 1992 to 1996 | 1.05 (1.00, 1.09) |
| Low-middle SDI | 1997 to 2001 | 1.05 (1.02, 1.09) |
| Low-middle SDI | 2002 to 2006 | 1.00 (1.00, 1.00) |
| Low-middle SDI | 2007 to 2011 | 1.01 (0.98, 1.05) |
| Low-middle SDI | 2012 to 2016 | 1.01 (0.98, 1.05) |
| Low-middle SDI | 2017 to 2021 | 1.05 (1.02, 1.09) |
| Low SDI | 1992 to 1996 | 1.09 (1.02, 1.17) |
| Low SDI | 1997 to 2001 | 1.10 (1.03, 1.17) |
| Low SDI | 2002 to 2006 | 1.00 (1.00, 1.00) |
| Low SDI | 2007 to 2011 | 0.97 (0.91, 1.02) |
| Low SDI | 2012 to 2016 | 0.96 (0.91, 1.02) |
| Low SDI | 2017 to 2021 | 0.99 (0.94, 1.05) |
