# Supplementary material for: Global, regional, and national temporal trends in prevalence for nasopharynx cancer across adolescents and young adults, 1990–2021: an age-period-cohort analysis based on the global burden of disease study 2021
Source: BMC Oral Health. 2025 Sep 26;25:1435. doi: 10.1186/s12903-025-06750-4 (PMC12465747; doi:10.1186/s12903-025-06750-4)
Supplement: Supplementary file 6 — Supplementary Material 6. Cohort effects on nasopharynx cancer prevalence in adolescents and young adults across SDI quintiles. [file 12903_2025_6750_MOESM6_ESM.docx]

**Supplementary Table 6** Cohort effects on nasopharynx cancer prevalence in adolescents and young adults across SDI quintiles

| **Location** | **Birth cohort** | **Prevalence rate ratio** |
| --- | --- | --- |
| Global | 1952 to 1961 | 0.79 (0.72, 0.87) |
| Global | 1957 to 1966 | 0.78 (0.72, 0.84) |
| Global | 1962 to 1971 | 0.86 (0.8, 0.92) |
| Global | 1967 to 1976 | 0.88 (0.82, 0.94) |
| Global | 1972 to 1981 | 0.86 (0.8, 0.91) |
| Global | 1977 to 1986 | 0.89 (0.84, 0.94) |
| Global | 1982 to 1991 | 1.00 (1.00, 1.00) |
| Global | 1987 to 1996 | 0.95 (0.88, 1.03) |
| Global | 1992 to 2001 | 0.82 (0.73, 0.92) |
| Global | 1997 to 2006 | 0.71 (0.60, 0.85) |
| High SDI | 1952 to 1961 | 1.05 (0.98, 1.13) |
| High SDI | 1957 to 1966 | 1.00 (0.94, 1.06) |
| High SDI | 1962 to 1971 | 0.95 (0.90, 1.01) |
| High SDI | 1967 to 1976 | 0.94 (0.89, 0.99) |
| High SDI | 1972 to 1981 | 0.98 (0.93, 1.03) |
| High SDI | 1977 to 1986 | 0.99 (0.95, 1.04) |
| High SDI | 1982 to 1991 | 1.00 (1.00, 1.00) |
| High SDI | 1987 to 1996 | 0.97 (0.91, 1.04) |
| High SDI | 1992 to 2001 | 0.86 (0.79, 0.94) |
| High SDI | 1997 to 2006 | 0.78 (0.68, 0.90) |
| High-middle SDI | 1952 to 1961 | 0.50 (0.43, 0.59) |
| High-middle SDI | 1957 to 1966 | 0.52 (0.46, 0.59) |
| High-middle SDI | 1962 to 1971 | 0.63 (0.56, 0.71) |
| High-middle SDI | 1967 to 1976 | 0.71 (0.63, 0.79) |
| High-middle SDI | 1972 to 1981 | 0.74 (0.66, 0.82) |
| High-middle SDI | 1977 to 1986 | 0.84 (0.76, 0.92) |
| High-middle SDI | 1982 to 1991 | 1.00 (1.00, 1.00) |
| High-middle SDI | 1987 to 1996 | 1.08 (0.94, 1.23) |
| High-middle SDI | 1992 to 2001 | 0.95 (0.78, 1.17) |
| High-middle SDI | 1997 to 2006 | 0.76 (0.55, 1.07) |
| Middle SDI | 1952 to 1961 | 0.89 (0.81, 0.96) |
| Middle SDI | 1957 to 1966 | 0.83 (0.78, 0.89) |
| Middle SDI | 1962 to 1971 | 0.91 (0.86, 0.97) |
| Middle SDI | 1967 to 1976 | 0.90 (0.84, 0.95) |
| Middle SDI | 1972 to 1981 | 0.88 (0.83, 0.93) |
| Middle SDI | 1977 to 1986 | 0.89 (0.84, 0.94) |
| Middle SDI | 1982 to 1991 | 1.00 (1.00, 1.00) |
| Middle SDI | 1987 to 1996 | 0.97 (0.90, 1.05) |
| Middle SDI | 1992 to 2001 | 0.91 (0.82, 1.01) |
| Middle SDI | 1997 to 2006 | 0.85 (0.72, 0.99) |
| Low-middle SDI | 1952 to 1961 | 1.03 (0.97, 1.09) |
| Low-middle SDI | 1957 to 1966 | 1.01 (0.97, 1.06) |
| Low-middle SDI | 1962 to 1971 | 1.00 (0.96, 1.05) |
| Low-middle SDI | 1967 to 1976 | 1.00 (0.96, 1.04) |
| Low-middle SDI | 1972 to 1981 | 0.99 (0.95, 1.03) |
| Low-middle SDI | 1977 to 1986 | 0.99 (0.95, 1.02) |
| Low-middle SDI | 1982 to 1991 | 1.00 (1.00, 1.00) |
| Low-middle SDI | 1987 to 1996 | 1.00 (0.96, 1.05) |
| Low-middle SDI | 1992 to 2001 | 0.99 (0.94, 1.05) |
| Low-middle SDI | 1997 to 2006 | 0.98 (0.91, 1.06) |
| Low SDI | 1952 to 1961 | 1.18 (1.07, 1.30) |
| Low SDI | 1957 to 1966 | 1.10 (1.02, 1.19) |
| Low SDI | 1962 to 1971 | 1.07 (1.00, 1.15) |
| Low SDI | 1967 to 1976 | 1.04 (0.98, 1.11) |
| Low SDI | 1972 to 1981 | 1.02 (0.96, 1.09) |
| Low SDI | 1977 to 1986 | 1.00 (0.94, 1.06) |
| Low SDI | 1982 to 1991 | 1.00 (1.00, 1.00) |
| Low SDI | 1987 to 1996 | 0.95 (0.89, 1.02) |
| Low SDI | 1992 to 2001 | 0.92 (0.84, 1.00) |
| Low SDI | 1997 to 2006 | 0.87 (0.78, 0.97) |
