# Supplementary material for: Global, regional, and national temporal trends in prevalence for nasopharynx cancer across adolescents and young adults, 1990–2021: an age-period-cohort analysis based on the global burden of disease study 2021
Source: BMC Oral Health. 2025 Sep 26;25:1435. doi: 10.1186/s12903-025-06750-4 (PMC12465747; doi:10.1186/s12903-025-06750-4)
Supplement: Supplementary file 7 — Supplementary Material 7. Age effects on nasopharynx cancer prevalence in adolescents and young adults across countries. [file 12903_2025_6750_MOESM7_ESM.docx]

**Supplementary Table 7** Age effects on nasopharynx cancer prevalence in adolescents and young adults across countries

| **Location** | **Age** | **Prevalence rate (per 100,000)** |
| --- | --- | --- |
| Afghanistan | 15 to 19 | 0.3 (0.18, 0.51) |
| Afghanistan | 20 to 24 | 0.31 (0.19, 0.5) |
| Afghanistan | 25 to 29 | 0.47 (0.3, 0.74) |
| Afghanistan | 30 to 34 | 0.69 (0.45, 1.07) |
| Afghanistan | 35 to 39 | 1.13 (0.69, 1.85) |
| Albania | 15 to 19 | 0.57 (0.23, 1.42) |
| Albania | 20 to 24 | 0.75 (0.32, 1.77) |
| Albania | 25 to 29 | 1 (0.43, 2.34) |
| Albania | 30 to 34 | 1.69 (0.75, 3.82) |
| Albania | 35 to 39 | 2.39 (0.94, 6.07) |
| Algeria | 15 to 19 | 2.19 (1.92, 2.5) |
| Algeria | 20 to 24 | 2.25 (1.99, 2.56) |
| Algeria | 25 to 29 | 3.34 (2.98, 3.74) |
| Algeria | 30 to 34 | 5.93 (5.36, 6.56) |
| Algeria | 35 to 39 | 9.81 (8.72, 11.04) |
| Angola | 15 to 19 | 0.13 (0.06, 0.28) |
| Angola | 20 to 24 | 0.17 (0.08, 0.33) |
| Angola | 25 to 29 | 0.2 (0.11, 0.39) |
| Angola | 30 to 34 | 0.31 (0.17, 0.57) |
| Angola | 35 to 39 | 0.62 (0.31, 1.23) |
| Argentina | 15 to 19 | 0.29 (0.2, 0.43) |
| Argentina | 20 to 24 | 0.33 (0.23, 0.47) |
| Argentina | 25 to 29 | 0.33 (0.23, 0.47) |
| Argentina | 30 to 34 | 0.47 (0.33, 0.66) |
| Argentina | 35 to 39 | 0.62 (0.42, 0.92) |
| Armenia | 15 to 19 | 0.3 (0.08, 1.1) |
| Armenia | 20 to 24 | 0.32 (0.09, 1.1) |
| Armenia | 25 to 29 | 0.35 (0.1, 1.19) |
| Armenia | 30 to 34 | 0.39 (0.11, 1.37) |
| Armenia | 35 to 39 | 0.41 (0.1, 1.8) |
| Australia | 15 to 19 | 1.97 (1.59, 2.43) |
| Australia | 20 to 24 | 1.88 (1.54, 2.29) |
| Australia | 25 to 29 | 3.79 (3.24, 4.45) |
| Australia | 30 to 34 | 6.18 (5.35, 7.13) |
| Australia | 35 to 39 | 6.75 (5.73, 7.95) |
| Austria | 15 to 19 | 1.02 (0.59, 1.75) |
| Austria | 20 to 24 | 1.08 (0.66, 1.76) |
| Austria | 25 to 29 | 1.2 (0.76, 1.91) |
| Austria | 30 to 34 | 1.74 (1.12, 2.69) |
| Austria | 35 to 39 | 2.47 (1.52, 4) |
| Azerbaijan | 15 to 19 | 0.26 (0.11, 0.61) |
| Azerbaijan | 20 to 24 | 0.21 (0.09, 0.5) |
| Azerbaijan | 25 to 29 | 0.3 (0.14, 0.65) |
| Azerbaijan | 30 to 34 | 0.29 (0.13, 0.65) |
| Azerbaijan | 35 to 39 | 0.33 (0.13, 0.86) |
| Bahrain | 15 to 19 | 0.49 (0.05, 5.21) |
| Bahrain | 20 to 24 | 0.87 (0.22, 3.41) |
| Bahrain | 25 to 29 | 0.91 (0.26, 3.12) |
| Bahrain | 30 to 34 | 1.78 (0.59, 5.3) |
| Bahrain | 35 to 39 | 2 (0.5, 7.92) |
| Bangladesh | 15 to 19 | 0.75 (0.66, 0.84) |
| Bangladesh | 20 to 24 | 0.65 (0.58, 0.74) |
| Bangladesh | 25 to 29 | 0.99 (0.89, 1.1) |
| Bangladesh | 30 to 34 | 1.56 (1.41, 1.73) |
| Bangladesh | 35 to 39 | 2.97 (2.65, 3.32) |
| Belarus | 15 to 19 | 0.22 (0.09, 0.53) |
| Belarus | 20 to 24 | 0.25 (0.11, 0.57) |
| Belarus | 25 to 29 | 0.36 (0.18, 0.75) |
| Belarus | 30 to 34 | 0.59 (0.29, 1.17) |
| Belarus | 35 to 39 | 0.91 (0.43, 1.92) |
| Belgium | 15 to 19 | 0.9 (0.55, 1.47) |
| Belgium | 20 to 24 | 1.2 (0.79, 1.83) |
| Belgium | 25 to 29 | 1.37 (0.92, 2.02) |
| Belgium | 30 to 34 | 2.05 (1.43, 2.94) |
| Belgium | 35 to 39 | 3.06 (2.05, 4.57) |
| Benin | 15 to 19 | 0.01 (0, 0.28) |
| Benin | 20 to 24 | 0.17 (0.05, 0.62) |
| Benin | 25 to 29 | 0.09 (0.02, 0.39) |
| Benin | 30 to 34 | 0.25 (0.08, 0.82) |
| Benin | 35 to 39 | 0.32 (0.08, 1.34) |
| Bhutan | 15 to 19 | 0.11 (0, 2.71) |
| Bhutan | 20 to 24 | 0.37 (0.03, 4.35) |
| Bhutan | 25 to 29 | 1.1 (0.24, 4.92) |
| Bhutan | 30 to 34 | 1.57 (0.33, 7.39) |
| Bhutan | 35 to 39 | 2.12 (0.36, 12.62) |
| Bolivia (Plurinational State of) | 15 to 19 | 0.12 (0.03, 0.4) |
| Bolivia (Plurinational State of) | 20 to 24 | 0.12 (0.04, 0.39) |
| Bolivia (Plurinational State of) | 25 to 29 | 0.13 (0.04, 0.41) |
| Bolivia (Plurinational State of) | 30 to 34 | 0.18 (0.06, 0.52) |
| Bolivia (Plurinational State of) | 35 to 39 | 0.31 (0.09, 1.04) |
| Bosnia and Herzegovina | 15 to 19 | 0.1 (0.01, 1.14) |
| Bosnia and Herzegovina | 20 to 24 | 0.23 (0.05, 1.05) |
| Bosnia and Herzegovina | 25 to 29 | 0.34 (0.09, 1.35) |
| Bosnia and Herzegovina | 30 to 34 | 0.71 (0.15, 3.25) |
| Bosnia and Herzegovina | 35 to 39 | 0.91 (0.17, 5.01) |
| Botswana | 15 to 19 | 0.45 (0.12, 1.65) |
| Botswana | 20 to 24 | 0.36 (0.09, 1.51) |
| Botswana | 25 to 29 | 0.43 (0.11, 1.61) |
| Botswana | 30 to 34 | 0.65 (0.17, 2.53) |
| Botswana | 35 to 39 | 0.99 (0.23, 4.27) |
| Brazil | 15 to 19 | 0.32 (0.27, 0.37) |
| Brazil | 20 to 24 | 0.35 (0.3, 0.4) |
| Brazil | 25 to 29 | 0.4 (0.34, 0.46) |
| Brazil | 30 to 34 | 0.59 (0.51, 0.68) |
| Brazil | 35 to 39 | 0.95 (0.81, 1.12) |
| Brunei Darussalam | 15 to 19 | 0.31 (0.02, 5.87) |
| Brunei Darussalam | 20 to 24 | 2.8 (0.81, 9.73) |
| Brunei Darussalam | 25 to 29 | 2.58 (0.75, 8.81) |
| Brunei Darussalam | 30 to 34 | 3.88 (1.29, 11.65) |
| Brunei Darussalam | 35 to 39 | 7.38 (2.14, 25.46) |
| Bulgaria | 15 to 19 | 0.56 (0.28, 1.11) |
| Bulgaria | 20 to 24 | 0.62 (0.32, 1.18) |
| Bulgaria | 25 to 29 | 0.89 (0.49, 1.6) |
| Bulgaria | 30 to 34 | 1.62 (0.96, 2.75) |
| Bulgaria | 35 to 39 | 2.93 (1.63, 5.26) |
| Burkina Faso | 15 to 19 | 0.07 (0.02, 0.23) |
| Burkina Faso | 20 to 24 | 0.14 (0.05, 0.37) |
| Burkina Faso | 25 to 29 | 0.11 (0.04, 0.32) |
| Burkina Faso | 30 to 34 | 0.27 (0.12, 0.61) |
| Burkina Faso | 35 to 39 | 0.47 (0.17, 1.29) |
| Burundi | 15 to 19 | 0.91 (0.58, 1.45) |
| Burundi | 20 to 24 | 0.79 (0.5, 1.24) |
| Burundi | 25 to 29 | 1.06 (0.69, 1.63) |
| Burundi | 30 to 34 | 1.53 (1.02, 2.27) |
| Burundi | 35 to 39 | 2.89 (1.83, 4.55) |
| Cambodia | 15 to 19 | 0.76 (0.54, 1.06) |
| Cambodia | 20 to 24 | 0.99 (0.73, 1.34) |
| Cambodia | 25 to 29 | 1.74 (1.34, 2.26) |
| Cambodia | 30 to 34 | 3.16 (2.5, 3.98) |
| Cambodia | 35 to 39 | 6.03 (4.59, 7.92) |
| Cameroon | 15 to 19 | 0.06 (0.02, 0.16) |
| Cameroon | 20 to 24 | 0.21 (0.11, 0.42) |
| Cameroon | 25 to 29 | 0.1 (0.05, 0.24) |
| Cameroon | 30 to 34 | 0.35 (0.19, 0.63) |
| Cameroon | 35 to 39 | 0.5 (0.23, 1.08) |
| Canada | 15 to 19 | 1.39 (1.12, 1.72) |
| Canada | 20 to 24 | 1.7 (1.4, 2.07) |
| Canada | 25 to 29 | 2.06 (1.71, 2.47) |
| Canada | 30 to 34 | 2.95 (2.5, 3.49) |
| Canada | 35 to 39 | 4.31 (3.57, 5.19) |
| Central African Republic | 15 to 19 | 0.22 (0.05, 0.9) |
| Central African Republic | 20 to 24 | 0.24 (0.07, 0.85) |
| Central African Republic | 25 to 29 | 0.28 (0.08, 0.97) |
| Central African Republic | 30 to 34 | 0.32 (0.09, 1.16) |
| Central African Republic | 35 to 39 | 0.55 (0.14, 2.21) |
| Chad | 15 to 19 | 0.01 (0, 0.19) |
| Chad | 20 to 24 | 0.14 (0.04, 0.5) |
| Chad | 25 to 29 | 0.08 (0.02, 0.34) |
| Chad | 30 to 34 | 0.22 (0.07, 0.69) |
| Chad | 35 to 39 | 0.34 (0.09, 1.31) |
| Chile | 15 to 19 | 0.17 (0.07, 0.37) |
| Chile | 20 to 24 | 0.19 (0.09, 0.4) |
| Chile | 25 to 29 | 0.22 (0.11, 0.45) |
| Chile | 30 to 34 | 0.34 (0.18, 0.66) |
| Chile | 35 to 39 | 0.48 (0.23, 1.03) |
| China | 15 to 19 | 3.01 (2.61, 3.46) |
| China | 20 to 24 | 4.47 (3.97, 5.03) |
| China | 25 to 29 | 8.5 (7.73, 9.35) |
| China | 30 to 34 | 19.71 (18.24, 21.29) |
| China | 35 to 39 | 33.19 (30.25, 36.42) |
| Colombia | 15 to 19 | 0.32 (0.22, 0.45) |
| Colombia | 20 to 24 | 0.37 (0.27, 0.5) |
| Colombia | 25 to 29 | 0.44 (0.32, 0.6) |
| Colombia | 30 to 34 | 0.53 (0.39, 0.72) |
| Colombia | 35 to 39 | 0.65 (0.46, 0.93) |
| Comoros | 15 to 19 | 0.13 (0.01, 2.85) |
| Comoros | 20 to 24 | 0.29 (0.02, 5.22) |
| Comoros | 25 to 29 | 1.09 (0.18, 6.56) |
| Comoros | 30 to 34 | 2.89 (0.52, 16.15) |
| Comoros | 35 to 39 | 3.3 (0.45, 24.09) |
| Congo | 15 to 19 | 0.19 (0.02, 1.45) |
| Congo | 20 to 24 | 0.27 (0.07, 1) |
| Congo | 25 to 29 | 0.31 (0.09, 1.1) |
| Congo | 30 to 34 | 0.41 (0.13, 1.31) |
| Congo | 35 to 39 | 0.76 (0.2, 2.87) |
| Costa Rica | 15 to 19 | 0.58 (0.27, 1.26) |
| Costa Rica | 20 to 24 | 0.6 (0.28, 1.31) |
| Costa Rica | 25 to 29 | 0.73 (0.36, 1.51) |
| Costa Rica | 30 to 34 | 1.03 (0.52, 2.07) |
| Costa Rica | 35 to 39 | 1.33 (0.6, 2.96) |
| Coted'Ivoire | 15 to 19 | 0.15 (0.08, 0.29) |
| Coted'Ivoire | 20 to 24 | 0.27 (0.15, 0.47) |
| Coted'Ivoire | 25 to 29 | 0.16 (0.09, 0.3) |
| Coted'Ivoire | 30 to 34 | 0.53 (0.32, 0.86) |
| Coted'Ivoire | 35 to 39 | 0.71 (0.39, 1.29) |
| Croatia | 15 to 19 | 0.33 (0.1, 1.05) |
| Croatia | 20 to 24 | 0.34 (0.11, 1.03) |
| Croatia | 25 to 29 | 0.59 (0.23, 1.52) |
| Croatia | 30 to 34 | 0.95 (0.37, 2.43) |
| Croatia | 35 to 39 | 1.52 (0.55, 4.19) |
| Cuba | 15 to 19 | 0.49 (0.27, 0.88) |
| Cuba | 20 to 24 | 0.51 (0.29, 0.89) |
| Cuba | 25 to 29 | 0.74 (0.45, 1.22) |
| Cuba | 30 to 34 | 1.05 (0.65, 1.68) |
| Cuba | 35 to 39 | 1.26 (0.74, 2.16) |
| Cyprus | 15 to 19 | 0.11 (0.01, 2.18) |
| Cyprus | 20 to 24 | 0.69 (0.13, 3.63) |
| Cyprus | 25 to 29 | 0.92 (0.22, 3.78) |
| Cyprus | 30 to 34 | 1.37 (0.29, 6.48) |
| Cyprus | 35 to 39 | 2.44 (0.39, 15.28) |
| Czechia | 15 to 19 | 0.78 (0.48, 1.27) |
| Czechia | 20 to 24 | 0.98 (0.64, 1.51) |
| Czechia | 25 to 29 | 1.31 (0.87, 1.96) |
| Czechia | 30 to 34 | 2.3 (1.57, 3.36) |
| Czechia | 35 to 39 | 3.77 (2.48, 5.75) |
| Democratic People's Republic of Korea | 15 to 19 | 1.22 (0.98, 1.53) |
| Democratic People's Republic of Korea | 20 to 24 | 1.56 (1.28, 1.9) |
| Democratic People's Republic of Korea | 25 to 29 | 3.25 (2.78, 3.81) |
| Democratic People's Republic of Korea | 30 to 34 | 6.29 (5.48, 7.22) |
| Democratic People's Republic of Korea | 35 to 39 | 9.6 (8.2, 11.24) |
| Democratic Republic of the Congo | 15 to 19 | 0.11 (0.07, 0.17) |
| Democratic Republic of the Congo | 20 to 24 | 0.13 (0.09, 0.21) |
| Democratic Republic of the Congo | 25 to 29 | 0.17 (0.11, 0.26) |
| Democratic Republic of the Congo | 30 to 34 | 0.27 (0.18, 0.39) |
| Democratic Republic of the Congo | 35 to 39 | 0.54 (0.35, 0.83) |
| Denmark | 15 to 19 | 0.46 (0.18, 1.17) |
| Denmark | 20 to 24 | 0.61 (0.27, 1.38) |
| Denmark | 25 to 29 | 0.7 (0.31, 1.54) |
| Denmark | 30 to 34 | 1.27 (0.62, 2.61) |
| Denmark | 35 to 39 | 1.93 (0.88, 4.25) |
| Djibouti | 15 to 19 | 0.79 (0.15, 4.19) |
| Djibouti | 20 to 24 | 0.78 (0.18, 3.28) |
| Djibouti | 25 to 29 | 1.21 (0.33, 4.38) |
| Djibouti | 30 to 34 | 2.47 (0.68, 8.96) |
| Djibouti | 35 to 39 | 4.42 (0.97, 20.12) |
| Dominican Republic | 15 to 19 | 0.32 (0.16, 0.64) |
| Dominican Republic | 20 to 24 | 0.4 (0.22, 0.73) |
| Dominican Republic | 25 to 29 | 0.65 (0.37, 1.12) |
| Dominican Republic | 30 to 34 | 0.83 (0.47, 1.46) |
| Dominican Republic | 35 to 39 | 1.09 (0.57, 2.07) |
| Ecuador | 15 to 19 | 0.17 (0.08, 0.4) |
| Ecuador | 20 to 24 | 0.15 (0.07, 0.34) |
| Ecuador | 25 to 29 | 0.21 (0.1, 0.45) |
| Ecuador | 30 to 34 | 0.29 (0.13, 0.63) |
| Ecuador | 35 to 39 | 0.44 (0.18, 1.04) |
| Egypt | 15 to 19 | 0.06 (0.04, 0.11) |
| Egypt | 20 to 24 | 0.06 (0.04, 0.1) |
| Egypt | 25 to 29 | 0.09 (0.05, 0.14) |
| Egypt | 30 to 34 | 0.14 (0.09, 0.22) |
| Egypt | 35 to 39 | 0.2 (0.12, 0.33) |
| El Salvador | 15 to 19 | 0.2 (0.06, 0.62) |
| El Salvador | 20 to 24 | 0.23 (0.08, 0.66) |
| El Salvador | 25 to 29 | 0.31 (0.12, 0.82) |
| El Salvador | 30 to 34 | 0.52 (0.2, 1.4) |
| El Salvador | 35 to 39 | 0.87 (0.28, 2.63) |
| Equatorial Guinea | 15 to 19 | 0.11 (0, 6.55) |
| Equatorial Guinea | 20 to 24 | 0.1 (0, 4.87) |
| Equatorial Guinea | 25 to 29 | 0.1 (0, 4.87) |
| Equatorial Guinea | 30 to 34 | 0.11 (0, 5.98) |
| Equatorial Guinea | 35 to 39 | 0.26 (0, 16.75) |
| Eritrea | 15 to 19 | 0.76 (0.41, 1.42) |
| Eritrea | 20 to 24 | 0.7 (0.38, 1.28) |
| Eritrea | 25 to 29 | 1.05 (0.6, 1.84) |
| Eritrea | 30 to 34 | 1.76 (1.04, 2.97) |
| Eritrea | 35 to 39 | 4.05 (2.26, 7.27) |
| Estonia | 15 to 19 | 0.21 (0.01, 5.13) |
| Estonia | 20 to 24 | 0.75 (0.15, 3.67) |
| Estonia | 25 to 29 | 0.83 (0.18, 3.88) |
| Estonia | 30 to 34 | 1.21 (0.3, 4.87) |
| Estonia | 35 to 39 | 1.57 (0.3, 8.24) |
| Eswatini | 15 to 19 | 0.6 (0.06, 6.61) |
| Eswatini | 20 to 24 | 0.2 (0.01, 5.28) |
| Eswatini | 25 to 29 | 0.23 (0.01, 5.58) |
| Eswatini | 30 to 34 | 1.37 (0.21, 9.12) |
| Eswatini | 35 to 39 | 4.5 (0.33, 61.71) |
| Ethiopia | 15 to 19 | 0.76 (0.65, 0.89) |
| Ethiopia | 20 to 24 | 0.73 (0.63, 0.86) |
| Ethiopia | 25 to 29 | 1.04 (0.89, 1.2) |
| Ethiopia | 30 to 34 | 1.52 (1.33, 1.74) |
| Ethiopia | 35 to 39 | 2.78 (2.38, 3.25) |
| Finland | 15 to 19 | 0.3 (0.1, 0.91) |
| Finland | 20 to 24 | 0.52 (0.21, 1.28) |
| Finland | 25 to 29 | 0.62 (0.26, 1.47) |
| Finland | 30 to 34 | 0.92 (0.41, 2.08) |
| Finland | 35 to 39 | 1.39 (0.56, 3.42) |
| France | 15 to 19 | 1.66 (1.44, 1.91) |
| France | 20 to 24 | 2.35 (2.08, 2.65) |
| France | 25 to 29 | 3.05 (2.73, 3.42) |
| France | 30 to 34 | 4.54 (4.08, 5.05) |
| France | 35 to 39 | 6.78 (6.01, 7.63) |
| Gabon | 15 to 19 | 0.19 (0, 7.23) |
| Gabon | 20 to 24 | 0.19 (0.01, 6.05) |
| Gabon | 25 to 29 | 0.2 (0.01, 6.04) |
| Gabon | 30 to 34 | 0.47 (0.05, 4.13) |
| Gabon | 35 to 39 | 3.02 (0.17, 52.66) |
| Georgia | 15 to 19 | 0.43 (0.15, 1.23) |
| Georgia | 20 to 24 | 0.48 (0.19, 1.22) |
| Georgia | 25 to 29 | 0.75 (0.31, 1.81) |
| Georgia | 30 to 34 | 0.78 (0.32, 1.93) |
| Georgia | 35 to 39 | 1.03 (0.38, 2.79) |
| Germany | 15 to 19 | 0.66 (0.53, 0.81) |
| Germany | 20 to 24 | 0.7 (0.57, 0.85) |
| Germany | 25 to 29 | 0.76 (0.63, 0.91) |
| Germany | 30 to 34 | 1.16 (0.97, 1.38) |
| Germany | 35 to 39 | 1.65 (1.37, 2) |
| Ghana | 15 to 19 | 0 (0, 0.11) |
| Ghana | 20 to 24 | 0.04 (0, 0.41) |
| Ghana | 25 to 29 | 0 (0, 0.06) |
| Ghana | 30 to 34 | 0.03 (0, 0.19) |
| Ghana | 35 to 39 | 0.02 (0, 0.2) |
| Greece | 15 to 19 | 1.29 (0.88, 1.89) |
| Greece | 20 to 24 | 1.86 (1.34, 2.58) |
| Greece | 25 to 29 | 2.05 (1.48, 2.84) |
| Greece | 30 to 34 | 2.83 (2.06, 3.88) |
| Greece | 35 to 39 | 3.82 (2.72, 5.36) |
| Greenland | 15 to 19 | 2.13 (0.04, 121.77) |
| Greenland | 20 to 24 | 2.06 (0.04, 97.59) |
| Greenland | 25 to 29 | 1.7 (0.04, 76.56) |
| Greenland | 30 to 34 | 5.57 (0.14, 224.84) |
| Greenland | 35 to 39 | 16.31 (0.44, 609.56) |
| Guam | 15 to 19 | 0.77 (0.01, 44.14) |
| Guam | 20 to 24 | 0.82 (0.02, 39.11) |
| Guam | 25 to 29 | 0.84 (0.02, 38.95) |
| Guam | 30 to 34 | 0.9 (0.02, 46.16) |
| Guam | 35 to 39 | 10.66 (0.26, 430.67) |
| Guatemala | 15 to 19 | 0.19 (0.09, 0.4) |
| Guatemala | 20 to 24 | 0.22 (0.1, 0.44) |
| Guatemala | 25 to 29 | 0.28 (0.14, 0.56) |
| Guatemala | 30 to 34 | 0.42 (0.21, 0.83) |
| Guatemala | 35 to 39 | 0.56 (0.26, 1.23) |
| Guinea | 15 to 19 | 0.11 (0.04, 0.35) |
| Guinea | 20 to 24 | 0.22 (0.09, 0.56) |
| Guinea | 25 to 29 | 0.16 (0.06, 0.47) |
| Guinea | 30 to 34 | 0.37 (0.15, 0.89) |
| Guinea | 35 to 39 | 0.47 (0.16, 1.35) |
| Guinea-Bissau | 15 to 19 | 0.12 (0, 4.94) |
| Guinea-Bissau | 20 to 24 | 0.12 (0, 4.01) |
| Guinea-Bissau | 25 to 29 | 0.14 (0, 4.36) |
| Guinea-Bissau | 30 to 34 | 0.3 (0.03, 2.88) |
| Guinea-Bissau | 35 to 39 | 0.48 (0.01, 18.64) |
| Haiti | 15 to 19 | 0.27 (0.13, 0.55) |
| Haiti | 20 to 24 | 0.31 (0.16, 0.62) |
| Haiti | 25 to 29 | 0.5 (0.27, 0.9) |
| Haiti | 30 to 34 | 0.58 (0.32, 1.05) |
| Haiti | 35 to 39 | 0.87 (0.44, 1.76) |
| Honduras | 15 to 19 | 0.12 (0.03, 0.45) |
| Honduras | 20 to 24 | 0.13 (0.04, 0.44) |
| Honduras | 25 to 29 | 0.13 (0.04, 0.45) |
| Honduras | 30 to 34 | 0.15 (0.04, 0.52) |
| Honduras | 35 to 39 | 0.27 (0.07, 1) |
| Hungary | 15 to 19 | 0.45 (0.23, 0.86) |
| Hungary | 20 to 24 | 0.57 (0.32, 1.03) |
| Hungary | 25 to 29 | 0.87 (0.51, 1.49) |
| Hungary | 30 to 34 | 1.43 (0.86, 2.36) |
| Hungary | 35 to 39 | 2.58 (1.5, 4.46) |
| Iceland | 15 to 19 | 0.59 (0.02, 19.19) |
| Iceland | 20 to 24 | 0.65 (0.02, 19.01) |
| Iceland | 25 to 29 | 0.68 (0.02, 18.81) |
| Iceland | 30 to 34 | 4.44 (0.62, 31.53) |
| Iceland | 35 to 39 | 7.72 (0.54, 109.83) |
| India | 15 to 19 | 0.62 (0.59, 0.64) |
| India | 20 to 24 | 0.65 (0.62, 0.68) |
| India | 25 to 29 | 0.97 (0.94, 1.01) |
| India | 30 to 34 | 1.64 (1.58, 1.7) |
| India | 35 to 39 | 3 (2.89, 3.12) |
| Indonesia | 15 to 19 | 0.76 (0.7, 0.84) |
| Indonesia | 20 to 24 | 0.91 (0.84, 0.98) |
| Indonesia | 25 to 29 | 1.5 (1.4, 1.61) |
| Indonesia | 30 to 34 | 2.6 (2.44, 2.77) |
| Indonesia | 35 to 39 | 4.54 (4.23, 4.87) |
| Iran (Islamic Republic of) | 15 to 19 | 0.24 (0.19, 0.32) |
| Iran (Islamic Republic of) | 20 to 24 | 0.24 (0.19, 0.31) |
| Iran (Islamic Republic of) | 25 to 29 | 0.36 (0.28, 0.45) |
| Iran (Islamic Republic of) | 30 to 34 | 0.54 (0.43, 0.67) |
| Iran (Islamic Republic of) | 35 to 39 | 0.73 (0.56, 0.95) |
| Iraq | 15 to 19 | 0.29 (0.2, 0.44) |
| Iraq | 20 to 24 | 0.36 (0.25, 0.51) |
| Iraq | 25 to 29 | 0.47 (0.33, 0.65) |
| Iraq | 30 to 34 | 0.83 (0.62, 1.13) |
| Iraq | 35 to 39 | 1.35 (0.95, 1.9) |
| Ireland | 15 to 19 | 0.9 (0.45, 1.8) |
| Ireland | 20 to 24 | 1.11 (0.6, 2.06) |
| Ireland | 25 to 29 | 1.47 (0.83, 2.59) |
| Ireland | 30 to 34 | 2.47 (1.44, 4.24) |
| Ireland | 35 to 39 | 3.55 (1.93, 6.52) |
| Israel | 15 to 19 | 0.65 (0.36, 1.18) |
| Israel | 20 to 24 | 0.89 (0.52, 1.5) |
| Israel | 25 to 29 | 0.99 (0.6, 1.65) |
| Israel | 30 to 34 | 1.48 (0.92, 2.38) |
| Israel | 35 to 39 | 2.16 (1.26, 3.71) |
| Italy | 15 to 19 | 1.37 (1.14, 1.64) |
| Italy | 20 to 24 | 1.66 (1.41, 1.94) |
| Italy | 25 to 29 | 1.65 (1.41, 1.92) |
| Italy | 30 to 34 | 2.89 (2.5, 3.33) |
| Italy | 35 to 39 | 3.87 (3.31, 4.51) |
| Jamaica | 15 to 19 | 0.39 (0.1, 1.49) |
| Jamaica | 20 to 24 | 0.45 (0.14, 1.47) |
| Jamaica | 25 to 29 | 0.52 (0.16, 1.68) |
| Jamaica | 30 to 34 | 0.77 (0.27, 2.24) |
| Jamaica | 35 to 39 | 1.14 (0.33, 3.98) |
| Japan | 15 to 19 | 0.5 (0.42, 0.6) |
| Japan | 20 to 24 | 0.85 (0.73, 0.99) |
| Japan | 25 to 29 | 0.97 (0.83, 1.12) |
| Japan | 30 to 34 | 1.31 (1.13, 1.52) |
| Japan | 35 to 39 | 1.81 (1.54, 2.13) |
| Jordan | 15 to 19 | 0.68 (0.4, 1.17) |
| Jordan | 20 to 24 | 0.85 (0.53, 1.35) |
| Jordan | 25 to 29 | 1.2 (0.79, 1.83) |
| Jordan | 30 to 34 | 2.08 (1.42, 3.03) |
| Jordan | 35 to 39 | 2.93 (1.85, 4.64) |
| Kazakhstan | 15 to 19 | 0.49 (0.32, 0.75) |
| Kazakhstan | 20 to 24 | 0.57 (0.38, 0.84) |
| Kazakhstan | 25 to 29 | 0.88 (0.62, 1.24) |
| Kazakhstan | 30 to 34 | 1.01 (0.71, 1.43) |
| Kazakhstan | 35 to 39 | 1.34 (0.89, 2.02) |
| Kenya | 15 to 19 | 0.89 (0.73, 1.09) |
| Kenya | 20 to 24 | 0.96 (0.79, 1.16) |
| Kenya | 25 to 29 | 1.52 (1.28, 1.8) |
| Kenya | 30 to 34 | 2.61 (2.22, 3.06) |
| Kenya | 35 to 39 | 5.16 (4.3, 6.19) |
| Kuwait | 15 to 19 | 0.66 (0.24, 1.84) |
| Kuwait | 20 to 24 | 0.83 (0.38, 1.83) |
| Kuwait | 25 to 29 | 0.9 (0.46, 1.74) |
| Kuwait | 30 to 34 | 1.37 (0.77, 2.44) |
| Kuwait | 35 to 39 | 1.35 (0.66, 2.75) |
| Kyrgyzstan | 15 to 19 | 0.35 (0.15, 0.82) |
| Kyrgyzstan | 20 to 24 | 0.42 (0.19, 0.91) |
| Kyrgyzstan | 25 to 29 | 0.72 (0.37, 1.42) |
| Kyrgyzstan | 30 to 34 | 0.98 (0.52, 1.87) |
| Kyrgyzstan | 35 to 39 | 1.58 (0.72, 3.44) |
| Lao People's Democratic Republic | 15 to 19 | 0.68 (0.39, 1.18) |
| Lao People's Democratic Republic | 20 to 24 | 0.81 (0.49, 1.35) |
| Lao People's Democratic Republic | 25 to 29 | 1.35 (0.86, 2.11) |
| Lao People's Democratic Republic | 30 to 34 | 2.38 (1.59, 3.57) |
| Lao People's Democratic Republic | 35 to 39 | 4.36 (2.75, 6.93) |
| Latvia | 15 to 19 | 0.45 (0.09, 2.26) |
| Latvia | 20 to 24 | 0.39 (0.05, 2.76) |
| Latvia | 25 to 29 | 0.4 (0.06, 2.52) |
| Latvia | 30 to 34 | 0.53 (0.11, 2.5) |
| Latvia | 35 to 39 | 0.7 (0.12, 4.03) |
| Lebanon | 15 to 19 | 0.36 (0.14, 0.94) |
| Lebanon | 20 to 24 | 0.47 (0.21, 1.05) |
| Lebanon | 25 to 29 | 0.76 (0.38, 1.5) |
| Lebanon | 30 to 34 | 1.62 (0.88, 2.95) |
| Lebanon | 35 to 39 | 2.35 (1.11, 4.99) |
| Lesotho | 15 to 19 | 0.22 (0.04, 1.39) |
| Lesotho | 20 to 24 | 0.34 (0.08, 1.49) |
| Lesotho | 25 to 29 | 0.68 (0.17, 2.76) |
| Lesotho | 30 to 34 | 1.35 (0.28, 6.54) |
| Lesotho | 35 to 39 | 3.74 (0.51, 27.2) |
| Liberia | 15 to 19 | 0.03 (0, 0.68) |
| Liberia | 20 to 24 | 0.17 (0.03, 1.08) |
| Liberia | 25 to 29 | 0.05 (0, 1.07) |
| Liberia | 30 to 34 | 0.31 (0.05, 1.89) |
| Liberia | 35 to 39 | 0.4 (0.04, 4.28) |
| Libya | 15 to 19 | 2.54 (1.89, 3.4) |
| Libya | 20 to 24 | 2.82 (2.16, 3.68) |
| Libya | 25 to 29 | 4.48 (3.54, 5.68) |
| Libya | 30 to 34 | 9.52 (7.69, 11.77) |
| Libya | 35 to 39 | 16.02 (12.49, 20.53) |
| Lithuania | 15 to 19 | 0.4 (0.11, 1.5) |
| Lithuania | 20 to 24 | 0.38 (0.11, 1.32) |
| Lithuania | 25 to 29 | 0.4 (0.12, 1.4) |
| Lithuania | 30 to 34 | 0.6 (0.18, 2) |
| Lithuania | 35 to 39 | 0.85 (0.23, 3.2) |
| Luxembourg | 15 to 19 | 0.4 (0.01, 12.07) |
| Luxembourg | 20 to 24 | 0.34 (0.01, 8.33) |
| Luxembourg | 25 to 29 | 1.82 (0.19, 17.46) |
| Luxembourg | 30 to 34 | 1.79 (0.27, 12) |
| Luxembourg | 35 to 39 | 1.82 (0.2, 16.25) |
| Madagascar | 15 to 19 | 0.65 (0.46, 0.92) |
| Madagascar | 20 to 24 | 0.66 (0.48, 0.92) |
| Madagascar | 25 to 29 | 0.96 (0.71, 1.3) |
| Madagascar | 30 to 34 | 1.43 (1.07, 1.9) |
| Madagascar | 35 to 39 | 2.53 (1.82, 3.51) |
| Malawi | 15 to 19 | 0.17 (0.08, 0.38) |
| Malawi | 20 to 24 | 0.16 (0.07, 0.36) |
| Malawi | 25 to 29 | 0.24 (0.11, 0.48) |
| Malawi | 30 to 34 | 0.39 (0.2, 0.77) |
| Malawi | 35 to 39 | 0.76 (0.36, 1.64) |
| Malaysia | 15 to 19 | 3.47 (3.08, 3.9) |
| Malaysia | 20 to 24 | 4.35 (3.92, 4.83) |
| Malaysia | 25 to 29 | 7.63 (6.98, 8.34) |
| Malaysia | 30 to 34 | 16.83 (15.65, 18.11) |
| Malaysia | 35 to 39 | 30.75 (28.17, 33.57) |
| Maldives | 15 to 19 | 0.44 (0.01, 17.63) |
| Maldives | 20 to 24 | 0.42 (0.01, 14.12) |
| Maldives | 25 to 29 | 0.43 (0.01, 13.45) |
| Maldives | 30 to 34 | 0.89 (0.1, 7.96) |
| Maldives | 35 to 39 | 2.03 (0.08, 51.75) |
| Mali | 15 to 19 | 0.08 (0.02, 0.24) |
| Mali | 20 to 24 | 0.12 (0.04, 0.36) |
| Mali | 25 to 29 | 0.09 (0.03, 0.29) |
| Mali | 30 to 34 | 0.19 (0.07, 0.47) |
| Mali | 35 to 39 | 0.25 (0.08, 0.79) |
| Malta | 15 to 19 | 3.26 (0.97, 10.98) |
| Malta | 20 to 24 | 3.29 (1.03, 10.51) |
| Malta | 25 to 29 | 4.03 (1.3, 12.46) |
| Malta | 30 to 34 | 5.89 (2.12, 16.39) |
| Malta | 35 to 39 | 9.23 (2.92, 29.16) |
| Mauritania | 15 to 19 | 0.03 (0, 1.09) |
| Mauritania | 20 to 24 | 0.07 (0, 2.26) |
| Mauritania | 25 to 29 | 0.06 (0, 1.92) |
| Mauritania | 30 to 34 | 0.26 (0.03, 1.95) |
| Mauritania | 35 to 39 | 0.33 (0.02, 7.24) |
| Mauritius | 15 to 19 | 0.51 (0.08, 3.34) |
| Mauritius | 20 to 24 | 0.95 (0.3, 3.08) |
| Mauritius | 25 to 29 | 1.24 (0.39, 3.94) |
| Mauritius | 30 to 34 | 3.17 (1.21, 8.27) |
| Mauritius | 35 to 39 | 5.64 (1.89, 16.86) |
| Mexico | 15 to 19 | 0.12 (0.08, 0.16) |
| Mexico | 20 to 24 | 0.14 (0.1, 0.19) |
| Mexico | 25 to 29 | 0.19 (0.14, 0.25) |
| Mexico | 30 to 34 | 0.28 (0.21, 0.37) |
| Mexico | 35 to 39 | 0.43 (0.31, 0.59) |
| Mongolia | 15 to 19 | 0.4 (0.12, 1.31) |
| Mongolia | 20 to 24 | 0.41 (0.13, 1.27) |
| Mongolia | 25 to 29 | 0.58 (0.21, 1.57) |
| Mongolia | 30 to 34 | 0.68 (0.25, 1.86) |
| Mongolia | 35 to 39 | 0.93 (0.29, 3.05) |
| Morocco | 15 to 19 | 0.67 (0.52, 0.87) |
| Morocco | 20 to 24 | 0.65 (0.51, 0.84) |
| Morocco | 25 to 29 | 0.93 (0.74, 1.17) |
| Morocco | 30 to 34 | 1.63 (1.32, 2.02) |
| Morocco | 35 to 39 | 2.87 (2.26, 3.63) |
| Mozambique | 15 to 19 | 0 (0, 0.08) |
| Mozambique | 20 to 24 | 0.01 (0, 0.15) |
| Mozambique | 25 to 29 | 0.05 (0.01, 0.23) |
| Mozambique | 30 to 34 | 0.08 (0.01, 0.43) |
| Mozambique | 35 to 39 | 0.18 (0.02, 1.32) |
| Myanmar | 15 to 19 | 0.76 (0.63, 0.92) |
| Myanmar | 20 to 24 | 0.9 (0.75, 1.08) |
| Myanmar | 25 to 29 | 1.48 (1.27, 1.73) |
| Myanmar | 30 to 34 | 2.26 (1.95, 2.61) |
| Myanmar | 35 to 39 | 3.77 (3.2, 4.43) |
| Namibia | 15 to 19 | 0.45 (0.12, 1.62) |
| Namibia | 20 to 24 | 0.47 (0.14, 1.6) |
| Namibia | 25 to 29 | 0.51 (0.15, 1.71) |
| Namibia | 30 to 34 | 0.55 (0.16, 1.92) |
| Namibia | 35 to 39 | 1.2 (0.32, 4.47) |
| Nepal | 15 to 19 | 0.62 (0.46, 0.83) |
| Nepal | 20 to 24 | 0.67 (0.5, 0.89) |
| Nepal | 25 to 29 | 0.97 (0.75, 1.27) |
| Nepal | 30 to 34 | 1.57 (1.23, 2) |
| Nepal | 35 to 39 | 2.89 (2.19, 3.81) |
| Netherlands | 15 to 19 | 1.41 (1.03, 1.94) |
| Netherlands | 20 to 24 | 1.35 (1, 1.82) |
| Netherlands | 25 to 29 | 2.15 (1.66, 2.79) |
| Netherlands | 30 to 34 | 3.16 (2.47, 4.04) |
| Netherlands | 35 to 39 | 4.55 (3.47, 5.97) |
| New Zealand | 15 to 19 | 0.98 (0.52, 1.84) |
| New Zealand | 20 to 24 | 1.25 (0.73, 2.15) |
| New Zealand | 25 to 29 | 2.21 (1.4, 3.5) |
| New Zealand | 30 to 34 | 4.13 (2.74, 6.23) |
| New Zealand | 35 to 39 | 4.12 (2.58, 6.58) |
| Nicaragua | 15 to 19 | 0.18 (0.05, 0.62) |
| Nicaragua | 20 to 24 | 0.19 (0.06, 0.62) |
| Nicaragua | 25 to 29 | 0.21 (0.06, 0.67) |
| Nicaragua | 30 to 34 | 0.32 (0.11, 0.91) |
| Nicaragua | 35 to 39 | 0.52 (0.16, 1.75) |
| Niger | 15 to 19 | 0.03 (0, 0.24) |
| Niger | 20 to 24 | 0.11 (0.03, 0.35) |
| Niger | 25 to 29 | 0.08 (0.02, 0.29) |
| Niger | 30 to 34 | 0.22 (0.08, 0.62) |
| Niger | 35 to 39 | 0.31 (0.09, 1.1) |
| Nigeria | 15 to 19 | 0.29 (0.25, 0.35) |
| Nigeria | 20 to 24 | 0.63 (0.55, 0.72) |
| Nigeria | 25 to 29 | 0.57 (0.5, 0.66) |
| Nigeria | 30 to 34 | 1.33 (1.19, 1.5) |
| Nigeria | 35 to 39 | 1.94 (1.69, 2.22) |
| North Macedonia | 15 to 19 | 0.38 (0.07, 2.07) |
| North Macedonia | 20 to 24 | 0.41 (0.1, 1.71) |
| North Macedonia | 25 to 29 | 0.65 (0.18, 2.34) |
| North Macedonia | 30 to 34 | 0.85 (0.2, 3.53) |
| North Macedonia | 35 to 39 | 1.56 (0.35, 7.05) |
| Norway | 15 to 19 | 0.58 (0.24, 1.38) |
| Norway | 20 to 24 | 0.71 (0.32, 1.59) |
| Norway | 25 to 29 | 1.07 (0.53, 2.18) |
| Norway | 30 to 34 | 0.72 (0.32, 1.62) |
| Norway | 35 to 39 | 0.75 (0.3, 1.86) |
| Oman | 15 to 19 | 0.4 (0.14, 1.18) |
| Oman | 20 to 24 | 0.47 (0.18, 1.27) |
| Oman | 25 to 29 | 0.59 (0.26, 1.31) |
| Oman | 30 to 34 | 0.89 (0.44, 1.81) |
| Oman | 35 to 39 | 1.29 (0.51, 3.25) |
| Pakistan | 15 to 19 | 0.88 (0.8, 0.97) |
| Pakistan | 20 to 24 | 0.93 (0.85, 1.02) |
| Pakistan | 25 to 29 | 1.36 (1.25, 1.48) |
| Pakistan | 30 to 34 | 2.19 (2.02, 2.37) |
| Pakistan | 35 to 39 | 4.17 (3.81, 4.57) |
| Palestine | 15 to 19 | 0.28 (0.08, 0.95) |
| Palestine | 20 to 24 | 0.28 (0.09, 0.91) |
| Palestine | 25 to 29 | 0.35 (0.11, 1.11) |
| Palestine | 30 to 34 | 0.47 (0.16, 1.33) |
| Palestine | 35 to 39 | 0.52 (0.15, 1.85) |
| Panama | 15 to 19 | 0.38 (0.12, 1.23) |
| Panama | 20 to 24 | 0.38 (0.13, 1.17) |
| Panama | 25 to 29 | 0.54 (0.2, 1.45) |
| Panama | 30 to 34 | 0.76 (0.29, 2.04) |
| Panama | 35 to 39 | 0.88 (0.28, 2.8) |
| Papua New Guinea | 15 to 19 | 0.02 (0, 0.27) |
| Papua New Guinea | 20 to 24 | 0.48 (0.22, 1.05) |
| Papua New Guinea | 25 to 29 | 0.26 (0.11, 0.63) |
| Papua New Guinea | 30 to 34 | 0.67 (0.32, 1.37) |
| Papua New Guinea | 35 to 39 | 1.57 (0.69, 3.56) |
| Paraguay | 15 to 19 | 0.2 (0.06, 0.66) |
| Paraguay | 20 to 24 | 0.18 (0.06, 0.57) |
| Paraguay | 25 to 29 | 0.17 (0.05, 0.58) |
| Paraguay | 30 to 34 | 0.31 (0.11, 0.91) |
| Paraguay | 35 to 39 | 0.39 (0.11, 1.37) |
| Peru | 15 to 19 | 0.11 (0.05, 0.22) |
| Peru | 20 to 24 | 0.12 (0.06, 0.24) |
| Peru | 25 to 29 | 0.15 (0.08, 0.28) |
| Peru | 30 to 34 | 0.25 (0.14, 0.44) |
| Peru | 35 to 39 | 0.4 (0.2, 0.78) |
| Philippines | 15 to 19 | 0.74 (0.65, 0.85) |
| Philippines | 20 to 24 | 1.02 (0.91, 1.15) |
| Philippines | 25 to 29 | 1.93 (1.75, 2.14) |
| Philippines | 30 to 34 | 3.58 (3.28, 3.92) |
| Philippines | 35 to 39 | 6.08 (5.49, 6.73) |
| Poland | 15 to 19 | 0.3 (0.2, 0.43) |
| Poland | 20 to 24 | 0.43 (0.31, 0.59) |
| Poland | 25 to 29 | 0.64 (0.48, 0.86) |
| Poland | 30 to 34 | 1.13 (0.86, 1.49) |
| Poland | 35 to 39 | 2.14 (1.58, 2.9) |
| Portugal | 15 to 19 | 0.89 (0.55, 1.44) |
| Portugal | 20 to 24 | 1.2 (0.79, 1.82) |
| Portugal | 25 to 29 | 1.44 (0.97, 2.14) |
| Portugal | 30 to 34 | 2.17 (1.49, 3.17) |
| Portugal | 35 to 39 | 3.41 (2.27, 5.12) |
| Puerto Rico | 15 to 19 | 0.66 (0.29, 1.5) |
| Puerto Rico | 20 to 24 | 1.03 (0.5, 2.11) |
| Puerto Rico | 25 to 29 | 1.4 (0.69, 2.81) |
| Puerto Rico | 30 to 34 | 1.87 (0.95, 3.68) |
| Puerto Rico | 35 to 39 | 2.05 (0.93, 4.53) |
| Qatar | 15 to 19 | 0.73 (0.11, 4.73) |
| Qatar | 20 to 24 | 0.53 (0.14, 1.97) |
| Qatar | 25 to 29 | 0.88 (0.4, 1.95) |
| Qatar | 30 to 34 | 1.43 (0.58, 3.55) |
| Qatar | 35 to 39 | 1.89 (0.52, 6.8) |
| Republic of Korea | 15 to 19 | 0.31 (0.22, 0.43) |
| Republic of Korea | 20 to 24 | 0.45 (0.34, 0.59) |
| Republic of Korea | 25 to 29 | 0.75 (0.59, 0.96) |
| Republic of Korea | 30 to 34 | 1.41 (1.13, 1.77) |
| Republic of Korea | 35 to 39 | 2.46 (1.92, 3.15) |
| Republic of Moldova | 15 to 19 | 0.66 (0.26, 1.69) |
| Republic of Moldova | 20 to 24 | 0.57 (0.23, 1.41) |
| Republic of Moldova | 25 to 29 | 0.45 (0.18, 1.15) |
| Republic of Moldova | 30 to 34 | 0.71 (0.29, 1.75) |
| Republic of Moldova | 35 to 39 | 1.44 (0.56, 3.73) |
| Romania | 15 to 19 | 0.48 (0.32, 0.72) |
| Romania | 20 to 24 | 0.6 (0.41, 0.87) |
| Romania | 25 to 29 | 0.97 (0.69, 1.37) |
| Romania | 30 to 34 | 1.8 (1.3, 2.48) |
| Romania | 35 to 39 | 3.75 (2.63, 5.33) |
| Russian Federation | 15 to 19 | 0.22 (0.17, 0.28) |
| Russian Federation | 20 to 24 | 0.26 (0.21, 0.33) |
| Russian Federation | 25 to 29 | 0.28 (0.23, 0.34) |
| Russian Federation | 30 to 34 | 0.47 (0.39, 0.57) |
| Russian Federation | 35 to 39 | 0.87 (0.71, 1.06) |
| Rwanda | 15 to 19 | 0.97 (0.65, 1.44) |
| Rwanda | 20 to 24 | 0.86 (0.58, 1.29) |
| Rwanda | 25 to 29 | 1.16 (0.8, 1.69) |
| Rwanda | 30 to 34 | 1.66 (1.17, 2.35) |
| Rwanda | 35 to 39 | 2.98 (2.01, 4.42) |
| Samoa | 15 to 19 | 0.51 (0.01, 29.51) |
| Samoa | 20 to 24 | 0.62 (0.01, 29.91) |
| Samoa | 25 to 29 | 0.71 (0.02, 33.05) |
| Samoa | 30 to 34 | 0.78 (0.02, 39.67) |
| Samoa | 35 to 39 | 8.46 (0.21, 342.72) |
| Saudi Arabia | 15 to 19 | 0.81 (0.64, 1.03) |
| Saudi Arabia | 20 to 24 | 1.09 (0.89, 1.34) |
| Saudi Arabia | 25 to 29 | 1.82 (1.54, 2.16) |
| Saudi Arabia | 30 to 34 | 3.66 (3.18, 4.21) |
| Saudi Arabia | 35 to 39 | 6.03 (5.04, 7.23) |
| Senegal | 15 to 19 | 0.06 (0.01, 0.23) |
| Senegal | 20 to 24 | 0.17 (0.07, 0.44) |
| Senegal | 25 to 29 | 0.11 (0.04, 0.35) |
| Senegal | 30 to 34 | 0.33 (0.13, 0.84) |
| Senegal | 35 to 39 | 0.45 (0.14, 1.42) |
| Serbia | 15 to 19 | 0.28 (0.12, 0.64) |
| Serbia | 20 to 24 | 0.32 (0.15, 0.71) |
| Serbia | 25 to 29 | 0.45 (0.22, 0.93) |
| Serbia | 30 to 34 | 0.83 (0.43, 1.59) |
| Serbia | 35 to 39 | 1.46 (0.71, 3.02) |
| Seychelles | 15 to 19 | 1.31 (0.02, 75.58) |
| Seychelles | 20 to 24 | 1.3 (0.03, 62.11) |
| Seychelles | 25 to 29 | 1.26 (0.03, 58.5) |
| Seychelles | 30 to 34 | 1.21 (0.02, 62.05) |
| Seychelles | 35 to 39 | 11.97 (0.3, 484.79) |
| Sierra Leone | 15 to 19 | 0.02 (0, 0.48) |
| Sierra Leone | 20 to 24 | 0.18 (0.04, 0.81) |
| Sierra Leone | 25 to 29 | 0.15 (0.03, 0.69) |
| Sierra Leone | 30 to 34 | 0.43 (0.11, 1.65) |
| Sierra Leone | 35 to 39 | 0.69 (0.12, 4.1) |
| Singapore | 15 to 19 | 4.13 (3.05, 5.59) |
| Singapore | 20 to 24 | 5.69 (4.48, 7.23) |
| Singapore | 25 to 29 | 6.78 (5.45, 8.44) |
| Singapore | 30 to 34 | 10.79 (8.77, 13.29) |
| Singapore | 35 to 39 | 16.75 (13.32, 21.06) |
| Slovakia | 15 to 19 | 0.27 (0.09, 0.8) |
| Slovakia | 20 to 24 | 0.38 (0.15, 0.94) |
| Slovakia | 25 to 29 | 0.54 (0.23, 1.27) |
| Slovakia | 30 to 34 | 1.04 (0.49, 2.22) |
| Slovakia | 35 to 39 | 2.01 (0.86, 4.7) |
| Slovenia | 15 to 19 | 0.67 (0.18, 2.47) |
| Slovenia | 20 to 24 | 0.63 (0.18, 2.19) |
| Slovenia | 25 to 29 | 0.63 (0.19, 2.1) |
| Slovenia | 30 to 34 | 1.08 (0.33, 3.57) |
| Slovenia | 35 to 39 | 1.55 (0.44, 5.48) |
| Solomon Islands | 15 to 19 | 0.2 (0, 11.29) |
| Solomon Islands | 20 to 24 | 0.21 (0, 9.87) |
| Solomon Islands | 25 to 29 | 0.21 (0, 9.95) |
| Solomon Islands | 30 to 34 | 0.22 (0, 11.29) |
| Solomon Islands | 35 to 39 | 2.27 (0.06, 91.82) |
| Somalia | 15 to 19 | 0.7 (0.46, 1.07) |
| Somalia | 20 to 24 | 0.62 (0.41, 0.94) |
| Somalia | 25 to 29 | 0.92 (0.62, 1.36) |
| Somalia | 30 to 34 | 1.42 (0.99, 2.04) |
| Somalia | 35 to 39 | 3.03 (2.02, 4.56) |
| South Africa | 15 to 19 | 0.23 (0.17, 0.33) |
| South Africa | 20 to 24 | 0.28 (0.21, 0.38) |
| South Africa | 25 to 29 | 0.34 (0.25, 0.46) |
| South Africa | 30 to 34 | 0.5 (0.38, 0.66) |
| South Africa | 35 to 39 | 0.81 (0.59, 1.09) |
| South Sudan | 15 to 19 | 0.53 (0.29, 0.96) |
| South Sudan | 20 to 24 | 0.49 (0.27, 0.88) |
| South Sudan | 25 to 29 | 0.78 (0.46, 1.32) |
| South Sudan | 30 to 34 | 1.23 (0.73, 2.08) |
| South Sudan | 35 to 39 | 2.54 (1.44, 4.49) |
| Spain | 15 to 19 | 1.49 (1.24, 1.8) |
| Spain | 20 to 24 | 1.7 (1.44, 2.01) |
| Spain | 25 to 29 | 2.02 (1.72, 2.36) |
| Spain | 30 to 34 | 2.88 (2.48, 3.35) |
| Spain | 35 to 39 | 3.85 (3.28, 4.53) |
| Sri Lanka | 15 to 19 | 0.79 (0.59, 1.07) |
| Sri Lanka | 20 to 24 | 1.15 (0.88, 1.5) |
| Sri Lanka | 25 to 29 | 1.74 (1.36, 2.21) |
| Sri Lanka | 30 to 34 | 2.88 (2.31, 3.59) |
| Sri Lanka | 35 to 39 | 4.43 (3.45, 5.68) |
| Sudan | 15 to 19 | 0.23 (0.14, 0.35) |
| Sudan | 20 to 24 | 0.23 (0.15, 0.36) |
| Sudan | 25 to 29 | 0.35 (0.23, 0.52) |
| Sudan | 30 to 34 | 0.52 (0.36, 0.75) |
| Sudan | 35 to 39 | 0.79 (0.51, 1.21) |
| Suriname | 15 to 19 | 0.21 (0, 12.4) |
| Suriname | 20 to 24 | 0.21 (0, 10.01) |
| Suriname | 25 to 29 | 0.24 (0, 11.2) |
| Suriname | 30 to 34 | 0.29 (0.01, 15.28) |
| Suriname | 35 to 39 | 1.73 (0.04, 74.09) |
| Sweden | 15 to 19 | 0.52 (0.28, 0.95) |
| Sweden | 20 to 24 | 0.72 (0.43, 1.21) |
| Sweden | 25 to 29 | 1.96 (1.34, 2.87) |
| Sweden | 30 to 34 | 2.4 (1.66, 3.46) |
| Sweden | 35 to 39 | 1.59 (1.02, 2.49) |
| Switzerland | 15 to 19 | 0.77 (0.4, 1.45) |
| Switzerland | 20 to 24 | 0.8 (0.45, 1.4) |
| Switzerland | 25 to 29 | 0.74 (0.42, 1.31) |
| Switzerland | 30 to 34 | 0.96 (0.56, 1.67) |
| Switzerland | 35 to 39 | 1.14 (0.63, 2.06) |
| Syrian Arab Republic | 15 to 19 | 0.15 (0.07, 0.31) |
| Syrian Arab Republic | 20 to 24 | 0.15 (0.07, 0.31) |
| Syrian Arab Republic | 25 to 29 | 0.21 (0.1, 0.44) |
| Syrian Arab Republic | 30 to 34 | 0.33 (0.16, 0.67) |
| Syrian Arab Republic | 35 to 39 | 0.43 (0.19, 0.97) |
| Taiwan (Province of China) | 15 to 19 | 5.91 (5.32, 6.57) |
| Taiwan (Province of China) | 20 to 24 | 7.14 (6.5, 7.84) |
| Taiwan (Province of China) | 25 to 29 | 13 (12.01, 14.08) |
| Taiwan (Province of China) | 30 to 34 | 28.72 (26.76, 30.84) |
| Taiwan (Province of China) | 35 to 39 | 42.3 (39.18, 45.67) |
| Tajikistan | 15 to 19 | 0.54 (0.29, 1.02) |
| Tajikistan | 20 to 24 | 0.47 (0.25, 0.88) |
| Tajikistan | 25 to 29 | 0.66 (0.38, 1.14) |
| Tajikistan | 30 to 34 | 0.68 (0.38, 1.21) |
| Tajikistan | 35 to 39 | 0.85 (0.43, 1.68) |
| Thailand | 15 to 19 | 2.35 (2.08, 2.66) |
| Thailand | 20 to 24 | 3.05 (2.74, 3.4) |
| Thailand | 25 to 29 | 5.58 (5.08, 6.14) |
| Thailand | 30 to 34 | 10.69 (9.82, 11.64) |
| Thailand | 35 to 39 | 15.88 (14.44, 17.47) |
| Timor-Leste | 15 to 19 | 0.18 (0.01, 2.57) |
| Timor-Leste | 20 to 24 | 0.33 (0.04, 2.92) |
| Timor-Leste | 25 to 29 | 1.25 (0.31, 4.94) |
| Timor-Leste | 30 to 34 | 1.96 (0.56, 6.85) |
| Timor-Leste | 35 to 39 | 2.74 (0.58, 13.02) |
| Togo | 15 to 19 | 0.02 (0, 0.47) |
| Togo | 20 to 24 | 0.25 (0.07, 0.91) |
| Togo | 25 to 29 | 0.13 (0.03, 0.59) |
| Togo | 30 to 34 | 0.33 (0.1, 1.08) |
| Togo | 35 to 39 | 0.47 (0.11, 2.02) |
| Trinidad and Tobago | 15 to 19 | 0.11 (0, 2.44) |
| Trinidad and Tobago | 20 to 24 | 0.36 (0.06, 2.16) |
| Trinidad and Tobago | 25 to 29 | 1.04 (0.23, 4.59) |
| Trinidad and Tobago | 30 to 34 | 1.52 (0.32, 7.25) |
| Trinidad and Tobago | 35 to 39 | 2.75 (0.38, 20.16) |
| Tunisia | 15 to 19 | 2.31 (1.83, 2.92) |
| Tunisia | 20 to 24 | 2.67 (2.15, 3.32) |
| Tunisia | 25 to 29 | 4.34 (3.58, 5.26) |
| Tunisia | 30 to 34 | 8.92 (7.53, 10.57) |
| Tunisia | 35 to 39 | 12.98 (10.64, 15.83) |
| Turkey | 15 to 19 | 0.89 (0.76, 1.05) |
| Turkey | 20 to 24 | 0.87 (0.75, 1.01) |
| Turkey | 25 to 29 | 1.15 (1, 1.33) |
| Turkey | 30 to 34 | 1.82 (1.59, 2.08) |
| Turkey | 35 to 39 | 2.47 (2.12, 2.89) |
| Turkmenistan | 15 to 19 | 0.53 (0.23, 1.21) |
| Turkmenistan | 20 to 24 | 0.58 (0.26, 1.26) |
| Turkmenistan | 25 to 29 | 0.82 (0.41, 1.64) |
| Turkmenistan | 30 to 34 | 0.89 (0.43, 1.82) |
| Turkmenistan | 35 to 39 | 1.25 (0.53, 2.9) |
| Uganda | 15 to 19 | 1.73 (1.45, 2.06) |
| Uganda | 20 to 24 | 1.57 (1.32, 1.88) |
| Uganda | 25 to 29 | 2.21 (1.87, 2.61) |
| Uganda | 30 to 34 | 3.43 (2.93, 4) |
| Uganda | 35 to 39 | 6.68 (5.6, 7.98) |
| Ukraine | 15 to 19 | 0.29 (0.2, 0.42) |
| Ukraine | 20 to 24 | 0.35 (0.25, 0.48) |
| Ukraine | 25 to 29 | 0.45 (0.33, 0.61) |
| Ukraine | 30 to 34 | 0.66 (0.5, 0.88) |
| Ukraine | 35 to 39 | 1.29 (0.95, 1.77) |
| United Arab Emirates | 15 to 19 | 0.68 (0.32, 1.42) |
| United Arab Emirates | 20 to 24 | 0.92 (0.58, 1.47) |
| United Arab Emirates | 25 to 29 | 1.08 (0.75, 1.57) |
| United Arab Emirates | 30 to 34 | 1.24 (0.85, 1.81) |
| United Arab Emirates | 35 to 39 | 1.56 (0.97, 2.49) |
| United Kingdom | 15 to 19 | 1.65 (1.42, 1.91) |
| United Kingdom | 20 to 24 | 1.89 (1.66, 2.15) |
| United Kingdom | 25 to 29 | 2.32 (2.05, 2.62) |
| United Kingdom | 30 to 34 | 3.3 (2.94, 3.71) |
| United Kingdom | 35 to 39 | 3.96 (3.47, 4.53) |
| United Republic of Tanzania | 15 to 19 | 0.72 (0.58, 0.9) |
| United Republic of Tanzania | 20 to 24 | 0.68 (0.54, 0.85) |
| United Republic of Tanzania | 25 to 29 | 1.05 (0.86, 1.28) |
| United Republic of Tanzania | 30 to 34 | 1.66 (1.38, 2.01) |
| United Republic of Tanzania | 35 to 39 | 3.16 (2.55, 3.92) |
| United States of America | 15 to 19 | 1.19 (1.1, 1.29) |
| United States of America | 20 to 24 | 1.73 (1.61, 1.85) |
| United States of America | 25 to 29 | 2.12 (1.99, 2.27) |
| United States of America | 30 to 34 | 2.72 (2.55, 2.9) |
| United States of America | 35 to 39 | 4.12 (3.84, 4.42) |
| Uruguay | 15 to 19 | 0.42 (0.14, 1.3) |
| Uruguay | 20 to 24 | 0.52 (0.2, 1.39) |
| Uruguay | 25 to 29 | 0.57 (0.2, 1.64) |
| Uruguay | 30 to 34 | 0.99 (0.37, 2.64) |
| Uruguay | 35 to 39 | 1.34 (0.45, 4) |
| Uzbekistan | 15 to 19 | 0.47 (0.34, 0.66) |
| Uzbekistan | 20 to 24 | 0.47 (0.34, 0.65) |
| Uzbekistan | 25 to 29 | 0.73 (0.55, 0.98) |
| Uzbekistan | 30 to 34 | 0.8 (0.6, 1.07) |
| Uzbekistan | 35 to 39 | 1.02 (0.72, 1.43) |
| Venezuela (Bolivarian Republic of) | 15 to 19 | 0.31 (0.2, 0.48) |
| Venezuela (Bolivarian Republic of) | 20 to 24 | 0.39 (0.26, 0.57) |
| Venezuela (Bolivarian Republic of) | 25 to 29 | 0.5 (0.35, 0.73) |
| Venezuela (Bolivarian Republic of) | 30 to 34 | 0.71 (0.49, 1.03) |
| Venezuela (Bolivarian Republic of) | 35 to 39 | 1.02 (0.66, 1.58) |
| Viet Nam | 15 to 19 | 2.54 (2.34, 2.75) |
| Viet Nam | 20 to 24 | 2.65 (2.46, 2.86) |
| Viet Nam | 25 to 29 | 3.95 (3.68, 4.22) |
| Viet Nam | 30 to 34 | 8.37 (7.9, 8.88) |
| Viet Nam | 35 to 39 | 16.74 (15.64, 17.92) |
| Yemen | 15 to 19 | 0.11 (0.05, 0.22) |
| Yemen | 20 to 24 | 0.12 (0.06, 0.24) |
| Yemen | 25 to 29 | 0.24 (0.14, 0.42) |
| Yemen | 30 to 34 | 0.42 (0.25, 0.71) |
| Yemen | 35 to 39 | 0.73 (0.4, 1.33) |
| Zambia | 15 to 19 | 0.86 (0.6, 1.23) |
| Zambia | 20 to 24 | 0.97 (0.7, 1.36) |
| Zambia | 25 to 29 | 1.54 (1.14, 2.08) |
| Zambia | 30 to 34 | 2.72 (2.08, 3.56) |
| Zambia | 35 to 39 | 6.06 (4.4, 8.35) |
| Zimbabwe | 15 to 19 | 0.34 (0.21, 0.58) |
| Zimbabwe | 20 to 24 | 0.39 (0.23, 0.65) |
| Zimbabwe | 25 to 29 | 0.62 (0.39, 1) |
| Zimbabwe | 30 to 34 | 1.11 (0.7, 1.75) |
| Zimbabwe | 35 to 39 | 2.62 (1.56, 4.38) |
