# Supplementary material for: Global, regional, and national temporal trends in prevalence for nasopharynx cancer across adolescents and young adults, 1990–2021: an age-period-cohort analysis based on the global burden of disease study 2021
Source: BMC Oral Health. 2025 Sep 26;25:1435. doi: 10.1186/s12903-025-06750-4 (PMC12465747; doi:10.1186/s12903-025-06750-4)
Supplement: Supplementary file 8 — Supplementary Material 8. Period effects on nasopharynx cancer prevalence in adolescents and young adults across countries. [file 12903_2025_6750_MOESM8_ESM.docx]

**Supplementary Table 8** Period effects on nasopharynx cancer prevalence in adolescents and young adults across countries

| **Location** | **Period** | **Prevalence rate ratio** |
| --- | --- | --- |
| Afghanistan | 1992 to 1996 | 1.1 (0.63, 1.91) |
| Afghanistan | 1997 to 2001 | 1.11 (0.68, 1.8) |
| Afghanistan | 2002 to 2006 | 1.00 (1.00, 1.00) |
| Afghanistan | 2007 to 2011 | 0.96 (0.61, 1.49) |
| Afghanistan | 2012 to 2016 | 0.91 (0.58, 1.44) |
| Afghanistan | 2017 to 2021 | 0.92 (0.58, 1.44) |
| Albania | 1992 to 1996 | 0.44 (0.15, 1.3) |
| Albania | 1997 to 2001 | 0.76 (0.32, 1.8) |
| Albania | 2002 to 2006 | 1.00 (1.00, 1.00) |
| Albania | 2007 to 2011 | 0.96 (0.42, 2.21) |
| Albania | 2012 to 2016 | 0.9 (0.38, 2.14) |
| Albania | 2017 to 2021 | 0.99 (0.42, 2.32) |
| Algeria | 1992 to 1996 | 0.94 (0.82, 1.09) |
| Algeria | 1997 to 2001 | 0.99 (0.87, 1.12) |
| Algeria | 2002 to 2006 | 1.00 (1.00, 1.00) |
| Algeria | 2007 to 2011 | 0.98 (0.87, 1.11) |
| Algeria | 2012 to 2016 | 1.03 (0.92, 1.16) |
| Algeria | 2017 to 2021 | 1.16 (1.04, 1.3) |
| Angola | 1992 to 1996 | 1.2 (0.51, 2.84) |
| Angola | 1997 to 2001 | 1.1 (0.5, 2.42) |
| Angola | 2002 to 2006 | 1.00 (1.00, 1.00) |
| Angola | 2007 to 2011 | 1.1 (0.54, 2.25) |
| Angola | 2012 to 2016 | 1.15 (0.58, 2.27) |
| Angola | 2017 to 2021 | 1.21 (0.62, 2.36) |
| Argentina | 1992 to 1996 | 1.02 (0.66, 1.56) |
| Argentina | 1997 to 2001 | 1.04 (0.71, 1.55) |
| Argentina | 2002 to 2006 | 1.00 (1.00, 1.00) |
| Argentina | 2007 to 2011 | 1.08 (0.75, 1.57) |
| Argentina | 2012 to 2016 | 1.05 (0.72, 1.53) |
| Argentina | 2017 to 2021 | 1.02 (0.7, 1.48) |
| Armenia | 1992 to 1996 | 0.87 (0.23, 3.34) |
| Armenia | 1997 to 2001 | 0.94 (0.26, 3.37) |
| Armenia | 2002 to 2006 | 1.00 (1.00, 1.00) |
| Armenia | 2007 to 2011 | 1.05 (0.29, 3.75) |
| Armenia | 2012 to 2016 | 1.08 (0.3, 3.95) |
| Armenia | 2017 to 2021 | 1.11 (0.29, 4.19) |
| Australia | 1992 to 1996 | 1.19 (0.99, 1.42) |
| Australia | 1997 to 2001 | 1.23 (1.06, 1.43) |
| Australia | 2002 to 2006 | 1.00 (1.00, 1.00) |
| Australia | 2007 to 2011 | 0.91 (0.79, 1.06) |
| Australia | 2012 to 2016 | 0.76 (0.65, 0.89) |
| Australia | 2017 to 2021 | 0.71 (0.6, 0.83) |
| Austria | 1992 to 1996 | 0.89 (0.54, 1.48) |
| Austria | 1997 to 2001 | 1.02 (0.66, 1.58) |
| Austria | 2002 to 2006 | 1.00 (1.00, 1.00) |
| Austria | 2007 to 2011 | 0.94 (0.6, 1.47) |
| Austria | 2012 to 2016 | 0.88 (0.56, 1.4) |
| Austria | 2017 to 2021 | 0.79 (0.5, 1.25) |
| Azerbaijan | 1992 to 1996 | 1.26 (0.48, 3.33) |
| Azerbaijan | 1997 to 2001 | 1.08 (0.41, 2.84) |
| Azerbaijan | 2002 to 2006 | 1.00 (1.00, 1.00) |
| Azerbaijan | 2007 to 2011 | 1.05 (0.41, 2.64) |
| Azerbaijan | 2012 to 2016 | 1.11 (0.44, 2.78) |
| Azerbaijan | 2017 to 2021 | 1.28 (0.52, 3.18) |
| Bahrain | 1992 to 1996 | 0.69 (0.06, 7.33) |
| Bahrain | 1997 to 2001 | 1.14 (0.23, 5.71) |
| Bahrain | 2002 to 2006 | 1.00 (1.00, 1.00) |
| Bahrain | 2007 to 2011 | 0.88 (0.24, 3.3) |
| Bahrain | 2012 to 2016 | 1.17 (0.32, 4.27) |
| Bahrain | 2017 to 2021 | 1.12 (0.3, 4.19) |
| Bangladesh | 1992 to 1996 | 1.07 (0.94, 1.22) |
| Bangladesh | 1997 to 2001 | 1 (0.89, 1.13) |
| Bangladesh | 2002 to 2006 | 1.00 (1.00, 1.00) |
| Bangladesh | 2007 to 2011 | 1.04 (0.93, 1.16) |
| Bangladesh | 2012 to 2016 | 1.02 (0.91, 1.14) |
| Bangladesh | 2017 to 2021 | 1.15 (1.04, 1.28) |
| Belarus | 1992 to 1996 | 1 (0.44, 2.27) |
| Belarus | 1997 to 2001 | 1.09 (0.55, 2.18) |
| Belarus | 2002 to 2006 | 1.00 (1.00, 1.00) |
| Belarus | 2007 to 2011 | 1.07 (0.54, 2.13) |
| Belarus | 2012 to 2016 | 1.02 (0.5, 2.06) |
| Belarus | 2017 to 2021 | 0.85 (0.41, 1.77) |
| Belgium | 1992 to 1996 | 1.15 (0.76, 1.75) |
| Belgium | 1997 to 2001 | 1.2 (0.82, 1.74) |
| Belgium | 2002 to 2006 | 1.00 (1.00, 1.00) |
| Belgium | 2007 to 2011 | 0.97 (0.66, 1.42) |
| Belgium | 2012 to 2016 | 1.04 (0.71, 1.53) |
| Belgium | 2017 to 2021 | 1.04 (0.71, 1.52) |
| Benin | 1992 to 1996 | 1.48 (0.22, 10.04) |
| Benin | 1997 to 2001 | 1.22 (0.22, 6.77) |
| Benin | 2002 to 2006 | 1.00 (1.00, 1.00) |
| Benin | 2007 to 2011 | 0.77 (0.14, 4.22) |
| Benin | 2012 to 2016 | 1.24 (0.26, 5.86) |
| Benin | 2017 to 2021 | 1.16 (0.25, 5.28) |
| Bhutan | 1992 to 1996 | 1.03 (0.13, 8.07) |
| Bhutan | 1997 to 2001 | 0.77 (0.1, 5.76) |
| Bhutan | 2002 to 2006 | 1.00 (1.00, 1.00) |
| Bhutan | 2007 to 2011 | 0.9 (0.17, 4.66) |
| Bhutan | 2012 to 2016 | 0.92 (0.16, 5.18) |
| Bhutan | 2017 to 2021 | 0.77 (0.13, 4.66) |
| Bolivia (Plurinational State of) | 1992 to 1996 | 1.39 (0.38, 5.07) |
| Bolivia (Plurinational State of) | 1997 to 2001 | 1.18 (0.35, 4.02) |
| Bolivia (Plurinational State of) | 2002 to 2006 | 1.00 (1.00, 1.00) |
| Bolivia (Plurinational State of) | 2007 to 2011 | 0.89 (0.27, 2.97) |
| Bolivia (Plurinational State of) | 2012 to 2016 | 0.96 (0.3, 3.06) |
| Bolivia (Plurinational State of) | 2017 to 2021 | 0.95 (0.31, 2.92) |
| Bosnia and Herzegovina | 1992 to 1996 | 0.35 (0.03, 4.52) |
| Bosnia and Herzegovina | 1997 to 2001 | 0.51 (0.07, 3.71) |
| Bosnia and Herzegovina | 2002 to 2006 | 1.00 (1.00, 1.00) |
| Bosnia and Herzegovina | 2007 to 2011 | 1.02 (0.24, 4.33) |
| Bosnia and Herzegovina | 2012 to 2016 | 0.96 (0.22, 4.29) |
| Bosnia and Herzegovina | 2017 to 2021 | 0.65 (0.11, 3.72) |
| Botswana | 1992 to 1996 | 0.9 (0.14, 5.8) |
| Botswana | 1997 to 2001 | 0.86 (0.16, 4.54) |
| Botswana | 2002 to 2006 | 1.00 (1.00, 1.00) |
| Botswana | 2007 to 2011 | 0.94 (0.23, 3.79) |
| Botswana | 2012 to 2016 | 0.92 (0.23, 3.61) |
| Botswana | 2017 to 2021 | 0.87 (0.21, 3.59) |
| Brazil | 1992 to 1996 | 0.6 (0.5, 0.73) |
| Brazil | 1997 to 2001 | 0.87 (0.74, 1.02) |
| Brazil | 2002 to 2006 | 1.00 (1.00, 1.00) |
| Brazil | 2007 to 2011 | 1.05 (0.9, 1.21) |
| Brazil | 2012 to 2016 | 1.06 (0.92, 1.23) |
| Brazil | 2017 to 2021 | 0.97 (0.84, 1.13) |
| Brunei Darussalam | 1992 to 1996 | 1.44 (0.32, 6.51) |
| Brunei Darussalam | 1997 to 2001 | 1.21 (0.31, 4.71) |
| Brunei Darussalam | 2002 to 2006 | 1.00 (1.00, 1.00) |
| Brunei Darussalam | 2007 to 2011 | 1.21 (0.35, 4.23) |
| Brunei Darussalam | 2012 to 2016 | 1.1 (0.3, 4.01) |
| Brunei Darussalam | 2017 to 2021 | 1.24 (0.34, 4.55) |
| Bulgaria | 1992 to 1996 | 0.88 (0.46, 1.67) |
| Bulgaria | 1997 to 2001 | 0.93 (0.52, 1.67) |
| Bulgaria | 2002 to 2006 | 1.00 (1.00, 1.00) |
| Bulgaria | 2007 to 2011 | 1.15 (0.67, 2) |
| Bulgaria | 2012 to 2016 | 1.26 (0.72, 2.21) |
| Bulgaria | 2017 to 2021 | 1.42 (0.83, 2.46) |
| Burkina Faso | 1992 to 1996 | 1.11 (0.28, 4.37) |
| Burkina Faso | 1997 to 2001 | 1.09 (0.34, 3.55) |
| Burkina Faso | 2002 to 2006 | 1.00 (1.00, 1.00) |
| Burkina Faso | 2007 to 2011 | 1.06 (0.38, 2.97) |
| Burkina Faso | 2012 to 2016 | 1.17 (0.42, 3.3) |
| Burkina Faso | 2017 to 2021 | 1.28 (0.48, 3.45) |
| Burundi | 1992 to 1996 | 1.2 (0.69, 2.09) |
| Burundi | 1997 to 2001 | 1.15 (0.69, 1.93) |
| Burundi | 2002 to 2006 | 1.00 (1.00, 1.00) |
| Burundi | 2007 to 2011 | 0.97 (0.6, 1.57) |
| Burundi | 2012 to 2016 | 0.96 (0.61, 1.53) |
| Burundi | 2017 to 2021 | 0.97 (0.62, 1.52) |
| Cambodia | 1992 to 1996 | 1.05 (0.73, 1.49) |
| Cambodia | 1997 to 2001 | 1.05 (0.77, 1.44) |
| Cambodia | 2002 to 2006 | 1.00 (1.00, 1.00) |
| Cambodia | 2007 to 2011 | 1.04 (0.78, 1.39) |
| Cambodia | 2012 to 2016 | 1.15 (0.87, 1.52) |
| Cambodia | 2017 to 2021 | 1.27 (0.98, 1.66) |
| Cameroon | 1992 to 1996 | 1.26 (0.45, 3.51) |
| Cameroon | 1997 to 2001 | 1.06 (0.41, 2.74) |
| Cameroon | 2002 to 2006 | 1.00 (1.00, 1.00) |
| Cameroon | 2007 to 2011 | 1.15 (0.51, 2.6) |
| Cameroon | 2012 to 2016 | 1.21 (0.54, 2.71) |
| Cameroon | 2017 to 2021 | 1.25 (0.58, 2.69) |
| Canada | 1992 to 1996 | 1.01 (0.82, 1.23) |
| Canada | 1997 to 2001 | 1.06 (0.89, 1.27) |
| Canada | 2002 to 2006 | 1.00 (1.00, 1.00) |
| Canada | 2007 to 2011 | 0.93 (0.77, 1.11) |
| Canada | 2012 to 2016 | 0.85 (0.71, 1.03) |
| Canada | 2017 to 2021 | 0.95 (0.79, 1.13) |
| Central African Republic | 1992 to 1996 | 1.24 (0.21, 7.35) |
| Central African Republic | 1997 to 2001 | 1.17 (0.32, 4.21) |
| Central African Republic | 2002 to 2006 | 1.00 (1.00, 1.00) |
| Central African Republic | 2007 to 2011 | 1.05 (0.3, 3.59) |
| Central African Republic | 2012 to 2016 | 0.98 (0.28, 3.42) |
| Central African Republic | 2017 to 2021 | 0.95 (0.26, 3.44) |
| Chad | 1992 to 1996 | 1.37 (0.21, 8.87) |
| Chad | 1997 to 2001 | 1.29 (0.24, 6.99) |
| Chad | 2002 to 2006 | 1.00 (1.00, 1.00) |
| Chad | 2007 to 2011 | 1.43 (0.32, 6.47) |
| Chad | 2012 to 2016 | 1.76 (0.4, 7.63) |
| Chad | 2017 to 2021 | 1.73 (0.43, 6.86) |
| Chile | 1992 to 1996 | 1.07 (0.44, 2.58) |
| Chile | 1997 to 2001 | 1.04 (0.46, 2.34) |
| Chile | 2002 to 2006 | 1.00 (1.00, 1.00) |
| Chile | 2007 to 2011 | 1.31 (0.62, 2.76) |
| Chile | 2012 to 2016 | 1.2 (0.56, 2.58) |
| Chile | 2017 to 2021 | 1.39 (0.66, 2.9) |
| China | 1992 to 1996 | 1.07 (0.95, 1.2) |
| China | 1997 to 2001 | 1.09 (0.99, 1.21) |
| China | 2002 to 2006 | 1.00 (1.00, 1.00) |
| China | 2007 to 2011 | 1.05 (0.95, 1.16) |
| China | 2012 to 2016 | 1.27 (1.14, 1.4) |
| China | 2017 to 2021 | 1.72 (1.57, 1.9) |
| Colombia | 1992 to 1996 | 0.98 (0.69, 1.4) |
| Colombia | 1997 to 2001 | 1.09 (0.79, 1.5) |
| Colombia | 2002 to 2006 | 1.00 (1.00, 1.00) |
| Colombia | 2007 to 2011 | 0.98 (0.71, 1.35) |
| Colombia | 2012 to 2016 | 0.91 (0.66, 1.26) |
| Colombia | 2017 to 2021 | 0.94 (0.68, 1.29) |
| Comoros | 1992 to 1996 | 0.68 (0.04, 10.49) |
| Comoros | 1997 to 2001 | 0.84 (0.1, 6.98) |
| Comoros | 2002 to 2006 | 1.00 (1.00, 1.00) |
| Comoros | 2007 to 2011 | 1.14 (0.15, 8.84) |
| Comoros | 2012 to 2016 | 1.56 (0.18, 13.39) |
| Comoros | 2017 to 2021 | 1.52 (0.22, 10.38) |
| Congo | 1992 to 1996 | 1.08 (0.19, 6.18) |
| Congo | 1997 to 2001 | 1 (0.25, 3.93) |
| Congo | 2002 to 2006 | 1.00 (1.00, 1.00) |
| Congo | 2007 to 2011 | 1.02 (0.27, 3.76) |
| Congo | 2012 to 2016 | 0.89 (0.24, 3.26) |
| Congo | 2017 to 2021 | 1.05 (0.31, 3.56) |
| Costa Rica | 1992 to 1996 | 0.95 (0.41, 2.23) |
| Costa Rica | 1997 to 2001 | 1.03 (0.48, 2.22) |
| Costa Rica | 2002 to 2006 | 1.00 (1.00, 1.00) |
| Costa Rica | 2007 to 2011 | 0.94 (0.44, 1.98) |
| Costa Rica | 2012 to 2016 | 0.93 (0.44, 1.95) |
| Costa Rica | 2017 to 2021 | 0.95 (0.46, 1.99) |
| Coted'Ivoire | 1992 to 1996 | 0.88 (0.4, 1.92) |
| Coted'Ivoire | 1997 to 2001 | 1.01 (0.53, 1.93) |
| Coted'Ivoire | 2002 to 2006 | 1.00 (1.00, 1.00) |
| Coted'Ivoire | 2007 to 2011 | 0.99 (0.54, 1.81) |
| Coted'Ivoire | 2012 to 2016 | 1.16 (0.65, 2.09) |
| Coted'Ivoire | 2017 to 2021 | 1.14 (0.65, 2.01) |
| Croatia | 1992 to 1996 | 0.66 (0.22, 2.01) |
| Croatia | 1997 to 2001 | 1.01 (0.41, 2.5) |
| Croatia | 2002 to 2006 | 1.00 (1.00, 1.00) |
| Croatia | 2007 to 2011 | 1.1 (0.46, 2.63) |
| Croatia | 2012 to 2016 | 1.07 (0.44, 2.63) |
| Croatia | 2017 to 2021 | 0.89 (0.35, 2.29) |
| Cuba | 1992 to 1996 | 0.93 (0.55, 1.58) |
| Cuba | 1997 to 2001 | 0.99 (0.61, 1.59) |
| Cuba | 2002 to 2006 | 1.00 (1.00, 1.00) |
| Cuba | 2007 to 2011 | 0.98 (0.6, 1.6) |
| Cuba | 2012 to 2016 | 1.03 (0.62, 1.72) |
| Cuba | 2017 to 2021 | 1.14 (0.69, 1.88) |
| Cyprus | 1992 to 1996 | 0.26 (0.01, 6.47) |
| Cyprus | 1997 to 2001 | 0.93 (0.11, 7.85) |
| Cyprus | 2002 to 2006 | 1.00 (1.00, 1.00) |
| Cyprus | 2007 to 2011 | 0.99 (0.2, 4.99) |
| Cyprus | 2012 to 2016 | 0.97 (0.19, 4.91) |
| Cyprus | 2017 to 2021 | 0.9 (0.17, 4.8) |
| Czechia | 1992 to 1996 | 0.54 (0.33, 0.9) |
| Czechia | 1997 to 2001 | 0.72 (0.48, 1.1) |
| Czechia | 2002 to 2006 | 1.00 (1.00, 1.00) |
| Czechia | 2007 to 2011 | 1.09 (0.76, 1.56) |
| Czechia | 2012 to 2016 | 0.97 (0.66, 1.41) |
| Czechia | 2017 to 2021 | 0.88 (0.59, 1.3) |
| Democratic People's Republic of Korea | 1992 to 1996 | 1.17 (0.97, 1.41) |
| Democratic People's Republic of Korea | 1997 to 2001 | 1.06 (0.9, 1.24) |
| Democratic People's Republic of Korea | 2002 to 2006 | 1.00 (1.00, 1.00) |
| Democratic People's Republic of Korea | 2007 to 2011 | 1.08 (0.93, 1.26) |
| Democratic People's Republic of Korea | 2012 to 2016 | 1.11 (0.95, 1.31) |
| Democratic People's Republic of Korea | 2017 to 2021 | 1.26 (1.08, 1.47) |
| Democratic Republic of the Congo | 1992 to 1996 | 1.18 (0.7, 1.99) |
| Democratic Republic of the Congo | 1997 to 2001 | 1.08 (0.67, 1.75) |
| Democratic Republic of the Congo | 2002 to 2006 | 1.00 (1.00, 1.00) |
| Democratic Republic of the Congo | 2007 to 2011 | 1 (0.64, 1.57) |
| Democratic Republic of the Congo | 2012 to 2016 | 1.11 (0.72, 1.71) |
| Democratic Republic of the Congo | 2017 to 2021 | 1.19 (0.79, 1.8) |
| Denmark | 1992 to 1996 | 0.89 (0.39, 2.02) |
| Denmark | 1997 to 2001 | 0.92 (0.45, 1.86) |
| Denmark | 2002 to 2006 | 1.00 (1.00, 1.00) |
| Denmark | 2007 to 2011 | 0.99 (0.5, 1.96) |
| Denmark | 2012 to 2016 | 0.8 (0.38, 1.68) |
| Denmark | 2017 to 2021 | 0.83 (0.41, 1.69) |
| Djibouti | 1992 to 1996 | 0.6 (0.05, 7.31) |
| Djibouti | 1997 to 2001 | 0.61 (0.08, 4.38) |
| Djibouti | 2002 to 2006 | 1.00 (1.00, 1.00) |
| Djibouti | 2007 to 2011 | 1.1 (0.29, 4.2) |
| Djibouti | 2012 to 2016 | 0.83 (0.21, 3.26) |
| Djibouti | 2017 to 2021 | 0.89 (0.22, 3.58) |
| Dominican Republic | 1992 to 1996 | 0.77 (0.36, 1.65) |
| Dominican Republic | 1997 to 2001 | 0.99 (0.52, 1.88) |
| Dominican Republic | 2002 to 2006 | 1.00 (1.00, 1.00) |
| Dominican Republic | 2007 to 2011 | 1.04 (0.56, 1.92) |
| Dominican Republic | 2012 to 2016 | 1.12 (0.61, 2.04) |
| Dominican Republic | 2017 to 2021 | 1.01 (0.55, 1.86) |
| Ecuador | 1992 to 1996 | 0.59 (0.2, 1.7) |
| Ecuador | 1997 to 2001 | 0.84 (0.36, 1.96) |
| Ecuador | 2002 to 2006 | 1.00 (1.00, 1.00) |
| Ecuador | 2007 to 2011 | 0.97 (0.45, 2.11) |
| Ecuador | 2012 to 2016 | 0.86 (0.39, 1.89) |
| Ecuador | 2017 to 2021 | 0.68 (0.3, 1.53) |
| Egypt | 1992 to 1996 | 0.97 (0.53, 1.78) |
| Egypt | 1997 to 2001 | 1.08 (0.64, 1.83) |
| Egypt | 2002 to 2006 | 1.00 (1.00, 1.00) |
| Egypt | 2007 to 2011 | 0.95 (0.57, 1.58) |
| Egypt | 2012 to 2016 | 0.92 (0.55, 1.51) |
| Egypt | 2017 to 2021 | 0.91 (0.56, 1.49) |
| El Salvador | 1992 to 1996 | 0.76 (0.22, 2.64) |
| El Salvador | 1997 to 2001 | 0.83 (0.26, 2.72) |
| El Salvador | 2002 to 2006 | 1.00 (1.00, 1.00) |
| El Salvador | 2007 to 2011 | 1.06 (0.36, 3.1) |
| El Salvador | 2012 to 2016 | 1.43 (0.51, 3.97) |
| El Salvador | 2017 to 2021 | 1.39 (0.49, 3.9) |
| Equatorial Guinea | 1992 to 1996 | 2.36 (0.04, 156.5) |
| Equatorial Guinea | 1997 to 2001 | 1.36 (0.02, 77.97) |
| Equatorial Guinea | 2002 to 2006 | 1.00 (1.00, 1.00) |
| Equatorial Guinea | 2007 to 2011 | 0.78 (0.01, 43.65) |
| Equatorial Guinea | 2012 to 2016 | 0.65 (0.01, 38.52) |
| Equatorial Guinea | 2017 to 2021 | 1.3 (0.03, 51.84) |
| Eritrea | 1992 to 1996 | 0.93 (0.44, 1.96) |
| Eritrea | 1997 to 2001 | 0.93 (0.48, 1.79) |
| Eritrea | 2002 to 2006 | 1.00 (1.00, 1.00) |
| Eritrea | 2007 to 2011 | 0.96 (0.54, 1.72) |
| Eritrea | 2012 to 2016 | 1 (0.57, 1.75) |
| Eritrea | 2017 to 2021 | 1.01 (0.58, 1.75) |
| Estonia | 1992 to 1996 | 1.99 (0.26, 15.29) |
| Estonia | 1997 to 2001 | 1.51 (0.2, 11.19) |
| Estonia | 2002 to 2006 | 1.00 (1.00, 1.00) |
| Estonia | 2007 to 2011 | 1.62 (0.24, 10.97) |
| Estonia | 2012 to 2016 | 1.31 (0.16, 10.49) |
| Estonia | 2017 to 2021 | 0.78 (0.09, 6.54) |
| Eswatini | 1992 to 1996 | 0.59 (0.01, 25.83) |
| Eswatini | 1997 to 2001 | 0.72 (0.02, 21.74) |
| Eswatini | 2002 to 2006 | 1.00 (1.00, 1.00) |
| Eswatini | 2007 to 2011 | 1 (0.05, 19.61) |
| Eswatini | 2012 to 2016 | 1.43 (0.07, 28.02) |
| Eswatini | 2017 to 2021 | 0.85 (0.07, 9.86) |
| Ethiopia | 1992 to 1996 | 1.21 (1.02, 1.44) |
| Ethiopia | 1997 to 2001 | 1.12 (0.95, 1.32) |
| Ethiopia | 2002 to 2006 | 1.00 (1.00, 1.00) |
| Ethiopia | 2007 to 2011 | 0.93 (0.79, 1.08) |
| Ethiopia | 2012 to 2016 | 0.92 (0.79, 1.06) |
| Ethiopia | 2017 to 2021 | 1 (0.87, 1.16) |
| Finland | 1992 to 1996 | 0.78 (0.29, 2.08) |
| Finland | 1997 to 2001 | 0.86 (0.36, 2.03) |
| Finland | 2002 to 2006 | 1.00 (1.00, 1.00) |
| Finland | 2007 to 2011 | 1.04 (0.46, 2.36) |
| Finland | 2012 to 2016 | 0.94 (0.4, 2.18) |
| Finland | 2017 to 2021 | 0.88 (0.39, 2.02) |
| France | 1992 to 1996 | 0.92 (0.81, 1.05) |
| France | 1997 to 2001 | 1.02 (0.9, 1.14) |
| France | 2002 to 2006 | 1.00 (1.00, 1.00) |
| France | 2007 to 2011 | 1.05 (0.94, 1.18) |
| France | 2012 to 2016 | 1.02 (0.91, 1.15) |
| France | 2017 to 2021 | 1.02 (0.91, 1.14) |
| Gabon | 1992 to 1996 | 1.08 (0.02, 54.6) |
| Gabon | 1997 to 2001 | 1.24 (0.03, 45.52) |
| Gabon | 2002 to 2006 | 1.00 (1.00, 1.00) |
| Gabon | 2007 to 2011 | 0.96 (0.03, 29.19) |
| Gabon | 2012 to 2016 | 0.95 (0.03, 26.36) |
| Gabon | 2017 to 2021 | 1.73 (0.09, 33.05) |
| Georgia | 1992 to 1996 | 1.45 (0.55, 3.82) |
| Georgia | 1997 to 2001 | 1.51 (0.61, 3.76) |
| Georgia | 2002 to 2006 | 1.00 (1.00, 1.00) |
| Georgia | 2007 to 2011 | 1.39 (0.54, 3.61) |
| Georgia | 2012 to 2016 | 1.4 (0.52, 3.74) |
| Georgia | 2017 to 2021 | 1.6 (0.59, 4.32) |
| Germany | 1992 to 1996 | 0.92 (0.77, 1.11) |
| Germany | 1997 to 2001 | 1.13 (0.96, 1.31) |
| Germany | 2002 to 2006 | 1.00 (1.00, 1.00) |
| Germany | 2007 to 2011 | 0.85 (0.72, 1.01) |
| Germany | 2012 to 2016 | 0.71 (0.6, 0.85) |
| Germany | 2017 to 2021 | 0.67 (0.56, 0.8) |
| Ghana | 1992 to 1996 | 2.56 (0.47, 14.06) |
| Ghana | 1997 to 2001 | 3.48 (0.78, 15.5) |
| Ghana | 2002 to 2006 | 1.00 (1.00, 1.00) |
| Ghana | 2007 to 2011 | 0.57 (0.06, 5.13) |
| Ghana | 2012 to 2016 | 0.7 (0.11, 4.32) |
| Ghana | 2017 to 2021 | 0.63 (0.11, 3.53) |
| Greece | 1992 to 1996 | 0.99 (0.73, 1.36) |
| Greece | 1997 to 2001 | 1.02 (0.77, 1.34) |
| Greece | 2002 to 2006 | 1.00 (1.00, 1.00) |
| Greece | 2007 to 2011 | 1.04 (0.8, 1.37) |
| Greece | 2012 to 2016 | 0.84 (0.62, 1.13) |
| Greece | 2017 to 2021 | 0.8 (0.58, 1.09) |
| Greenland | 1992 to 1996 | 2.61 (0.07, 92.7) |
| Greenland | 1997 to 2001 | 2.2 (0.18, 26.31) |
| Greenland | 2002 to 2006 | 1.00 (1.00, 1.00) |
| Greenland | 2007 to 2011 | 1.65 (0.06, 43.24) |
| Greenland | 2012 to 2016 | 1.56 (0.07, 37.09) |
| Greenland | 2017 to 2021 | 1.43 (0.07, 28.74) |
| Guam | 1992 to 1996 | 0.79 (0.02, 38.49) |
| Guam | 1997 to 2001 | 0.77 (0.02, 27.63) |
| Guam | 2002 to 2006 | 1.00 (1.00, 1.00) |
| Guam | 2007 to 2011 | 0.83 (0.03, 24.49) |
| Guam | 2012 to 2016 | 0.84 (0.03, 22.13) |
| Guam | 2017 to 2021 | 0.87 (0.04, 19.72) |
| Guatemala | 1992 to 1996 | 0.83 (0.34, 2.04) |
| Guatemala | 1997 to 2001 | 0.89 (0.4, 1.94) |
| Guatemala | 2002 to 2006 | 1.00 (1.00, 1.00) |
| Guatemala | 2007 to 2011 | 0.94 (0.46, 1.92) |
| Guatemala | 2012 to 2016 | 0.84 (0.41, 1.7) |
| Guatemala | 2017 to 2021 | 0.75 (0.36, 1.54) |
| Guinea | 1992 to 1996 | 0.88 (0.26, 2.96) |
| Guinea | 1997 to 2001 | 0.98 (0.33, 2.86) |
| Guinea | 2002 to 2006 | 1.00 (1.00, 1.00) |
| Guinea | 2007 to 2011 | 0.9 (0.32, 2.48) |
| Guinea | 2012 to 2016 | 1.1 (0.42, 2.88) |
| Guinea | 2017 to 2021 | 0.99 (0.38, 2.54) |
| Guinea-Bissau | 1992 to 1996 | 1.93 (0.03, 127.87) |
| Guinea-Bissau | 1997 to 2001 | 1.4 (0.02, 78.16) |
| Guinea-Bissau | 2002 to 2006 | 1.00 (1.00, 1.00) |
| Guinea-Bissau | 2007 to 2011 | 0.88 (0.02, 49.33) |
| Guinea-Bissau | 2012 to 2016 | 0.84 (0.01, 50.03) |
| Guinea-Bissau | 2017 to 2021 | 3.15 (0.11, 90.79) |
| Haiti | 1992 to 1996 | 0.87 (0.38, 2.01) |
| Haiti | 1997 to 2001 | 0.97 (0.46, 2.04) |
| Haiti | 2002 to 2006 | 1.00 (1.00, 1.00) |
| Haiti | 2007 to 2011 | 0.96 (0.49, 1.89) |
| Haiti | 2012 to 2016 | 0.97 (0.5, 1.88) |
| Haiti | 2017 to 2021 | 1.02 (0.53, 1.96) |
| Honduras | 1992 to 1996 | 1.36 (0.37, 5.01) |
| Honduras | 1997 to 2001 | 1.04 (0.3, 3.61) |
| Honduras | 2002 to 2006 | 1.00 (1.00, 1.00) |
| Honduras | 2007 to 2011 | 0.89 (0.27, 2.96) |
| Honduras | 2012 to 2016 | 0.79 (0.24, 2.65) |
| Honduras | 2017 to 2021 | 0.7 (0.21, 2.36) |
| Hungary | 1992 to 1996 | 0.67 (0.37, 1.23) |
| Hungary | 1997 to 2001 | 0.86 (0.52, 1.42) |
| Hungary | 2002 to 2006 | 1.00 (1.00, 1.00) |
| Hungary | 2007 to 2011 | 0.99 (0.62, 1.58) |
| Hungary | 2012 to 2016 | 0.87 (0.53, 1.42) |
| Hungary | 2017 to 2021 | 0.85 (0.52, 1.41) |
| Iceland | 1992 to 1996 | 0.8 (0.02, 39.03) |
| Iceland | 1997 to 2001 | 1.09 (0.03, 39.81) |
| Iceland | 2002 to 2006 | 1.00 (1.00, 1.00) |
| Iceland | 2007 to 2011 | 1.54 (0.06, 37.01) |
| Iceland | 2012 to 2016 | 1.27 (0.07, 23.93) |
| Iceland | 2017 to 2021 | 1.08 (0.07, 17.59) |
| India | 1992 to 1996 | 1.07 (1.02, 1.12) |
| India | 1997 to 2001 | 1.12 (1.07, 1.16) |
| India | 2002 to 2006 | 1.00 (1.00, 1.00) |
| India | 2007 to 2011 | 1.01 (0.97, 1.05) |
| India | 2012 to 2016 | 1.01 (0.97, 1.04) |
| India | 2017 to 2021 | 1.04 (1, 1.08) |
| Indonesia | 1992 to 1996 | 1.04 (0.96, 1.13) |
| Indonesia | 1997 to 2001 | 1.04 (0.96, 1.11) |
| Indonesia | 2002 to 2006 | 1.00 (1.00, 1.00) |
| Indonesia | 2007 to 2011 | 1 (0.93, 1.07) |
| Indonesia | 2012 to 2016 | 1 (0.94, 1.07) |
| Indonesia | 2017 to 2021 | 1.02 (0.96, 1.09) |
| Iran (Islamic Republic of) | 1992 to 1996 | 0.78 (0.56, 1.08) |
| Iran (Islamic Republic of) | 1997 to 2001 | 0.9 (0.68, 1.2) |
| Iran (Islamic Republic of) | 2002 to 2006 | 1.00 (1.00, 1.00) |
| Iran (Islamic Republic of) | 2007 to 2011 | 1.01 (0.78, 1.3) |
| Iran (Islamic Republic of) | 2012 to 2016 | 1.04 (0.81, 1.35) |
| Iran (Islamic Republic of) | 2017 to 2021 | 1.11 (0.87, 1.43) |
| Iraq | 1992 to 1996 | 0.96 (0.64, 1.45) |
| Iraq | 1997 to 2001 | 1 (0.71, 1.42) |
| Iraq | 2002 to 2006 | 1.00 (1.00, 1.00) |
| Iraq | 2007 to 2011 | 0.95 (0.68, 1.31) |
| Iraq | 2012 to 2016 | 0.88 (0.63, 1.21) |
| Iraq | 2017 to 2021 | 0.9 (0.66, 1.23) |
| Ireland | 1992 to 1996 | 0.74 (0.34, 1.58) |
| Ireland | 1997 to 2001 | 0.86 (0.45, 1.63) |
| Ireland | 2002 to 2006 | 1.00 (1.00, 1.00) |
| Ireland | 2007 to 2011 | 1.16 (0.67, 2.02) |
| Ireland | 2012 to 2016 | 1.04 (0.59, 1.86) |
| Ireland | 2017 to 2021 | 0.93 (0.51, 1.67) |
| Israel | 1992 to 1996 | 0.77 (0.41, 1.45) |
| Israel | 1997 to 2001 | 1.03 (0.61, 1.74) |
| Israel | 2002 to 2006 | 1.00 (1.00, 1.00) |
| Israel | 2007 to 2011 | 0.93 (0.57, 1.52) |
| Israel | 2012 to 2016 | 0.85 (0.51, 1.4) |
| Israel | 2017 to 2021 | 0.83 (0.51, 1.37) |
| Italy | 1992 to 1996 | 0.94 (0.81, 1.09) |
| Italy | 1997 to 2001 | 1.12 (0.99, 1.27) |
| Italy | 2002 to 2006 | 1.00 (1.00, 1.00) |
| Italy | 2007 to 2011 | 0.96 (0.84, 1.09) |
| Italy | 2012 to 2016 | 0.87 (0.76, 1) |
| Italy | 2017 to 2021 | 0.75 (0.65, 0.87) |
| Jamaica | 1992 to 1996 | 1.08 (0.24, 4.78) |
| Jamaica | 1997 to 2001 | 1.2 (0.34, 4.25) |
| Jamaica | 2002 to 2006 | 1.00 (1.00, 1.00) |
| Jamaica | 2007 to 2011 | 1.14 (0.33, 3.88) |
| Jamaica | 2012 to 2016 | 1.36 (0.41, 4.51) |
| Jamaica | 2017 to 2021 | 1.28 (0.38, 4.29) |
| Japan | 1992 to 1996 | 0.69 (0.58, 0.82) |
| Japan | 1997 to 2001 | 0.91 (0.79, 1.06) |
| Japan | 2002 to 2006 | 1.00 (1.00, 1.00) |
| Japan | 2007 to 2011 | 1.12 (0.98, 1.29) |
| Japan | 2012 to 2016 | 1.06 (0.92, 1.23) |
| Japan | 2017 to 2021 | 0.97 (0.83, 1.13) |
| Jordan | 1992 to 1996 | 0.91 (0.5, 1.67) |
| Jordan | 1997 to 2001 | 0.97 (0.57, 1.67) |
| Jordan | 2002 to 2006 | 1.00 (1.00, 1.00) |
| Jordan | 2007 to 2011 | 0.99 (0.61, 1.6) |
| Jordan | 2012 to 2016 | 1.02 (0.64, 1.62) |
| Jordan | 2017 to 2021 | 0.97 (0.62, 1.52) |
| Kazakhstan | 1992 to 1996 | 1.08 (0.69, 1.7) |
| Kazakhstan | 1997 to 2001 | 0.9 (0.58, 1.42) |
| Kazakhstan | 2002 to 2006 | 1.00 (1.00, 1.00) |
| Kazakhstan | 2007 to 2011 | 1.13 (0.75, 1.71) |
| Kazakhstan | 2012 to 2016 | 1.32 (0.89, 1.97) |
| Kazakhstan | 2017 to 2021 | 1.26 (0.84, 1.89) |
| Kenya | 1992 to 1996 | 0.93 (0.74, 1.17) |
| Kenya | 1997 to 2001 | 0.9 (0.73, 1.1) |
| Kenya | 2002 to 2006 | 1.00 (1.00, 1.00) |
| Kenya | 2007 to 2011 | 1.03 (0.86, 1.23) |
| Kenya | 2012 to 2016 | 1.06 (0.89, 1.27) |
| Kenya | 2017 to 2021 | 1.08 (0.91, 1.28) |
| Kuwait | 1992 to 1996 | 1.02 (0.42, 2.47) |
| Kuwait | 1997 to 2001 | 0.96 (0.44, 2.08) |
| Kuwait | 2002 to 2006 | 1.00 (1.00, 1.00) |
| Kuwait | 2007 to 2011 | 0.82 (0.4, 1.67) |
| Kuwait | 2012 to 2016 | 0.74 (0.37, 1.51) |
| Kuwait | 2017 to 2021 | 0.76 (0.38, 1.5) |
| Kyrgyzstan | 1992 to 1996 | 0.88 (0.32, 2.44) |
| Kyrgyzstan | 1997 to 2001 | 1.08 (0.45, 2.59) |
| Kyrgyzstan | 2002 to 2006 | 1.00 (1.00, 1.00) |
| Kyrgyzstan | 2007 to 2011 | 1.19 (0.52, 2.71) |
| Kyrgyzstan | 2012 to 2016 | 1.47 (0.67, 3.2) |
| Kyrgyzstan | 2017 to 2021 | 1.65 (0.77, 3.55) |
| Lao People's Democratic Republic | 1992 to 1996 | 1.09 (0.62, 1.93) |
| Lao People's Democratic Republic | 1997 to 2001 | 1.08 (0.66, 1.78) |
| Lao People's Democratic Republic | 2002 to 2006 | 1.00 (1.00, 1.00) |
| Lao People's Democratic Republic | 2007 to 2011 | 1.01 (0.63, 1.6) |
| Lao People's Democratic Republic | 2012 to 2016 | 0.99 (0.63, 1.58) |
| Lao People's Democratic Republic | 2017 to 2021 | 1.06 (0.68, 1.65) |
| Latvia | 1992 to 1996 | 1.68 (0.4, 7) |
| Latvia | 1997 to 2001 | 1.11 (0.32, 3.84) |
| Latvia | 2002 to 2006 | 1.00 (1.00, 1.00) |
| Latvia | 2007 to 2011 | 0.72 (0.17, 3.02) |
| Latvia | 2012 to 2016 | 0.39 (0.07, 2.22) |
| Latvia | 2017 to 2021 | 0.37 (0.07, 2.02) |
| Lebanon | 1992 to 1996 | 0.79 (0.26, 2.34) |
| Lebanon | 1997 to 2001 | 1.07 (0.43, 2.62) |
| Lebanon | 2002 to 2006 | 1.00 (1.00, 1.00) |
| Lebanon | 2007 to 2011 | 1.22 (0.53, 2.79) |
| Lebanon | 2012 to 2016 | 1.32 (0.6, 2.92) |
| Lebanon | 2017 to 2021 | 1.33 (0.62, 2.86) |
| Lesotho | 1992 to 1996 | 0.22 (0.01, 7.22) |
| Lesotho | 1997 to 2001 | 0.35 (0.01, 8.76) |
| Lesotho | 2002 to 2006 | 1.00 (1.00, 1.00) |
| Lesotho | 2007 to 2011 | 1.71 (0.22, 13.26) |
| Lesotho | 2012 to 2016 | 1.3 (0.17, 9.68) |
| Lesotho | 2017 to 2021 | 1.03 (0.13, 8.34) |
| Liberia | 1992 to 1996 | 2.28 (0.04, 143.41) |
| Liberia | 1997 to 2001 | 1.42 (0.03, 78.06) |
| Liberia | 2002 to 2006 | 1.00 (1.00, 1.00) |
| Liberia | 2007 to 2011 | 4.21 (0.18, 98.45) |
| Liberia | 2012 to 2016 | 3.83 (0.15, 96.1) |
| Liberia | 2017 to 2021 | 3.59 (0.14, 90.76) |
| Libya | 1992 to 1996 | 0.81 (0.58, 1.14) |
| Libya | 1997 to 2001 | 0.86 (0.64, 1.14) |
| Libya | 2002 to 2006 | 1.00 (1.00, 1.00) |
| Libya | 2007 to 2011 | 1.21 (0.95, 1.54) |
| Libya | 2012 to 2016 | 1.25 (0.98, 1.59) |
| Libya | 2017 to 2021 | 1.19 (0.94, 1.52) |
| Lithuania | 1992 to 1996 | 1.1 (0.33, 3.62) |
| Lithuania | 1997 to 2001 | 0.89 (0.31, 2.56) |
| Lithuania | 2002 to 2006 | 1.00 (1.00, 1.00) |
| Lithuania | 2007 to 2011 | 1.05 (0.37, 3.02) |
| Lithuania | 2012 to 2016 | 0.82 (0.25, 2.72) |
| Lithuania | 2017 to 2021 | 0.41 (0.09, 1.96) |
| Luxembourg | 1992 to 1996 | 0.91 (0.12, 6.78) |
| Luxembourg | 1997 to 2001 | 0.83 (0.16, 4.25) |
| Luxembourg | 2002 to 2006 | 1.00 (1.00, 1.00) |
| Luxembourg | 2007 to 2011 | 0.75 (0.15, 3.78) |
| Luxembourg | 2012 to 2016 | 0.53 (0.08, 3.54) |
| Luxembourg | 2017 to 2021 | 0.47 (0.06, 3.47) |
| Madagascar | 1992 to 1996 | 1.06 (0.72, 1.57) |
| Madagascar | 1997 to 2001 | 1.07 (0.76, 1.52) |
| Madagascar | 2002 to 2006 | 1.00 (1.00, 1.00) |
| Madagascar | 2007 to 2011 | 1 (0.73, 1.39) |
| Madagascar | 2012 to 2016 | 1.01 (0.74, 1.39) |
| Madagascar | 2017 to 2021 | 1.03 (0.76, 1.4) |
| Malawi | 1992 to 1996 | 1.09 (0.45, 2.65) |
| Malawi | 1997 to 2001 | 1.17 (0.52, 2.64) |
| Malawi | 2002 to 2006 | 1.00 (1.00, 1.00) |
| Malawi | 2007 to 2011 | 1.12 (0.52, 2.39) |
| Malawi | 2012 to 2016 | 1.06 (0.5, 2.25) |
| Malawi | 2017 to 2021 | 1.16 (0.57, 2.39) |
| Malaysia | 1992 to 1996 | 1.13 (1.01, 1.27) |
| Malaysia | 1997 to 2001 | 1.09 (0.99, 1.2) |
| Malaysia | 2002 to 2006 | 1.00 (1.00, 1.00) |
| Malaysia | 2007 to 2011 | 1 (0.91, 1.1) |
| Malaysia | 2012 to 2016 | 1.17 (1.06, 1.28) |
| Malaysia | 2017 to 2021 | 1.51 (1.39, 1.65) |
| Maldives | 1992 to 1996 | 1.89 (0.03, 124.53) |
| Maldives | 1997 to 2001 | 1.39 (0.02, 77.83) |
| Maldives | 2002 to 2006 | 1.00 (1.00, 1.00) |
| Maldives | 2007 to 2011 | 0.83 (0.01, 46.4) |
| Maldives | 2012 to 2016 | 1.48 (0.04, 59.52) |
| Maldives | 2017 to 2021 | 2.21 (0.08, 63.65) |
| Mali | 1992 to 1996 | 1.32 (0.36, 4.82) |
| Mali | 1997 to 2001 | 1.05 (0.31, 3.62) |
| Mali | 2002 to 2006 | 1.00 (1.00, 1.00) |
| Mali | 2007 to 2011 | 1.02 (0.32, 3.28) |
| Mali | 2012 to 2016 | 0.98 (0.32, 3.07) |
| Mali | 2017 to 2021 | 1.25 (0.43, 3.67) |
| Malta | 1992 to 1996 | 1.05 (0.29, 3.86) |
| Malta | 1997 to 2001 | 0.96 (0.29, 3.16) |
| Malta | 2002 to 2006 | 1.00 (1.00, 1.00) |
| Malta | 2007 to 2011 | 1.13 (0.39, 3.25) |
| Malta | 2012 to 2016 | 0.96 (0.31, 3.03) |
| Malta | 2017 to 2021 | 0.92 (0.28, 3.05) |
| Mauritania | 1992 to 1996 | 1.72 (0.03, 111) |
| Mauritania | 1997 to 2001 | 1.26 (0.02, 70.21) |
| Mauritania | 2002 to 2006 | 1.00 (1.00, 1.00) |
| Mauritania | 2007 to 2011 | 0.99 (0.02, 55.22) |
| Mauritania | 2012 to 2016 | 3.27 (0.11, 99.09) |
| Mauritania | 2017 to 2021 | 4.24 (0.17, 108.94) |
| Mauritius | 1992 to 1996 | 0.84 (0.23, 3.12) |
| Mauritius | 1997 to 2001 | 0.93 (0.31, 2.84) |
| Mauritius | 2002 to 2006 | 1.00 (1.00, 1.00) |
| Mauritius | 2007 to 2011 | 1.27 (0.44, 3.67) |
| Mauritius | 2012 to 2016 | 1.28 (0.44, 3.74) |
| Mauritius | 2017 to 2021 | 1.55 (0.57, 4.22) |
| Mexico | 1992 to 1996 | 0.92 (0.64, 1.33) |
| Mexico | 1997 to 2001 | 1.02 (0.73, 1.41) |
| Mexico | 2002 to 2006 | 1.00 (1.00, 1.00) |
| Mexico | 2007 to 2011 | 1.09 (0.8, 1.49) |
| Mexico | 2012 to 2016 | 1.11 (0.81, 1.51) |
| Mexico | 2017 to 2021 | 1.26 (0.94, 1.7) |
| Mongolia | 1992 to 1996 | 1.27 (0.33, 4.8) |
| Mongolia | 1997 to 2001 | 1.12 (0.31, 4.01) |
| Mongolia | 2002 to 2006 | 1.00 (1.00, 1.00) |
| Mongolia | 2007 to 2011 | 1.11 (0.32, 3.77) |
| Mongolia | 2012 to 2016 | 1.5 (0.47, 4.78) |
| Mongolia | 2017 to 2021 | 1.47 (0.45, 4.79) |
| Morocco | 1992 to 1996 | 0.96 (0.74, 1.25) |
| Morocco | 1997 to 2001 | 0.97 (0.77, 1.22) |
| Morocco | 2002 to 2006 | 1.00 (1.00, 1.00) |
| Morocco | 2007 to 2011 | 1.02 (0.82, 1.27) |
| Morocco | 2012 to 2016 | 0.97 (0.78, 1.22) |
| Morocco | 2017 to 2021 | 0.92 (0.74, 1.14) |
| Mozambique | 1992 to 1996 | 0.51 (0.02, 14.15) |
| Mozambique | 1997 to 2001 | 1.3 (0.15, 11.47) |
| Mozambique | 2002 to 2006 | 1.00 (1.00, 1.00) |
| Mozambique | 2007 to 2011 | 1.57 (0.25, 9.86) |
| Mozambique | 2012 to 2016 | 1.63 (0.22, 11.86) |
| Mozambique | 2017 to 2021 | 1.75 (0.29, 10.43) |
| Myanmar | 1992 to 1996 | 1.13 (0.95, 1.33) |
| Myanmar | 1997 to 2001 | 1.09 (0.94, 1.27) |
| Myanmar | 2002 to 2006 | 1.00 (1.00, 1.00) |
| Myanmar | 2007 to 2011 | 0.9 (0.77, 1.04) |
| Myanmar | 2012 to 2016 | 0.87 (0.75, 1.01) |
| Myanmar | 2017 to 2021 | 0.94 (0.81, 1.09) |
| Namibia | 1992 to 1996 | 1.18 (0.32, 4.35) |
| Namibia | 1997 to 2001 | 1.07 (0.31, 3.64) |
| Namibia | 2002 to 2006 | 1.00 (1.00, 1.00) |
| Namibia | 2007 to 2011 | 0.94 (0.28, 3.13) |
| Namibia | 2012 to 2016 | 0.87 (0.26, 2.91) |
| Namibia | 2017 to 2021 | 0.8 (0.24, 2.69) |
| Nepal | 1992 to 1996 | 1.14 (0.83, 1.58) |
| Nepal | 1997 to 2001 | 1.06 (0.79, 1.42) |
| Nepal | 2002 to 2006 | 1.00 (1.00, 1.00) |
| Nepal | 2007 to 2011 | 1.06 (0.8, 1.4) |
| Nepal | 2012 to 2016 | 1.12 (0.86, 1.47) |
| Nepal | 2017 to 2021 | 1.16 (0.89, 1.51) |
| Netherlands | 1992 to 1996 | 0.96 (0.73, 1.27) |
| Netherlands | 1997 to 2001 | 0.99 (0.78, 1.26) |
| Netherlands | 2002 to 2006 | 1.00 (1.00, 1.00) |
| Netherlands | 2007 to 2011 | 0.96 (0.75, 1.22) |
| Netherlands | 2012 to 2016 | 0.86 (0.66, 1.11) |
| Netherlands | 2017 to 2021 | 0.9 (0.7, 1.15) |
| New Zealand | 1992 to 1996 | 1.22 (0.73, 2.03) |
| New Zealand | 1997 to 2001 | 1.14 (0.75, 1.74) |
| New Zealand | 2002 to 2006 | 1.00 (1.00, 1.00) |
| New Zealand | 2007 to 2011 | 0.95 (0.62, 1.45) |
| New Zealand | 2012 to 2016 | 0.78 (0.49, 1.24) |
| New Zealand | 2017 to 2021 | 0.67 (0.42, 1.07) |
| Nicaragua | 1992 to 1996 | 1.33 (0.36, 4.87) |
| Nicaragua | 1997 to 2001 | 1.02 (0.29, 3.52) |
| Nicaragua | 2002 to 2006 | 1.00 (1.00, 1.00) |
| Nicaragua | 2007 to 2011 | 0.94 (0.28, 3.14) |
| Nicaragua | 2012 to 2016 | 1.04 (0.32, 3.35) |
| Nicaragua | 2017 to 2021 | 1.08 (0.35, 3.35) |
| Niger | 1992 to 1996 | 1.02 (0.17, 6.05) |
| Niger | 1997 to 2001 | 0.75 (0.17, 3.4) |
| Niger | 2002 to 2006 | 1.00 (1.00, 1.00) |
| Niger | 2007 to 2011 | 1.08 (0.29, 3.94) |
| Niger | 2012 to 2016 | 1.18 (0.33, 4.22) |
| Niger | 2017 to 2021 | 1.2 (0.36, 3.99) |
| Nigeria | 1992 to 1996 | 1.06 (0.89, 1.26) |
| Nigeria | 1997 to 2001 | 1.05 (0.91, 1.22) |
| Nigeria | 2002 to 2006 | 1.00 (1.00, 1.00) |
| Nigeria | 2007 to 2011 | 1 (0.87, 1.15) |
| Nigeria | 2012 to 2016 | 1.14 (1, 1.31) |
| Nigeria | 2017 to 2021 | 1.16 (1.02, 1.33) |
| North Macedonia | 1992 to 1996 | 0.48 (0.04, 5.29) |
| North Macedonia | 1997 to 2001 | 0.63 (0.13, 2.95) |
| North Macedonia | 2002 to 2006 | 1.00 (1.00, 1.00) |
| North Macedonia | 2007 to 2011 | 0.91 (0.26, 3.16) |
| North Macedonia | 2012 to 2016 | 0.89 (0.24, 3.27) |
| North Macedonia | 2017 to 2021 | 0.66 (0.15, 2.9) |
| Norway | 1992 to 1996 | 0.78 (0.34, 1.79) |
| Norway | 1997 to 2001 | 1.01 (0.48, 2.11) |
| Norway | 2002 to 2006 | 1.00 (1.00, 1.00) |
| Norway | 2007 to 2011 | 0.91 (0.43, 1.93) |
| Norway | 2012 to 2016 | 0.79 (0.36, 1.7) |
| Norway | 2017 to 2021 | 0.73 (0.32, 1.65) |
| Oman | 1992 to 1996 | 1.07 (0.31, 3.69) |
| Oman | 1997 to 2001 | 1.01 (0.31, 3.28) |
| Oman | 2002 to 2006 | 1.00 (1.00, 1.00) |
| Oman | 2007 to 2011 | 0.89 (0.31, 2.56) |
| Oman | 2012 to 2016 | 1.19 (0.45, 3.18) |
| Oman | 2017 to 2021 | 1.26 (0.5, 3.21) |
| Pakistan | 1992 to 1996 | 0.89 (0.79, 1) |
| Pakistan | 1997 to 2001 | 0.96 (0.87, 1.06) |
| Pakistan | 2002 to 2006 | 1.00 (1.00, 1.00) |
| Pakistan | 2007 to 2011 | 0.98 (0.9, 1.08) |
| Pakistan | 2012 to 2016 | 0.98 (0.9, 1.07) |
| Pakistan | 2017 to 2021 | 0.98 (0.9, 1.07) |
| Palestine | 1992 to 1996 | 1.65 (0.44, 6.27) |
| Palestine | 1997 to 2001 | 1.27 (0.35, 4.55) |
| Palestine | 2002 to 2006 | 1.00 (1.00, 1.00) |
| Palestine | 2007 to 2011 | 0.81 (0.23, 2.88) |
| Palestine | 2012 to 2016 | 1.02 (0.31, 3.4) |
| Palestine | 2017 to 2021 | 1.08 (0.33, 3.53) |
| Panama | 1992 to 1996 | 0.95 (0.27, 3.35) |
| Panama | 1997 to 2001 | 0.84 (0.25, 2.78) |
| Panama | 2002 to 2006 | 1.00 (1.00, 1.00) |
| Panama | 2007 to 2011 | 0.92 (0.31, 2.75) |
| Panama | 2012 to 2016 | 1.06 (0.36, 3.15) |
| Panama | 2017 to 2021 | 1.01 (0.34, 3.02) |
| Papua New Guinea | 1992 to 1996 | 1.05 (0.34, 3.3) |
| Papua New Guinea | 1997 to 2001 | 1.08 (0.42, 2.74) |
| Papua New Guinea | 2002 to 2006 | 1.00 (1.00, 1.00) |
| Papua New Guinea | 2007 to 2011 | 1 (0.42, 2.36) |
| Papua New Guinea | 2012 to 2016 | 1.02 (0.41, 2.56) |
| Papua New Guinea | 2017 to 2021 | 0.95 (0.37, 2.47) |
| Paraguay | 1992 to 1996 | 1.03 (0.23, 4.61) |
| Paraguay | 1997 to 2001 | 1.1 (0.31, 3.93) |
| Paraguay | 2002 to 2006 | 1.00 (1.00, 1.00) |
| Paraguay | 2007 to 2011 | 1.21 (0.36, 4.13) |
| Paraguay | 2012 to 2016 | 1.22 (0.37, 4.02) |
| Paraguay | 2017 to 2021 | 1.27 (0.39, 4.15) |
| Peru | 1992 to 1996 | 0.79 (0.35, 1.79) |
| Peru | 1997 to 2001 | 0.82 (0.39, 1.72) |
| Peru | 2002 to 2006 | 1.00 (1.00, 1.00) |
| Peru | 2007 to 2011 | 0.98 (0.5, 1.92) |
| Peru | 2012 to 2016 | 1.11 (0.58, 2.13) |
| Peru | 2017 to 2021 | 1.25 (0.67, 2.33) |
| Philippines | 1992 to 1996 | 1.04 (0.92, 1.17) |
| Philippines | 1997 to 2001 | 1.01 (0.91, 1.12) |
| Philippines | 2002 to 2006 | 1.00 (1.00, 1.00) |
| Philippines | 2007 to 2011 | 0.94 (0.85, 1.04) |
| Philippines | 2012 to 2016 | 0.95 (0.86, 1.05) |
| Philippines | 2017 to 2021 | 1.01 (0.92, 1.11) |
| Poland | 1992 to 1996 | 0.56 (0.38, 0.83) |
| Poland | 1997 to 2001 | 0.84 (0.61, 1.15) |
| Poland | 2002 to 2006 | 1.00 (1.00, 1.00) |
| Poland | 2007 to 2011 | 1.08 (0.81, 1.43) |
| Poland | 2012 to 2016 | 1.03 (0.77, 1.38) |
| Poland | 2017 to 2021 | 0.98 (0.73, 1.31) |
| Portugal | 1992 to 1996 | 1 (0.67, 1.5) |
| Portugal | 1997 to 2001 | 1.14 (0.81, 1.61) |
| Portugal | 2002 to 2006 | 1.00 (1.00, 1.00) |
| Portugal | 2007 to 2011 | 1.07 (0.76, 1.52) |
| Portugal | 2012 to 2016 | 0.99 (0.69, 1.44) |
| Portugal | 2017 to 2021 | 0.95 (0.65, 1.38) |
| Puerto Rico | 1992 to 1996 | 0.84 (0.37, 1.9) |
| Puerto Rico | 1997 to 2001 | 0.93 (0.44, 1.94) |
| Puerto Rico | 2002 to 2006 | 1.00 (1.00, 1.00) |
| Puerto Rico | 2007 to 2011 | 1.16 (0.58, 2.32) |
| Puerto Rico | 2012 to 2016 | 1.08 (0.52, 2.24) |
| Puerto Rico | 2017 to 2021 | 1.13 (0.53, 2.38) |
| Qatar | 1992 to 1996 | 0.7 (0.06, 8.16) |
| Qatar | 1997 to 2001 | 0.81 (0.11, 5.9) |
| Qatar | 2002 to 2006 | 1.00 (1.00, 1.00) |
| Qatar | 2007 to 2011 | 1.3 (0.33, 5.07) |
| Qatar | 2012 to 2016 | 1.14 (0.3, 4.36) |
| Qatar | 2017 to 2021 | 1.14 (0.29, 4.41) |
| Republic of Korea | 1992 to 1996 | 0.8 (0.61, 1.06) |
| Republic of Korea | 1997 to 2001 | 0.9 (0.7, 1.14) |
| Republic of Korea | 2002 to 2006 | 1.00 (1.00, 1.00) |
| Republic of Korea | 2007 to 2011 | 1.32 (1.06, 1.64) |
| Republic of Korea | 2012 to 2016 | 1.21 (0.96, 1.53) |
| Republic of Korea | 2017 to 2021 | 1.21 (0.96, 1.52) |
| Republic of Moldova | 1992 to 1996 | 1.45 (0.54, 3.9) |
| Republic of Moldova | 1997 to 2001 | 1.13 (0.44, 2.95) |
| Republic of Moldova | 2002 to 2006 | 1.00 (1.00, 1.00) |
| Republic of Moldova | 2007 to 2011 | 1.2 (0.47, 3.04) |
| Republic of Moldova | 2012 to 2016 | 1.19 (0.45, 3.12) |
| Republic of Moldova | 2017 to 2021 | 1.11 (0.42, 2.93) |
| Romania | 1992 to 1996 | 0.58 (0.38, 0.88) |
| Romania | 1997 to 2001 | 0.78 (0.55, 1.11) |
| Romania | 2002 to 2006 | 1.00 (1.00, 1.00) |
| Romania | 2007 to 2011 | 1.18 (0.86, 1.61) |
| Romania | 2012 to 2016 | 1.23 (0.89, 1.71) |
| Romania | 2017 to 2021 | 1.18 (0.85, 1.65) |
| Russian Federation | 1992 to 1996 | 1.06 (0.84, 1.33) |
| Russian Federation | 1997 to 2001 | 0.91 (0.74, 1.13) |
| Russian Federation | 2002 to 2006 | 1.00 (1.00, 1.00) |
| Russian Federation | 2007 to 2011 | 1.11 (0.91, 1.35) |
| Russian Federation | 2012 to 2016 | 1.08 (0.88, 1.32) |
| Russian Federation | 2017 to 2021 | 1.08 (0.89, 1.32) |
| Rwanda | 1992 to 1996 | 1.28 (0.82, 2.01) |
| Rwanda | 1997 to 2001 | 1.26 (0.84, 1.89) |
| Rwanda | 2002 to 2006 | 1.00 (1.00, 1.00) |
| Rwanda | 2007 to 2011 | 0.91 (0.61, 1.35) |
| Rwanda | 2012 to 2016 | 0.88 (0.6, 1.29) |
| Rwanda | 2017 to 2021 | 0.88 (0.61, 1.27) |
| Samoa | 1992 to 1996 | 1.03 (0.02, 51.03) |
| Samoa | 1997 to 2001 | 1 (0.03, 37.33) |
| Samoa | 2002 to 2006 | 1.00 (1.00, 1.00) |
| Samoa | 2007 to 2011 | 0.99 (0.03, 29.98) |
| Samoa | 2012 to 2016 | 0.96 (0.04, 26.26) |
| Samoa | 2017 to 2021 | 0.89 (0.04, 21.16) |
| Saudi Arabia | 1992 to 1996 | 0.92 (0.7, 1.22) |
| Saudi Arabia | 1997 to 2001 | 0.94 (0.74, 1.2) |
| Saudi Arabia | 2002 to 2006 | 1.00 (1.00, 1.00) |
| Saudi Arabia | 2007 to 2011 | 1.16 (0.95, 1.43) |
| Saudi Arabia | 2012 to 2016 | 1.34 (1.1, 1.63) |
| Saudi Arabia | 2017 to 2021 | 1.53 (1.27, 1.84) |
| Senegal | 1992 to 1996 | 0.57 (0.11, 2.92) |
| Senegal | 1997 to 2001 | 0.66 (0.19, 2.36) |
| Senegal | 2002 to 2006 | 1.00 (1.00, 1.00) |
| Senegal | 2007 to 2011 | 0.87 (0.31, 2.45) |
| Senegal | 2012 to 2016 | 0.99 (0.36, 2.73) |
| Senegal | 2017 to 2021 | 0.93 (0.34, 2.55) |
| Serbia | 1992 to 1996 | 0.83 (0.36, 1.89) |
| Serbia | 1997 to 2001 | 0.87 (0.42, 1.8) |
| Serbia | 2002 to 2006 | 1.00 (1.00, 1.00) |
| Serbia | 2007 to 2011 | 1.09 (0.55, 2.16) |
| Serbia | 2012 to 2016 | 0.99 (0.49, 2) |
| Serbia | 2017 to 2021 | 1.03 (0.51, 2.05) |
| Seychelles | 1992 to 1996 | 0.92 (0.02, 46.77) |
| Seychelles | 1997 to 2001 | 0.98 (0.03, 36.5) |
| Seychelles | 2002 to 2006 | 1.00 (1.00, 1.00) |
| Seychelles | 2007 to 2011 | 1.04 (0.03, 31.61) |
| Seychelles | 2012 to 2016 | 1.09 (0.04, 30.4) |
| Seychelles | 2017 to 2021 | 1.14 (0.05, 28.25) |
| Sierra Leone | 1992 to 1996 | 0.34 (0.01, 8.72) |
| Sierra Leone | 1997 to 2001 | 1.01 (0.12, 8.53) |
| Sierra Leone | 2002 to 2006 | 1.00 (1.00, 1.00) |
| Sierra Leone | 2007 to 2011 | 0.79 (0.14, 4.6) |
| Sierra Leone | 2012 to 2016 | 0.75 (0.13, 4.11) |
| Sierra Leone | 2017 to 2021 | 0.85 (0.17, 4.25) |
| Singapore | 1992 to 1996 | 0.81 (0.62, 1.05) |
| Singapore | 1997 to 2001 | 0.94 (0.76, 1.18) |
| Singapore | 2002 to 2006 | 1.00 (1.00, 1.00) |
| Singapore | 2007 to 2011 | 1.02 (0.83, 1.27) |
| Singapore | 2012 to 2016 | 0.87 (0.7, 1.09) |
| Singapore | 2017 to 2021 | 0.76 (0.61, 0.95) |
| Slovakia | 1992 to 1996 | 0.63 (0.22, 1.83) |
| Slovakia | 1997 to 2001 | 0.92 (0.39, 2.18) |
| Slovakia | 2002 to 2006 | 1.00 (1.00, 1.00) |
| Slovakia | 2007 to 2011 | 1.05 (0.47, 2.33) |
| Slovakia | 2012 to 2016 | 1.03 (0.46, 2.33) |
| Slovakia | 2017 to 2021 | 0.99 (0.44, 2.22) |
| Slovenia | 1992 to 1996 | 1.19 (0.32, 4.42) |
| Slovenia | 1997 to 2001 | 1.09 (0.36, 3.26) |
| Slovenia | 2002 to 2006 | 1.00 (1.00, 1.00) |
| Slovenia | 2007 to 2011 | 0.88 (0.3, 2.59) |
| Slovenia | 2012 to 2016 | 0.79 (0.23, 2.64) |
| Slovenia | 2017 to 2021 | 0.49 (0.12, 2.01) |
| Solomon Islands | 1992 to 1996 | 1.11 (0.02, 55.98) |
| Solomon Islands | 1997 to 2001 | 1.05 (0.03, 39.13) |
| Solomon Islands | 2002 to 2006 | 1.00 (1.00, 1.00) |
| Solomon Islands | 2007 to 2011 | 0.95 (0.03, 29) |
| Solomon Islands | 2012 to 2016 | 0.91 (0.03, 25.26) |
| Solomon Islands | 2017 to 2021 | 0.86 (0.03, 21.34) |
| Somalia | 1992 to 1996 | 1.06 (0.63, 1.79) |
| Somalia | 1997 to 2001 | 1.06 (0.67, 1.68) |
| Somalia | 2002 to 2006 | 1.00 (1.00, 1.00) |
| Somalia | 2007 to 2011 | 1.02 (0.68, 1.54) |
| Somalia | 2012 to 2016 | 1 (0.68, 1.48) |
| Somalia | 2017 to 2021 | 1.01 (0.69, 1.49) |
| South Africa | 1992 to 1996 | 0.88 (0.64, 1.22) |
| South Africa | 1997 to 2001 | 0.81 (0.6, 1.08) |
| South Africa | 2002 to 2006 | 1.00 (1.00, 1.00) |
| South Africa | 2007 to 2011 | 0.79 (0.6, 1.04) |
| South Africa | 2012 to 2016 | 0.65 (0.49, 0.87) |
| South Africa | 2017 to 2021 | 0.74 (0.56, 0.97) |
| South Sudan | 1992 to 1996 | 1.18 (0.62, 2.26) |
| South Sudan | 1997 to 2001 | 1.07 (0.59, 1.96) |
| South Sudan | 2002 to 2006 | 1.00 (1.00, 1.00) |
| South Sudan | 2007 to 2011 | 1.14 (0.65, 2) |
| South Sudan | 2012 to 2016 | 1.26 (0.73, 2.17) |
| South Sudan | 2017 to 2021 | 1.32 (0.76, 2.29) |
| Spain | 1992 to 1996 | 1.11 (0.96, 1.29) |
| Spain | 1997 to 2001 | 1.11 (0.97, 1.26) |
| Spain | 2002 to 2006 | 1.00 (1.00, 1.00) |
| Spain | 2007 to 2011 | 0.88 (0.77, 1.01) |
| Spain | 2012 to 2016 | 0.77 (0.66, 0.88) |
| Spain | 2017 to 2021 | 0.77 (0.67, 0.89) |
| Sri Lanka | 1992 to 1996 | 0.86 (0.66, 1.13) |
| Sri Lanka | 1997 to 2001 | 1.02 (0.8, 1.28) |
| Sri Lanka | 2002 to 2006 | 1.00 (1.00, 1.00) |
| Sri Lanka | 2007 to 2011 | 0.96 (0.76, 1.21) |
| Sri Lanka | 2012 to 2016 | 0.94 (0.74, 1.18) |
| Sri Lanka | 2017 to 2021 | 1.1 (0.88, 1.38) |
| Sudan | 1992 to 1996 | 1.06 (0.65, 1.72) |
| Sudan | 1997 to 2001 | 1.08 (0.7, 1.67) |
| Sudan | 2002 to 2006 | 1.00 (1.00, 1.00) |
| Sudan | 2007 to 2011 | 0.96 (0.64, 1.46) |
| Sudan | 2012 to 2016 | 0.91 (0.6, 1.37) |
| Sudan | 2017 to 2021 | 0.92 (0.62, 1.36) |
| Suriname | 1992 to 1996 | 1.86 (0.03, 122.77) |
| Suriname | 1997 to 2001 | 1.21 (0.02, 68.99) |
| Suriname | 2002 to 2006 | 1.00 (1.00, 1.00) |
| Suriname | 2007 to 2011 | 1.72 (0.04, 69.02) |
| Suriname | 2012 to 2016 | 2.07 (0.05, 84.36) |
| Suriname | 2017 to 2021 | 2.41 (0.06, 95.39) |
| Sweden | 1992 to 1996 | 1.07 (0.65, 1.76) |
| Sweden | 1997 to 2001 | 1.21 (0.8, 1.82) |
| Sweden | 2002 to 2006 | 1.00 (1.00, 1.00) |
| Sweden | 2007 to 2011 | 0.93 (0.61, 1.44) |
| Sweden | 2012 to 2016 | 0.87 (0.55, 1.36) |
| Sweden | 2017 to 2021 | 0.9 (0.58, 1.41) |
| Switzerland | 1992 to 1996 | 1.06 (0.63, 1.79) |
| Switzerland | 1997 to 2001 | 1.06 (0.68, 1.66) |
| Switzerland | 2002 to 2006 | 1.00 (1.00, 1.00) |
| Switzerland | 2007 to 2011 | 0.9 (0.57, 1.43) |
| Switzerland | 2012 to 2016 | 0.6 (0.35, 1.01) |
| Switzerland | 2017 to 2021 | 0.4 (0.23, 0.71) |
| Syrian Arab Republic | 1992 to 1996 | 0.91 (0.4, 2.09) |
| Syrian Arab Republic | 1997 to 2001 | 0.92 (0.44, 1.95) |
| Syrian Arab Republic | 2002 to 2006 | 1.00 (1.00, 1.00) |
| Syrian Arab Republic | 2007 to 2011 | 0.94 (0.47, 1.84) |
| Syrian Arab Republic | 2012 to 2016 | 0.96 (0.47, 1.95) |
| Syrian Arab Republic | 2017 to 2021 | 1.09 (0.5, 2.37) |
| Taiwan (Province of China) | 1992 to 1996 | 1.11 (1.03, 1.2) |
| Taiwan (Province of China) | 1997 to 2001 | 1.06 (1, 1.13) |
| Taiwan (Province of China) | 2002 to 2006 | 1.00 (1.00, 1.00) |
| Taiwan (Province of China) | 2007 to 2011 | 0.91 (0.85, 0.97) |
| Taiwan (Province of China) | 2012 to 2016 | 0.83 (0.78, 0.89) |
| Taiwan (Province of China) | 2017 to 2021 | 0.78 (0.72, 0.83) |
| Tajikistan | 1992 to 1996 | 1.44 (0.7, 2.93) |
| Tajikistan | 1997 to 2001 | 1.28 (0.65, 2.52) |
| Tajikistan | 2002 to 2006 | 1.00 (1.00, 1.00) |
| Tajikistan | 2007 to 2011 | 0.93 (0.47, 1.84) |
| Tajikistan | 2012 to 2016 | 1.1 (0.58, 2.11) |
| Tajikistan | 2017 to 2021 | 1.07 (0.56, 2.05) |
| Thailand | 1992 to 1996 | 0.71 (0.64, 0.79) |
| Thailand | 1997 to 2001 | 0.94 (0.87, 1.03) |
| Thailand | 2002 to 2006 | 1.00 (1.00, 1.00) |
| Thailand | 2007 to 2011 | 0.94 (0.87, 1.02) |
| Thailand | 2012 to 2016 | 0.98 (0.9, 1.07) |
| Thailand | 2017 to 2021 | 1.15 (1.06, 1.26) |
| Timor-Leste | 1992 to 1996 | 1.03 (0.15, 6.99) |
| Timor-Leste | 1997 to 2001 | 1.01 (0.19, 5.34) |
| Timor-Leste | 2002 to 2006 | 1.00 (1.00, 1.00) |
| Timor-Leste | 2007 to 2011 | 1 (0.2, 5.08) |
| Timor-Leste | 2012 to 2016 | 1.72 (0.37, 7.86) |
| Timor-Leste | 2017 to 2021 | 1.62 (0.37, 7.17) |
| Togo | 1992 to 1996 | 1.41 (0.21, 9.62) |
| Togo | 1997 to 2001 | 1.12 (0.2, 6.24) |
| Togo | 2002 to 2006 | 1.00 (1.00, 1.00) |
| Togo | 2007 to 2011 | 0.71 (0.13, 3.9) |
| Togo | 2012 to 2016 | 0.99 (0.19, 5.05) |
| Togo | 2017 to 2021 | 1.12 (0.24, 5.18) |
| Trinidad and Tobago | 1992 to 1996 | 0.17 (0.01, 4.66) |
| Trinidad and Tobago | 1997 to 2001 | 0.29 (0.01, 6.09) |
| Trinidad and Tobago | 2002 to 2006 | 1.00 (1.00, 1.00) |
| Trinidad and Tobago | 2007 to 2011 | 1.02 (0.19, 5.61) |
| Trinidad and Tobago | 2012 to 2016 | 0.75 (0.12, 4.81) |
| Trinidad and Tobago | 2017 to 2021 | 0.73 (0.1, 5.53) |
| Tunisia | 1992 to 1996 | 0.84 (0.65, 1.07) |
| Tunisia | 1997 to 2001 | 0.91 (0.74, 1.13) |
| Tunisia | 2002 to 2006 | 1.00 (1.00, 1.00) |
| Tunisia | 2007 to 2011 | 1.04 (0.85, 1.26) |
| Tunisia | 2012 to 2016 | 1.13 (0.93, 1.37) |
| Tunisia | 2017 to 2021 | 1.23 (1.02, 1.49) |
| Turkey | 1992 to 1996 | 0.98 (0.82, 1.16) |
| Turkey | 1997 to 2001 | 1.01 (0.86, 1.18) |
| Turkey | 2002 to 2006 | 1.00 (1.00, 1.00) |
| Turkey | 2007 to 2011 | 1.14 (0.98, 1.32) |
| Turkey | 2012 to 2016 | 1.13 (0.97, 1.31) |
| Turkey | 2017 to 2021 | 1.13 (0.97, 1.3) |
| Turkmenistan | 1992 to 1996 | 1.15 (0.45, 2.94) |
| Turkmenistan | 1997 to 2001 | 1.07 (0.43, 2.62) |
| Turkmenistan | 2002 to 2006 | 1.00 (1.00, 1.00) |
| Turkmenistan | 2007 to 2011 | 1.23 (0.52, 2.87) |
| Turkmenistan | 2012 to 2016 | 1.57 (0.7, 3.54) |
| Turkmenistan | 2017 to 2021 | 1.63 (0.71, 3.71) |
| Uganda | 1992 to 1996 | 0.89 (0.73, 1.1) |
| Uganda | 1997 to 2001 | 1.05 (0.87, 1.25) |
| Uganda | 2002 to 2006 | 1.00 (1.00, 1.00) |
| Uganda | 2007 to 2011 | 0.87 (0.73, 1.03) |
| Uganda | 2012 to 2016 | 0.86 (0.73, 1.02) |
| Uganda | 2017 to 2021 | 0.93 (0.79, 1.1) |
| Ukraine | 1992 to 1996 | 0.96 (0.68, 1.36) |
| Ukraine | 1997 to 2001 | 0.91 (0.66, 1.26) |
| Ukraine | 2002 to 2006 | 1.00 (1.00, 1.00) |
| Ukraine | 2007 to 2011 | 1.12 (0.83, 1.51) |
| Ukraine | 2012 to 2016 | 1.18 (0.87, 1.6) |
| Ukraine | 2017 to 2021 | 1.28 (0.95, 1.73) |
| United Arab Emirates | 1992 to 1996 | 0.95 (0.44, 2.04) |
| United Arab Emirates | 1997 to 2001 | 0.95 (0.5, 1.84) |
| United Arab Emirates | 2002 to 2006 | 1.00 (1.00, 1.00) |
| United Arab Emirates | 2007 to 2011 | 1.03 (0.63, 1.7) |
| United Arab Emirates | 2012 to 2016 | 1.1 (0.66, 1.81) |
| United Arab Emirates | 2017 to 2021 | 1.02 (0.6, 1.74) |
| United Kingdom | 1992 to 1996 | 0.97 (0.84, 1.13) |
| United Kingdom | 1997 to 2001 | 1.01 (0.88, 1.15) |
| United Kingdom | 2002 to 2006 | 1.00 (1.00, 1.00) |
| United Kingdom | 2007 to 2011 | 1.08 (0.95, 1.22) |
| United Kingdom | 2012 to 2016 | 1.09 (0.96, 1.23) |
| United Kingdom | 2017 to 2021 | 1.07 (0.95, 1.22) |
| United Republic of Tanzania | 1992 to 1996 | 1.03 (0.8, 1.32) |
| United Republic of Tanzania | 1997 to 2001 | 1.02 (0.81, 1.27) |
| United Republic of Tanzania | 2002 to 2006 | 1.00 (1.00, 1.00) |
| United Republic of Tanzania | 2007 to 2011 | 1.01 (0.82, 1.24) |
| United Republic of Tanzania | 2012 to 2016 | 0.98 (0.8, 1.21) |
| United Republic of Tanzania | 2017 to 2021 | 1 (0.82, 1.22) |
| United States of America | 1992 to 1996 | 1.05 (0.97, 1.14) |
| United States of America | 1997 to 2001 | 1.04 (0.97, 1.12) |
| United States of America | 2002 to 2006 | 1.00 (1.00, 1.00) |
| United States of America | 2007 to 2011 | 0.97 (0.91, 1.04) |
| United States of America | 2012 to 2016 | 0.94 (0.88, 1.01) |
| United States of America | 2017 to 2021 | 0.96 (0.9, 1.03) |
| Uruguay | 1992 to 1996 | 0.97 (0.29, 3.25) |
| Uruguay | 1997 to 2001 | 0.96 (0.32, 2.88) |
| Uruguay | 2002 to 2006 | 1.00 (1.00, 1.00) |
| Uruguay | 2007 to 2011 | 1.55 (0.58, 4.15) |
| Uruguay | 2012 to 2016 | 1.33 (0.47, 3.77) |
| Uruguay | 2017 to 2021 | 1.36 (0.49, 3.79) |
| Uzbekistan | 1992 to 1996 | 1 (0.68, 1.48) |
| Uzbekistan | 1997 to 2001 | 1.02 (0.71, 1.45) |
| Uzbekistan | 2002 to 2006 | 1.00 (1.00, 1.00) |
| Uzbekistan | 2007 to 2011 | 1.01 (0.72, 1.42) |
| Uzbekistan | 2012 to 2016 | 1.13 (0.81, 1.57) |
| Uzbekistan | 2017 to 2021 | 1.34 (0.97, 1.85) |
| Venezuela (Bolivarian Republic of) | 1992 to 1996 | 0.61 (0.34, 1.07) |
| Venezuela (Bolivarian Republic of) | 1997 to 2001 | 0.86 (0.53, 1.38) |
| Venezuela (Bolivarian Republic of) | 2002 to 2006 | 1.00 (1.00, 1.00) |
| Venezuela (Bolivarian Republic of) | 2007 to 2011 | 1.27 (0.84, 1.91) |
| Venezuela (Bolivarian Republic of) | 2012 to 2016 | 1.3 (0.87, 1.96) |
| Venezuela (Bolivarian Republic of) | 2017 to 2021 | 1.3 (0.85, 1.98) |
| Viet Nam | 1992 to 1996 | 0.9 (0.82, 0.98) |
| Viet Nam | 1997 to 2001 | 0.96 (0.89, 1.04) |
| Viet Nam | 2002 to 2006 | 1.00 (1.00, 1.00) |
| Viet Nam | 2007 to 2011 | 1.08 (1.01, 1.16) |
| Viet Nam | 2012 to 2016 | 1.33 (1.24, 1.43) |
| Viet Nam | 2017 to 2021 | 1.58 (1.48, 1.68) |
| Yemen | 1992 to 1996 | 0.85 (0.38, 1.9) |
| Yemen | 1997 to 2001 | 0.88 (0.45, 1.72) |
| Yemen | 2002 to 2006 | 1.00 (1.00, 1.00) |
| Yemen | 2007 to 2011 | 0.87 (0.49, 1.57) |
| Yemen | 2012 to 2016 | 0.85 (0.48, 1.51) |
| Yemen | 2017 to 2021 | 0.76 (0.43, 1.33) |
| Zambia | 1992 to 1996 | 1.12 (0.73, 1.74) |
| Zambia | 1997 to 2001 | 1.05 (0.7, 1.58) |
| Zambia | 2002 to 2006 | 1.00 (1.00, 1.00) |
| Zambia | 2007 to 2011 | 1.17 (0.81, 1.67) |
| Zambia | 2012 to 2016 | 1.52 (1.09, 2.13) |
| Zambia | 2017 to 2021 | 1.72 (1.24, 2.38) |
| Zimbabwe | 1992 to 1996 | 0.43 (0.21, 0.86) |
| Zimbabwe | 1997 to 2001 | 0.67 (0.39, 1.15) |
| Zimbabwe | 2002 to 2006 | 1.00 (1.00, 1.00) |
| Zimbabwe | 2007 to 2011 | 0.9 (0.57, 1.45) |
| Zimbabwe | 2012 to 2016 | 0.94 (0.59, 1.5) |
| Zimbabwe | 2017 to 2021 | 0.97 (0.61, 1.53) |
