# Supplementary material for: Global, regional, and national temporal trends in prevalence for nasopharynx cancer across adolescents and young adults, 1990–2021: an age-period-cohort analysis based on the global burden of disease study 2021
Source: BMC Oral Health. 2025 Sep 26;25:1435. doi: 10.1186/s12903-025-06750-4 (PMC12465747; doi:10.1186/s12903-025-06750-4)
Supplement: Supplementary file 9 — Supplementary Material 9. Cohort effects on nasopharynx cancer prevalence in adolescents and young adults across countries. [file 12903_2025_6750_MOESM9_ESM.docx]

**Supplementary Table 9** Cohort effects on nasopharynx cancer prevalence in adolescents and young adults across countries

| **Location** | **Birth cohort** | **Prevalence rate ratio** |
| --- | --- | --- |
| Afghanistan | 1952 to 1961 | 1.53 (0.55, 4.31) |
| Afghanistan | 1957 to 1966 | 1.26 (0.56, 2.84) |
| Afghanistan | 1962 to 1971 | 1.22 (0.68, 2.19) |
| Afghanistan | 1967 to 1976 | 1.18 (0.7, 1.97) |
| Afghanistan | 1972 to 1981 | 1.11 (0.67, 1.83) |
| Afghanistan | 1977 to 1986 | 1.03 (0.62, 1.72) |
| Afghanistan | 1982 to 1991 | 1.00 (1.00, 1.00) |
| Afghanistan | 1987 to 1996 | 1 (0.57, 1.75) |
| Afghanistan | 1992 to 2001 | 1 (0.52, 1.91) |
| Afghanistan | 1997 to 2006 | 0.94 (0.4, 2.22) |
| Albania | 1952 to 1961 | 0.48 (0.08, 2.73) |
| Albania | 1957 to 1966 | 0.51 (0.15, 1.75) |
| Albania | 1962 to 1971 | 0.54 (0.18, 1.65) |
| Albania | 1967 to 1976 | 0.66 (0.24, 1.79) |
| Albania | 1972 to 1981 | 0.86 (0.34, 2.16) |
| Albania | 1977 to 1986 | 0.89 (0.36, 2.19) |
| Albania | 1982 to 1991 | 1.00 (1.00, 1.00) |
| Albania | 1987 to 1996 | 1.12 (0.38, 3.28) |
| Albania | 1992 to 2001 | 1.47 (0.41, 5.3) |
| Albania | 1997 to 2006 | 1.04 (0.11, 9.77) |
| Algeria | 1952 to 1961 | 0.82 (0.66, 1.03) |
| Algeria | 1957 to 1966 | 0.85 (0.72, 1.01) |
| Algeria | 1962 to 1971 | 0.89 (0.77, 1.02) |
| Algeria | 1967 to 1976 | 0.92 (0.8, 1.05) |
| Algeria | 1972 to 1981 | 0.94 (0.83, 1.06) |
| Algeria | 1977 to 1986 | 0.95 (0.84, 1.06) |
| Algeria | 1982 to 1991 | 1.00 (1.00, 1.00) |
| Algeria | 1987 to 1996 | 1.03 (0.88, 1.2) |
| Algeria | 1992 to 2001 | 1.11 (0.91, 1.34) |
| Algeria | 1997 to 2006 | 1.12 (0.86, 1.45) |
| Angola | 1952 to 1961 | 0.96 (0.27, 3.35) |
| Angola | 1957 to 1966 | 0.84 (0.3, 2.39) |
| Angola | 1962 to 1971 | 1.05 (0.44, 2.52) |
| Angola | 1967 to 1976 | 1.06 (0.47, 2.35) |
| Angola | 1972 to 1981 | 1.09 (0.53, 2.24) |
| Angola | 1977 to 1986 | 1.02 (0.51, 2.04) |
| Angola | 1982 to 1991 | 1.00 (1.00, 1.00) |
| Angola | 1987 to 1996 | 1.09 (0.49, 2.42) |
| Angola | 1992 to 2001 | 1.04 (0.4, 2.71) |
| Angola | 1997 to 2006 | 0.96 (0.26, 3.56) |
| Argentina | 1952 to 1961 | 1.17 (0.6, 2.27) |
| Argentina | 1957 to 1966 | 1.08 (0.64, 1.82) |
| Argentina | 1962 to 1971 | 0.99 (0.61, 1.59) |
| Argentina | 1967 to 1976 | 0.97 (0.63, 1.49) |
| Argentina | 1972 to 1981 | 0.97 (0.65, 1.43) |
| Argentina | 1977 to 1986 | 1 (0.68, 1.46) |
| Argentina | 1982 to 1991 | 1.00 (1.00, 1.00) |
| Argentina | 1987 to 1996 | 1.03 (0.66, 1.62) |
| Argentina | 1992 to 2001 | 1.13 (0.67, 1.9) |
| Argentina | 1997 to 2006 | 1.07 (0.51, 2.25) |
| Armenia | 1952 to 1961 | 0.88 (0.07, 11.51) |
| Armenia | 1957 to 1966 | 0.87 (0.13, 5.84) |
| Armenia | 1962 to 1971 | 1.12 (0.23, 5.52) |
| Armenia | 1967 to 1976 | 1.24 (0.29, 5.21) |
| Armenia | 1972 to 1981 | 1.17 (0.31, 4.45) |
| Armenia | 1977 to 1986 | 1.04 (0.27, 3.98) |
| Armenia | 1982 to 1991 | 1.00 (1.00, 1.00) |
| Armenia | 1987 to 1996 | 1.16 (0.25, 5.43) |
| Armenia | 1992 to 2001 | 1.67 (0.27, 10.27) |
| Armenia | 1997 to 2006 | 2.01 (0.17, 24.15) |
| Australia | 1952 to 1961 | 1.68 (1.32, 2.14) |
| Australia | 1957 to 1966 | 1.51 (1.25, 1.82) |
| Australia | 1962 to 1971 | 1.41 (1.18, 1.68) |
| Australia | 1967 to 1976 | 1.37 (1.15, 1.64) |
| Australia | 1972 to 1981 | 1.27 (1.06, 1.51) |
| Australia | 1977 to 1986 | 1.12 (0.95, 1.33) |
| Australia | 1982 to 1991 | 1.00 (1.00, 1.00) |
| Australia | 1987 to 1996 | 0.86 (0.68, 1.1) |
| Australia | 1992 to 2001 | 0.64 (0.45, 0.93) |
| Australia | 1997 to 2006 | 0.49 (0.27, 0.86) |
| Austria | 1952 to 1961 | 1.16 (0.55, 2.45) |
| Austria | 1957 to 1966 | 1.06 (0.59, 1.9) |
| Austria | 1962 to 1971 | 1.01 (0.6, 1.72) |
| Austria | 1967 to 1976 | 0.98 (0.59, 1.64) |
| Austria | 1972 to 1981 | 0.93 (0.56, 1.56) |
| Austria | 1977 to 1986 | 0.98 (0.6, 1.61) |
| Austria | 1982 to 1991 | 1.00 (1.00, 1.00) |
| Austria | 1987 to 1996 | 0.95 (0.51, 1.76) |
| Austria | 1992 to 2001 | 0.79 (0.34, 1.81) |
| Austria | 1997 to 2006 | 0.69 (0.19, 2.51) |
| Azerbaijan | 1952 to 1961 | 0.93 (0.16, 5.45) |
| Azerbaijan | 1957 to 1966 | 0.86 (0.24, 3.1) |
| Azerbaijan | 1962 to 1971 | 0.94 (0.32, 2.72) |
| Azerbaijan | 1967 to 1976 | 1.11 (0.43, 2.91) |
| Azerbaijan | 1972 to 1981 | 0.98 (0.39, 2.46) |
| Azerbaijan | 1977 to 1986 | 0.91 (0.36, 2.27) |
| Azerbaijan | 1982 to 1991 | 1.00 (1.00, 1.00) |
| Azerbaijan | 1987 to 1996 | 0.94 (0.34, 2.55) |
| Azerbaijan | 1992 to 2001 | 1.04 (0.3, 3.65) |
| Azerbaijan | 1997 to 2006 | 1.02 (0.18, 5.77) |
| Bahrain | 1952 to 1961 | 1.04 (0.09, 11.72) |
| Bahrain | 1957 to 1966 | 0.77 (0.13, 4.43) |
| Bahrain | 1962 to 1971 | 0.59 (0.08, 4.52) |
| Bahrain | 1967 to 1976 | 0.88 (0.16, 4.76) |
| Bahrain | 1972 to 1981 | 0.86 (0.19, 3.81) |
| Bahrain | 1977 to 1986 | 0.9 (0.27, 3.03) |
| Bahrain | 1982 to 1991 | 1.00 (1.00, 1.00) |
| Bahrain | 1987 to 1996 | 0.82 (0.14, 4.84) |
| Bahrain | 1992 to 2001 | 1.37 (0.15, 12.31) |
| Bahrain | 1997 to 2006 | 1.96 (0.07, 51.92) |
| Bangladesh | 1952 to 1961 | 1.06 (0.88, 1.28) |
| Bangladesh | 1957 to 1966 | 1.02 (0.87, 1.18) |
| Bangladesh | 1962 to 1971 | 0.99 (0.87, 1.13) |
| Bangladesh | 1967 to 1976 | 0.93 (0.81, 1.05) |
| Bangladesh | 1972 to 1981 | 0.91 (0.81, 1.03) |
| Bangladesh | 1977 to 1986 | 0.95 (0.85, 1.07) |
| Bangladesh | 1982 to 1991 | 1.00 (1.00, 1.00) |
| Bangladesh | 1987 to 1996 | 1.08 (0.94, 1.24) |
| Bangladesh | 1992 to 2001 | 1.1 (0.93, 1.31) |
| Bangladesh | 1997 to 2006 | 1.11 (0.89, 1.38) |
| Belarus | 1952 to 1961 | 1.67 (0.6, 4.6) |
| Belarus | 1957 to 1966 | 1.25 (0.52, 2.99) |
| Belarus | 1962 to 1971 | 1.12 (0.48, 2.6) |
| Belarus | 1967 to 1976 | 1.07 (0.47, 2.44) |
| Belarus | 1972 to 1981 | 1.07 (0.48, 2.36) |
| Belarus | 1977 to 1986 | 1.09 (0.51, 2.34) |
| Belarus | 1982 to 1991 | 1.00 (1.00, 1.00) |
| Belarus | 1987 to 1996 | 1.14 (0.41, 3.18) |
| Belarus | 1992 to 2001 | 0.92 (0.19, 4.42) |
| Belarus | 1997 to 2006 | 1.13 (0.13, 10.2) |
| Belgium | 1952 to 1961 | 1.09 (0.61, 1.98) |
| Belgium | 1957 to 1966 | 1.04 (0.65, 1.68) |
| Belgium | 1962 to 1971 | 1.03 (0.65, 1.61) |
| Belgium | 1967 to 1976 | 1.08 (0.7, 1.67) |
| Belgium | 1972 to 1981 | 1.05 (0.69, 1.61) |
| Belgium | 1977 to 1986 | 1.03 (0.68, 1.57) |
| Belgium | 1982 to 1991 | 1.00 (1.00, 1.00) |
| Belgium | 1987 to 1996 | 0.9 (0.53, 1.55) |
| Belgium | 1992 to 2001 | 0.85 (0.43, 1.69) |
| Belgium | 1997 to 2006 | 0.85 (0.3, 2.36) |
| Benin | 1952 to 1961 | 1.01 (0.08, 12.7) |
| Benin | 1957 to 1966 | 0.98 (0.15, 6.22) |
| Benin | 1962 to 1971 | 0.91 (0.13, 6.4) |
| Benin | 1967 to 1976 | 0.96 (0.2, 4.75) |
| Benin | 1972 to 1981 | 0.96 (0.2, 4.62) |
| Benin | 1977 to 1986 | 0.92 (0.22, 3.92) |
| Benin | 1982 to 1991 | 1.00 (1.00, 1.00) |
| Benin | 1987 to 1996 | 0.75 (0.12, 4.46) |
| Benin | 1992 to 2001 | 0.86 (0.12, 6.09) |
| Benin | 1997 to 2006 | 0.44 (0, 442.45) |
| Bhutan | 1952 to 1961 | 1.33 (0.08, 21.44) |
| Bhutan | 1957 to 1966 | 1.53 (0.2, 11.9) |
| Bhutan | 1962 to 1971 | 1.53 (0.25, 9.47) |
| Bhutan | 1967 to 1976 | 1.04 (0.11, 10.14) |
| Bhutan | 1972 to 1981 | 1.09 (0.16, 7.38) |
| Bhutan | 1977 to 1986 | 1.35 (0.23, 7.78) |
| Bhutan | 1982 to 1991 | 1.00 (1.00, 1.00) |
| Bhutan | 1987 to 1996 | 1.92 (0.22, 16.55) |
| Bhutan | 1992 to 2001 | 0.63 (0, 87.86) |
| Bhutan | 1997 to 2006 | 1.34 (0, 1520.92) |
| Bolivia (Plurinational State of) | 1952 to 1961 | 1.45 (0.21, 10.07) |
| Bolivia (Plurinational State of) | 1957 to 1966 | 1.28 (0.26, 6.38) |
| Bolivia (Plurinational State of) | 1962 to 1971 | 1.26 (0.31, 5.22) |
| Bolivia (Plurinational State of) | 1967 to 1976 | 1.17 (0.31, 4.39) |
| Bolivia (Plurinational State of) | 1972 to 1981 | 0.99 (0.28, 3.48) |
| Bolivia (Plurinational State of) | 1977 to 1986 | 1.14 (0.35, 3.65) |
| Bolivia (Plurinational State of) | 1982 to 1991 | 1.00 (1.00, 1.00) |
| Bolivia (Plurinational State of) | 1987 to 1996 | 0.79 (0.18, 3.54) |
| Bolivia (Plurinational State of) | 1992 to 2001 | 0.74 (0.12, 4.34) |
| Bolivia (Plurinational State of) | 1997 to 2006 | 0.73 (0.07, 8.02) |
| Bosnia and Herzegovina | 1952 to 1961 | 0.45 (0.03, 6.58) |
| Bosnia and Herzegovina | 1957 to 1966 | 0.47 (0.06, 3.41) |
| Bosnia and Herzegovina | 1962 to 1971 | 0.35 (0.04, 3.38) |
| Bosnia and Herzegovina | 1967 to 1976 | 0.32 (0.03, 2.92) |
| Bosnia and Herzegovina | 1972 to 1981 | 0.6 (0.1, 3.51) |
| Bosnia and Herzegovina | 1977 to 1986 | 0.69 (0.14, 3.24) |
| Bosnia and Herzegovina | 1982 to 1991 | 1.00 (1.00, 1.00) |
| Bosnia and Herzegovina | 1987 to 1996 | 2.14 (0.36, 12.86) |
| Bosnia and Herzegovina | 1992 to 2001 | 0.34 (0, 40.5) |
| Bosnia and Herzegovina | 1997 to 2006 | 0.81 (0, 734.04) |
| Botswana | 1952 to 1961 | 1.33 (0.1, 17.49) |
| Botswana | 1957 to 1966 | 1.37 (0.2, 9.47) |
| Botswana | 1962 to 1971 | 1.05 (0.15, 7.48) |
| Botswana | 1967 to 1976 | 0.79 (0.12, 5.26) |
| Botswana | 1972 to 1981 | 1.16 (0.28, 4.82) |
| Botswana | 1977 to 1986 | 1 (0.23, 4.25) |
| Botswana | 1982 to 1991 | 1.00 (1.00, 1.00) |
| Botswana | 1987 to 1996 | 1.14 (0.24, 5.49) |
| Botswana | 1992 to 2001 | 1.2 (0.18, 7.9) |
| Botswana | 1997 to 2006 | 1.09 (0.09, 13.39) |
| Brazil | 1952 to 1961 | 0.8 (0.6, 1.09) |
| Brazil | 1957 to 1966 | 0.76 (0.61, 0.96) |
| Brazil | 1962 to 1971 | 0.78 (0.64, 0.96) |
| Brazil | 1967 to 1976 | 0.8 (0.67, 0.95) |
| Brazil | 1972 to 1981 | 0.82 (0.7, 0.97) |
| Brazil | 1977 to 1986 | 0.9 (0.77, 1.06) |
| Brazil | 1982 to 1991 | 1.00 (1.00, 1.00) |
| Brazil | 1987 to 1996 | 1.21 (1.01, 1.46) |
| Brazil | 1992 to 2001 | 1.5 (1.22, 1.86) |
| Brazil | 1997 to 2006 | 1.66 (1.24, 2.23) |
| Brunei Darussalam | 1952 to 1961 | 0.96 (0.14, 6.72) |
| Brunei Darussalam | 1957 to 1966 | 0.9 (0.18, 4.61) |
| Brunei Darussalam | 1962 to 1971 | 1.03 (0.23, 4.66) |
| Brunei Darussalam | 1967 to 1976 | 1.14 (0.29, 4.48) |
| Brunei Darussalam | 1972 to 1981 | 1.26 (0.34, 4.76) |
| Brunei Darussalam | 1977 to 1986 | 1.22 (0.35, 4.25) |
| Brunei Darussalam | 1982 to 1991 | 1.00 (1.00, 1.00) |
| Brunei Darussalam | 1987 to 1996 | 0.89 (0.16, 5.06) |
| Brunei Darussalam | 1992 to 2001 | 0.82 (0.08, 8.09) |
| Brunei Darussalam | 1997 to 2006 | 0.81 (0, 802.43) |
| Bulgaria | 1952 to 1961 | 0.63 (0.26, 1.52) |
| Bulgaria | 1957 to 1966 | 0.68 (0.33, 1.43) |
| Bulgaria | 1962 to 1971 | 0.74 (0.37, 1.46) |
| Bulgaria | 1967 to 1976 | 0.77 (0.4, 1.48) |
| Bulgaria | 1972 to 1981 | 0.87 (0.46, 1.62) |
| Bulgaria | 1977 to 1986 | 0.99 (0.54, 1.79) |
| Bulgaria | 1982 to 1991 | 1.00 (1.00, 1.00) |
| Bulgaria | 1987 to 1996 | 1.19 (0.53, 2.66) |
| Bulgaria | 1992 to 2001 | 1.36 (0.47, 3.88) |
| Bulgaria | 1997 to 2006 | 1.67 (0.43, 6.46) |
| Burkina Faso | 1952 to 1961 | 0.43 (0.04, 4.12) |
| Burkina Faso | 1957 to 1966 | 0.72 (0.16, 3.19) |
| Burkina Faso | 1962 to 1971 | 0.79 (0.21, 3.01) |
| Burkina Faso | 1967 to 1976 | 0.96 (0.31, 2.99) |
| Burkina Faso | 1972 to 1981 | 0.87 (0.28, 2.74) |
| Burkina Faso | 1977 to 1986 | 0.9 (0.33, 2.4) |
| Burkina Faso | 1982 to 1991 | 1.00 (1.00, 1.00) |
| Burkina Faso | 1987 to 1996 | 0.81 (0.22, 2.97) |
| Burkina Faso | 1992 to 2001 | 0.99 (0.25, 3.94) |
| Burkina Faso | 1997 to 2006 | 0.6 (0.05, 6.66) |
| Burundi | 1952 to 1961 | 1.39 (0.65, 2.95) |
| Burundi | 1957 to 1966 | 1.21 (0.65, 2.26) |
| Burundi | 1962 to 1971 | 1.13 (0.64, 2) |
| Burundi | 1967 to 1976 | 1.1 (0.65, 1.88) |
| Burundi | 1972 to 1981 | 1.07 (0.66, 1.73) |
| Burundi | 1977 to 1986 | 1.02 (0.65, 1.6) |
| Burundi | 1982 to 1991 | 1.00 (1.00, 1.00) |
| Burundi | 1987 to 1996 | 0.92 (0.54, 1.57) |
| Burundi | 1992 to 2001 | 0.9 (0.47, 1.7) |
| Burundi | 1997 to 2006 | 0.8 (0.35, 1.82) |
| Cambodia | 1952 to 1961 | 0.8 (0.5, 1.28) |
| Cambodia | 1957 to 1966 | 0.82 (0.57, 1.17) |
| Cambodia | 1962 to 1971 | 0.86 (0.61, 1.2) |
| Cambodia | 1967 to 1976 | 0.91 (0.66, 1.26) |
| Cambodia | 1972 to 1981 | 0.95 (0.7, 1.28) |
| Cambodia | 1977 to 1986 | 0.97 (0.74, 1.27) |
| Cambodia | 1982 to 1991 | 1.00 (1.00, 1.00) |
| Cambodia | 1987 to 1996 | 1.03 (0.73, 1.46) |
| Cambodia | 1992 to 2001 | 1.05 (0.66, 1.66) |
| Cambodia | 1997 to 2006 | 1.15 (0.6, 2.18) |
| Cameroon | 1952 to 1961 | 0.6 (0.12, 3.08) |
| Cameroon | 1957 to 1966 | 0.81 (0.27, 2.44) |
| Cameroon | 1962 to 1971 | 0.79 (0.27, 2.27) |
| Cameroon | 1967 to 1976 | 0.91 (0.38, 2.15) |
| Cameroon | 1972 to 1981 | 0.87 (0.38, 1.97) |
| Cameroon | 1977 to 1986 | 0.97 (0.47, 2) |
| Cameroon | 1982 to 1991 | 1.00 (1.00, 1.00) |
| Cameroon | 1987 to 1996 | 0.89 (0.34, 2.33) |
| Cameroon | 1992 to 2001 | 0.93 (0.33, 2.61) |
| Cameroon | 1997 to 2006 | 0.52 (0.06, 4.89) |
| Canada | 1952 to 1961 | 1.1 (0.83, 1.46) |
| Canada | 1957 to 1966 | 1.05 (0.84, 1.31) |
| Canada | 1962 to 1971 | 1.01 (0.82, 1.25) |
| Canada | 1967 to 1976 | 0.97 (0.79, 1.2) |
| Canada | 1972 to 1981 | 0.98 (0.8, 1.19) |
| Canada | 1977 to 1986 | 0.98 (0.81, 1.18) |
| Canada | 1982 to 1991 | 1.00 (1.00, 1.00) |
| Canada | 1987 to 1996 | 0.96 (0.76, 1.23) |
| Canada | 1992 to 2001 | 0.82 (0.6, 1.13) |
| Canada | 1997 to 2006 | 0.63 (0.38, 1.05) |
| Central African Republic | 1952 to 1961 | 1.11 (0.09, 13.22) |
| Central African Republic | 1957 to 1966 | 1.21 (0.18, 7.94) |
| Central African Republic | 1962 to 1971 | 1.21 (0.24, 6.13) |
| Central African Republic | 1967 to 1976 | 1.31 (0.26, 6.65) |
| Central African Republic | 1972 to 1981 | 1.2 (0.28, 5.07) |
| Central African Republic | 1977 to 1986 | 1.14 (0.3, 4.28) |
| Central African Republic | 1982 to 1991 | 1.00 (1.00, 1.00) |
| Central African Republic | 1987 to 1996 | 0.92 (0.2, 4.38) |
| Central African Republic | 1992 to 2001 | 0.86 (0.13, 5.81) |
| Central African Republic | 1997 to 2006 | 0.77 (0.06, 10.84) |
| Chad | 1952 to 1961 | 0.83 (0.07, 9.87) |
| Chad | 1957 to 1966 | 0.92 (0.15, 5.59) |
| Chad | 1962 to 1971 | 0.92 (0.13, 6.28) |
| Chad | 1967 to 1976 | 1.12 (0.24, 5.24) |
| Chad | 1972 to 1981 | 0.87 (0.19, 4) |
| Chad | 1977 to 1986 | 1.24 (0.34, 4.51) |
| Chad | 1982 to 1991 | 1.00 (1.00, 1.00) |
| Chad | 1987 to 1996 | 1.09 (0.21, 5.61) |
| Chad | 1992 to 2001 | 0.97 (0.14, 6.6) |
| Chad | 1997 to 2006 | 5.38 (0.15, 197.19) |
| Chile | 1952 to 1961 | 0.75 (0.2, 2.77) |
| Chile | 1957 to 1966 | 0.7 (0.25, 1.92) |
| Chile | 1962 to 1971 | 0.73 (0.29, 1.83) |
| Chile | 1967 to 1976 | 0.76 (0.33, 1.75) |
| Chile | 1972 to 1981 | 0.89 (0.41, 1.95) |
| Chile | 1977 to 1986 | 0.94 (0.44, 1.99) |
| Chile | 1982 to 1991 | 1.00 (1.00, 1.00) |
| Chile | 1987 to 1996 | 1.12 (0.47, 2.65) |
| Chile | 1992 to 2001 | 1 (0.33, 3.07) |
| Chile | 1997 to 2006 | 0.93 (0.18, 4.87) |
| China | 1952 to 1961 | 0.61 (0.53, 0.7) |
| China | 1957 to 1966 | 0.59 (0.52, 0.66) |
| China | 1962 to 1971 | 0.63 (0.57, 0.7) |
| China | 1967 to 1976 | 0.71 (0.63, 0.79) |
| China | 1972 to 1981 | 0.81 (0.73, 0.9) |
| China | 1977 to 1986 | 0.92 (0.84, 1.01) |
| China | 1982 to 1991 | 1.00 (1.00, 1.00) |
| China | 1987 to 1996 | 1.09 (0.95, 1.24) |
| China | 1992 to 2001 | 1.05 (0.85, 1.29) |
| China | 1997 to 2006 | 0.87 (0.62, 1.23) |
| Colombia | 1952 to 1961 | 1.22 (0.67, 2.24) |
| Colombia | 1957 to 1966 | 1.1 (0.7, 1.73) |
| Colombia | 1962 to 1971 | 1.07 (0.72, 1.58) |
| Colombia | 1967 to 1976 | 1.03 (0.72, 1.49) |
| Colombia | 1972 to 1981 | 1.05 (0.74, 1.47) |
| Colombia | 1977 to 1986 | 1.03 (0.74, 1.44) |
| Colombia | 1982 to 1991 | 1.00 (1.00, 1.00) |
| Colombia | 1987 to 1996 | 0.98 (0.66, 1.46) |
| Colombia | 1992 to 2001 | 0.94 (0.58, 1.53) |
| Colombia | 1997 to 2006 | 0.93 (0.47, 1.86) |
| Comoros | 1952 to 1961 | 1.32 (0.07, 25.93) |
| Comoros | 1957 to 1966 | 1.13 (0.12, 10.39) |
| Comoros | 1962 to 1971 | 0.87 (0.08, 9.19) |
| Comoros | 1967 to 1976 | 0.76 (0.07, 8.77) |
| Comoros | 1972 to 1981 | 0.68 (0.07, 6.63) |
| Comoros | 1977 to 1986 | 0.65 (0.08, 5.15) |
| Comoros | 1982 to 1991 | 1.00 (1.00, 1.00) |
| Comoros | 1987 to 1996 | 1.44 (0.11, 18.42) |
| Comoros | 1992 to 2001 | 4.79 (0.16, 140.56) |
| Comoros | 1997 to 2006 | 11.72 (0.24, 576.3) |
| Congo | 1952 to 1961 | 0.93 (0.08, 10.62) |
| Congo | 1957 to 1966 | 1.04 (0.17, 6.17) |
| Congo | 1962 to 1971 | 1.27 (0.27, 6) |
| Congo | 1967 to 1976 | 0.97 (0.2, 4.79) |
| Congo | 1972 to 1981 | 0.93 (0.22, 3.87) |
| Congo | 1977 to 1986 | 0.93 (0.24, 3.54) |
| Congo | 1982 to 1991 | 1.00 (1.00, 1.00) |
| Congo | 1987 to 1996 | 0.98 (0.19, 5.15) |
| Congo | 1992 to 2001 | 1.01 (0.12, 8.31) |
| Congo | 1997 to 2006 | 0.95 (0.05, 19.91) |
| Costa Rica | 1952 to 1961 | 1.33 (0.35, 5.05) |
| Costa Rica | 1957 to 1966 | 1.18 (0.43, 3.27) |
| Costa Rica | 1962 to 1971 | 1.06 (0.43, 2.62) |
| Costa Rica | 1967 to 1976 | 1 (0.42, 2.38) |
| Costa Rica | 1972 to 1981 | 1.01 (0.45, 2.25) |
| Costa Rica | 1977 to 1986 | 1.01 (0.46, 2.19) |
| Costa Rica | 1982 to 1991 | 1.00 (1.00, 1.00) |
| Costa Rica | 1987 to 1996 | 1.15 (0.47, 2.85) |
| Costa Rica | 1992 to 2001 | 1.09 (0.35, 3.36) |
| Costa Rica | 1997 to 2006 | 0.93 (0.18, 4.86) |
| Coted'Ivoire | 1952 to 1961 | 0.79 (0.24, 2.6) |
| Coted'Ivoire | 1957 to 1966 | 0.78 (0.34, 1.81) |
| Coted'Ivoire | 1962 to 1971 | 0.79 (0.36, 1.77) |
| Coted'Ivoire | 1967 to 1976 | 0.87 (0.44, 1.71) |
| Coted'Ivoire | 1972 to 1981 | 0.87 (0.46, 1.62) |
| Coted'Ivoire | 1977 to 1986 | 0.95 (0.54, 1.69) |
| Coted'Ivoire | 1982 to 1991 | 1.00 (1.00, 1.00) |
| Coted'Ivoire | 1987 to 1996 | 1.12 (0.54, 2.29) |
| Coted'Ivoire | 1992 to 2001 | 1.12 (0.5, 2.52) |
| Coted'Ivoire | 1997 to 2006 | 1.03 (0.3, 3.52) |
| Croatia | 1952 to 1961 | 1.06 (0.25, 4.4) |
| Croatia | 1957 to 1966 | 0.8 (0.23, 2.83) |
| Croatia | 1962 to 1971 | 0.78 (0.24, 2.54) |
| Croatia | 1967 to 1976 | 1 (0.34, 2.96) |
| Croatia | 1972 to 1981 | 0.92 (0.33, 2.6) |
| Croatia | 1977 to 1986 | 0.99 (0.35, 2.77) |
| Croatia | 1982 to 1991 | 1.00 (1.00, 1.00) |
| Croatia | 1987 to 1996 | 0.97 (0.23, 4.05) |
| Croatia | 1992 to 2001 | 1.3 (0.23, 7.17) |
| Croatia | 1997 to 2006 | 1.7 (0.16, 17.94) |
| Cuba | 1952 to 1961 | 0.73 (0.28, 1.91) |
| Cuba | 1957 to 1966 | 0.88 (0.46, 1.7) |
| Cuba | 1962 to 1971 | 0.91 (0.52, 1.62) |
| Cuba | 1967 to 1976 | 0.99 (0.57, 1.71) |
| Cuba | 1972 to 1981 | 1 (0.57, 1.74) |
| Cuba | 1977 to 1986 | 1.03 (0.59, 1.81) |
| Cuba | 1982 to 1991 | 1.00 (1.00, 1.00) |
| Cuba | 1987 to 1996 | 1.03 (0.52, 2.04) |
| Cuba | 1992 to 2001 | 1.11 (0.47, 2.62) |
| Cuba | 1997 to 2006 | 1.17 (0.36, 3.83) |
| Cyprus | 1952 to 1961 | 1.21 (0.08, 18.62) |
| Cyprus | 1957 to 1966 | 0.35 (0.02, 7.95) |
| Cyprus | 1962 to 1971 | 0.5 (0.04, 6.04) |
| Cyprus | 1967 to 1976 | 0.49 (0.05, 4.98) |
| Cyprus | 1972 to 1981 | 0.82 (0.13, 5.18) |
| Cyprus | 1977 to 1986 | 0.81 (0.15, 4.22) |
| Cyprus | 1982 to 1991 | 1.00 (1.00, 1.00) |
| Cyprus | 1987 to 1996 | 1.41 (0.21, 9.59) |
| Cyprus | 1992 to 2001 | 2.18 (0.14, 33.36) |
| Cyprus | 1997 to 2006 | 1.85 (0, 1999.94) |
| Czechia | 1952 to 1961 | 0.72 (0.36, 1.41) |
| Czechia | 1957 to 1966 | 0.67 (0.38, 1.18) |
| Czechia | 1962 to 1971 | 0.68 (0.41, 1.14) |
| Czechia | 1967 to 1976 | 0.69 (0.43, 1.1) |
| Czechia | 1972 to 1981 | 0.79 (0.51, 1.21) |
| Czechia | 1977 to 1986 | 0.89 (0.58, 1.36) |
| Czechia | 1982 to 1991 | 1.00 (1.00, 1.00) |
| Czechia | 1987 to 1996 | 1.14 (0.66, 1.98) |
| Czechia | 1992 to 2001 | 1.33 (0.63, 2.79) |
| Czechia | 1997 to 2006 | 1.31 (0.43, 4.02) |
| Democratic People's Republic of Korea | 1952 to 1961 | 0.84 (0.65, 1.07) |
| Democratic People's Republic of Korea | 1957 to 1966 | 0.87 (0.72, 1.05) |
| Democratic People's Republic of Korea | 1962 to 1971 | 0.89 (0.75, 1.07) |
| Democratic People's Republic of Korea | 1967 to 1976 | 0.92 (0.77, 1.1) |
| Democratic People's Republic of Korea | 1972 to 1981 | 0.95 (0.79, 1.13) |
| Democratic People's Republic of Korea | 1977 to 1986 | 0.96 (0.81, 1.13) |
| Democratic People's Republic of Korea | 1982 to 1991 | 1.00 (1.00, 1.00) |
| Democratic People's Republic of Korea | 1987 to 1996 | 1.01 (0.81, 1.26) |
| Democratic People's Republic of Korea | 1992 to 2001 | 0.97 (0.71, 1.33) |
| Democratic People's Republic of Korea | 1997 to 2006 | 0.89 (0.55, 1.44) |
| Democratic Republic of the Congo | 1952 to 1961 | 0.87 (0.4, 1.87) |
| Democratic Republic of the Congo | 1957 to 1966 | 0.94 (0.51, 1.7) |
| Democratic Republic of the Congo | 1962 to 1971 | 0.99 (0.58, 1.68) |
| Democratic Republic of the Congo | 1967 to 1976 | 0.97 (0.59, 1.59) |
| Democratic Republic of the Congo | 1972 to 1981 | 0.94 (0.59, 1.5) |
| Democratic Republic of the Congo | 1977 to 1986 | 0.94 (0.61, 1.45) |
| Democratic Republic of the Congo | 1982 to 1991 | 1.00 (1.00, 1.00) |
| Democratic Republic of the Congo | 1987 to 1996 | 0.99 (0.6, 1.65) |
| Democratic Republic of the Congo | 1992 to 2001 | 0.96 (0.52, 1.76) |
| Democratic Republic of the Congo | 1997 to 2006 | 0.93 (0.41, 2.12) |
| Denmark | 1952 to 1961 | 1.04 (0.33, 3.31) |
| Denmark | 1957 to 1966 | 1.07 (0.42, 2.71) |
| Denmark | 1962 to 1971 | 1.01 (0.43, 2.39) |
| Denmark | 1967 to 1976 | 0.98 (0.42, 2.27) |
| Denmark | 1972 to 1981 | 0.9 (0.39, 2.11) |
| Denmark | 1977 to 1986 | 0.95 (0.41, 2.18) |
| Denmark | 1982 to 1991 | 1.00 (1.00, 1.00) |
| Denmark | 1987 to 1996 | 1.09 (0.4, 2.96) |
| Denmark | 1992 to 2001 | 0.81 (0.21, 3.16) |
| Denmark | 1997 to 2006 | 0.65 (0.07, 5.99) |
| Djibouti | 1952 to 1961 | 0.89 (0.07, 11.53) |
| Djibouti | 1957 to 1966 | 1 (0.16, 6.29) |
| Djibouti | 1962 to 1971 | 0.61 (0.08, 4.93) |
| Djibouti | 1967 to 1976 | 0.55 (0.07, 4.17) |
| Djibouti | 1972 to 1981 | 0.62 (0.12, 3.25) |
| Djibouti | 1977 to 1986 | 0.7 (0.17, 2.93) |
| Djibouti | 1982 to 1991 | 1.00 (1.00, 1.00) |
| Djibouti | 1987 to 1996 | 1.13 (0.23, 5.6) |
| Djibouti | 1992 to 2001 | 1.41 (0.18, 11.22) |
| Djibouti | 1997 to 2006 | 1.31 (0.07, 23.98) |
| Dominican Republic | 1952 to 1961 | 0.9 (0.26, 3.03) |
| Dominican Republic | 1957 to 1966 | 0.84 (0.35, 2.02) |
| Dominican Republic | 1962 to 1971 | 0.84 (0.4, 1.78) |
| Dominican Republic | 1967 to 1976 | 0.81 (0.4, 1.64) |
| Dominican Republic | 1972 to 1981 | 0.84 (0.44, 1.61) |
| Dominican Republic | 1977 to 1986 | 0.93 (0.5, 1.72) |
| Dominican Republic | 1982 to 1991 | 1.00 (1.00, 1.00) |
| Dominican Republic | 1987 to 1996 | 1.02 (0.49, 2.12) |
| Dominican Republic | 1992 to 2001 | 1.16 (0.47, 2.88) |
| Dominican Republic | 1997 to 2006 | 1.42 (0.4, 5.02) |
| Ecuador | 1952 to 1961 | 0.91 (0.17, 4.94) |
| Ecuador | 1957 to 1966 | 0.95 (0.29, 3.16) |
| Ecuador | 1962 to 1971 | 0.81 (0.27, 2.38) |
| Ecuador | 1967 to 1976 | 0.85 (0.33, 2.21) |
| Ecuador | 1972 to 1981 | 0.82 (0.34, 1.97) |
| Ecuador | 1977 to 1986 | 0.91 (0.4, 2.07) |
| Ecuador | 1982 to 1991 | 1.00 (1.00, 1.00) |
| Ecuador | 1987 to 1996 | 0.92 (0.34, 2.49) |
| Ecuador | 1992 to 2001 | 1.08 (0.34, 3.5) |
| Ecuador | 1997 to 2006 | 0.93 (0.17, 5.18) |
| Egypt | 1952 to 1961 | 1.36 (0.57, 3.27) |
| Egypt | 1957 to 1966 | 1.15 (0.58, 2.29) |
| Egypt | 1962 to 1971 | 1.05 (0.57, 1.95) |
| Egypt | 1967 to 1976 | 1.01 (0.56, 1.8) |
| Egypt | 1972 to 1981 | 0.98 (0.57, 1.67) |
| Egypt | 1977 to 1986 | 0.96 (0.58, 1.6) |
| Egypt | 1982 to 1991 | 1.00 (1.00, 1.00) |
| Egypt | 1987 to 1996 | 0.97 (0.52, 1.82) |
| Egypt | 1992 to 2001 | 0.93 (0.42, 2.04) |
| Egypt | 1997 to 2006 | 1.03 (0.37, 2.82) |
| El Salvador | 1952 to 1961 | 0.39 (0.04, 4.01) |
| El Salvador | 1957 to 1966 | 0.66 (0.14, 3.17) |
| El Salvador | 1962 to 1971 | 0.64 (0.16, 2.47) |
| El Salvador | 1967 to 1976 | 0.78 (0.24, 2.61) |
| El Salvador | 1972 to 1981 | 0.82 (0.27, 2.5) |
| El Salvador | 1977 to 1986 | 0.85 (0.28, 2.59) |
| El Salvador | 1982 to 1991 | 1.00 (1.00, 1.00) |
| El Salvador | 1987 to 1996 | 1.4 (0.43, 4.59) |
| El Salvador | 1992 to 2001 | 1.43 (0.35, 5.86) |
| El Salvador | 1997 to 2006 | 1.79 (0.26, 12.14) |
| Equatorial Guinea | 1952 to 1961 | 1.17 (0, 2499.59) |
| Equatorial Guinea | 1957 to 1966 | 1.8 (0.01, 615.88) |
| Equatorial Guinea | 1962 to 1971 | 1.86 (0.01, 269.33) |
| Equatorial Guinea | 1967 to 1976 | 1.76 (0.02, 160.14) |
| Equatorial Guinea | 1972 to 1981 | 1.51 (0.02, 102.66) |
| Equatorial Guinea | 1977 to 1986 | 2.3 (0.04, 138.62) |
| Equatorial Guinea | 1982 to 1991 | 1.00 (1.00, 1.00) |
| Equatorial Guinea | 1987 to 1996 | 0.79 (0.01, 103.22) |
| Equatorial Guinea | 1992 to 2001 | 0.57 (0, 178.65) |
| Equatorial Guinea | 1997 to 2006 | 0.3 (0, 719.49) |
| Eritrea | 1952 to 1961 | 0.9 (0.32, 2.55) |
| Eritrea | 1957 to 1966 | 0.87 (0.37, 2.05) |
| Eritrea | 1962 to 1971 | 0.89 (0.42, 1.89) |
| Eritrea | 1967 to 1976 | 0.94 (0.48, 1.86) |
| Eritrea | 1972 to 1981 | 0.94 (0.51, 1.75) |
| Eritrea | 1977 to 1986 | 0.93 (0.52, 1.68) |
| Eritrea | 1982 to 1991 | 1.00 (1.00, 1.00) |
| Eritrea | 1987 to 1996 | 1.01 (0.5, 2.05) |
| Eritrea | 1992 to 2001 | 0.98 (0.41, 2.33) |
| Eritrea | 1997 to 2006 | 0.98 (0.32, 3.03) |
| Estonia | 1952 to 1961 | 1.59 (0.15, 16.45) |
| Estonia | 1957 to 1966 | 1.07 (0.16, 7.21) |
| Estonia | 1962 to 1971 | 1.01 (0.18, 5.64) |
| Estonia | 1967 to 1976 | 0.85 (0.15, 4.68) |
| Estonia | 1972 to 1981 | 0.54 (0.08, 3.87) |
| Estonia | 1977 to 1986 | 0.79 (0.15, 4.14) |
| Estonia | 1982 to 1991 | 1.00 (1.00, 1.00) |
| Estonia | 1987 to 1996 | 0.21 (0, 9.94) |
| Estonia | 1992 to 2001 | 0.39 (0, 47.57) |
| Estonia | 1997 to 2006 | 0.92 (0, 1061.38) |
| Eswatini | 1952 to 1961 | 0.06 (0, 62.62) |
| Eswatini | 1957 to 1966 | 0.44 (0.01, 17.61) |
| Eswatini | 1962 to 1971 | 0.36 (0.01, 8.48) |
| Eswatini | 1967 to 1976 | 0.37 (0.01, 12.28) |
| Eswatini | 1972 to 1981 | 0.26 (0.01, 6.85) |
| Eswatini | 1977 to 1986 | 0.49 (0.05, 5.29) |
| Eswatini | 1982 to 1991 | 1.00 (1.00, 1.00) |
| Eswatini | 1987 to 1996 | 1.02 (0.04, 28.2) |
| Eswatini | 1992 to 2001 | 0.24 (0, 40.63) |
| Eswatini | 1997 to 2006 | 0.19 (0, 165.51) |
| Ethiopia | 1952 to 1961 | 1.31 (1.01, 1.7) |
| Ethiopia | 1957 to 1966 | 1.23 (1, 1.52) |
| Ethiopia | 1962 to 1971 | 1.17 (0.97, 1.41) |
| Ethiopia | 1967 to 1976 | 1.13 (0.95, 1.34) |
| Ethiopia | 1972 to 1981 | 1.08 (0.92, 1.27) |
| Ethiopia | 1977 to 1986 | 1.04 (0.89, 1.21) |
| Ethiopia | 1982 to 1991 | 1.00 (1.00, 1.00) |
| Ethiopia | 1987 to 1996 | 0.94 (0.78, 1.13) |
| Ethiopia | 1992 to 2001 | 0.89 (0.72, 1.1) |
| Ethiopia | 1997 to 2006 | 0.83 (0.63, 1.09) |
| Finland | 1952 to 1961 | 1.02 (0.26, 3.92) |
| Finland | 1957 to 1966 | 0.96 (0.32, 2.89) |
| Finland | 1962 to 1971 | 0.93 (0.33, 2.6) |
| Finland | 1967 to 1976 | 0.97 (0.36, 2.6) |
| Finland | 1972 to 1981 | 0.86 (0.33, 2.24) |
| Finland | 1977 to 1986 | 0.93 (0.37, 2.32) |
| Finland | 1982 to 1991 | 1.00 (1.00, 1.00) |
| Finland | 1987 to 1996 | 1.04 (0.33, 3.3) |
| Finland | 1992 to 2001 | 1.26 (0.3, 5.34) |
| Finland | 1997 to 2006 | 1.23 (0.12, 12.48) |
| France | 1952 to 1961 | 0.99 (0.83, 1.18) |
| France | 1957 to 1966 | 0.86 (0.74, 0.99) |
| France | 1962 to 1971 | 0.81 (0.7, 0.93) |
| France | 1967 to 1976 | 0.84 (0.74, 0.95) |
| France | 1972 to 1981 | 0.88 (0.77, 0.99) |
| France | 1977 to 1986 | 0.95 (0.84, 1.07) |
| France | 1982 to 1991 | 1.00 (1.00, 1.00) |
| France | 1987 to 1996 | 0.99 (0.84, 1.15) |
| France | 1992 to 2001 | 0.91 (0.74, 1.11) |
| France | 1997 to 2006 | 0.83 (0.61, 1.13) |
| Gabon | 1952 to 1961 | 0.05 (0, 61.33) |
| Gabon | 1957 to 1966 | 0.4 (0.01, 18.01) |
| Gabon | 1962 to 1971 | 0.46 (0.01, 17.5) |
| Gabon | 1967 to 1976 | 0.46 (0.01, 16.81) |
| Gabon | 1972 to 1981 | 0.44 (0.01, 14.67) |
| Gabon | 1977 to 1986 | 0.27 (0.01, 5.34) |
| Gabon | 1982 to 1991 | 1.00 (1.00, 1.00) |
| Gabon | 1987 to 1996 | 0.37 (0, 35.65) |
| Gabon | 1992 to 2001 | 0.32 (0, 72.31) |
| Gabon | 1997 to 2006 | 0.22 (0, 338.82) |
| Georgia | 1952 to 1961 | 0.67 (0.14, 3.3) |
| Georgia | 1957 to 1966 | 0.74 (0.22, 2.52) |
| Georgia | 1962 to 1971 | 0.93 (0.32, 2.72) |
| Georgia | 1967 to 1976 | 1.06 (0.39, 2.88) |
| Georgia | 1972 to 1981 | 1.07 (0.4, 2.82) |
| Georgia | 1977 to 1986 | 0.98 (0.36, 2.67) |
| Georgia | 1982 to 1991 | 1.00 (1.00, 1.00) |
| Georgia | 1987 to 1996 | 0.86 (0.24, 3.01) |
| Georgia | 1992 to 2001 | 0.94 (0.18, 4.9) |
| Georgia | 1997 to 2006 | 1.05 (0.11, 10.41) |
| Germany | 1952 to 1961 | 1.9 (1.46, 2.46) |
| Germany | 1957 to 1966 | 1.41 (1.13, 1.75) |
| Germany | 1962 to 1971 | 1.27 (1.04, 1.56) |
| Germany | 1967 to 1976 | 1.23 (1.01, 1.51) |
| Germany | 1972 to 1981 | 1.16 (0.95, 1.42) |
| Germany | 1977 to 1986 | 1.12 (0.92, 1.35) |
| Germany | 1982 to 1991 | 1.00 (1.00, 1.00) |
| Germany | 1987 to 1996 | 0.89 (0.68, 1.16) |
| Germany | 1992 to 2001 | 0.76 (0.53, 1.07) |
| Germany | 1997 to 2006 | 0.65 (0.39, 1.11) |
| Ghana | 1952 to 1961 | 14.36 (1.18, 175.02) |
| Ghana | 1957 to 1966 | 6.75 (0.81, 56.36) |
| Ghana | 1962 to 1971 | 4.74 (0.55, 41.24) |
| Ghana | 1967 to 1976 | 3.76 (0.38, 37.15) |
| Ghana | 1972 to 1981 | 2.32 (0.2, 27.13) |
| Ghana | 1977 to 1986 | 1.6 (0.17, 15.12) |
| Ghana | 1982 to 1991 | 1.00 (1.00, 1.00) |
| Ghana | 1987 to 1996 | 0.93 (0.04, 22.75) |
| Ghana | 1992 to 2001 | 0.63 (0.04, 10.18) |
| Ghana | 1997 to 2006 | 0.57 (0, 640.93) |
| Greece | 1952 to 1961 | 1.19 (0.71, 1.98) |
| Greece | 1957 to 1966 | 1.2 (0.8, 1.82) |
| Greece | 1962 to 1971 | 1.2 (0.83, 1.74) |
| Greece | 1967 to 1976 | 1.14 (0.81, 1.61) |
| Greece | 1972 to 1981 | 1.15 (0.82, 1.61) |
| Greece | 1977 to 1986 | 1.13 (0.8, 1.58) |
| Greece | 1982 to 1991 | 1.00 (1.00, 1.00) |
| Greece | 1987 to 1996 | 0.89 (0.55, 1.43) |
| Greece | 1992 to 2001 | 0.9 (0.49, 1.66) |
| Greece | 1997 to 2006 | 0.8 (0.3, 2.12) |
| Greenland | 1952 to 1961 | 2.09 (0.03, 152.8) |
| Greenland | 1957 to 1966 | 2.06 (0.04, 94.17) |
| Greenland | 1962 to 1971 | 2.08 (0.04, 105.34) |
| Greenland | 1967 to 1976 | 1.4 (0.02, 85.83) |
| Greenland | 1972 to 1981 | 1.58 (0.03, 85.19) |
| Greenland | 1977 to 1986 | 1.36 (0.03, 72.58) |
| Greenland | 1982 to 1991 | 1.00 (1.00, 1.00) |
| Greenland | 1987 to 1996 | 1.03 (0.01, 132.37) |
| Greenland | 1992 to 2001 | 1.05 (0, 320.75) |
| Greenland | 1997 to 2006 | 1.17 (0, 2614.65) |
| Guam | 1952 to 1961 | 0.84 (0.01, 80.87) |
| Guam | 1957 to 1966 | 0.8 (0.01, 84.58) |
| Guam | 1962 to 1971 | 1.19 (0.02, 80.1) |
| Guam | 1967 to 1976 | 0.82 (0.01, 52.21) |
| Guam | 1972 to 1981 | 0.96 (0.02, 53.26) |
| Guam | 1977 to 1986 | 1 (0.02, 55.2) |
| Guam | 1982 to 1991 | 1.00 (1.00, 1.00) |
| Guam | 1987 to 1996 | 0.98 (0.01, 125.42) |
| Guam | 1992 to 2001 | 0.98 (0, 298.19) |
| Guam | 1997 to 2006 | 1.07 (0, 2399.04) |
| Guatemala | 1952 to 1961 | 1.31 (0.31, 5.49) |
| Guatemala | 1957 to 1966 | 1.04 (0.33, 3.25) |
| Guatemala | 1962 to 1971 | 1.19 (0.47, 3.03) |
| Guatemala | 1967 to 1976 | 0.94 (0.38, 2.3) |
| Guatemala | 1972 to 1981 | 1.06 (0.49, 2.28) |
| Guatemala | 1977 to 1986 | 0.99 (0.47, 2.08) |
| Guatemala | 1982 to 1991 | 1.00 (1.00, 1.00) |
| Guatemala | 1987 to 1996 | 0.95 (0.39, 2.3) |
| Guatemala | 1992 to 2001 | 0.97 (0.34, 2.8) |
| Guatemala | 1997 to 2006 | 1.03 (0.24, 4.36) |
| Guinea | 1952 to 1961 | 1.28 (0.21, 7.91) |
| Guinea | 1957 to 1966 | 0.93 (0.21, 4.22) |
| Guinea | 1962 to 1971 | 1.13 (0.32, 3.96) |
| Guinea | 1967 to 1976 | 1.02 (0.32, 3.23) |
| Guinea | 1972 to 1981 | 1.06 (0.36, 3.17) |
| Guinea | 1977 to 1986 | 1.11 (0.4, 3.04) |
| Guinea | 1982 to 1991 | 1.00 (1.00, 1.00) |
| Guinea | 1987 to 1996 | 1.22 (0.38, 3.99) |
| Guinea | 1992 to 2001 | 1.32 (0.36, 4.82) |
| Guinea | 1997 to 2006 | 1.37 (0.21, 8.88) |
| Guinea-Bissau | 1952 to 1961 | 0.26 (0, 408.59) |
| Guinea-Bissau | 1957 to 1966 | 0.34 (0, 54.45) |
| Guinea-Bissau | 1962 to 1971 | 0.49 (0.01, 38.11) |
| Guinea-Bissau | 1967 to 1976 | 0.57 (0.01, 30.95) |
| Guinea-Bissau | 1972 to 1981 | 0.57 (0.01, 24.6) |
| Guinea-Bissau | 1977 to 1986 | 0.8 (0.03, 21.17) |
| Guinea-Bissau | 1982 to 1991 | 1.00 (1.00, 1.00) |
| Guinea-Bissau | 1987 to 1996 | 0.49 (0.01, 48.37) |
| Guinea-Bissau | 1992 to 2001 | 0.39 (0, 91.28) |
| Guinea-Bissau | 1997 to 2006 | 0.18 (0, 286.31) |
| Haiti | 1952 to 1961 | 0.9 (0.23, 3.55) |
| Haiti | 1957 to 1966 | 0.91 (0.32, 2.58) |
| Haiti | 1962 to 1971 | 0.89 (0.37, 2.13) |
| Haiti | 1967 to 1976 | 0.96 (0.44, 2.09) |
| Haiti | 1972 to 1981 | 0.98 (0.49, 1.97) |
| Haiti | 1977 to 1986 | 0.94 (0.48, 1.82) |
| Haiti | 1982 to 1991 | 1.00 (1.00, 1.00) |
| Haiti | 1987 to 1996 | 0.95 (0.43, 2.1) |
| Haiti | 1992 to 2001 | 1.08 (0.41, 2.87) |
| Haiti | 1997 to 2006 | 1.23 (0.34, 4.47) |
| Honduras | 1952 to 1961 | 1.34 (0.11, 15.77) |
| Honduras | 1957 to 1966 | 1.66 (0.26, 10.73) |
| Honduras | 1962 to 1971 | 2.08 (0.46, 9.41) |
| Honduras | 1967 to 1976 | 1.72 (0.43, 6.94) |
| Honduras | 1972 to 1981 | 1.39 (0.37, 5.19) |
| Honduras | 1977 to 1986 | 1.2 (0.32, 4.48) |
| Honduras | 1982 to 1991 | 1.00 (1.00, 1.00) |
| Honduras | 1987 to 1996 | 0.87 (0.19, 4.07) |
| Honduras | 1992 to 2001 | 0.79 (0.13, 4.83) |
| Honduras | 1997 to 2006 | 0.76 (0.06, 8.95) |
| Hungary | 1952 to 1961 | 1.09 (0.51, 2.31) |
| Hungary | 1957 to 1966 | 0.97 (0.49, 1.9) |
| Hungary | 1962 to 1971 | 0.83 (0.44, 1.57) |
| Hungary | 1967 to 1976 | 0.79 (0.43, 1.44) |
| Hungary | 1972 to 1981 | 0.82 (0.47, 1.45) |
| Hungary | 1977 to 1986 | 0.89 (0.51, 1.57) |
| Hungary | 1982 to 1991 | 1.00 (1.00, 1.00) |
| Hungary | 1987 to 1996 | 1.1 (0.52, 2.29) |
| Hungary | 1992 to 2001 | 1.22 (0.46, 3.21) |
| Hungary | 1997 to 2006 | 1.02 (0.21, 4.87) |
| Iceland | 1952 to 1961 | 0.74 (0.02, 26.3) |
| Iceland | 1957 to 1966 | 0.49 (0.01, 18.79) |
| Iceland | 1962 to 1971 | 0.57 (0.02, 19.54) |
| Iceland | 1967 to 1976 | 0.44 (0.01, 15.58) |
| Iceland | 1972 to 1981 | 0.67 (0.04, 12.03) |
| Iceland | 1977 to 1986 | 0.77 (0.07, 8.68) |
| Iceland | 1982 to 1991 | 1.00 (1.00, 1.00) |
| Iceland | 1987 to 1996 | 0.61 (0.01, 54.4) |
| Iceland | 1992 to 2001 | 0.75 (0, 143.84) |
| Iceland | 1997 to 2006 | 0.96 (0, 1195.96) |
| India | 1952 to 1961 | 1.09 (1.02, 1.16) |
| India | 1957 to 1966 | 1.02 (0.97, 1.07) |
| India | 1962 to 1971 | 0.99 (0.95, 1.04) |
| India | 1967 to 1976 | 0.99 (0.95, 1.04) |
| India | 1972 to 1981 | 0.99 (0.95, 1.03) |
| India | 1977 to 1986 | 0.98 (0.94, 1.02) |
| India | 1982 to 1991 | 1.00 (1.00, 1.00) |
| India | 1987 to 1996 | 0.96 (0.91, 1.01) |
| India | 1992 to 2001 | 0.92 (0.86, 0.98) |
| India | 1997 to 2006 | 0.89 (0.82, 0.97) |
| Indonesia | 1952 to 1961 | 1.04 (0.93, 1.16) |
| Indonesia | 1957 to 1966 | 1.02 (0.93, 1.11) |
| Indonesia | 1962 to 1971 | 1.01 (0.93, 1.1) |
| Indonesia | 1967 to 1976 | 1.01 (0.93, 1.09) |
| Indonesia | 1972 to 1981 | 1 (0.93, 1.08) |
| Indonesia | 1977 to 1986 | 0.99 (0.92, 1.07) |
| Indonesia | 1982 to 1991 | 1.00 (1.00, 1.00) |
| Indonesia | 1987 to 1996 | 0.98 (0.89, 1.08) |
| Indonesia | 1992 to 2001 | 0.98 (0.87, 1.11) |
| Indonesia | 1997 to 2006 | 1.01 (0.85, 1.21) |
| Iran (Islamic Republic of) | 1952 to 1961 | 0.77 (0.44, 1.33) |
| Iran (Islamic Republic of) | 1957 to 1966 | 0.76 (0.51, 1.13) |
| Iran (Islamic Republic of) | 1962 to 1971 | 0.78 (0.56, 1.1) |
| Iran (Islamic Republic of) | 1967 to 1976 | 0.81 (0.6, 1.1) |
| Iran (Islamic Republic of) | 1972 to 1981 | 0.88 (0.67, 1.14) |
| Iran (Islamic Republic of) | 1977 to 1986 | 0.94 (0.73, 1.2) |
| Iran (Islamic Republic of) | 1982 to 1991 | 1.00 (1.00, 1.00) |
| Iran (Islamic Republic of) | 1987 to 1996 | 1.07 (0.78, 1.46) |
| Iran (Islamic Republic of) | 1992 to 2001 | 1.22 (0.81, 1.84) |
| Iran (Islamic Republic of) | 1997 to 2006 | 1.27 (0.72, 2.23) |
| Iraq | 1952 to 1961 | 1.25 (0.68, 2.3) |
| Iraq | 1957 to 1966 | 1.17 (0.73, 1.88) |
| Iraq | 1962 to 1971 | 1.14 (0.75, 1.73) |
| Iraq | 1967 to 1976 | 1.12 (0.76, 1.63) |
| Iraq | 1972 to 1981 | 1.03 (0.72, 1.48) |
| Iraq | 1977 to 1986 | 1 (0.71, 1.42) |
| Iraq | 1982 to 1991 | 1.00 (1.00, 1.00) |
| Iraq | 1987 to 1996 | 0.99 (0.64, 1.52) |
| Iraq | 1992 to 2001 | 0.97 (0.57, 1.65) |
| Iraq | 1997 to 2006 | 1.18 (0.59, 2.33) |
| Ireland | 1952 to 1961 | 0.62 (0.21, 1.89) |
| Ireland | 1957 to 1966 | 0.65 (0.28, 1.5) |
| Ireland | 1962 to 1971 | 0.63 (0.29, 1.38) |
| Ireland | 1967 to 1976 | 0.7 (0.35, 1.4) |
| Ireland | 1972 to 1981 | 0.79 (0.42, 1.47) |
| Ireland | 1977 to 1986 | 0.9 (0.5, 1.61) |
| Ireland | 1982 to 1991 | 1.00 (1.00, 1.00) |
| Ireland | 1987 to 1996 | 1.01 (0.46, 2.22) |
| Ireland | 1992 to 2001 | 0.73 (0.23, 2.27) |
| Ireland | 1997 to 2006 | 0.82 (0.17, 4) |
| Israel | 1952 to 1961 | 1.05 (0.41, 2.72) |
| Israel | 1957 to 1966 | 0.99 (0.47, 2.1) |
| Israel | 1962 to 1971 | 0.98 (0.51, 1.88) |
| Israel | 1967 to 1976 | 1.01 (0.56, 1.81) |
| Israel | 1972 to 1981 | 1 (0.58, 1.74) |
| Israel | 1977 to 1986 | 0.98 (0.58, 1.67) |
| Israel | 1982 to 1991 | 1.00 (1.00, 1.00) |
| Israel | 1987 to 1996 | 0.99 (0.51, 1.92) |
| Israel | 1992 to 2001 | 0.93 (0.41, 2.12) |
| Israel | 1997 to 2006 | 0.9 (0.27, 2.95) |
| Italy | 1952 to 1961 | 1.24 (0.98, 1.56) |
| Italy | 1957 to 1966 | 1.27 (1.06, 1.52) |
| Italy | 1962 to 1971 | 1.2 (1.01, 1.42) |
| Italy | 1967 to 1976 | 1.1 (0.94, 1.3) |
| Italy | 1972 to 1981 | 1.07 (0.91, 1.25) |
| Italy | 1977 to 1986 | 1.02 (0.86, 1.19) |
| Italy | 1982 to 1991 | 1.00 (1.00, 1.00) |
| Italy | 1987 to 1996 | 0.93 (0.75, 1.15) |
| Italy | 1992 to 2001 | 0.82 (0.62, 1.08) |
| Italy | 1997 to 2006 | 0.77 (0.5, 1.16) |
| Jamaica | 1952 to 1961 | 0.58 (0.05, 6.45) |
| Jamaica | 1957 to 1966 | 0.82 (0.16, 4.22) |
| Jamaica | 1962 to 1971 | 0.65 (0.14, 2.95) |
| Jamaica | 1967 to 1976 | 0.88 (0.23, 3.35) |
| Jamaica | 1972 to 1981 | 0.83 (0.21, 3.24) |
| Jamaica | 1977 to 1986 | 0.99 (0.3, 3.26) |
| Jamaica | 1982 to 1991 | 1.00 (1.00, 1.00) |
| Jamaica | 1987 to 1996 | 0.81 (0.18, 3.67) |
| Jamaica | 1992 to 2001 | 0.84 (0.14, 5.08) |
| Jamaica | 1997 to 2006 | 1.05 (0.09, 12.93) |
| Japan | 1952 to 1961 | 0.67 (0.5, 0.89) |
| Japan | 1957 to 1966 | 0.69 (0.56, 0.86) |
| Japan | 1962 to 1971 | 0.76 (0.63, 0.91) |
| Japan | 1967 to 1976 | 0.79 (0.67, 0.94) |
| Japan | 1972 to 1981 | 0.86 (0.73, 1.01) |
| Japan | 1977 to 1986 | 0.91 (0.77, 1.07) |
| Japan | 1982 to 1991 | 1.00 (1.00, 1.00) |
| Japan | 1987 to 1996 | 1.05 (0.85, 1.29) |
| Japan | 1992 to 2001 | 1.07 (0.83, 1.39) |
| Japan | 1997 to 2006 | 1.15 (0.76, 1.74) |
| Jordan | 1952 to 1961 | 1.04 (0.4, 2.69) |
| Jordan | 1957 to 1966 | 0.99 (0.5, 1.97) |
| Jordan | 1962 to 1971 | 0.92 (0.5, 1.66) |
| Jordan | 1967 to 1976 | 0.94 (0.55, 1.6) |
| Jordan | 1972 to 1981 | 0.93 (0.57, 1.51) |
| Jordan | 1977 to 1986 | 0.98 (0.63, 1.51) |
| Jordan | 1982 to 1991 | 1.00 (1.00, 1.00) |
| Jordan | 1987 to 1996 | 1.07 (0.63, 1.81) |
| Jordan | 1992 to 2001 | 1.03 (0.53, 2) |
| Jordan | 1997 to 2006 | 1.02 (0.41, 2.57) |
| Kazakhstan | 1952 to 1961 | 0.72 (0.35, 1.48) |
| Kazakhstan | 1957 to 1966 | 0.75 (0.43, 1.29) |
| Kazakhstan | 1962 to 1971 | 0.8 (0.49, 1.31) |
| Kazakhstan | 1967 to 1976 | 0.86 (0.55, 1.35) |
| Kazakhstan | 1972 to 1981 | 0.98 (0.65, 1.47) |
| Kazakhstan | 1977 to 1986 | 1.01 (0.68, 1.5) |
| Kazakhstan | 1982 to 1991 | 1.00 (1.00, 1.00) |
| Kazakhstan | 1987 to 1996 | 0.99 (0.62, 1.59) |
| Kazakhstan | 1992 to 2001 | 1.17 (0.63, 2.17) |
| Kazakhstan | 1997 to 2006 | 1.38 (0.59, 3.24) |
| Kenya | 1952 to 1961 | 0.78 (0.55, 1.1) |
| Kenya | 1957 to 1966 | 0.84 (0.64, 1.09) |
| Kenya | 1962 to 1971 | 0.88 (0.7, 1.11) |
| Kenya | 1967 to 1976 | 0.91 (0.74, 1.13) |
| Kenya | 1972 to 1981 | 0.93 (0.77, 1.13) |
| Kenya | 1977 to 1986 | 0.96 (0.8, 1.15) |
| Kenya | 1982 to 1991 | 1.00 (1.00, 1.00) |
| Kenya | 1987 to 1996 | 1.03 (0.82, 1.28) |
| Kenya | 1992 to 2001 | 1.07 (0.82, 1.4) |
| Kenya | 1997 to 2006 | 1.14 (0.81, 1.61) |
| Kuwait | 1952 to 1961 | 1.81 (0.52, 6.38) |
| Kuwait | 1957 to 1966 | 1.56 (0.63, 3.88) |
| Kuwait | 1962 to 1971 | 1.44 (0.63, 3.26) |
| Kuwait | 1967 to 1976 | 1.18 (0.51, 2.73) |
| Kuwait | 1972 to 1981 | 1.19 (0.56, 2.53) |
| Kuwait | 1977 to 1986 | 1.07 (0.54, 2.15) |
| Kuwait | 1982 to 1991 | 1.00 (1.00, 1.00) |
| Kuwait | 1987 to 1996 | 1 (0.39, 2.58) |
| Kuwait | 1992 to 2001 | 0.78 (0.2, 3.01) |
| Kuwait | 1997 to 2006 | 1.28 (0.22, 7.47) |
| Kyrgyzstan | 1952 to 1961 | 0.61 (0.15, 2.55) |
| Kyrgyzstan | 1957 to 1966 | 0.51 (0.17, 1.56) |
| Kyrgyzstan | 1962 to 1971 | 0.61 (0.23, 1.61) |
| Kyrgyzstan | 1967 to 1976 | 0.67 (0.26, 1.69) |
| Kyrgyzstan | 1972 to 1981 | 0.81 (0.36, 1.83) |
| Kyrgyzstan | 1977 to 1986 | 0.93 (0.44, 1.94) |
| Kyrgyzstan | 1982 to 1991 | 1.00 (1.00, 1.00) |
| Kyrgyzstan | 1987 to 1996 | 1.08 (0.45, 2.55) |
| Kyrgyzstan | 1992 to 2001 | 1.32 (0.44, 3.92) |
| Kyrgyzstan | 1997 to 2006 | 1.54 (0.34, 6.87) |
| Lao People's Democratic Republic | 1952 to 1961 | 1.17 (0.55, 2.49) |
| Lao People's Democratic Republic | 1957 to 1966 | 1.12 (0.62, 2.04) |
| Lao People's Democratic Republic | 1962 to 1971 | 1.1 (0.63, 1.92) |
| Lao People's Democratic Republic | 1967 to 1976 | 1.02 (0.59, 1.75) |
| Lao People's Democratic Republic | 1972 to 1981 | 1.04 (0.63, 1.71) |
| Lao People's Democratic Republic | 1977 to 1986 | 1.01 (0.64, 1.62) |
| Lao People's Democratic Republic | 1982 to 1991 | 1.00 (1.00, 1.00) |
| Lao People's Democratic Republic | 1987 to 1996 | 1.08 (0.6, 1.92) |
| Lao People's Democratic Republic | 1992 to 2001 | 1.03 (0.49, 2.19) |
| Lao People's Democratic Republic | 1997 to 2006 | 0.99 (0.34, 2.87) |
| Latvia | 1952 to 1961 | 3.81 (0.44, 32.8) |
| Latvia | 1957 to 1966 | 2.76 (0.45, 16.97) |
| Latvia | 1962 to 1971 | 1.48 (0.24, 9.22) |
| Latvia | 1967 to 1976 | 1.5 (0.24, 9.5) |
| Latvia | 1972 to 1981 | 1.38 (0.23, 8.23) |
| Latvia | 1977 to 1986 | 1.4 (0.25, 7.95) |
| Latvia | 1982 to 1991 | 1.00 (1.00, 1.00) |
| Latvia | 1987 to 1996 | 0.18 (0, 9.44) |
| Latvia | 1992 to 2001 | 0.28 (0, 30.17) |
| Latvia | 1997 to 2006 | 0.24 (0, 146.36) |
| Lebanon | 1952 to 1961 | 0.79 (0.19, 3.24) |
| Lebanon | 1957 to 1966 | 0.66 (0.23, 1.91) |
| Lebanon | 1962 to 1971 | 0.7 (0.26, 1.86) |
| Lebanon | 1967 to 1976 | 0.78 (0.32, 1.89) |
| Lebanon | 1972 to 1981 | 0.78 (0.35, 1.76) |
| Lebanon | 1977 to 1986 | 0.82 (0.39, 1.69) |
| Lebanon | 1982 to 1991 | 1.00 (1.00, 1.00) |
| Lebanon | 1987 to 1996 | 1.24 (0.52, 2.97) |
| Lebanon | 1992 to 2001 | 1.47 (0.46, 4.75) |
| Lebanon | 1997 to 2006 | 1.54 (0.27, 8.87) |
| Lesotho | 1952 to 1961 | 0.45 (0.02, 10.35) |
| Lesotho | 1957 to 1966 | 0.34 (0.01, 9.3) |
| Lesotho | 1962 to 1971 | 0.18 (0.01, 3.46) |
| Lesotho | 1967 to 1976 | 0.15 (0.01, 2.16) |
| Lesotho | 1972 to 1981 | 0.27 (0.03, 2.11) |
| Lesotho | 1977 to 1986 | 0.44 (0.08, 2.41) |
| Lesotho | 1982 to 1991 | 1.00 (1.00, 1.00) |
| Lesotho | 1987 to 1996 | 1.42 (0.26, 7.65) |
| Lesotho | 1992 to 2001 | 2.39 (0.27, 21.64) |
| Lesotho | 1997 to 2006 | 3.71 (0.16, 84.42) |
| Liberia | 1952 to 1961 | 0.14 (0, 116.94) |
| Liberia | 1957 to 1966 | 0.19 (0, 23.03) |
| Liberia | 1962 to 1971 | 0.49 (0.01, 30.46) |
| Liberia | 1967 to 1976 | 0.65 (0.04, 12.18) |
| Liberia | 1972 to 1981 | 0.76 (0.07, 8.48) |
| Liberia | 1977 to 1986 | 0.81 (0.09, 7.42) |
| Liberia | 1982 to 1991 | 1.00 (1.00, 1.00) |
| Liberia | 1987 to 1996 | 1.03 (0.07, 15.85) |
| Liberia | 1992 to 2001 | 1.24 (0.07, 21.57) |
| Liberia | 1997 to 2006 | 0.63 (0, 719.93) |
| Libya | 1952 to 1961 | 0.66 (0.41, 1.08) |
| Libya | 1957 to 1966 | 0.67 (0.47, 0.97) |
| Libya | 1962 to 1971 | 0.69 (0.5, 0.95) |
| Libya | 1967 to 1976 | 0.71 (0.53, 0.95) |
| Libya | 1972 to 1981 | 0.77 (0.59, 1) |
| Libya | 1977 to 1986 | 0.88 (0.69, 1.11) |
| Libya | 1982 to 1991 | 1.00 (1.00, 1.00) |
| Libya | 1987 to 1996 | 1.12 (0.82, 1.54) |
| Libya | 1992 to 2001 | 1.24 (0.83, 1.86) |
| Libya | 1997 to 2006 | 1.39 (0.79, 2.42) |
| Lithuania | 1952 to 1961 | 2.42 (0.4, 14.52) |
| Lithuania | 1957 to 1966 | 1.88 (0.42, 8.42) |
| Lithuania | 1962 to 1971 | 1.71 (0.44, 6.66) |
| Lithuania | 1967 to 1976 | 1.19 (0.32, 4.45) |
| Lithuania | 1972 to 1981 | 1.13 (0.32, 4.02) |
| Lithuania | 1977 to 1986 | 0.97 (0.25, 3.66) |
| Lithuania | 1982 to 1991 | 1.00 (1.00, 1.00) |
| Lithuania | 1987 to 1996 | 0.83 (0.14, 5.08) |
| Lithuania | 1992 to 2001 | 0.98 (0.09, 10.85) |
| Lithuania | 1997 to 2006 | 0.25 (0, 149.12) |
| Luxembourg | 1952 to 1961 | 1.88 (0.09, 38.54) |
| Luxembourg | 1957 to 1966 | 1.68 (0.16, 17.93) |
| Luxembourg | 1962 to 1971 | 1.9 (0.2, 18.04) |
| Luxembourg | 1967 to 1976 | 1.38 (0.13, 14.73) |
| Luxembourg | 1972 to 1981 | 1.39 (0.12, 16.04) |
| Luxembourg | 1977 to 1986 | 1.35 (0.14, 13.28) |
| Luxembourg | 1982 to 1991 | 1.00 (1.00, 1.00) |
| Luxembourg | 1987 to 1996 | 0.46 (0.01, 33.39) |
| Luxembourg | 1992 to 2001 | 0.85 (0, 150.25) |
| Luxembourg | 1997 to 2006 | 0.81 (0, 973.57) |
| Madagascar | 1952 to 1961 | 1.14 (0.64, 2.02) |
| Madagascar | 1957 to 1966 | 1.09 (0.69, 1.71) |
| Madagascar | 1962 to 1971 | 1.05 (0.7, 1.57) |
| Madagascar | 1967 to 1976 | 1.02 (0.7, 1.48) |
| Madagascar | 1972 to 1981 | 1.01 (0.72, 1.42) |
| Madagascar | 1977 to 1986 | 1 (0.73, 1.39) |
| Madagascar | 1982 to 1991 | 1.00 (1.00, 1.00) |
| Madagascar | 1987 to 1996 | 1.01 (0.69, 1.49) |
| Madagascar | 1992 to 2001 | 1.02 (0.65, 1.6) |
| Madagascar | 1997 to 2006 | 1.03 (0.57, 1.84) |
| Malawi | 1952 to 1961 | 1.06 (0.29, 3.89) |
| Malawi | 1957 to 1966 | 0.91 (0.31, 2.69) |
| Malawi | 1962 to 1971 | 1.04 (0.41, 2.61) |
| Malawi | 1967 to 1976 | 1.03 (0.44, 2.44) |
| Malawi | 1972 to 1981 | 1.08 (0.49, 2.39) |
| Malawi | 1977 to 1986 | 1.02 (0.48, 2.18) |
| Malawi | 1982 to 1991 | 1.00 (1.00, 1.00) |
| Malawi | 1987 to 1996 | 1.11 (0.45, 2.76) |
| Malawi | 1992 to 2001 | 1.01 (0.34, 2.98) |
| Malawi | 1997 to 2006 | 0.98 (0.26, 3.76) |
| Malaysia | 1952 to 1961 | 0.74 (0.64, 0.86) |
| Malaysia | 1957 to 1966 | 0.78 (0.7, 0.88) |
| Malaysia | 1962 to 1971 | 0.83 (0.75, 0.92) |
| Malaysia | 1967 to 1976 | 0.86 (0.78, 0.96) |
| Malaysia | 1972 to 1981 | 0.9 (0.82, 1) |
| Malaysia | 1977 to 1986 | 0.95 (0.87, 1.03) |
| Malaysia | 1982 to 1991 | 1.00 (1.00, 1.00) |
| Malaysia | 1987 to 1996 | 1.04 (0.93, 1.17) |
| Malaysia | 1992 to 2001 | 1.12 (0.96, 1.3) |
| Malaysia | 1997 to 2006 | 1.11 (0.89, 1.38) |
| Maldives | 1952 to 1961 | 0.29 (0, 357.36) |
| Maldives | 1957 to 1966 | 0.4 (0, 56.42) |
| Maldives | 1962 to 1971 | 0.58 (0.01, 42.26) |
| Maldives | 1967 to 1976 | 0.69 (0.01, 35.99) |
| Maldives | 1972 to 1981 | 1.23 (0.03, 46.08) |
| Maldives | 1977 to 1986 | 0.61 (0.03, 14.27) |
| Maldives | 1982 to 1991 | 1.00 (1.00, 1.00) |
| Maldives | 1987 to 1996 | 0.5 (0.01, 48.69) |
| Maldives | 1992 to 2001 | 0.5 (0, 114.92) |
| Maldives | 1997 to 2006 | 0.49 (0, 777.27) |
| Mali | 1952 to 1961 | 0.77 (0.07, 8.03) |
| Mali | 1957 to 1966 | 0.86 (0.16, 4.65) |
| Mali | 1962 to 1971 | 1.33 (0.34, 5.27) |
| Mali | 1967 to 1976 | 1.14 (0.31, 4.12) |
| Mali | 1972 to 1981 | 1.18 (0.37, 3.8) |
| Mali | 1977 to 1986 | 1.07 (0.35, 3.26) |
| Mali | 1982 to 1991 | 1.00 (1.00, 1.00) |
| Mali | 1987 to 1996 | 0.93 (0.23, 3.73) |
| Mali | 1992 to 2001 | 0.99 (0.23, 4.2) |
| Mali | 1997 to 2006 | 0.91 (0.14, 6.11) |
| Malta | 1952 to 1961 | 0.71 (0.11, 4.67) |
| Malta | 1957 to 1966 | 0.73 (0.15, 3.56) |
| Malta | 1962 to 1971 | 0.86 (0.21, 3.51) |
| Malta | 1967 to 1976 | 1.1 (0.32, 3.77) |
| Malta | 1972 to 1981 | 1.01 (0.31, 3.32) |
| Malta | 1977 to 1986 | 1.14 (0.37, 3.54) |
| Malta | 1982 to 1991 | 1.00 (1.00, 1.00) |
| Malta | 1987 to 1996 | 0.96 (0.22, 4.26) |
| Malta | 1992 to 2001 | 1.28 (0.22, 7.38) |
| Malta | 1997 to 2006 | 0.16 (0, 88.2) |
| Mauritania | 1952 to 1961 | 0.15 (0, 177.39) |
| Mauritania | 1957 to 1966 | 0.2 (0, 27.51) |
| Mauritania | 1962 to 1971 | 0.36 (0, 25.69) |
| Mauritania | 1967 to 1976 | 0.46 (0.01, 23.76) |
| Mauritania | 1972 to 1981 | 1.01 (0.03, 36.89) |
| Mauritania | 1977 to 1986 | 1.1 (0.08, 14.65) |
| Mauritania | 1982 to 1991 | 1.00 (1.00, 1.00) |
| Mauritania | 1987 to 1996 | 0.58 (0.01, 55.45) |
| Mauritania | 1992 to 2001 | 2.31 (0.05, 97.36) |
| Mauritania | 1997 to 2006 | 0.47 (0, 625) |
| Mauritius | 1952 to 1961 | 0.76 (0.16, 3.57) |
| Mauritius | 1957 to 1966 | 0.66 (0.18, 2.43) |
| Mauritius | 1962 to 1971 | 0.7 (0.19, 2.58) |
| Mauritius | 1967 to 1976 | 0.77 (0.22, 2.67) |
| Mauritius | 1972 to 1981 | 0.8 (0.24, 2.7) |
| Mauritius | 1977 to 1986 | 0.92 (0.3, 2.82) |
| Mauritius | 1982 to 1991 | 1.00 (1.00, 1.00) |
| Mauritius | 1987 to 1996 | 1.51 (0.36, 6.37) |
| Mauritius | 1992 to 2001 | 1.5 (0.22, 10.4) |
| Mauritius | 1997 to 2006 | 2.05 (0.12, 34.78) |
| Mexico | 1952 to 1961 | 0.78 (0.44, 1.39) |
| Mexico | 1957 to 1966 | 0.78 (0.5, 1.21) |
| Mexico | 1962 to 1971 | 0.8 (0.55, 1.18) |
| Mexico | 1967 to 1976 | 0.85 (0.6, 1.2) |
| Mexico | 1972 to 1981 | 0.87 (0.63, 1.21) |
| Mexico | 1977 to 1986 | 0.92 (0.67, 1.27) |
| Mexico | 1982 to 1991 | 1.00 (1.00, 1.00) |
| Mexico | 1987 to 1996 | 1.11 (0.77, 1.61) |
| Mexico | 1992 to 2001 | 1.17 (0.74, 1.83) |
| Mexico | 1997 to 2006 | 1.12 (0.59, 2.11) |
| Mongolia | 1952 to 1961 | 0.69 (0.06, 7.38) |
| Mongolia | 1957 to 1966 | 0.67 (0.12, 3.77) |
| Mongolia | 1962 to 1971 | 0.73 (0.17, 3.11) |
| Mongolia | 1967 to 1976 | 0.99 (0.27, 3.53) |
| Mongolia | 1972 to 1981 | 0.87 (0.27, 2.82) |
| Mongolia | 1977 to 1986 | 0.95 (0.31, 2.94) |
| Mongolia | 1982 to 1991 | 1.00 (1.00, 1.00) |
| Mongolia | 1987 to 1996 | 1.03 (0.27, 3.84) |
| Mongolia | 1992 to 2001 | 0.96 (0.17, 5.44) |
| Mongolia | 1997 to 2006 | 1.06 (0.1, 11.59) |
| Morocco | 1952 to 1961 | 1.21 (0.83, 1.75) |
| Morocco | 1957 to 1966 | 1.15 (0.85, 1.55) |
| Morocco | 1962 to 1971 | 1.1 (0.84, 1.45) |
| Morocco | 1967 to 1976 | 1.04 (0.8, 1.36) |
| Morocco | 1972 to 1981 | 1.02 (0.79, 1.31) |
| Morocco | 1977 to 1986 | 1.02 (0.8, 1.3) |
| Morocco | 1982 to 1991 | 1.00 (1.00, 1.00) |
| Morocco | 1987 to 1996 | 1.02 (0.75, 1.38) |
| Morocco | 1992 to 2001 | 1.11 (0.76, 1.61) |
| Morocco | 1997 to 2006 | 1.26 (0.77, 2.05) |
| Mozambique | 1952 to 1961 | 1.07 (0.06, 18.68) |
| Mozambique | 1957 to 1966 | 0.42 (0.02, 10.31) |
| Mozambique | 1962 to 1971 | 0.69 (0.05, 9.28) |
| Mozambique | 1967 to 1976 | 0.87 (0.07, 10.37) |
| Mozambique | 1972 to 1981 | 0.82 (0.09, 7.3) |
| Mozambique | 1977 to 1986 | 0.93 (0.15, 5.56) |
| Mozambique | 1982 to 1991 | 1.00 (1.00, 1.00) |
| Mozambique | 1987 to 1996 | 1.07 (0.09, 12.88) |
| Mozambique | 1992 to 2001 | 4.41 (0.16, 125.39) |
| Mozambique | 1997 to 2006 | 9.39 (0.2, 442.61) |
| Myanmar | 1952 to 1961 | 1.38 (1.09, 1.77) |
| Myanmar | 1957 to 1966 | 1.28 (1.05, 1.55) |
| Myanmar | 1962 to 1971 | 1.21 (1.01, 1.45) |
| Myanmar | 1967 to 1976 | 1.16 (0.97, 1.38) |
| Myanmar | 1972 to 1981 | 1.1 (0.92, 1.3) |
| Myanmar | 1977 to 1986 | 1.03 (0.87, 1.22) |
| Myanmar | 1982 to 1991 | 1.00 (1.00, 1.00) |
| Myanmar | 1987 to 1996 | 0.93 (0.74, 1.16) |
| Myanmar | 1992 to 2001 | 0.92 (0.69, 1.21) |
| Myanmar | 1997 to 2006 | 0.91 (0.62, 1.33) |
| Namibia | 1952 to 1961 | 1.04 (0.09, 12.26) |
| Namibia | 1957 to 1966 | 1.78 (0.32, 10.02) |
| Namibia | 1962 to 1971 | 1.55 (0.34, 6.98) |
| Namibia | 1967 to 1976 | 1.36 (0.34, 5.46) |
| Namibia | 1972 to 1981 | 1.22 (0.33, 4.54) |
| Namibia | 1977 to 1986 | 1.1 (0.3, 4.1) |
| Namibia | 1982 to 1991 | 1.00 (1.00, 1.00) |
| Namibia | 1987 to 1996 | 0.92 (0.2, 4.3) |
| Namibia | 1992 to 2001 | 0.93 (0.15, 5.69) |
| Namibia | 1997 to 2006 | 0.93 (0.08, 10.95) |
| Nepal | 1952 to 1961 | 0.94 (0.59, 1.5) |
| Nepal | 1957 to 1966 | 0.95 (0.66, 1.37) |
| Nepal | 1962 to 1971 | 0.95 (0.69, 1.33) |
| Nepal | 1967 to 1976 | 0.94 (0.69, 1.28) |
| Nepal | 1972 to 1981 | 0.95 (0.71, 1.27) |
| Nepal | 1977 to 1986 | 0.98 (0.74, 1.29) |
| Nepal | 1982 to 1991 | 1.00 (1.00, 1.00) |
| Nepal | 1987 to 1996 | 1 (0.71, 1.4) |
| Nepal | 1992 to 2001 | 0.99 (0.66, 1.48) |
| Nepal | 1997 to 2006 | 0.99 (0.57, 1.71) |
| Netherlands | 1952 to 1961 | 1.08 (0.72, 1.61) |
| Netherlands | 1957 to 1966 | 1.06 (0.77, 1.46) |
| Netherlands | 1962 to 1971 | 1.02 (0.76, 1.36) |
| Netherlands | 1967 to 1976 | 1.03 (0.77, 1.38) |
| Netherlands | 1972 to 1981 | 1.01 (0.76, 1.35) |
| Netherlands | 1977 to 1986 | 0.99 (0.75, 1.32) |
| Netherlands | 1982 to 1991 | 1.00 (1.00, 1.00) |
| Netherlands | 1987 to 1996 | 0.95 (0.66, 1.37) |
| Netherlands | 1992 to 2001 | 0.86 (0.53, 1.4) |
| Netherlands | 1997 to 2006 | 0.82 (0.42, 1.59) |
| New Zealand | 1952 to 1961 | 1.46 (0.72, 3) |
| New Zealand | 1957 to 1966 | 1.42 (0.83, 2.42) |
| New Zealand | 1962 to 1971 | 1.46 (0.88, 2.42) |
| New Zealand | 1967 to 1976 | 1.38 (0.84, 2.28) |
| New Zealand | 1972 to 1981 | 1.21 (0.73, 2.01) |
| New Zealand | 1977 to 1986 | 1.11 (0.68, 1.82) |
| New Zealand | 1982 to 1991 | 1.00 (1.00, 1.00) |
| New Zealand | 1987 to 1996 | 0.84 (0.43, 1.67) |
| New Zealand | 1992 to 2001 | 0.7 (0.26, 1.92) |
| New Zealand | 1997 to 2006 | 0.33 (0.04, 2.65) |
| Nicaragua | 1952 to 1961 | 0.75 (0.07, 8.13) |
| Nicaragua | 1957 to 1966 | 0.9 (0.16, 5.2) |
| Nicaragua | 1962 to 1971 | 1.29 (0.31, 5.33) |
| Nicaragua | 1967 to 1976 | 1.2 (0.32, 4.49) |
| Nicaragua | 1972 to 1981 | 1.02 (0.29, 3.56) |
| Nicaragua | 1977 to 1986 | 1.15 (0.36, 3.72) |
| Nicaragua | 1982 to 1991 | 1.00 (1.00, 1.00) |
| Nicaragua | 1987 to 1996 | 0.84 (0.19, 3.78) |
| Nicaragua | 1992 to 2001 | 0.81 (0.14, 4.81) |
| Nicaragua | 1997 to 2006 | 0.82 (0.07, 8.96) |
| Niger | 1952 to 1961 | 0.6 (0.05, 6.74) |
| Niger | 1957 to 1966 | 0.71 (0.13, 3.96) |
| Niger | 1962 to 1971 | 0.9 (0.15, 5.23) |
| Niger | 1967 to 1976 | 0.83 (0.19, 3.63) |
| Niger | 1972 to 1981 | 0.99 (0.26, 3.7) |
| Niger | 1977 to 1986 | 0.95 (0.28, 3.19) |
| Niger | 1982 to 1991 | 1.00 (1.00, 1.00) |
| Niger | 1987 to 1996 | 0.98 (0.2, 4.81) |
| Niger | 1992 to 2001 | 1.18 (0.2, 6.8) |
| Niger | 1997 to 2006 | 1.5 (0.07, 33.18) |
| Nigeria | 1952 to 1961 | 0.9 (0.7, 1.16) |
| Nigeria | 1957 to 1966 | 0.9 (0.74, 1.08) |
| Nigeria | 1962 to 1971 | 0.91 (0.77, 1.09) |
| Nigeria | 1967 to 1976 | 0.93 (0.8, 1.09) |
| Nigeria | 1972 to 1981 | 0.95 (0.82, 1.1) |
| Nigeria | 1977 to 1986 | 0.97 (0.84, 1.11) |
| Nigeria | 1982 to 1991 | 1.00 (1.00, 1.00) |
| Nigeria | 1987 to 1996 | 1.02 (0.87, 1.21) |
| Nigeria | 1992 to 2001 | 1.04 (0.86, 1.27) |
| Nigeria | 1997 to 2006 | 1.02 (0.76, 1.37) |
| North Macedonia | 1952 to 1961 | 0.55 (0.04, 6.94) |
| North Macedonia | 1957 to 1966 | 0.67 (0.1, 4.59) |
| North Macedonia | 1962 to 1971 | 0.62 (0.08, 4.93) |
| North Macedonia | 1967 to 1976 | 0.7 (0.12, 4.11) |
| North Macedonia | 1972 to 1981 | 0.63 (0.12, 3.28) |
| North Macedonia | 1977 to 1986 | 0.83 (0.2, 3.46) |
| North Macedonia | 1982 to 1991 | 1.00 (1.00, 1.00) |
| North Macedonia | 1987 to 1996 | 1.33 (0.27, 6.62) |
| North Macedonia | 1992 to 2001 | 1.4 (0.1, 18.79) |
| North Macedonia | 1997 to 2006 | 0.28 (0, 194.28) |
| Norway | 1952 to 1961 | 1.53 (0.33, 7.12) |
| Norway | 1957 to 1966 | 1.51 (0.49, 4.68) |
| Norway | 1962 to 1971 | 1.15 (0.44, 2.99) |
| Norway | 1967 to 1976 | 1.03 (0.42, 2.5) |
| Norway | 1972 to 1981 | 1.23 (0.54, 2.78) |
| Norway | 1977 to 1986 | 1.08 (0.47, 2.51) |
| Norway | 1982 to 1991 | 1.00 (1.00, 1.00) |
| Norway | 1987 to 1996 | 1.01 (0.39, 2.64) |
| Norway | 1992 to 2001 | 1.08 (0.3, 3.84) |
| Norway | 1997 to 2006 | 1.17 (0.21, 6.6) |
| Oman | 1952 to 1961 | 0.87 (0.15, 4.9) |
| Oman | 1957 to 1966 | 0.8 (0.19, 3.28) |
| Oman | 1962 to 1971 | 0.82 (0.24, 2.86) |
| Oman | 1967 to 1976 | 1 (0.34, 2.99) |
| Oman | 1972 to 1981 | 0.94 (0.35, 2.54) |
| Oman | 1977 to 1986 | 0.95 (0.41, 2.23) |
| Oman | 1982 to 1991 | 1.00 (1.00, 1.00) |
| Oman | 1987 to 1996 | 1.15 (0.41, 3.23) |
| Oman | 1992 to 2001 | 1.12 (0.26, 4.73) |
| Oman | 1997 to 2006 | 0.91 (0.09, 8.92) |
| Pakistan | 1952 to 1961 | 0.98 (0.83, 1.15) |
| Pakistan | 1957 to 1966 | 0.95 (0.84, 1.09) |
| Pakistan | 1962 to 1971 | 0.94 (0.84, 1.05) |
| Pakistan | 1967 to 1976 | 0.93 (0.84, 1.04) |
| Pakistan | 1972 to 1981 | 0.95 (0.87, 1.05) |
| Pakistan | 1977 to 1986 | 0.97 (0.89, 1.06) |
| Pakistan | 1982 to 1991 | 1.00 (1.00, 1.00) |
| Pakistan | 1987 to 1996 | 1.02 (0.92, 1.14) |
| Pakistan | 1992 to 2001 | 1.05 (0.92, 1.2) |
| Pakistan | 1997 to 2006 | 1.08 (0.9, 1.28) |
| Palestine | 1952 to 1961 | 1.5 (0.13, 16.81) |
| Palestine | 1957 to 1966 | 1.27 (0.22, 7.39) |
| Palestine | 1962 to 1971 | 1.24 (0.28, 5.51) |
| Palestine | 1967 to 1976 | 1.24 (0.32, 4.84) |
| Palestine | 1972 to 1981 | 1.3 (0.37, 4.58) |
| Palestine | 1977 to 1986 | 1.26 (0.38, 4.12) |
| Palestine | 1982 to 1991 | 1.00 (1.00, 1.00) |
| Palestine | 1987 to 1996 | 0.95 (0.24, 3.82) |
| Palestine | 1992 to 2001 | 0.64 (0.11, 3.75) |
| Palestine | 1997 to 2006 | 0.57 (0.05, 6.31) |
| Panama | 1952 to 1961 | 0.64 (0.06, 6.76) |
| Panama | 1957 to 1966 | 0.63 (0.11, 3.5) |
| Panama | 1962 to 1971 | 0.82 (0.21, 3.22) |
| Panama | 1967 to 1976 | 0.96 (0.29, 3.21) |
| Panama | 1972 to 1981 | 0.93 (0.3, 2.89) |
| Panama | 1977 to 1986 | 0.87 (0.28, 2.7) |
| Panama | 1982 to 1991 | 1.00 (1.00, 1.00) |
| Panama | 1987 to 1996 | 1.02 (0.28, 3.79) |
| Panama | 1992 to 2001 | 0.75 (0.13, 4.2) |
| Panama | 1997 to 2006 | 0.77 (0.07, 8.35) |
| Papua New Guinea | 1952 to 1961 | 1.24 (0.35, 4.34) |
| Papua New Guinea | 1957 to 1966 | 0.95 (0.32, 2.86) |
| Papua New Guinea | 1962 to 1971 | 1.03 (0.36, 2.95) |
| Papua New Guinea | 1967 to 1976 | 0.97 (0.39, 2.43) |
| Papua New Guinea | 1972 to 1981 | 0.87 (0.34, 2.21) |
| Papua New Guinea | 1977 to 1986 | 1.03 (0.46, 2.35) |
| Papua New Guinea | 1982 to 1991 | 1.00 (1.00, 1.00) |
| Papua New Guinea | 1987 to 1996 | 0.97 (0.32, 2.93) |
| Papua New Guinea | 1992 to 2001 | 0.92 (0.25, 3.39) |
| Papua New Guinea | 1997 to 2006 | 0.67 (0, 625.65) |
| Paraguay | 1952 to 1961 | 0.96 (0.09, 10.63) |
| Paraguay | 1957 to 1966 | 0.92 (0.16, 5.33) |
| Paraguay | 1962 to 1971 | 0.83 (0.14, 4.97) |
| Paraguay | 1967 to 1976 | 1.33 (0.35, 5.02) |
| Paraguay | 1972 to 1981 | 1.14 (0.33, 3.99) |
| Paraguay | 1977 to 1986 | 1.1 (0.33, 3.6) |
| Paraguay | 1982 to 1991 | 1.00 (1.00, 1.00) |
| Paraguay | 1987 to 1996 | 1.15 (0.28, 4.79) |
| Paraguay | 1992 to 2001 | 1.62 (0.37, 7.13) |
| Paraguay | 1997 to 2006 | 1.52 (0.21, 10.75) |
| Peru | 1952 to 1961 | 0.72 (0.21, 2.5) |
| Peru | 1957 to 1966 | 0.69 (0.27, 1.8) |
| Peru | 1962 to 1971 | 0.68 (0.29, 1.59) |
| Peru | 1967 to 1976 | 0.77 (0.35, 1.66) |
| Peru | 1972 to 1981 | 0.78 (0.39, 1.59) |
| Peru | 1977 to 1986 | 0.9 (0.47, 1.74) |
| Peru | 1982 to 1991 | 1.00 (1.00, 1.00) |
| Peru | 1987 to 1996 | 1.1 (0.5, 2.4) |
| Peru | 1992 to 2001 | 1.3 (0.53, 3.22) |
| Peru | 1997 to 2006 | 1.54 (0.47, 5.08) |
| Philippines | 1952 to 1961 | 1.1 (0.94, 1.3) |
| Philippines | 1957 to 1966 | 1.11 (0.98, 1.26) |
| Philippines | 1962 to 1971 | 1.09 (0.97, 1.23) |
| Philippines | 1967 to 1976 | 1.06 (0.94, 1.19) |
| Philippines | 1972 to 1981 | 1.02 (0.91, 1.14) |
| Philippines | 1977 to 1986 | 1.01 (0.91, 1.13) |
| Philippines | 1982 to 1991 | 1.00 (1.00, 1.00) |
| Philippines | 1987 to 1996 | 1 (0.87, 1.14) |
| Philippines | 1992 to 2001 | 1.03 (0.86, 1.24) |
| Philippines | 1997 to 2006 | 1.06 (0.82, 1.37) |
| Poland | 1952 to 1961 | 0.67 (0.42, 1.06) |
| Poland | 1957 to 1966 | 0.64 (0.43, 0.96) |
| Poland | 1962 to 1971 | 0.65 (0.44, 0.96) |
| Poland | 1967 to 1976 | 0.68 (0.48, 0.97) |
| Poland | 1972 to 1981 | 0.75 (0.54, 1.03) |
| Poland | 1977 to 1986 | 0.84 (0.63, 1.14) |
| Poland | 1982 to 1991 | 1.00 (1.00, 1.00) |
| Poland | 1987 to 1996 | 1.14 (0.76, 1.71) |
| Poland | 1992 to 2001 | 1.31 (0.75, 2.27) |
| Poland | 1997 to 2006 | 1.29 (0.52, 3.2) |
| Portugal | 1952 to 1961 | 1.02 (0.55, 1.87) |
| Portugal | 1957 to 1966 | 1.05 (0.64, 1.72) |
| Portugal | 1962 to 1971 | 1.11 (0.71, 1.75) |
| Portugal | 1967 to 1976 | 1.05 (0.69, 1.61) |
| Portugal | 1972 to 1981 | 0.99 (0.65, 1.5) |
| Portugal | 1977 to 1986 | 0.91 (0.59, 1.39) |
| Portugal | 1982 to 1991 | 1.00 (1.00, 1.00) |
| Portugal | 1987 to 1996 | 0.93 (0.53, 1.64) |
| Portugal | 1992 to 2001 | 0.94 (0.46, 1.91) |
| Portugal | 1997 to 2006 | 1.02 (0.36, 2.84) |
| Puerto Rico | 1952 to 1961 | 0.6 (0.14, 2.54) |
| Puerto Rico | 1957 to 1966 | 0.68 (0.24, 1.91) |
| Puerto Rico | 1962 to 1971 | 0.71 (0.29, 1.73) |
| Puerto Rico | 1967 to 1976 | 0.75 (0.33, 1.7) |
| Puerto Rico | 1972 to 1981 | 0.89 (0.41, 1.89) |
| Puerto Rico | 1977 to 1986 | 0.97 (0.46, 2.05) |
| Puerto Rico | 1982 to 1991 | 1.00 (1.00, 1.00) |
| Puerto Rico | 1987 to 1996 | 1.05 (0.42, 2.64) |
| Puerto Rico | 1992 to 2001 | 1.03 (0.31, 3.45) |
| Puerto Rico | 1997 to 2006 | 0.72 (0.08, 6.3) |
| Qatar | 1952 to 1961 | 0.91 (0.09, 9.68) |
| Qatar | 1957 to 1966 | 0.94 (0.18, 4.82) |
| Qatar | 1962 to 1971 | 0.71 (0.1, 5.04) |
| Qatar | 1967 to 1976 | 0.61 (0.1, 3.83) |
| Qatar | 1972 to 1981 | 0.75 (0.2, 2.89) |
| Qatar | 1977 to 1986 | 0.81 (0.3, 2.16) |
| Qatar | 1982 to 1991 | 1.00 (1.00, 1.00) |
| Qatar | 1987 to 1996 | 1.4 (0.43, 4.56) |
| Qatar | 1992 to 2001 | 1.89 (0.29, 12.54) |
| Qatar | 1997 to 2006 | 1.62 (0.08, 31.67) |
| Republic of Korea | 1952 to 1961 | 0.54 (0.37, 0.8) |
| Republic of Korea | 1957 to 1966 | 0.63 (0.46, 0.86) |
| Republic of Korea | 1962 to 1971 | 0.73 (0.55, 0.97) |
| Republic of Korea | 1967 to 1976 | 0.78 (0.6, 1.03) |
| Republic of Korea | 1972 to 1981 | 0.86 (0.66, 1.12) |
| Republic of Korea | 1977 to 1986 | 0.94 (0.73, 1.21) |
| Republic of Korea | 1982 to 1991 | 1.00 (1.00, 1.00) |
| Republic of Korea | 1987 to 1996 | 1.08 (0.77, 1.52) |
| Republic of Korea | 1992 to 2001 | 1.26 (0.81, 1.95) |
| Republic of Korea | 1997 to 2006 | 1.35 (0.66, 2.75) |
| Republic of Moldova | 1952 to 1961 | 0.84 (0.2, 3.44) |
| Republic of Moldova | 1957 to 1966 | 0.93 (0.28, 3.06) |
| Republic of Moldova | 1962 to 1971 | 1.16 (0.4, 3.39) |
| Republic of Moldova | 1967 to 1976 | 1 (0.36, 2.77) |
| Republic of Moldova | 1972 to 1981 | 0.9 (0.34, 2.38) |
| Republic of Moldova | 1977 to 1986 | 0.81 (0.3, 2.21) |
| Republic of Moldova | 1982 to 1991 | 1.00 (1.00, 1.00) |
| Republic of Moldova | 1987 to 1996 | 0.82 (0.24, 2.87) |
| Republic of Moldova | 1992 to 2001 | 0.75 (0.15, 3.82) |
| Republic of Moldova | 1997 to 2006 | 0.84 (0.09, 8) |
| Romania | 1952 to 1961 | 0.43 (0.25, 0.76) |
| Romania | 1957 to 1966 | 0.51 (0.32, 0.83) |
| Romania | 1962 to 1971 | 0.57 (0.37, 0.86) |
| Romania | 1967 to 1976 | 0.62 (0.42, 0.92) |
| Romania | 1972 to 1981 | 0.68 (0.47, 0.98) |
| Romania | 1977 to 1986 | 0.8 (0.56, 1.15) |
| Romania | 1982 to 1991 | 1.00 (1.00, 1.00) |
| Romania | 1987 to 1996 | 1.19 (0.73, 1.92) |
| Romania | 1992 to 2001 | 1.35 (0.72, 2.55) |
| Romania | 1997 to 2006 | 1.66 (0.69, 3.97) |
| Russian Federation | 1952 to 1961 | 0.81 (0.6, 1.1) |
| Russian Federation | 1957 to 1966 | 0.8 (0.61, 1.03) |
| Russian Federation | 1962 to 1971 | 0.86 (0.67, 1.11) |
| Russian Federation | 1967 to 1976 | 0.99 (0.79, 1.25) |
| Russian Federation | 1972 to 1981 | 1.13 (0.92, 1.39) |
| Russian Federation | 1977 to 1986 | 1.1 (0.9, 1.35) |
| Russian Federation | 1982 to 1991 | 1.00 (1.00, 1.00) |
| Russian Federation | 1987 to 1996 | 0.98 (0.73, 1.31) |
| Russian Federation | 1992 to 2001 | 0.99 (0.67, 1.46) |
| Russian Federation | 1997 to 2006 | 0.94 (0.53, 1.65) |
| Rwanda | 1952 to 1961 | 1.72 (0.91, 3.24) |
| Rwanda | 1957 to 1966 | 1.55 (0.93, 2.6) |
| Rwanda | 1962 to 1971 | 1.41 (0.88, 2.25) |
| Rwanda | 1967 to 1976 | 1.24 (0.79, 1.95) |
| Rwanda | 1972 to 1981 | 1.12 (0.74, 1.7) |
| Rwanda | 1977 to 1986 | 1.08 (0.73, 1.59) |
| Rwanda | 1982 to 1991 | 1.00 (1.00, 1.00) |
| Rwanda | 1987 to 1996 | 0.92 (0.57, 1.49) |
| Rwanda | 1992 to 2001 | 0.79 (0.44, 1.42) |
| Rwanda | 1997 to 2006 | 0.76 (0.36, 1.6) |
| Samoa | 1952 to 1961 | 1.34 (0.01, 129.7) |
| Samoa | 1957 to 1966 | 1.15 (0.01, 122.54) |
| Samoa | 1962 to 1971 | 1.05 (0.01, 76.1) |
| Samoa | 1967 to 1976 | 1.01 (0.02, 64.59) |
| Samoa | 1972 to 1981 | 1.05 (0.02, 58.13) |
| Samoa | 1977 to 1986 | 1.07 (0.02, 59.11) |
| Samoa | 1982 to 1991 | 1.00 (1.00, 1.00) |
| Samoa | 1987 to 1996 | 0.96 (0.01, 122.97) |
| Samoa | 1992 to 2001 | 0.96 (0, 293.72) |
| Samoa | 1997 to 2006 | 0.98 (0, 2192.45) |
| Saudi Arabia | 1952 to 1961 | 0.61 (0.43, 0.87) |
| Saudi Arabia | 1957 to 1966 | 0.58 (0.45, 0.76) |
| Saudi Arabia | 1962 to 1971 | 0.61 (0.47, 0.78) |
| Saudi Arabia | 1967 to 1976 | 0.68 (0.54, 0.85) |
| Saudi Arabia | 1972 to 1981 | 0.77 (0.64, 0.94) |
| Saudi Arabia | 1977 to 1986 | 0.86 (0.73, 1.02) |
| Saudi Arabia | 1982 to 1991 | 1.00 (1.00, 1.00) |
| Saudi Arabia | 1987 to 1996 | 1.12 (0.9, 1.4) |
| Saudi Arabia | 1992 to 2001 | 1.23 (0.91, 1.65) |
| Saudi Arabia | 1997 to 2006 | 1.27 (0.8, 2) |
| Senegal | 1952 to 1961 | 0.61 (0.06, 6.22) |
| Senegal | 1957 to 1966 | 0.57 (0.11, 3.08) |
| Senegal | 1962 to 1971 | 0.57 (0.11, 3) |
| Senegal | 1967 to 1976 | 0.8 (0.24, 2.67) |
| Senegal | 1972 to 1981 | 0.79 (0.24, 2.66) |
| Senegal | 1977 to 1986 | 0.92 (0.32, 2.67) |
| Senegal | 1982 to 1991 | 1.00 (1.00, 1.00) |
| Senegal | 1987 to 1996 | 0.97 (0.26, 3.67) |
| Senegal | 1992 to 2001 | 1.34 (0.32, 5.57) |
| Senegal | 1997 to 2006 | 1.19 (0.09, 15.36) |
| Serbia | 1952 to 1961 | 0.76 (0.26, 2.23) |
| Serbia | 1957 to 1966 | 0.7 (0.28, 1.74) |
| Serbia | 1962 to 1971 | 0.73 (0.31, 1.73) |
| Serbia | 1967 to 1976 | 0.77 (0.34, 1.75) |
| Serbia | 1972 to 1981 | 0.89 (0.41, 1.9) |
| Serbia | 1977 to 1986 | 0.91 (0.44, 1.91) |
| Serbia | 1982 to 1991 | 1.00 (1.00, 1.00) |
| Serbia | 1987 to 1996 | 1.09 (0.42, 2.88) |
| Serbia | 1992 to 2001 | 0.97 (0.25, 3.7) |
| Serbia | 1997 to 2006 | 0.7 (0.08, 6.13) |
| Seychelles | 1952 to 1961 | 0.15 (0, 264.33) |
| Seychelles | 1957 to 1966 | 1.26 (0.01, 133.64) |
| Seychelles | 1962 to 1971 | 1.15 (0.02, 83.24) |
| Seychelles | 1967 to 1976 | 1.09 (0.02, 69.28) |
| Seychelles | 1972 to 1981 | 1.05 (0.02, 58.03) |
| Seychelles | 1977 to 1986 | 1.02 (0.02, 56.32) |
| Seychelles | 1982 to 1991 | 1.00 (1.00, 1.00) |
| Seychelles | 1987 to 1996 | 1.01 (0.01, 129.82) |
| Seychelles | 1992 to 2001 | 1.06 (0, 322.02) |
| Seychelles | 1997 to 2006 | 1.09 (0, 2437.96) |
| Sierra Leone | 1952 to 1961 | 1.06 (0.07, 15.7) |
| Sierra Leone | 1957 to 1966 | 0.34 (0.02, 7.59) |
| Sierra Leone | 1962 to 1971 | 0.42 (0.04, 4.8) |
| Sierra Leone | 1967 to 1976 | 0.42 (0.05, 3.95) |
| Sierra Leone | 1972 to 1981 | 0.45 (0.06, 3.28) |
| Sierra Leone | 1977 to 1986 | 0.66 (0.13, 3.25) |
| Sierra Leone | 1982 to 1991 | 1.00 (1.00, 1.00) |
| Sierra Leone | 1987 to 1996 | 0.99 (0.15, 6.49) |
| Sierra Leone | 1992 to 2001 | 0.74 (0.05, 10.7) |
| Sierra Leone | 1997 to 2006 | 0.49 (0, 527.32) |
| Singapore | 1952 to 1961 | 1.81 (1.3, 2.53) |
| Singapore | 1957 to 1966 | 1.42 (1.08, 1.88) |
| Singapore | 1962 to 1971 | 1.16 (0.89, 1.51) |
| Singapore | 1967 to 1976 | 0.98 (0.76, 1.26) |
| Singapore | 1972 to 1981 | 0.83 (0.65, 1.06) |
| Singapore | 1977 to 1986 | 0.83 (0.66, 1.05) |
| Singapore | 1982 to 1991 | 1.00 (1.00, 1.00) |
| Singapore | 1987 to 1996 | 1.06 (0.79, 1.43) |
| Singapore | 1992 to 2001 | 1.25 (0.83, 1.9) |
| Singapore | 1997 to 2006 | 1.55 (0.83, 2.89) |
| Slovakia | 1952 to 1961 | 0.86 (0.25, 2.94) |
| Slovakia | 1957 to 1966 | 0.66 (0.22, 2) |
| Slovakia | 1962 to 1971 | 0.7 (0.25, 1.99) |
| Slovakia | 1967 to 1976 | 0.7 (0.27, 1.83) |
| Slovakia | 1972 to 1981 | 0.75 (0.3, 1.86) |
| Slovakia | 1977 to 1986 | 0.82 (0.35, 1.93) |
| Slovakia | 1982 to 1991 | 1.00 (1.00, 1.00) |
| Slovakia | 1987 to 1996 | 0.96 (0.28, 3.26) |
| Slovakia | 1992 to 2001 | 1.11 (0.21, 5.72) |
| Slovakia | 1997 to 2006 | 1.58 (0.16, 15.73) |
| Slovenia | 1952 to 1961 | 1.32 (0.21, 8.22) |
| Slovenia | 1957 to 1966 | 1.28 (0.28, 5.92) |
| Slovenia | 1962 to 1971 | 1.2 (0.29, 4.85) |
| Slovenia | 1967 to 1976 | 1.19 (0.32, 4.45) |
| Slovenia | 1972 to 1981 | 1.08 (0.3, 3.8) |
| Slovenia | 1977 to 1986 | 1.03 (0.29, 3.67) |
| Slovenia | 1982 to 1991 | 1.00 (1.00, 1.00) |
| Slovenia | 1987 to 1996 | 1.36 (0.3, 6.29) |
| Slovenia | 1992 to 2001 | 0.16 (0, 14.91) |
| Slovenia | 1997 to 2006 | 0.19 (0, 112.76) |
| Solomon Islands | 1952 to 1961 | 0.25 (0, 421.98) |
| Solomon Islands | 1957 to 1966 | 2.02 (0.02, 214.02) |
| Solomon Islands | 1962 to 1971 | 1.64 (0.02, 118.68) |
| Solomon Islands | 1967 to 1976 | 1.32 (0.02, 83.9) |
| Solomon Islands | 1972 to 1981 | 1.14 (0.02, 63.32) |
| Solomon Islands | 1977 to 1986 | 1.07 (0.02, 59.41) |
| Solomon Islands | 1982 to 1991 | 1.00 (1.00, 1.00) |
| Solomon Islands | 1987 to 1996 | 0.92 (0.01, 118.66) |
| Solomon Islands | 1992 to 2001 | 0.83 (0, 254.21) |
| Solomon Islands | 1997 to 2006 | 0.75 (0, 1689.5) |
| Somalia | 1952 to 1961 | 1.12 (0.57, 2.2) |
| Somalia | 1957 to 1966 | 1.06 (0.58, 1.92) |
| Somalia | 1962 to 1971 | 1.01 (0.58, 1.75) |
| Somalia | 1967 to 1976 | 0.96 (0.59, 1.56) |
| Somalia | 1972 to 1981 | 1 (0.66, 1.51) |
| Somalia | 1977 to 1986 | 1 (0.68, 1.49) |
| Somalia | 1982 to 1991 | 1.00 (1.00, 1.00) |
| Somalia | 1987 to 1996 | 0.96 (0.59, 1.56) |
| Somalia | 1992 to 2001 | 0.96 (0.55, 1.68) |
| Somalia | 1997 to 2006 | 0.93 (0.46, 1.86) |
| South Africa | 1952 to 1961 | 1.5 (0.91, 2.46) |
| South Africa | 1957 to 1966 | 1.34 (0.9, 1.99) |
| South Africa | 1962 to 1971 | 1.28 (0.9, 1.83) |
| South Africa | 1967 to 1976 | 1.29 (0.93, 1.79) |
| South Africa | 1972 to 1981 | 1.19 (0.87, 1.64) |
| South Africa | 1977 to 1986 | 1.09 (0.8, 1.48) |
| South Africa | 1982 to 1991 | 1.00 (1.00, 1.00) |
| South Africa | 1987 to 1996 | 0.99 (0.67, 1.47) |
| South Africa | 1992 to 2001 | 1.02 (0.63, 1.68) |
| South Africa | 1997 to 2006 | 0.98 (0.48, 1.97) |
| South Sudan | 1952 to 1961 | 0.92 (0.36, 2.38) |
| South Sudan | 1957 to 1966 | 0.9 (0.42, 1.95) |
| South Sudan | 1962 to 1971 | 0.93 (0.47, 1.83) |
| South Sudan | 1967 to 1976 | 0.91 (0.48, 1.73) |
| South Sudan | 1972 to 1981 | 0.93 (0.52, 1.68) |
| South Sudan | 1977 to 1986 | 0.95 (0.54, 1.69) |
| South Sudan | 1982 to 1991 | 1.00 (1.00, 1.00) |
| South Sudan | 1987 to 1996 | 1.09 (0.55, 2.17) |
| South Sudan | 1992 to 2001 | 1.13 (0.5, 2.54) |
| South Sudan | 1997 to 2006 | 1.3 (0.49, 3.45) |
| Spain | 1952 to 1961 | 1.56 (1.23, 1.98) |
| Spain | 1957 to 1966 | 1.55 (1.28, 1.87) |
| Spain | 1962 to 1971 | 1.42 (1.19, 1.69) |
| Spain | 1967 to 1976 | 1.23 (1.04, 1.46) |
| Spain | 1972 to 1981 | 1.09 (0.92, 1.28) |
| Spain | 1977 to 1986 | 1.04 (0.88, 1.23) |
| Spain | 1982 to 1991 | 1.00 (1.00, 1.00) |
| Spain | 1987 to 1996 | 0.92 (0.73, 1.16) |
| Spain | 1992 to 2001 | 0.8 (0.59, 1.09) |
| Spain | 1997 to 2006 | 0.69 (0.44, 1.08) |
| Sri Lanka | 1952 to 1961 | 0.88 (0.59, 1.31) |
| Sri Lanka | 1957 to 1966 | 0.91 (0.67, 1.24) |
| Sri Lanka | 1962 to 1971 | 0.95 (0.72, 1.26) |
| Sri Lanka | 1967 to 1976 | 0.92 (0.7, 1.21) |
| Sri Lanka | 1972 to 1981 | 0.92 (0.71, 1.2) |
| Sri Lanka | 1977 to 1986 | 0.96 (0.75, 1.23) |
| Sri Lanka | 1982 to 1991 | 1.00 (1.00, 1.00) |
| Sri Lanka | 1987 to 1996 | 1.06 (0.76, 1.47) |
| Sri Lanka | 1992 to 2001 | 1.13 (0.75, 1.71) |
| Sri Lanka | 1997 to 2006 | 1.1 (0.61, 1.98) |
| Sudan | 1952 to 1961 | 1.46 (0.71, 3.02) |
| Sudan | 1957 to 1966 | 1.26 (0.72, 2.23) |
| Sudan | 1962 to 1971 | 1.17 (0.71, 1.92) |
| Sudan | 1967 to 1976 | 1.12 (0.7, 1.79) |
| Sudan | 1972 to 1981 | 1.07 (0.69, 1.66) |
| Sudan | 1977 to 1986 | 1.01 (0.66, 1.55) |
| Sudan | 1982 to 1991 | 1.00 (1.00, 1.00) |
| Sudan | 1987 to 1996 | 1 (0.6, 1.65) |
| Sudan | 1992 to 2001 | 1.03 (0.56, 1.88) |
| Sudan | 1997 to 2006 | 0.95 (0.43, 2.12) |
| Suriname | 1952 to 1961 | 0.16 (0, 277.27) |
| Suriname | 1957 to 1966 | 0.4 (0, 128.16) |
| Suriname | 1962 to 1971 | 0.6 (0, 84.67) |
| Suriname | 1967 to 1976 | 1.41 (0.02, 92.04) |
| Suriname | 1972 to 1981 | 1.32 (0.02, 74.06) |
| Suriname | 1977 to 1986 | 1.24 (0.02, 69.49) |
| Suriname | 1982 to 1991 | 1.00 (1.00, 1.00) |
| Suriname | 1987 to 1996 | 0.96 (0.01, 125.06) |
| Suriname | 1992 to 2001 | 0.91 (0, 279.88) |
| Suriname | 1997 to 2006 | 0.86 (0, 1943.37) |
| Sweden | 1952 to 1961 | 1.37 (0.64, 2.92) |
| Sweden | 1957 to 1966 | 1.42 (0.86, 2.34) |
| Sweden | 1962 to 1971 | 1.18 (0.75, 1.86) |
| Sweden | 1967 to 1976 | 1.05 (0.66, 1.68) |
| Sweden | 1972 to 1981 | 1.07 (0.67, 1.7) |
| Sweden | 1977 to 1986 | 1 (0.63, 1.57) |
| Sweden | 1982 to 1991 | 1.00 (1.00, 1.00) |
| Sweden | 1987 to 1996 | 1.04 (0.6, 1.79) |
| Sweden | 1992 to 2001 | 0.87 (0.34, 2.19) |
| Sweden | 1997 to 2006 | 0.66 (0.14, 3.07) |
| Switzerland | 1952 to 1961 | 3.04 (1.32, 7) |
| Switzerland | 1957 to 1966 | 2.59 (1.32, 5.1) |
| Switzerland | 1962 to 1971 | 2.21 (1.19, 4.11) |
| Switzerland | 1967 to 1976 | 1.85 (1.02, 3.33) |
| Switzerland | 1972 to 1981 | 1.43 (0.79, 2.59) |
| Switzerland | 1977 to 1986 | 1.21 (0.67, 2.2) |
| Switzerland | 1982 to 1991 | 1.00 (1.00, 1.00) |
| Switzerland | 1987 to 1996 | 0.83 (0.38, 1.84) |
| Switzerland | 1992 to 2001 | 0.6 (0.2, 1.84) |
| Switzerland | 1997 to 2006 | 0.74 (0.15, 3.53) |
| Syrian Arab Republic | 1952 to 1961 | 1 (0.23, 4.27) |
| Syrian Arab Republic | 1957 to 1966 | 1.05 (0.37, 3.03) |
| Syrian Arab Republic | 1962 to 1971 | 0.86 (0.33, 2.22) |
| Syrian Arab Republic | 1967 to 1976 | 0.92 (0.4, 2.14) |
| Syrian Arab Republic | 1972 to 1981 | 0.87 (0.4, 1.91) |
| Syrian Arab Republic | 1977 to 1986 | 0.92 (0.44, 1.95) |
| Syrian Arab Republic | 1982 to 1991 | 1.00 (1.00, 1.00) |
| Syrian Arab Republic | 1987 to 1996 | 1.15 (0.43, 3.08) |
| Syrian Arab Republic | 1992 to 2001 | 1.15 (0.37, 3.54) |
| Syrian Arab Republic | 1997 to 2006 | 1.13 (0.27, 4.8) |
| Taiwan (Province of China) | 1952 to 1961 | 1.4 (1.26, 1.55) |
| Taiwan (Province of China) | 1957 to 1966 | 1.4 (1.28, 1.52) |
| Taiwan (Province of China) | 1962 to 1971 | 1.38 (1.27, 1.5) |
| Taiwan (Province of China) | 1967 to 1976 | 1.35 (1.24, 1.47) |
| Taiwan (Province of China) | 1972 to 1981 | 1.29 (1.19, 1.4) |
| Taiwan (Province of China) | 1977 to 1986 | 1.16 (1.07, 1.26) |
| Taiwan (Province of China) | 1982 to 1991 | 1.00 (1.00, 1.00) |
| Taiwan (Province of China) | 1987 to 1996 | 0.89 (0.79, 1.01) |
| Taiwan (Province of China) | 1992 to 2001 | 0.87 (0.73, 1.03) |
| Taiwan (Province of China) | 1997 to 2006 | 0.83 (0.63, 1.09) |
| Tajikistan | 1952 to 1961 | 1.34 (0.39, 4.68) |
| Tajikistan | 1957 to 1966 | 1.21 (0.49, 3) |
| Tajikistan | 1962 to 1971 | 1.27 (0.6, 2.71) |
| Tajikistan | 1967 to 1976 | 1.19 (0.58, 2.44) |
| Tajikistan | 1972 to 1981 | 1.09 (0.56, 2.12) |
| Tajikistan | 1977 to 1986 | 1.09 (0.58, 2.05) |
| Tajikistan | 1982 to 1991 | 1.00 (1.00, 1.00) |
| Tajikistan | 1987 to 1996 | 0.94 (0.46, 1.93) |
| Tajikistan | 1992 to 2001 | 0.83 (0.34, 2.05) |
| Tajikistan | 1997 to 2006 | 0.77 (0.23, 2.62) |
| Thailand | 1952 to 1961 | 0.63 (0.54, 0.74) |
| Thailand | 1957 to 1966 | 0.64 (0.57, 0.72) |
| Thailand | 1962 to 1971 | 0.75 (0.67, 0.83) |
| Thailand | 1967 to 1976 | 0.84 (0.76, 0.94) |
| Thailand | 1972 to 1981 | 0.92 (0.83, 1.02) |
| Thailand | 1977 to 1986 | 0.94 (0.86, 1.04) |
| Thailand | 1982 to 1991 | 1.00 (1.00, 1.00) |
| Thailand | 1987 to 1996 | 1.07 (0.93, 1.22) |
| Thailand | 1992 to 2001 | 1.1 (0.92, 1.31) |
| Thailand | 1997 to 2006 | 1.15 (0.89, 1.49) |
| Timor-Leste | 1952 to 1961 | 1.29 (0.15, 11.33) |
| Timor-Leste | 1957 to 1966 | 0.74 (0.11, 4.79) |
| Timor-Leste | 1962 to 1971 | 0.88 (0.17, 4.7) |
| Timor-Leste | 1967 to 1976 | 0.95 (0.16, 5.77) |
| Timor-Leste | 1972 to 1981 | 1.07 (0.19, 5.86) |
| Timor-Leste | 1977 to 1986 | 0.99 (0.2, 4.77) |
| Timor-Leste | 1982 to 1991 | 1.00 (1.00, 1.00) |
| Timor-Leste | 1987 to 1996 | 1.24 (0.16, 9.39) |
| Timor-Leste | 1992 to 2001 | 2.72 (0.21, 35.32) |
| Timor-Leste | 1997 to 2006 | 3.43 (0.1, 116.01) |
| Togo | 1952 to 1961 | 0.88 (0.07, 11.22) |
| Togo | 1957 to 1966 | 0.9 (0.14, 5.73) |
| Togo | 1962 to 1971 | 0.79 (0.11, 5.59) |
| Togo | 1967 to 1976 | 0.83 (0.17, 4.11) |
| Togo | 1972 to 1981 | 0.93 (0.19, 4.45) |
| Togo | 1977 to 1986 | 0.69 (0.15, 3.19) |
| Togo | 1982 to 1991 | 1.00 (1.00, 1.00) |
| Togo | 1987 to 1996 | 0.79 (0.13, 4.7) |
| Togo | 1992 to 2001 | 0.46 (0.04, 4.89) |
| Togo | 1997 to 2006 | 0.4 (0, 411.47) |
| Trinidad and Tobago | 1952 to 1961 | 0.06 (0, 46.15) |
| Trinidad and Tobago | 1957 to 1966 | 0.38 (0.01, 9.78) |
| Trinidad and Tobago | 1962 to 1971 | 0.17 (0.01, 3.57) |
| Trinidad and Tobago | 1967 to 1976 | 0.28 (0.03, 3.06) |
| Trinidad and Tobago | 1972 to 1981 | 0.42 (0.06, 2.93) |
| Trinidad and Tobago | 1977 to 1986 | 0.51 (0.09, 2.92) |
| Trinidad and Tobago | 1982 to 1991 | 1.00 (1.00, 1.00) |
| Trinidad and Tobago | 1987 to 1996 | 1.21 (0.11, 13.56) |
| Trinidad and Tobago | 1992 to 2001 | 0.75 (0.01, 94.93) |
| Trinidad and Tobago | 1997 to 2006 | 1.61 (0, 1873.37) |
| Tunisia | 1952 to 1961 | 0.71 (0.5, 1.01) |
| Tunisia | 1957 to 1966 | 0.7 (0.53, 0.91) |
| Tunisia | 1962 to 1971 | 0.75 (0.59, 0.95) |
| Tunisia | 1967 to 1976 | 0.79 (0.63, 0.99) |
| Tunisia | 1972 to 1981 | 0.86 (0.7, 1.06) |
| Tunisia | 1977 to 1986 | 0.93 (0.76, 1.13) |
| Tunisia | 1982 to 1991 | 1.00 (1.00, 1.00) |
| Tunisia | 1987 to 1996 | 1.09 (0.84, 1.42) |
| Tunisia | 1992 to 2001 | 1.21 (0.86, 1.7) |
| Tunisia | 1997 to 2006 | 1.28 (0.79, 2.05) |
| Turkey | 1952 to 1961 | 0.95 (0.73, 1.25) |
| Turkey | 1957 to 1966 | 0.88 (0.71, 1.08) |
| Turkey | 1962 to 1971 | 0.86 (0.71, 1.03) |
| Turkey | 1967 to 1976 | 0.87 (0.73, 1.03) |
| Turkey | 1972 to 1981 | 0.89 (0.76, 1.04) |
| Turkey | 1977 to 1986 | 0.94 (0.81, 1.1) |
| Turkey | 1982 to 1991 | 1.00 (1.00, 1.00) |
| Turkey | 1987 to 1996 | 1.06 (0.88, 1.28) |
| Turkey | 1992 to 2001 | 1.1 (0.88, 1.38) |
| Turkey | 1997 to 2006 | 1.12 (0.82, 1.52) |
| Turkmenistan | 1952 to 1961 | 0.56 (0.11, 3.04) |
| Turkmenistan | 1957 to 1966 | 0.59 (0.18, 2) |
| Turkmenistan | 1962 to 1971 | 0.68 (0.25, 1.88) |
| Turkmenistan | 1967 to 1976 | 0.82 (0.33, 2.04) |
| Turkmenistan | 1972 to 1981 | 0.85 (0.37, 1.95) |
| Turkmenistan | 1977 to 1986 | 0.93 (0.42, 2.07) |
| Turkmenistan | 1982 to 1991 | 1.00 (1.00, 1.00) |
| Turkmenistan | 1987 to 1996 | 1.11 (0.46, 2.67) |
| Turkmenistan | 1992 to 2001 | 1.12 (0.38, 3.33) |
| Turkmenistan | 1997 to 2006 | 1.23 (0.28, 5.42) |
| Uganda | 1952 to 1961 | 1.13 (0.82, 1.57) |
| Uganda | 1957 to 1966 | 1.06 (0.82, 1.36) |
| Uganda | 1962 to 1971 | 1.03 (0.83, 1.28) |
| Uganda | 1967 to 1976 | 1.03 (0.85, 1.26) |
| Uganda | 1972 to 1981 | 1.02 (0.85, 1.22) |
| Uganda | 1977 to 1986 | 0.99 (0.83, 1.18) |
| Uganda | 1982 to 1991 | 1.00 (1.00, 1.00) |
| Uganda | 1987 to 1996 | 0.99 (0.81, 1.21) |
| Uganda | 1992 to 2001 | 0.97 (0.77, 1.23) |
| Uganda | 1997 to 2006 | 0.92 (0.68, 1.24) |
| Ukraine | 1952 to 1961 | 0.6 (0.37, 0.98) |
| Ukraine | 1957 to 1966 | 0.7 (0.47, 1.05) |
| Ukraine | 1962 to 1971 | 0.81 (0.55, 1.18) |
| Ukraine | 1967 to 1976 | 0.91 (0.64, 1.29) |
| Ukraine | 1972 to 1981 | 0.99 (0.71, 1.37) |
| Ukraine | 1977 to 1986 | 0.98 (0.72, 1.35) |
| Ukraine | 1982 to 1991 | 1.00 (1.00, 1.00) |
| Ukraine | 1987 to 1996 | 1.05 (0.68, 1.61) |
| Ukraine | 1992 to 2001 | 1.18 (0.67, 2.08) |
| Ukraine | 1997 to 2006 | 1.33 (0.6, 2.97) |
| United Arab Emirates | 1952 to 1961 | 1.12 (0.4, 3.1) |
| United Arab Emirates | 1957 to 1966 | 0.94 (0.42, 2.12) |
| United Arab Emirates | 1962 to 1971 | 0.92 (0.48, 1.79) |
| United Arab Emirates | 1967 to 1976 | 0.89 (0.5, 1.61) |
| United Arab Emirates | 1972 to 1981 | 0.86 (0.53, 1.4) |
| United Arab Emirates | 1977 to 1986 | 0.81 (0.53, 1.22) |
| United Arab Emirates | 1982 to 1991 | 1.00 (1.00, 1.00) |
| United Arab Emirates | 1987 to 1996 | 1.25 (0.67, 2.33) |
| United Arab Emirates | 1992 to 2001 | 1.23 (0.48, 3.15) |
| United Arab Emirates | 1997 to 2006 | 0.87 (0.17, 4.32) |
| United Kingdom | 1952 to 1961 | 0.73 (0.58, 0.93) |
| United Kingdom | 1957 to 1966 | 0.78 (0.66, 0.93) |
| United Kingdom | 1962 to 1971 | 0.85 (0.73, 0.99) |
| United Kingdom | 1967 to 1976 | 0.94 (0.82, 1.08) |
| United Kingdom | 1972 to 1981 | 0.98 (0.86, 1.13) |
| United Kingdom | 1977 to 1986 | 1 (0.88, 1.14) |
| United Kingdom | 1982 to 1991 | 1.00 (1.00, 1.00) |
| United Kingdom | 1987 to 1996 | 1 (0.85, 1.18) |
| United Kingdom | 1992 to 2001 | 0.9 (0.73, 1.12) |
| United Kingdom | 1997 to 2006 | 0.86 (0.62, 1.18) |
| United Republic of Tanzania | 1952 to 1961 | 1.09 (0.75, 1.58) |
| United Republic of Tanzania | 1957 to 1966 | 1.05 (0.78, 1.41) |
| United Republic of Tanzania | 1962 to 1971 | 1.02 (0.78, 1.32) |
| United Republic of Tanzania | 1967 to 1976 | 1.01 (0.79, 1.29) |
| United Republic of Tanzania | 1972 to 1981 | 1 (0.8, 1.25) |
| United Republic of Tanzania | 1977 to 1986 | 1 (0.8, 1.23) |
| United Republic of Tanzania | 1982 to 1991 | 1.00 (1.00, 1.00) |
| United Republic of Tanzania | 1987 to 1996 | 1 (0.78, 1.3) |
| United Republic of Tanzania | 1992 to 2001 | 1.01 (0.74, 1.37) |
| United Republic of Tanzania | 1997 to 2006 | 0.98 (0.66, 1.46) |
| United States of America | 1952 to 1961 | 1.06 (0.95, 1.18) |
| United States of America | 1957 to 1966 | 1.01 (0.93, 1.1) |
| United States of America | 1962 to 1971 | 0.93 (0.86, 1.01) |
| United States of America | 1967 to 1976 | 0.93 (0.86, 1.01) |
| United States of America | 1972 to 1981 | 0.98 (0.91, 1.06) |
| United States of America | 1977 to 1986 | 0.99 (0.92, 1.06) |
| United States of America | 1982 to 1991 | 1.00 (1.00, 1.00) |
| United States of America | 1987 to 1996 | 0.94 (0.86, 1.02) |
| United States of America | 1992 to 2001 | 0.77 (0.68, 0.87) |
| United States of America | 1997 to 2006 | 0.7 (0.58, 0.85) |
| Uruguay | 1952 to 1961 | 0.68 (0.11, 4.33) |
| Uruguay | 1957 to 1966 | 0.8 (0.2, 3.24) |
| Uruguay | 1962 to 1971 | 0.85 (0.23, 3.09) |
| Uruguay | 1967 to 1976 | 0.84 (0.26, 2.7) |
| Uruguay | 1972 to 1981 | 0.82 (0.27, 2.54) |
| Uruguay | 1977 to 1986 | 0.99 (0.34, 2.92) |
| Uruguay | 1982 to 1991 | 1.00 (1.00, 1.00) |
| Uruguay | 1987 to 1996 | 1.52 (0.46, 4.95) |
| Uruguay | 1992 to 2001 | 1.7 (0.42, 6.87) |
| Uruguay | 1997 to 2006 | 1.03 (0.1, 10.75) |
| Uzbekistan | 1952 to 1961 | 0.79 (0.4, 1.57) |
| Uzbekistan | 1957 to 1966 | 0.79 (0.48, 1.28) |
| Uzbekistan | 1962 to 1971 | 0.84 (0.56, 1.26) |
| Uzbekistan | 1967 to 1976 | 0.9 (0.62, 1.31) |
| Uzbekistan | 1972 to 1981 | 0.97 (0.69, 1.36) |
| Uzbekistan | 1977 to 1986 | 0.96 (0.7, 1.33) |
| Uzbekistan | 1982 to 1991 | 1.00 (1.00, 1.00) |
| Uzbekistan | 1987 to 1996 | 1.1 (0.77, 1.59) |
| Uzbekistan | 1992 to 2001 | 1.22 (0.78, 1.92) |
| Uzbekistan | 1997 to 2006 | 1.15 (0.61, 2.18) |
| Venezuela (Bolivarian Republic of) | 1952 to 1961 | 0.5 (0.19, 1.27) |
| Venezuela (Bolivarian Republic of) | 1957 to 1966 | 0.52 (0.27, 1.02) |
| Venezuela (Bolivarian Republic of) | 1962 to 1971 | 0.53 (0.3, 0.95) |
| Venezuela (Bolivarian Republic of) | 1967 to 1976 | 0.59 (0.36, 0.97) |
| Venezuela (Bolivarian Republic of) | 1972 to 1981 | 0.68 (0.44, 1.06) |
| Venezuela (Bolivarian Republic of) | 1977 to 1986 | 0.82 (0.54, 1.23) |
| Venezuela (Bolivarian Republic of) | 1982 to 1991 | 1.00 (1.00, 1.00) |
| Venezuela (Bolivarian Republic of) | 1987 to 1996 | 1.2 (0.76, 1.89) |
| Venezuela (Bolivarian Republic of) | 1992 to 2001 | 1.5 (0.85, 2.65) |
| Venezuela (Bolivarian Republic of) | 1997 to 2006 | 1.63 (0.72, 3.68) |
| Viet Nam | 1952 to 1961 | 0.55 (0.48, 0.62) |
| Viet Nam | 1957 to 1966 | 0.6 (0.55, 0.66) |
| Viet Nam | 1962 to 1971 | 0.68 (0.62, 0.73) |
| Viet Nam | 1967 to 1976 | 0.74 (0.68, 0.8) |
| Viet Nam | 1972 to 1981 | 0.79 (0.73, 0.85) |
| Viet Nam | 1977 to 1986 | 0.87 (0.81, 0.93) |
| Viet Nam | 1982 to 1991 | 1.00 (1.00, 1.00) |
| Viet Nam | 1987 to 1996 | 1.16 (1.06, 1.26) |
| Viet Nam | 1992 to 2001 | 1.35 (1.21, 1.51) |
| Viet Nam | 1997 to 2006 | 1.54 (1.33, 1.78) |
| Yemen | 1952 to 1961 | 1.41 (0.52, 3.81) |
| Yemen | 1957 to 1966 | 1.18 (0.52, 2.65) |
| Yemen | 1962 to 1971 | 0.98 (0.45, 2.12) |
| Yemen | 1967 to 1976 | 0.99 (0.49, 2.03) |
| Yemen | 1972 to 1981 | 0.98 (0.53, 1.84) |
| Yemen | 1977 to 1986 | 1 (0.56, 1.78) |
| Yemen | 1982 to 1991 | 1.00 (1.00, 1.00) |
| Yemen | 1987 to 1996 | 1.03 (0.49, 2.16) |
| Yemen | 1992 to 2001 | 0.95 (0.35, 2.6) |
| Yemen | 1997 to 2006 | 0.86 (0.22, 3.41) |
| Zambia | 1952 to 1961 | 0.53 (0.28, 1) |
| Zambia | 1957 to 1966 | 0.56 (0.34, 0.92) |
| Zambia | 1962 to 1971 | 0.66 (0.43, 1.02) |
| Zambia | 1967 to 1976 | 0.77 (0.52, 1.13) |
| Zambia | 1972 to 1981 | 0.84 (0.6, 1.19) |
| Zambia | 1977 to 1986 | 0.92 (0.67, 1.25) |
| Zambia | 1982 to 1991 | 1.00 (1.00, 1.00) |
| Zambia | 1987 to 1996 | 1.09 (0.75, 1.57) |
| Zambia | 1992 to 2001 | 1.14 (0.73, 1.78) |
| Zambia | 1997 to 2006 | 1.2 (0.67, 2.13) |
| Zimbabwe | 1952 to 1961 | 0.47 (0.16, 1.32) |
| Zimbabwe | 1957 to 1966 | 0.48 (0.21, 1.09) |
| Zimbabwe | 1962 to 1971 | 0.58 (0.29, 1.13) |
| Zimbabwe | 1967 to 1976 | 0.67 (0.37, 1.22) |
| Zimbabwe | 1972 to 1981 | 0.78 (0.46, 1.32) |
| Zimbabwe | 1977 to 1986 | 0.88 (0.54, 1.44) |
| Zimbabwe | 1982 to 1991 | 1.00 (1.00, 1.00) |
| Zimbabwe | 1987 to 1996 | 1.15 (0.63, 2.11) |
| Zimbabwe | 1992 to 2001 | 1.43 (0.7, 2.9) |
| Zimbabwe | 1997 to 2006 | 1.63 (0.65, 4.12) |
